# Redox Imbalance and Methylation Disturbances in Early Childhood Obesity

**DOI:** 10.1155/2021/2207125

**Published:** 2021-08-17

**Authors:** Pedro Barbosa, Stepan Melnyk, Sirish C. Bennuri, Leanna Delhey, Andreia Reis, Gabriela R. Moura, Elisabet Børsheim, Shannon Rose, Eugenia Carvalho

**Affiliations:** ^1^PhD Programme in Experimental Biology and Biomedicine, Institute for Interdisciplinary Research (IIIUC), University of Coimbra, Coimbra, Portugal; ^2^Center for Neuroscience and Cell Biology, University of Coimbra, Coimbra, Portugal; ^3^Institute for Interdisciplinary Research, University of Coimbra, Coimbra, Portugal; ^4^Arkansas Children's Research Institute, Little Rock, AR, USA; ^5^Department of Epidemiology, University of Arkansas for Medical Sciences, Little Rock, AR, USA; ^6^Institute of Biomedicine (iBiMED) & Department of Medical Sciences (DCM), University of Aveiro, Aveiro, Portugal; ^7^Arkansas Children's Nutrition Center, Little Rock, AR, USA; ^8^Department of Pediatrics, University of Arkansas for Medical Sciences, Little Rock, AR, USA; ^9^Department of Geriatrics, University of Arkansas for Medical Sciences, Little Rock, AR, USA

## Abstract

Obesity is increasing worldwide in prepubertal children, reducing the age of onset of associated comorbidities, including type 2 diabetes. Sulfur-containing amino acids, methionine, cysteine, and their derivatives play important roles in the transmethylation and transsulfuration pathways. Dysregulation of these pathways leads to alterations in the cellular methylation patterns and an imbalanced redox state. Therefore, we tested the hypothesis that one-carbon metabolism is already dysregulated in prepubertal children with obesity. Peripheral blood was collected from 64 children, and the plasma metabolites from transmethylation and transsulfuration pathways were quantified by HPLC. The cohort was stratified by BMI z-scores and HOMA-IR indices into healthy lean (HL), healthy obese (HO), and unhealthy obese (UHO). Fasting insulin levels were higher in the HO group compared to the HL, while the UHO had the highest. All groups presented normal fasting glycemia. Furthermore, high-density lipoprotein (HDL) was lower while triglycerides and lactate levels were higher in the UHO compared to HO subjects. S-adenosylhomocysteine (SAH) and total homocysteine levels were increased in the HO group compared to HL. Additionally, glutathione metabolism was also altered. Free cystine and oxidized glutathione (GSSG) were increased in the HO as compared to HL subjects. Importantly, the adipocyte secretory function was already compromised at this young age. Elevated circulating leptin and decreased adiponectin levels were observed in the UHO as compared to the HO subjects. Some of these alterations were concomitant with alterations in the DNA methylation patterns in the obese group, independent of the impaired insulin levels. In conclusion, our study informs on novel and important metabolic alterations in the transmethylation and the transsulfuration pathways in the early stages of obesity. Moreover, the altered secretory function of the adipocyte very early in life may be relevant in identifying early metabolic markers of disease that may inform on the increased risk for specific future comorbidities in this population.

## 1. Introduction

Obesity is a rapidly growing epidemic that is contributing to the significant increase in metabolic diseases worldwide. It is characterized by excess adipose tissue expansion and is associated with low-grade inflammation and metabolic dysfunction [1]. The continuous release of proinflammatory cytokines [1] and adipokines (e.g., leptin) by dysregulated adipose tissue may contribute to the obesity-associated inflammation [2]. It is thought that chronic low-grade inflammation induces chronic oxidative stress, and that both contribute to the obesity-related insulin resistance (IR) and type 2 diabetes (T2D) development [3]. Due to the drastic increase in early childhood obesity, the journey to T2D development is starting earlier in life. This in turn increases the risk for other severe health complications over the lifespan, such as hypertension, cardiovascular diseases (CVD), retinopathy, and neuropathy, that appear to increase as the age of T2D onset decreases [4–6]. Further, there are several important differences in the pathophysiology of obesity-associated comorbidities in adults compared to children, including early *β*-cell decline and time to T2D treatment failure, as well as the lack of appropriate pharmacological medications approved for earlier ages, and longer duration of the disease [7–10].

Early alterations in the redox and methylation status that are associated with obesity may play a significant role in the early onset of metabolic disturbances in children with obesity. The thiol group plays an important role in biological systems [11]. It appears in the sulfur-containing amino acids methionine and cysteine, and their derivatives, such as glutathione (GSH) and other low molecular weight intermediates in the transmethylation and transsulfuration pathways [11, 12], also known as aminothiols. Thiols are responsible for scavenging reactive oxygen species (ROS) and maintaining redox homeostasis [13]. In particular, cysteine is primarily responsible for maintaining the redox state in plasma [14], while GSH maintains intracellular redox homeostasis, acting directly or indirectly through enzymatic activity [13]. Recent studies postulate dysfunction in the redox homeostasis in obese children [15, 16]. Lechuga-Sancho et al. [15] have identified an altered oxidative status in erythrocytes from obesity-associated insulin resistant children, even before those changes occurred in plasma. Besides, Zalewska et al. [16] reported alterations in the saliva redox status followed by higher oxidative damage in obese when compared to overweight children.

Transmethylation and transsulfuration intermediates are also critically important for methylation of DNA, proteins, and lipids [14] with methionine-derived S-adenosylmethionine (SAM), being the primary methyl group donor [17]. Imbalance in the transmethylation and transsulfuration pathways is linked with obesity-related inflammation [18]. It has also been shown that high levels of circulating homocysteine, resulting from the S-adenosylhomocysteine (SAH) degradation, are linked to an increased oxidation status in circulation [19]. Moreover, the hyperhomocysteinemia resulting from the imbalance of transsulfuration and transmethylation metabolites has been linked to further risk of obesity-associated CVD, such as atherosclerosis [18, 19]. Interestingly, a study conducted in mice suggested that high levels of SAH in the circulation could be involved with alterations at the epigenetic levels by inhibiting the DNA methyltransferase enzymes. The same study also indicated a possible relation between high levels of SAH and endothelial dysfunction [20].

One-carbon metabolism pathways, including those described above, have been implicated in important metabolic processes that include redox defenses and epigenetic alterations, which are both altered in obesity [11]. However, it is not known how soon this can happen in life and whether these pathways, if becoming altered in prepubertal children with obesity, can facilitate the early onset of obesity-related comorbidities.

Therefore, the present study is mainly aimed at testing the hypothesis that one-carbon metabolism perturbation is already present in the early stages of obesity development, in prepubertal children. Therefore, transsulfuration and transmethylation metabolite levels were quantified and related with their systemic oxidative stress, genomic methylation status, and inflammatory marker levels in children of normal weight or with overweight/obesity.

## 2. Material and Methods

### 2.1. Study Cohort

A group of 64 prepubertal children (5-9 years old, Tables 1 and 2 from Results) were recruited after approval of the study by the Institutional Review Board (IRB) (protocol number 206164) at the University of Arkansas for Medical Science and following the guidelines of Declaration of Helsinki (1964). This clinical study was registered at ClinicalTrials.gov (NCT03323294). The inclusion criteria were age 5-9 years at the date of the visit (i.e., 5- <10 years), and the exclusion criteria were the presence of known chronic illnesses/disorders that might affect study outcome measures, such as type 1 diabetes mellitus, neurologic, developmental, endocrine, hepatic, autoimmune, cardiac, and renal disorders; use of any medication could affect study outcomes, e.g., antipsychotics, thyroid hormone replacement therapy, inhalation/oral steroids, insulin, anabolic drugs and stimulants, or being classified as underweight based on the CDC growth charts (http://www.cdc.gov/growthcharts).

Anthropometric variables were collected for all study participants and sex (male/female), age (years), weight (kg), height (cm), and waist circumference (cm) were included (Tables 1 and 2). For data analyses, children with an age ≥ 9 years and 6 months, but <10 years, were considered 10 years old (Tables 1 and 2). The weight was measured using a calibrated Avery Berkel, HL122 Series Platform Scale (Dynamic Scales, Terre Haute, IN, USA) wearing minimal clothing, while height was obtained using a stadiometer (Novel Products, Rockton, IL, USA). The waist circumference was measured as reported previously [21].

Body mass index (BMI) was calculated from body mass and height as kg/m^2^ and adjusted for age and sex according to the Centers for Diseases Control and Prevention (http://www.cdc.gov/growthcharts). The participants were considered overweight or obese if their age- and sex-adjusted BMI was above the 85th percentile (i.e., BMI z − score (BMIz) > 1.04). Although during the stratification present in Statistical Analysis, all the participants with overweight and obesity were included in obese groups. Clinical outcomes such as systolic and diastolic blood pressure (mmHg), as well as heart rate (bpm), were also measured using a digital sphygmomanometer (Tables 1 and 2). The measurement was performed on an arm rested at heart level, and the cuff was placed two fingers above the brachial artery. These measurements were performed at the Pediatric Clinical Research Unit from Arkansas Children's Hospital using a GE Carescape V100 Dinamap vital sign monitor following the standard procedures for this unit. The instrument is calibrated for children.

Fat-free mass, fat mass, and total body water were also measured using the Tanita Body Composition Analyzer (Model TBF-300A; Tanita Corporation of America, Inc., Arlington Heights, IL, USA).

### 2.2. Blood Collection and Processing

Fasting venous blood samples were collected in EDTA tubes to isolate peripheral blood mononuclear cells (PBMCs), as previously described [21]. Plasma samples were collected after whole blood centrifugation (1,500 × g for 30 min at 4°C). Thereafter, samples were stored up to 1-2 years at -80°C, until the study was concluded, so that all samples could be measured together to reduce batch effects. Then, the plasma volume was replaced with wash buffer consisting of Ca^2+^/Mg^2+^-free PBS supplemented with 2 mM EDTA and 0.1% BSA (Sigma Aldrich, St. Louis, MO). To perform the gradient separation, Histopaque-1077 (Sigma Aldrich) was used. The diluted blood was layered on histopaque and centrifuged at 400 × g for 30 min at room temperature. The white cloudy layer of PBMCs was collected and washed two times with ~20 ml of room temperature wash buffer. PBMCs were counted using a hemocytometer (Bright-Line; Hausser Scientific, Horsham, PA), and 2-5 million PBMCs were pelleted, snap frozen on dry ice, and stored up to two years at -80°C, until the study was concluded [21].

### 2.3. Biochemical Measures

Fasting insulin concentration was measured in plasma using the Mesoscale Discovery Platform (MSD Multi-Array Assay System, Gaithersburg, MD, USA) according to the manufacturer's protocol. Fasting plasma glucose was measured using YSI 2900 biochemistry analyzer (YSI Life Sciences, Yellow Springs, OH, USA). The lipid profile was quantified in plasma using a RX Daytona clinical analyzer accordingly to the manufacturer's instructions (Randox Laboratories-IS Limited, Kearneysville, WV, USA)—nonesterified fatty acids (NEFA: mmol/L), glycerol (*μ*mol/L), high-density lipoprotein (HDL: mmol/L), low-density lipoprotein (LDL: mmol/L), and triglycerides (TGs: mmol/L). Additionally, plasma lactate (mmol/L) and CRP were measured using the same methodology.

Fasting insulin (*μ*U/mL) and glucose concentrations (mmol/L) were used to calculate HOMA-IR and HOMA-*β* using the following equations:

(1) $$\begin{array}{c} \text{HOMA}-\text{IR}=\frac{\text{fGlucose }\left(\text{mmol}/\text{L}\right)\times \text{fInsulin }\left(\mu \text{U}/\text{mL}\right)}{22.5},\\ \text{HOMA}-\beta =\frac{\text{fInsulin }\left(\mu \text{U}/\text{mL}\right)\times 20}{\text{fGlucose }\left(\text{mmol}/\text{L}\right)-3.5}. \end{array}$$

HOMA-IR was used to determine the insulin sensitivity status for each participant. When HOMA − IR ≥ 2, the participant was considered insulin resistant [22–24].

### 2.4. Sample Preparation for Aminothiol Analysis

Plasma was prepared for analysis as previously described by Melnyk et al. [25] in order to determine free reduced and oxidized or total reduced aminothiols. Briefly, to assess the total concentration of aminothiols, 50 *μ*L of a solution containing 1.43 M of sodium borohydride, 66 mM sodium hydroxide, 1.5 *μ*M EDTA, and 10 *μ*L *n*-amyl alcohol was added to 200 *μ*L of plasma and incubated for 30 min at 40°C. Thereafter, the proteins were precipitated by incubation for 10 min with cold 10% meta-phosphoric acid, the samples were centrifuged for 15 min at 14,000 RPM, and 20 *μ*L of supernatant was measured by HPLC. To assess the free and oxidized aminothiol concentration, an equal volume of 10% meta-phosphoric acid was added to the plasma samples and treated as previously described [25].

### 2.5. Aminothiols and Oxidative Damage Marker Identification

Total and free aminothiols were separated using a Shimadzu HPLC with a Shimadzu pump model 580 on a 5 *μ*m, 4.6 × 150 mm i.d. reverse-phase C_18_ column (MCM, Inc., Tokyo, Japan) with the thermostat at 25°C. An isocratic mobile phase composed of 50 mM sodium phosphate, 1.0 mM of reagent OSA, and 2% acetonitrile (v/v) at pH 2.7 was used. The detection of all compounds was carried out using a Coulochem II EC detector, model 5200A (ESA, Inc.). The identification was carried out using external standards for each compound: methionine, homocysteine, cysteine, cystine, cysteinylglycine, reduced and oxidized glutathione, gamma-glutamylcysteine, 3-nitro-tyrosine, and 3-chloro-tyrosine, as previously described [25].

The percentage of oxidized GSH was obtained using the following equation [14]:

(2) $$\begin{array}{c} \%\text{oxidized GSH}=\frac{2\text{GSSG}}{\text{free GSH}+2\text{GSSG}}\times 100. \end{array}$$

### 2.6. Plasma Adipokines and Cytokine Quantification

A plasma adipokine and cytokine kit was used to measure leptin, IL-1*β*, IL-6, IL-8, MCP-1, and TNF-*α* by multiplexing using a Milliplex® Map Human Adipokine Panel (Millipore®, MA, USA). Adiponectin was also measured using a human Adiponectin ELISA (Millipore®, MA, USA). All procedures were performed according to the manufacturer's instructions.

### 2.7. DNA Methylation Profile

DNA methylation was assessed in the isolated PBMCs. The Puregene Blood Kit (Gentra Systems, Inc., Minneapolis, MN, USA) was used to extract the DNA, and it was further bisulfite-converted and purified using an EZ DNA Methylation-Gold kit (Zymo Research, Irvine, CA, USA) according to the manufacturer's protocol [26]. After bisulfite-conversion, the methylation was determined using the Infinium MethylationEPIC bead chip from Illumina®. The acquired data was followed by a quality control analysis of samples and probes, followed by further normalization using the Bioconductor packages *minfi* v1.34.0 and *watermelon* version 1.32.0 [27, 28] in R version 4.0.2 [29, 30]. In order to reduce the bias within-array, the data was normalized combining Noob+BMIQ (*β*-mixture quantile normalization) in order to improve signal intensities [27, 31]. After normalization, the data was filtered and probes that failed (*p* value >0.01) were removed. All probes mapped to the X and Y chromosomes were also removed to avoid sex chromosome bias. Finally, cross-reactive probes [32] and probes including known SNPs were also removed, according to Illumina recommendations, before final statistical analysis [33].

### 2.8. Statistical Analysis

The original statistical power for the present study was computed with a total of 110 children based on a one-factor ANOVA, 80% power and *α* = 0.05 were assumed, and a minimum detectable Cohen's *f* effect size of 0.33 was used. To test the differences between HL and HO and between HO and UHO, a *t*-test was performed when data fulfilled all the assumptions—the normal distribution was tested by the Shapiro-Wilk test, and the variance homogeneity was tested by the Levene's test. Otherwise, a Wilcoxon signed-rank test was performed. Results are presented as mean ± standard deviation (sd) and median (Q1–Q3) according to the respective test. The correlation between continuous variables was assessed using the Spearman's Rank-Order correlation, and the coefficient (rho) is shown for each correlation. A *p* value <0.05 was considered statistically significant. These tests were performed using the R version 4.0.2 [29, 30].

For statistical analysis of methylation results, differentially methylated positions (DMPs) were tested among groups using limma v3.44.3 [34] R package, after converting *β* values into *M* values. Covariates such as sex, age, and race were adjusted to the linear model. The *p* values were adjusted by the Benjamini-Hochberg method (false discovery rate [FDR]) [35]. DMPs were considered significative for FDR < 0.1. Gene set enrichment analyses were performed using the webtool STRING database v11.0b [36], for Gene Ontology (GO), KEGG (Kyoto Encyclopedia of Genes and Genomes), and Reactome pathways. Significant results were defined as FDR < 0.05.

## 3. Results

### 3.1. Physiologic and Biochemical Characterization of Study Population

The physiologic and biochemical characteristics of the study population were stratified according to the BMIz and HOMA-IR, as shown in Tables 1 and 2, respectively. The HO showed a significantly higher BMIz (*p* < 0.01) and waist circumference (*p* < 0.01), as compared with the HL subjects. In addition, the HO displayed a significantly higher fat-free mass (*p* < 0.01) and fat mass (*p* < 0.01) compared with HL. Interestingly, the HO showed significantly elevated plasma insulin levels (*p* < 0.01) when compared with the HL, despite normal fasting glucose levels and a HOMA − IR < 2. The HL presented a better *β*-cell insulin secretory function (HOMA-*β*) compared to the HO group (Table 1). Although obesity is normally characterized by dyslipidemia, this was not observed in the HO group, as their lipid profile was similar to that of HL. The systolic blood pressure was elevated in the HO as compared to HL subjects (*p* = 0.047).

Differences between the HO and UHO groups are presented in Table 2. The data indicate that UHO participants had higher BMIz as compared to the HO. Their higher BMIz was caused by a significant higher fat mass, as well as fat-free mass, that was accompanied by a significantly higher total body water in UHO compared to the HO. Insulin was significantly higher in the UHO group, in comparison to the HO group. This was also accompanied by a significant insulin secretory dysfunction, as represented by the HOMA-*β* index (*p* = 0.0145). Interestingly, these metabolic defects are already present in this prepubertal cohort of obese children, even in the presence of normal fasting plasma glycemia. The fasting glucose levels are similar among all the groups. While there were no differences in plasma cholesterol levels in the HL vs. HO, the UHO presented dyslipidemia which was characterized by a decrease in HDL cholesterol (*p* = 0.01) and significant increase in triglycerides levels (*p* = 0.041). The LDL cholesterol levels were slightly increased in the UHO compared to the HO participants, but the difference did not reach statistical significance (*p* = 0.34) in the UHO vs. HO participants. In spite of the young age of this cohort of UHO subjects, they already presented significantly elevated systolic blood pressure, as compared to the HO. Furthermore, the plasma lactate concentration was also significantly higher in the UHO compared to the HO. The lactate concentration was fairly well correlated with high levels of plasma insulin (Spearman's correlation, rho = 0.42, *p* value <0.01) and with HOMA-IR (Spearman's correlation, rho = 0.44, *p* value ≤0.01) (Supplementary Table 1B).

### 3.2. Transmethylation Metabolites

Transmethylation metabolites, such as methionine, SAH, SAM, adenosine, and homocysteine were quantified in the three groups of participants (Table 3). Of the evaluated metabolites, SAH levels were significantly increased in the HO group compared with the HL subjects. Interestingly, similar results were also observed for homocysteine levels. However, there was no difference in the SAM/SAH ratio. This ratio is frequently used to predict the methylation capacity of the cells. When UHO were compared with HO, no significant differences were found between groups.

### 3.3. Transsulfuration Metabolites

In parallel to the measurements of plasma transmethylation metabolites, Table 4 shows the levels of plasma metabolites related with the redox state as well as the transsulfuration pathway. When comparing the HL with the HO prepubertal children, several metabolites were significantly altered. The HO children exhibited increased levels of total cysteine (tCysteine; free circulating+protein-bound), while free cysteine (fCysteine) was not different as compared to the HL. Similarly, increased levels of cystine were observed. The cysteine oxidation ratio (fCysteine/cystine) is an important redox buffer responsible for maintaining the plasma redox state. However, the ratio was not different between groups. The homocysteine levels described above play an important role in the transsulfuration pathway, since it acts as an intermediary metabolite between both the transmethylation and the transsulfuration pathways. In fact, homocysteine is the main source of cysteine that is used to synthetize glutathione (GSH). Total reduced GSH (tGSH) and free reduced GSH (fGSH) were also measured but showed no significant differences between groups. On the other hand, the oxidized glutathione (GSSG) was significantly elevated in the HO compared with the HL subjects. The evaluation of tGSH/GSSG and fGSH/GSSG ratios showed a significant reduction in the antioxidant capacity and consequently an increase in oxidative stress in plasma of the HO children. The percentage of oxidized GSH was also higher in HO subjects compared to HL. Additionally, the levels of cysteinylglycine in HO group were also significantly higher, when compared with the HL.

Biomarkers of nitrosative stress, 3-chloro-tyrosine and 3-nitro-tyrosine, showed no significant differences between groups.

### 3.4. Inflammation Patterns

Oxidative stress is normally accompanied by an increase in the systemic inflammatory status. Therefore, we measured inflammatory cytokines as well as the inflammatory marker, C-reactive protein (CRP) in the plasma (Table 5). While CRP was elevated in HO as compared with the HL, no significant alterations were observed for inflammatory cytokines except for the unexpected finding of reduced TNF alpha levels in UHO as compared to HO subjects. Also, presented in Table 5 are the circulating levels of leptin and adiponectin, which are important cytokines secreted by adipose tissue. Adiponectin was reduced in the UHO as compared to the HO subjects while leptin levels and the leptin/adiponectin ratio were significantly elevated in the UHO as compared to the HO as well as in HO when compared to the HL groups. Increased levels of CRP were correlated with the leptin levels (Spearman's correlation, rho = 0.69, *p* < 0.01) (Supplementary Table 1A).

### 3.5. DNA Methylation Pattern

**Figure 1:**
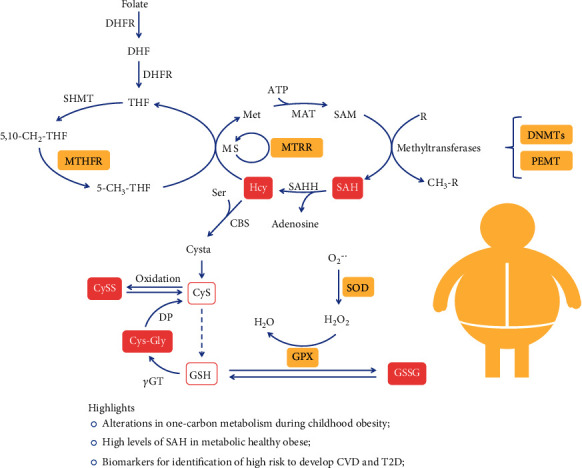
One-carbon metabolism perturbations during childhood obesity. Yellow squares represent the enzymes that have altered DNA methylation pattern; pink squares represent increased metabolites found associated to obesity; bold symbols represent enzymes; DHFR: dihydrofolate reductase; DHF: dihydrofolate; THF: tetrahydrofolate; Met: methionine; MAT: S-adenosylmethionine synthetase; SAM: S-adenosylmethionine; SAH: S-adenosylhomocysteine; SAHH: SAH hydrolase; Hcy: homocysteine; MS: methionine synthase; MTRR: methionine synthase reductase; Ser: serine; Cysta: cystationine; CyS: cysteine; CySS: cystine; GSH: glutathione; *γ*GT: *γ*-glutamyl transpeptidase; Cys-Gly: cysteinylglycine; DP: dipeptidase; GPX: glutathione peroxidase; GSSG: oxidized glutathione; SOD: superoxide dismutase.

Since the transmethylation and transsulfuration pathways have a complementary loop as shown in Figure 1, and to further support our described findings of metabolic perturbations with possible effect at the DNA methylation mechanism, we analyzed the DNA methylation profile in PBMCs from a subset of the participants (*N* = 14 HL, *N* = 16 HO, and *N* = 11 UHO). Since no significant differences were observed between the HO and the UHO groups, both groups were merged (OverallObese) to achieve higher statistical power. Therefore, the HL (*n* = 14) were compared with the OverallObese subjects (*n* = 27) adjusting the model for HOMA-IR, thus enabling us to better isolate the effect of obesity.

From the cytosine-phosphate-guanines (CpGs) analyzed, 4677 were differentially methylated between the two groups (FDR < 0.1) (Supplementary Table 2). Furthermore, 35% of the significant DMPs presented a reduction in the methylation status (hypomethylation) in the OverallObese group, while 65% of the DMPs were hypermethylated in the same group. Moreover, 24 DMPs were selected from the list of 4677 DMPs based on their association with genes that are directly or indirectly related with one-carbon metabolism and consequently associated with oxidative stress and/or methylation processes [37–39], as explained before (Table 6).

Some of these DMPs are associated with genes involved in the expression of important enzymes, such as methionine synthase reductase (*MTRR* gene), methylenetetrahydrofolate dehydrogenase (*MTHFD1* gene), methylenetrahydrofolate reductase (*MTHFR* gene), and glycine-N-methyltransferase (*GNMT* gene). These enzymes are key in one-carbon metabolism and are responsible for methionine regeneration through the homocysteine conversion and the transmethylation pathway [40]. From these important results, it is possible to predict a downregulation in these enzymes since their genes are hypermethylated (positive *β* value or fold change in Table 6). The alteration in the methylation status of these enzymes could indeed explain the observed increase in SAH and homocysteine plasma levels observed in the HO (Table 3). Moreover, the methylation results also showed alteration in the regulation of genes involved in oxidative stress, which include glutathione peroxidase 1 and 7 (*GPX1* and GPX7), oxidation resistance 1 (*OXR1*), and superoxide dismutase 1 (*SOD1*). All these genes were hypermethylated in the OverallObese group, suggesting epigenetic-mediated downregulation. Interestingly, the enzymes responsible for DNA methylation, i.e., DNA methyltransferases (transcribed by the gene *DNMT1*), seem to be downregulated in the OverallObese group as well.

**Figure 2:**
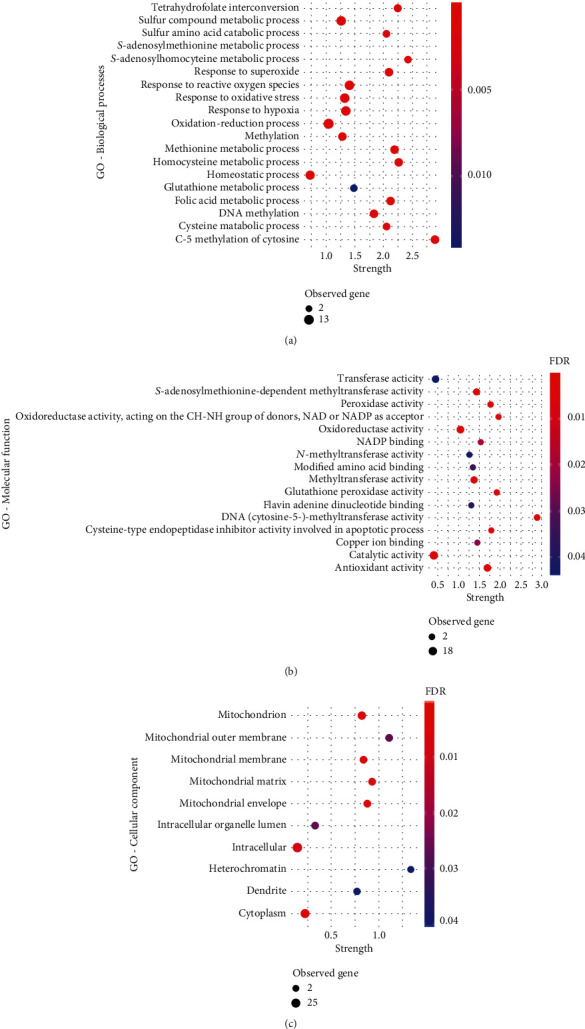
Gene Ontology (GO) term enrichment analysis for the genes related to the selected 24 DMPs. (a) GO terms for biological process terms; (b) GO terms for molecular function terms; (c) GO terms for cellular component terms; strength—enrichment effect, measured by the log_10_ of the number of proteins observed divided by the number of expected proteins. GO: Gene Ontology; FDR: false discovery rate; observed genes: number of genes (DMPs) involved in each GO term.

**Figure 3:**
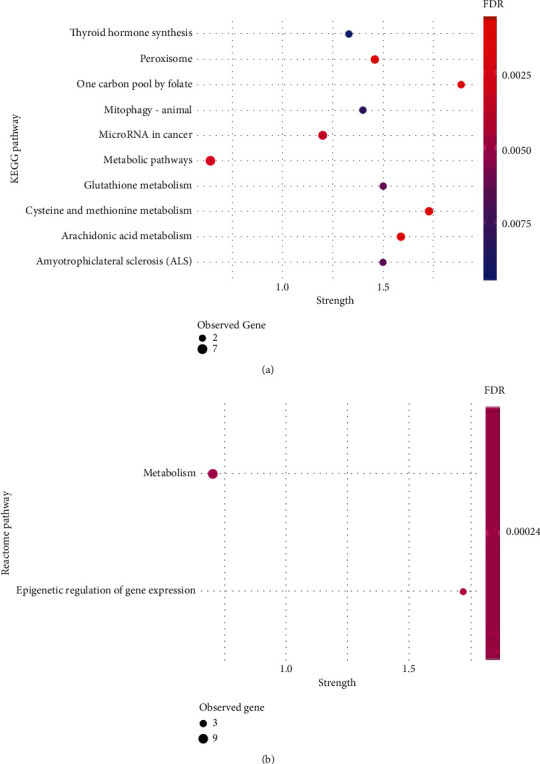
Pathway enrichment analysis for genes related to the selected 24 DMPs. (a) KEGG pathways; (b) Reactome pathways; strength—enrichment effect, measured by the log_10_ of the number of proteins observed divided by the number of expected proteins. GO: Gene Ontology; FDR: false discovery rate; observed genes: number of genes (DMPs) involved in a particular pathway.

In order to disclose the biological meaning of these results, gene enrichment analysis was performed for the 24 DMP set, using the STRING database [36]. Importantly, the enrichment analysis showed 207 significant biological processes (Supplementary Table 3). Furthermore, 19 of them are terms involved in aminothiol metabolism, such as “response to oxidative stress,” “methylation,” “S-adenosylhomocysteine metabolic process,” and “homocysteine metabolic process” (Figure 2(a)). In addition, 16 molecular functions, including SAM-dependent methyltransferase and peroxidase activity and 10 cellular components, including mitochondrial components, appear to be affected through the methylation pattern (Figures 2(b) and 2(c)). Interestingly, mitochondria seem to be of the most affected cellular component (Figure 2(c)). The KEGG pathway analysis, shown in Figure 3(a), indicates alterations in cysteine, methionine, and glutathione metabolism, as well as “one-carbon pool by folate.” The Reactome pathway enrichment corroborates previous findings by showing alterations in metabolism and their association to epigenetic regulation (Figure 3(b)).

## 4. Discussion

Overnutrition and poor-quality diets are triggering a severe increase in obesity worldwide, with resulting metabolic disorders starting early in life, in particular during childhood [41]. Our results show that obesity has already caused profound changes in several aspects of metabolism in a cohort of prepubertal children, particularly in the one-carbon metabolism, of particular importance in the folate, transmethylation, and transsulfuration pathways. These studies, evaluating early childhood obesity, can inform on the potential origin of the related comorbidities that start plaguing many, already in young adulthood. We first chose to stratify children with obesity into two groups, the healthy and unhealthy obese, in order to understand the main differences between both conditions, where the unhealthy obese already presented insulin resistance. Our main criteria for this stratification were based on the HOMA − IR ≥ 2, although different phenotypes could be used to characterize and differentiate the metabolic state of this population, as reviewed by Phillips [41], who described various valid ways to characterize and distinguish between metabolically healthy and metabolically unhealthy obese children [41]. This separation between obese groups may give some important insights into adulthood comorbidities linked to obesity [41], and our analyses have revealed essential differences in several metabolic processes already emerging in this pediatric cohort. All pediatric subjects in this study presented normoglycemia, while the state of insulin resistance driven by the high circulating insulin concentrations observed in the UHO group likely reflects an attempt to maintain their euglycemia [42]. This is in agreement with other studies indicating that the insulin-resistant state could be present years before any alteration in circulating glucose are detected [43, 44]. Chronic periods of insulin resistance, even in the absence of elevated fasting glucose, may be an important contributing factor to the early blood pressure alterations also observed in our cohort [45, 46]. The increase in insulin production is reflected by the increased HOMA-*β* observed in the obese unhealthy subjects, reflecting increased *β*-cell activity. Similar to insulin, lactate levels were also elevated in the insulin-resistant obese group. This phenomenon has already been observed by Hosking et al. [47] in children with insulin resistance, indicating a positive correlation between insulin resistance and lactate levels during childhood and adolescence, even after controlling for BMIz as covariate BMIz. Furthermore, Berhane et al. [48] have shown that plasma lactate was increased in adults during a hyperinsulinemic euglycemic clamp, a method that mimics the hyperinsulinemic state. Besides, they also indicated that high levels of lactate were present even before the insulin resistance was clinically detected [48]. Our data show that children as young as 5 to 10 years of age already show significantly elevated levels of lactate in the circulation, when comparing the HO to the UHO subjects. Moreover, there was a positive correlation between insulin and lactate levels as previously demonstrated [47]. Obesity-related insulin resistance has been involved in the development of other metabolic conditions, including CVD [49, 50] and lipid dysregulation [51, 52]. The insulin-resistant UHO subjects also presented lipid dysregulation and increased systolic blood pressure, risk factors for CVD. Furthermore, chronic low-grade inflammation and increased oxidative stress are characteristics of obesity [15]. The quantification of thiol molecules during prepubertal obesity and insulin resistance has not been well described. Some of these molecules are intermediaries of one-carbon metabolism and are important for the maintenance of redox homeostasis and the methylation capacity of cells by acting on transsulfuration and transmethylation pathways. Our data clearly show important differences in the transmethylation pathway, especially when comparing the HL and HO subjects. The HO showed higher levels of SAH and total homocysteine, similarity to the previously reported findings by Kumar et al. [53]. These molecules have already been implicated in the development of atherosclerosis and CVD [20, 54]. Similar alterations have been identified in diabetic patients with renal dysfunction, and the homocysteine levels were also positively correlated with insulin levels [40]. In addition, Chiang et al. [40] have observed the effect of insulin (1 *μ*M) in HepG2 cell lines and showed that mRNA expression of different enzymes related with one-carbon metabolism, such as MTRR, was reduced during the treatment with insulin. In agreement, the HO subjects in the present study had higher fasting insulin levels that are accompanied with high levels of homocysteine in plasma, compared to their lean counterparts. Importantly, DNA methylation analysis, from PBMCs isolated from the prepubertal children with obesity, showed hypermethylation of different CpGs that are localized in the vicinity of important metabolic genes, including *PEMT*, *MTRR*, and *MTHFR*, suggesting that an epigenetic effect could be the cause for this downregulation. Gene Ontology enrichment analysis also showed alteration in different key biological processes, molecular functions, and cellular components related with one-carbon metabolism, as shown in Figure 2. Even though the SAM/SAH ratio was not altered in our cohort, there were already alterations in the methylation profile of PBMCs in the obese subjects. Yi et al. [55] correlated high levels of homocysteine and SAH with a decrease in DNA methylation of lymphocytes. High SAH levels are also described as inhibitors of methyltransferase processes [20]. Furthermore, alterations in the transsulfuration pathway were also identified, in particular when comparing the HL with the HO subjects. This pathway is important in the redox state maintenance, since it is responsible for the synthesis of molecules, such as cysteine and GSH, that act as important ROS scavengers [14]. Interestingly, increased insulin levels have been shown to disrupt the redox homeostasis and consequently alter the defense mechanisms against excess ROS production [56]. Elshorbagy et al. [57] indicated that alterations in plasma total cysteine are associated with an increase in BMI, in adults. Similarly, we also observed higher levels of total cysteine in the HO subjects compared to their lean counterparts. On the other hand, the levels of fCysteine remain unchanged between groups even in the presence of higher levels of cystine, especially in the obese groups. Furthermore, the fCysteine/cystine ratio is an important plasma marker that defines an imbalance in redox homeostasis [58]. However, no differences were identified in that ratio between groups in our cohort, although the levels of GSSG were increased in the obese groups. Similar results were postulated by Choromańska et al. [59] who reported high levels of GSSG in plasma of obese adults with hypertension. While the fCysteine/cystine ratio was not altered, we observed significant alterations in the GSH/GSSG ratio in the HO group, with an increase in the percentage of GSH oxidation compared to HL. Few studies of this kind have been performed in prepubertal children with obesity; however, it is known that in adults, insulin resistance and dyslipidemia have a significant impact on oxidative stress [3, 60]. In line with the alterations detected in the redox homeostasis, increased plasma inflammation was also observed, in the children with obesity, in particular CRP and leptin levels. Pedersen et al. [61] demonstrated that hyperinsulinemia during obesity induced the expression of inflammation-associated genes. Importantly, the role leptin plays in inflammatory exacerbation has been reviewed [2]. Interestingly, high levels of homocysteine have also previously been implicated with inflammation [62].

These metabolic alterations are key factors for future development of obesity-related comorbidities, particularly in people with insulin resistance, and have been described, particularly in adults, as important predictive markers of CVD, including atherosclerosis [20, 54, 63]. Additionally, our data show significant differences in adipocyte secretion of adipokines. While leptin levels were increased in both obese groups, adiponectin levels were decreased in the UHO group. Our results are in agreement with other studies indicating low adiponectin levels, as a marker of adipocyte secretory dysfunction, together with elevated leptin secretion [64, 65]. Our data indicate an evident alteration in adipocyte secretory patterns, especially in the obese- and insulin-resistant subjects. Landgraf et al. [66] corroborate the presence of adipose tissue dysfunction during childhood obesity, making the correlation between adipocyte expansion and inflammation. Interestingly, the high leptin and low adiponectin levels together with the increased plasma lactate are early important markers that differentiate obese subjects with insulin resistance from their metabolic healthy counterpart. Our results show a deregulated adipocyte secretory function, even under normoglycemia in prepubertal children with obesity.

Moreover, our data also show significant changes in the methylation patterns of PBMCs. Circulating cells have been considered important surrogate markers for different type of diseases, since they are in contact with the continuous changing circulating molecules from surrounding tissues. Therefore, small metabolic changes that may occur in the body will induce key modifications in the biology of these cells [67]. In agreement, our results reflect the impact of obesity on the epigenetic patterns of PBMC DNA. In particular, we noticed alterations of the methylation degree in the vicinity of genes that are involved in one-carbon metabolism, such as the transmethylation and transsulfuration pathways. Previous studies have shown alterations in methylation patterns associated with obesity and obesity-related insulin resistance [68–70]. The lack of significant differences in these metabolic pathways and in the DNA methylation results when comparing the HO and the UHO subjects might likely be due to the low study power when we split the obese subjects into two different groups. Our study is limited by relatively small sample sizes as we were unable to fully reach our recruitment goals. Unfortunately, we do not have the DNA methylation data for the entire cohort. In future studies, we would like to increase the *N* in each group and invite the subjects back two years later for a follow-up visit.

Finally, the physiological and biochemical characteristics of the young prepubertal children show that among the obese subjects, there are indeed a group of HO that are still metabolically healthy as the HL, while the UHO already presented metabolic dysfunction, even under normoglycemia. This suggests that the adult obesity phenotype is starting to set in at an early age, raising important questions about the reversibility of this condition and the future health of these subjects.

In conclusion, our study showed the presence of deep alteration in one-carbon metabolism and related pathways in children with obesity (Figure 1), as young as 5-9 years of age. These alterations are mainly driven by obesity and elevated insulin levels. Most importantly, even the metabolic healthy obese subjects show increased levels of important metabolic markers, such as SAH, that is related with developing future comorbidities. This may reflect an important transition phase between different stages of obesity and insulin resistance. Specific markers that could identify this transition would be extremely important in identifying populations early at high risk of developing CVD and T2D. The data presented provide insight on important metabolic changes that occur in obesity early in life (summarized in Figure 1). Importantly, the metabolic healthy obese children seem to have some compensatory mechanisms to maintain important features unchanged, including the lipid profile and glucose levels. Also, some important alterations are already established at the epigenetic level, although some of these alterations could still be reversed by possible changing lifestyle habits [39].

## Figures and Tables

**Table 1:** Physiologic and biochemical characteristics of healthy prepubertal children stratified per BMIz.

Characteristics	*n*	HL	*n*	HO	*p* value
Sex: male/female	20	14/6	28	15/13	
Age (years)	20	7.0 (6.0–8.0)	28	7.0 (6.0–8.3)	ns
BMIz	20	0.078 ± 0.663	28	1.846 ± 0.557	<0.01
WC (cm)	20	55.0 (51.4-56.6)	28	61.5 (56.8-75.1)	<0.01
Systolic BP (mmHg)	20	99.7 ± 9.71	28	106.71 ± 10.39	0.047
Diastolic BP (mmHg)	20	58.7 ± 9.30	28	62.29 ± 7.65	ns
Heart rate (bpm)	20	78 ± 12	28	79 ± 11	ns
Fat mass (kg)	20	3.9 (3.3-5.2)	28	9.4 (6.5-14.4)	<0.01
Free-fat mass (kg)	20	19.0 (18.1-21.7)	28	23.3 (20.3-26.0)	<0.01
Total body water (kg)	20	13.9 (13.3-15.9)	28	17.1 (14.8-19.0)	<0.01
Insulin (*μ*U/mL)	20	3.65 ± 1.55	28	5	<0.01
Glucose (mmol/L)	20	4.91 (4.75-5.12)	27	5.03 (4.60-5.35)	ns
HOMA-IR	20	0.79 (0.54-0.92)	27	1.21 (1.00-1.49)	<0.01
HOMA-*β*	20	50.17 (30.65-66.85)	27	72.05 (44.40-128.60)	0.015
HDL cholesterol (mmol/L)	20	1.47 ± 0.31	27	1.40 ± 0.29	ns
LDL cholesterol (mmol/L)	20	2.16 ± 0.61	27	2.35 ± 0.76	ns
Triglycerides (mmol/L)	20	0.52 (0.41-0.65)	27	0.53 (0.37-0.82)	ns
Total cholesterol (mmol/L)	20	3.73 ± 0.68	27	3.89 ± 0.81	ns
NEFA (mmol/L)	20	0.10 (0.06-0.16)	27	0.08 (0.04-0.13)	ns
Glycerol (*μ*mol/L)	20	77.27 (64.19-130.24)	27	82.77 (68.20-92.10)	ns
Lactate (mmol/L)	20	2.07 ± 0.57	27	2.03 ± 0.47	ns

HL: healthy lean; HO: healthy obese; BMIz: BMI z-score; WC: waist circumference; BP: blood pressure; BMR: basal metabolic rate; HOMA-IR: homeostatic model assessment of insulin resistance; HOMA-*β*: homeostatic model assessment of *β*-cell function; LDL: low-density lipoprotein; HDL: high-density lipoprotein; NEFA: nonesterified fatty acids; ns: nonsignificant; *p* value <0.05 was considered significant.

**Table 2:** Physiologic and biochemical characteristics of prepubertal children with obesity stratified per HOMA-IR.

Characteristics	*n*	HO	*n*	UHO	*p* value
Sex: male/female	28	15/13	16	7/9	
Age: years	28	7.0 (6.0–8.3)	16	8.0 (7.0–9.0)	ns
BMIz	28	1.85 ± 0.56	16	2.45 ± 0.55	<0.01
WC (cm)	28	65.6 ± 12.1	16	77.5 ± 12.3	ns
Systolic BP (mmHg)	28	106.7 ± 10.4	16	113.5 ± 9.1	0.035
Diastolic BP (mmHg)	28	62.3 ± 7.7	16	64.75 ± 8.4	ns
Heart rate (bpm)	28	79 ± 11	16	83 ± 10	ns
Fat mass (kg)	28	9.4 (6.5-14.4)	16	19.3 (16.8-23.9)	<0.01
Fat-free mass (kg)	28	23.3 (20.3–26.0)	16	29.2 (26.4-31.8)	<0.01
Total body water (kg)	28	17.0 (14.8-19.0)	16	21.4 (19.3-23.3)	<0.01
Insulin (*μ*U/mL)	28	5.75 (4.56-7.10)	16	15.19 (10.33-25.20)	<0.01
Glucose (mmol/L)	27	4.94 ± 0.52	16	5.28 ± 0.79	ns
HOMA-IR	27	1.21 (1.00-1.49)	16	3.00 (2.24-6.24)	<0.01
HOMA-*β*	27	72.05 (44.40-128.60)	16	200.74 (143.46-300.65)	<0.01
HDL cholesterol (mmol/L)	27	1.33 (1.18-1.59)	14	1.19 (1.05-1.24)	0.010
LDL cholesterol (mmol/L)	27	2.27 (2.01-2.97)	14	3.03 (2.16-3.25)	ns
Triglycerides (mmol/L)	27	0.53 (0.37-0.82)	14	0.75 (0.65-1.20)	0.041
Total cholesterol (mmol/L)	27	3.81 (3.47-4.51)	14	4.37 (3.47-4.58)	ns
NEFA (mmol/L)	27	0.08 (0.03-0.13)	16	0.09 (0.07-0.11)	ns
Glycerol (*μ*mol/L)	27	82.77 (68.20-92.10)	14	91.12 (73.80-111.00)	ns
Lactate (mmol/L)	27	2.03 ± 0.47	14	2.62 ± 0.60	<0.01

HO: healthy obese; UHO: unhealthy obese; BMIz: BMI z-score; WC: waist circumference; BP: blood pressure; BMR: basal metabolic rate; HOMA-IR: homeostatic model assessment of insulin resistance; HOMA-*β*: homeostatic model assessment of *β*-cell function; LDL: low-density lipoprotein; HDL: high-density lipoprotein; NEFA: nonesterified fatty acids; ns: nonsignificant; *p* value <0.05 was considered significant.

**Table 3:** Plasma transmethylation metabolite concentrations in prepubertal children stratified per BMIz and HOMA-IR.

Metabolites	HL (*n* = 20)	HO (*n* = 28)	*p* value^∗^	UHO (*n* = 16)	*p* value^∗∗^
Methionine (*μ*mol/L)	19.22 ± 3.14	20.70 ± 3.53	ns	19.81 ± 3.44	ns
SAH (nmol/L)	19.32 (17.13-20.94)	23.34 (18.42-25.31)	0.027	21.76 (20.41-26.56)	ns
SAM (nmol/L)	45.24 ± 7.69	49.65 ± 8.00	ns	49.83 ± 9.75	ns
SAM/SAH ratio	2.35 ± 0.47	2.29 ± 0.53	ns	2.16 ± 0.53	ns
Adenosine (*μ*mol/L)	0.16 ± 0.06	0.19 ± 0.06	ns	0.19 ± 0.06	ns
Total homocysteine (*μ*mol/L)	5.06 (4.68-5.62)	6.23 (5.33-7.18)	<0.01	6.36 (5.46-6.86)	ns

HL: healthy lean; HO: healthy obese; UHO: unhealthy obese; SAM: S-adenosylmethyonine; SAH: S-adenosylhomocysteine; ^∗^LH-HO comparison; ^∗∗^HO-UHO comparison; ns: nonsignificant; *p* value <0.05 was considered significant.

**Table 4:** Circulating transsulfuration and oxidative damage metabolites in prepubertal children stratified per BMIz and HOMA-IR.

Metabolites	HL (*n* = 20)	HO (*n* = 28)	*p* value^∗^	UHO (*n* = 16)	*p* value^∗∗^
Total cysteine (*μ*mol/L)	183.80 (165.43-195.58)	199.75 (178.20-214.20)	0.020	195.60 (184.80-207.13)	ns
Free cysteine (nmol/L)	19.23 ± 2.50	20.75 ± 3.09	ns	20.86 ± 2.73	ns
Cystine (nmol/L)	18.45 (17.35-19.50)	20.30 (19.28-21.83)	0.005	20.15 (17.55-21.43)	ns
Free cysteine/cystine	1.04 ± 0.11	1.04 ± 0.10	ns	1.05 ± 0.13	ns
Total 𝝲-glutamylcysteine (*μ*mol/L)	1.65 ± 0.22	1.67 ± 0.25	ns	1.71 ± 0.17	ns
Total reduced GSH (*μ*mol/L)	5.63 ± 1.04	5.70 ± 1.08	ns	5.60 ± 0.71	ns
Free reduced GSH (*μ*mol/L)	1.75 (1.57-1.92)	1.77 (1.60-1.87)	ns	1.79 (1.61-1.84)	ns
GSSG (*μ*mol/L)	0.17 (0.15-0.20)	0.21 (0.17-0.26)	0.014	0.19 (0.17-0.23)	ns
Total reduced GSH/GSSG	32.81 ± 11.12	26.72 ± 8.66	0.038	30.15 ± 10.00	ns
Free reduced GSH/GSSG	10.00 ± 2.73	8.28 ± 2.43	0.026	9.36 ± 2.68	ns
Cysteinylglycine (*μ*mol/L)	31.73 ± 6.52	38.67 ± 7.99	0.003	38.68 ± 6.69	ns
Oxidized GSH (%)	16.17 (14.56–18.93)	19.05 (17.07–21.42)	0.021	17.40 (15.66–21.14)	ns
3-Chloro-tyrosine (nmol/L)	42.35 ± 7.34	46.44 ± 10.19	ns	44.88 ± 9.10	ns
3-Nitro-tyrosine (nmol/L)	30.38 ± 8.63	33.24 ± 7.77	ns	33.46 ± 5.50	ns

HL: healthy lean; HO: healthy obese; UHO: unhealthy obese; GSH: reduced glutathione; GSSG: glutathione dissulfide; ^∗^HL-HO comparison; ^∗∗^HO-UHO comparison; ns: nonsignificant; *p* value <0.05 was considered significant.

**Table 5:** Markers of inflammation and adipocyte function in prepubertal children stratified per BMIz and HOMA-IR.

	HL		HO		*p* value^∗^	UHO		*p* value^∗∗^
	*n*		*n*			*n*		
CRP (mg/L)	20	0.15 (0.15-0.19)	27	0.80 (0.35-2.28)	<0.01	14	1.16 (0.41-2.87)	ns
IL-6 (pg/mL)	19	13.73 (5.32-49.58)	28	8.31 (3.45-37.77)	ns	16	8.95 (2.13-13.08)	ns
IL-8 (pg/mL)	18	4.88 (4.09-13.75)	22	5.55 (4.22-10.40)	ns	9	3.39 (3.08-7.60)	ns
MCP1 (pg/mL)	19	127.78 ± 36.691	28	125.614 ± 41.909	ns	16	115.94 ± 39.06	ns
TNF alpha (pg/mL)	19	6.49 (5.43-7.91)	28	6.12 (4.32-7.67)	ns	16	3.86 (2.40-5.18)	0.015
IL-1beta (pg/mL)	16	0.90 (0.54-1.40)	23	0.76 (0.57-2.19)	ns	12	0.57 (0.57-0.80)	ns
Leptin (ng/mL)	19	88.77 (51.30-104.25)	28	662.30 (187.46-1620.67)	<0.01	16	1439.34 (951.73-1647.30)	0.036
Adiponectin (ng/mL)	20	13928.27 (11255.19-17488.51)	27	13917.50 (10686.40-18460.15)	ns	16	7800.46 (7091.7-9684.40)	<0.01
Leptin/adiponectin	19	0.006 (0.003-0.008)	27	0.057 (0.012-0.114)	<0.01	16	0.165 (0.121-0.249)	<0.01

HL: healthy lean; HO: healthy obese; UHO: unhealthy obese; CRP: C-reactive protein; IL: interleukin; MCP-1: monocyte chemoattractant protein 1; ^∗^HL-HO comparison; ^∗∗^HO-UHO comparison; ns: nonsignificant; *p* value <0.05 was considered significant.

**Table 6:** Differentially methylated positions in healthy lean (HL) and OverallObese prepubertal children that are related with genes involved in one-carbon metabolism.

DMP	Chr	Position	Genes	Δ*β*	Log_2_ fold change	*p* value^∗^
cg14819132	17	17495032	PEMT	0.011	0.553	0.050
cg00214165	5	7869652	MTRR; FASTKD3	0.026	0.617	0.056
cg02956320	2	169643050	NOSTRIN	-0.073	-0.137	0.056
cg05065230	3	49395807	GPX1	0.012	0.651	0.063
cg06293195	22	36878654	TXN2	-0.078	-0.146	0.064
cg19948014	21	33032656	SOD1	0.007	0.552	0.070
cg22473973	10	133794911	BNIP3	0.012	0.437	0.073
cg19014302	19	18303893	MPV17L2	0.017	1.148	0.076
cg07941301	6	42928277	GNMT	0.015	0.740	0.078
cg01495361	20	31369590	DNMT3B	-0.055	-0.089	0.079
cg27619163	17	7982806	ALOX12B	0.044	1.474	0.083
cg05065765	3	38206519	OXSR1	0.016	0.632	0.084
cg04550070	11	73694480	UCP2	0.011	0.565	0.085
cg26978822	16	56622779	MT3	-0.019	-0.029	0.084
cg03452047	1	53067911	GPX7	0.012	0.391	0.087
cg26748435	14	64854866	MTHFD1	0.022	0.600	0.088
cg04372675	8	107283146	OXR1	0.019	0.510	0.094
cg06858294	17	7983203	ALOX12B	0.030	0.834	0.093
cg19642128	8	26240703	BNIP3L	0.031	1.068	0.094
cg13722539	11	64085131	PRDX5; TRMT112	0.006	0.406	0.094
cg10216074	2	25467197	DNMT3A	0.015	0.025	0.097
cg22545535	17	17495014	PEMT	0.024	0.941	0.096
cg08869383	1	11865661	MTHFR; CLCN6	0.010	0.464	0.098
cg09692733	19	10249298	DNMT1	0.006	0.009	0.096

DMP: differentially methylated position; Chr: chromosome; ^∗^adjusted *p* value using false discovery rate (FDR).

## Data Availability

Additional data can be found in supplementary tables.

## Supplementary Materials

Supplementary 1 Supplementary Table 1: correlation of clinical and biochemical parameters (A) in healthy lean and healthy obese participants and (B) in healthy obese and unhealthy obese participants.

Supplementary 2 Supplementary Table 2: complete list of differentially methylated positions (DMPs).

Supplementary 3 Supplementary Table 3: GO term enrichment results for biological processes.
