# Supplementary material for: Redox Imbalance and Methylation Disturbances in Early Childhood Obesity
Source: Oxid Med Cell Longev. 2021 Aug 17;2021:2207125. doi: 10.1155/2021/2207125 (PMC8387800; doi:10.1155/2021/2207125)
Supplement: Supplementary 1 — Supplementary Table 1: correlation of clinical and biochemical parameters (A) in healthy lean and healthy obese participants and (B) in healthy obese and unhealthy obese participants. [file 2207125.f1.pdf]

Supplementary Table 1

A - Correlation of clinical and biochemical parameters in Healthy Lean and Healthy Obese participants

	BMIz	WC	SBP	DBP	HR	Insulin	Glucose	HOMA-IR	HOMA-B	LDL	HDL	Triglycerides	Total cholesterol	NEFA	Glycerol	Lactate	CRP	Leptin	Adiponectin
BMIz	1	0.706***	0.425**	0.246	0.019	0.616***	-0.052	0.593***	0.467***	0.197	-0.244	0.186	0.148	-0.024	0.098	0.071	0.727***	0.797***	-0.189
WC	0.706***	1	0.249	0.120	-0.084	0.544***	0.001	0.543***	0.328*	0.091	-0.435**	0.308*	0.003	0.006	0.143	-0.023	0.597***	0.665***	-0.354*
SBP	0.425**	0.249	1	0.575***	0.176	0.450**	-0.139	0.390**	0.444**	0.288*	0.102	0.154	0.365*	-0.031	0.051	0.038	0.220	0.341*	-0.079
DBP	0.246	0.12	0.575***	1	0.09	0.277	-0.133	0.264	0.307*	0.0336	0.059	0.127	0.115	0.079	0.260	0.030	0.287	0.093	-0.117
HR	0.019	-0.084	0.176	0.09	1	-0.052	-0.025	-0.059	0.009	0.214	-0.018	-0.023	0.17	0.030	-0.009	-0.029	-0.110	0.005	0.104
Insulin	0.616***	0.544***	0.450**	0.277	-0.052	1	-0.190	0.969***	0.866***	0.067	-0.125	0.339*	0.099	-0.219	-0.046	0.052	0.538***	0.703***	- 0.370**
Glucose	-0.052	0.001	-0.139	-0.133	-0.025	-0.19	1	0.0252	(-)0.595***	-0.078	0.056	0.027	-0.071	-0.147	-0.089	0.220	-0.165	-0.106	0.181
HOMA-IR	0.593***	0.543***	0.390**	0.264	-0.059	0.969***	0.0252	1	0.738***	0.069	-0.139	0.332*	0.088	-0.223	-0.053	0.069	0.506***	0.665***	-0.353*
HOMA-B	0.467***	0.328*	0.444**	0.307*	0.009	0.866***	-0.595***	0.738***	1	0.085	-0.063	0.204	0.123	-0.160	-0.041	-0.042	0.467***	0.557***	-0.302*
LDL	0.197	0.091	0.288*	0.0336	0.214	0.067	-0.078	0.069	0.085	1	-0.169	0.344*	0.905***	0.174	0.217	-0.041	0.153	0.251	0.064
HDL	-0.244	-0.435**	0.102	0.059	-0.018	-0.125	0.056	-0.139	-0.063	-0.169	1	-0.149	0.202	0.242	0.202	0.106	-0.442**	-0.189	0.342*
Triglycerides	0.186	0.308*	0.154	0.127	-0.023	0.339*	0.027	0.332*	0.204	0.344*	-0.149	1	0.354*	0.015	0.333*	0.304*	0.235	0.264	-0.234
Total cholesterol	0.148	0.003	0.365*	0.115	0.170	0.099	-0.071	0.088	0.123	0.905***	0.202	0.354*	1	0.239	0.301*	0.066	0.025	0.249	0.134
NEFA	-0.024	0.006	-0.031	0.079	0.03	-0.219	-0.147	-0.223	-0.16	0.174	0.242	0.015	0.239	1	0.705***	0.027	-0.047	-0.137	0.122
Glycerol	0.098	0.143	0.051	0.260	-0.009	-0.046	-0.089	-0.053	-0.041	0.217	0.202	0.333*	0.301*	0.705***	1	0.342*	0.125	0.069	-0.068
Lactate	0.071	-0.023	0.038	0.03	-0.029	0.052	0.220	0.069	-0.042	-0.041	0.106	0.304*	0.066	0.027	0.342*	1	-0.010	0.003	-0.069
CRP	0.727***	0.597***	0.22	0.287	-0.110	0.538***	-0.165	0.506***	0.467***	0.153	-0.442**	0.235	0.025	-0.047	0.125	-0.01	1	0.695***	-0.284
Leptin	0.797***	0.665***	0.341*	0.093	0.005	0.703***	-0.106	0.665***	0.557***	0.251	-0.189	0.264	0.249	-0.137	0.069	0.003	0.695***	1	-0.215
Adiponectin	-0.189	-0.354*	-0.079	-0.117	0.104	-0.370**	0.181	-0.353*	-0.302*	0.064	0.342*	-0.234	0.134	0.122	-0.068	-0.069	-0.284	-0.215	1
Methionine	0.161	0.083	0.212	0.266	-0.053	0.313*	0.0446	0.332*	0.235	0.083	0.08	0.269	0.158	-0.151	0.029	0.144	0.146	0.222	-0.150
SAM	0.199	0.049	0.289*	0.239	-0.04	0.314*	0.210	0.348*	0.141	0.163	0.08	0.236	0.195	-0.192	0.016	0.244	0.180	0.179	0.030
SAH	0.248	0.039	0.115	-0.032	-0.330*	0.198	0.117	0.185	0.091	-0.092	0.03	-0.072	-0.078	-0.381**	-0.207	0.063	0.217	0.263	-0.071
Adenosine	0.202	0.055	-0.140	-0.108	0.036	0.152	0.0857	0.148	0.056	-0.079	-0.126	-0.185	-0.132	-0.334*	-0.135	0.234	0.185	0.233	-0.163
Total homocysteine	0.367*	0.146	-0.051	-0.271	0.042	0.244	0.187	0.223	0.107	-0.012	-0.181	-0.079	-0.093	-0.529***	-0.369*	0.029	0.268	0.311?*	-0.154
Total Cysteine	0.279	0.217	0.193	0.008	0.036	0.238	0.142	0.238	0.055	0.175	-0.164	0.055	0.086	-0.271	-0.173	-0.079	0.139	0.321*	0.029
Free Cysteine	0.276	0.289*	0.172	-0.029	0.082	0.186	0.089	0.203	0.064	0.375**	-0.064	0.114	0.338*	0.147	0.169	-0.069	0.244	0.372**	0.104
Cystine	0.303*	0.208	0.073	-0.142	0.103	0.194	0.176	0.212	0.0314	0.230	-0.012	0.078	0.224	-0.020	0.026	0.052	0.256	0.359*	-0.061
Total Reduced GSH	0.038	0.177	0.176	0.081	-0.079	0.208	0.067	0.183	0.130	-0.079	-0.128	0.130	-0.092	-0.195	-0.089	0.033	0.011	-0.055	-0.058
Free Reduced GSH	-0.006	0.079	0.234	0.079	-0.091	0.075	0.184	0.063	-0.017	-0.173	0.002	-0.101	-0.177	-0.269	-0.199	0.010	-0.002	-0.074	0.097
GSSG	0.234	0.031	0.104	-0.004	0.094	0.012	0.122	-0.027	-0.035	-0.064	0.157	-0.102	0.008	-0.138	0.0048	0.213	0.078	0.259	0.230
Cystinyl glycine	0.357*	0.141	0.128	-0.070	0.036	0.197	0.332*	0.231	0.008	-0.088	-0.057	-0.016	-0.094	-0.176	-0.155	0.203	0.278	0.193	-0.081
Total γ-Glutamyl-Cysteine	-0.0001	0.111	0.07	0.182	-0.102	0.028	-0.026	0.042	0.070	0.013	-0.205	-0.107	-0.089	-0.175	-0.187	-0.254	0.087	-0.114	0.025
Oxidized GSH	0.233	-0.019	-0.035	-0.096	0.134	-0.046	0.072	-0.083	-0.075	0.035	0.127	-0.033	0.081	-0.018	0.076	0.243	0.085	0.243	0.197
3-Chloro-tyrosine	0.227	0.19	-0.121	-0.255	-0.076	0.096	0.154	0.074	-0.042	-0.047	-0.314*	0.054	-0.143	-0.142	-0.024	0.161	0.237	0.310*	-0.273
3-Nitro-tyrosine	0.100	-0.171	0.001	-0.063	-0.178	-0.021	0.108	0.003	0.026	-0.178	0.134	-0.033	-0.09	-0.193	-0.084	0.264	0.186	0.159	-0.083

	Methionine	SAM	SAH	Adenosine	Total homocysteine	Total Cysteine	Free Cysteine	Cystine	Total Reduced GSH	Free Reduced GSH	GSSG	Cystinyl glycine	Total γ-Glutamyl-Cysteine	Oxidized GSH	3-Chloro-tyrosine	3-Nitro-tyrosine
BMIz	0.161	0.199	0.248	0.202	0.367*	0.279	0.276	0.303*	0.038	-0.006	0.234	0.357*	-0.0001	0.233	0.227	0.100
WC	0.083	0.049	0.039	0.055	0.146	0.217	0.289*	0.208	0.177	0.079	0.031	0.141	0.111	-0.019	0.19	-0.171
SBP	0.212	0.289*	0.115	-0.14	-0.051	0.193	0.172	0.073	0.176	0.234	0.104	0.128	0.07	-0.035	-0.121	0.001
DBP	0.266	0.239	-0.032	-0.108	-0.271	0.008	-0.029	-0.142	0.081	0.079	-0.004	-0.07	0.182	-0.096	-0.255	-0.063
HR	-0.053	-0.04	-0.330*	0.036	0.042	0.036	0.082	0.103	-0.079	-0.091	0.094	0.036	-0.102	0.134	-0.076	-0.178
Insulin	0.313*	0.314*	0.198	0.152	0.244	0.238	0.186	0.194	0.208	0.075	0.012	0.197	0.028	-0.046	0.096	-0.021
Glucose	0.0446	0.21	0.117	0.085	0.187	0.142	0.089	0.176	0.067	0.184	0.122	0.332*	-0.026	0.072	0.154	0.108
HOMA-IR	0.332*	0.348*	0.185	0.148	0.223	0.238	0.203	0.212	0.183	0.063	-0.027	0.231	0.042	-0.083	0.074	0.003
HOMA-B	0.235	0.141	0.091	0.056	0.107	0.055	0.064	0.0314	0.13	-0.017	-0.035	0.008	0.07	-0.075	-0.042	0.026
LDL	0.083	0.163	-0.092	-0.079	-0.012	0.175	0.375**	0.23	-0.079	-0.173	-0.064	-0.088	0.013	0.035	-0.047	-0.178
HDL	0.080	0.08	0.030	-0.126	-0.181	-0.164	-0.064	-0.012	-0.128	0.002	0.157	-0.057	-0.205	0.127	-0.314*	0.134
Triglycerides	0.269	0.236	-0.072	-0.185	-0.079	0.055	0.114	0.078	0.13	-0.101	-0.102	-0.016	-0.107	-0.033	0.054	-0.033
Total cholesterol	0.158	0.195	-0.078	-0.132	-0.093	0.086	0.338*	0.224	-0.092	-0.177	0.008	-0.094	-0.089	0.081	-0.143	-0.09
NEFA	-0.151	-0.192	-0.381**	-0.334*	-0.529***	-0.271	0.147	-0.02	-0.195	-0.269	-0.138	-0.176	-0.175	-0.018	-0.142	-0.193
Glycerol	0.029	0.016	-0.207	-0.135	-0.369*	-0.173	0.169	0.026	-0.089	-0.199	0.0048	-0.155	-0.187	0.076	-0.024	-0.084
Lactate	0.144	0.244	0.063	0.234	0.029	-0.079	-0.069	0.052	0.033	0.01	0.213	0.203	-0.254	0.243	0.161	0.264
CRP	0.146	0.18	0.217	0.185	0.268	0.139	0.244	0.256	0.011	-0.002	0.078	0.278	0.087	0.085	0.237	0.186
Leptin	0.222	0.179	0.263	0.233	0.311?*	0.321*	0.372**	0.359*	-0.055	-0.074	0.259	0.193	-0.114	0.243	0.310*	0.159
Adiponectin	-0.150	0.03	-0.071	-0.163	-0.154	0.029	0.104	-0.061	-0.058	0.097	0.23	-0.081	0.025	0.197	-0.273	-0.083
Methionine	1	0.612***	0.140	0.213	-0.016	0.424**	0.249	0.216	0.475***	0.39**	0.151	0.225	0.385**	-0.068	-0.02	0.024
SAM	0.612***	1	0.191	0.373**	0.051	0.499***	0.197	0.165	0.302*	0.387**	0.089	0.135	0.212	-0.01	-0.049	-0.095
SAH	0.140	0.191	1	0.249	0.603***	0.168	0.2	0.287*	0.14	0.348*	0.274	0.388**	0.12	0.091	0.183	0.371**
Adenosine	0.213	0.373**	0.249	1	0.41**	0.312*	0.175	0.351*	0.125	0.314*	0.342*	0.41**	0.174	0.204	0.178	0.108
Total homocysteine	-0.016	0.051	0.603***	0.410**	1	0.159	0.087	0.300*	0.151	0.222	0.241	0.570***	0.082	0.133	0.333*	0.274
Total Cysteine	0.424**	0.499***	0.168	0.312*	0.159	1	0.614***	0.551***	0.123	0.208	0.385**	0.224	0.206	0.311*	0.035	0.002
Free Cysteine	0.249	0.197	0.200	0.175	0.087	0.614***	1	0.708***	0.091	0.141	0.174	0.261	0.224	0.105	-0.064	-0.115
Cystine	0.216	0.165	0.287*	0.351*	0.300*	0.551***	0.708***	1	0.077	0.173	0.507***	0.513***	0.079	0.401**	0.307*	0.246
Total Reduced GSH	0.475***	0.302*	0.140	0.125	0.151	0.123	0.091	0.077	1	0.794***	-0.02	0.285*	0.715***	-0.442**	0.113	-0.356*
Free Reduced GSH	0.390**	0.387**	0.348*	0.314*	0.222	0.208	0.141	0.173	0.794***	1	0.226	0.425**	0.630***	-0.276	0.026	-0.155
GSSG	0.151	0.089	0.274	0.342*	0.241	0.385**	0.174	0.507***	-0.02	0.226	1	0.409**	-0.167	0.835***	0.211	0.431**
Cystinyl glycine	0.225	0.135	0.388**	0.41**	0.570***	0.224	0.261	0.513***	0.285*	0.425**	0.409**	1	0.141	0.194	0.257	0.316*
Total γ-Glutamyl-Cysteine	0.385**	0.212	0.120	0.174	0.082	0.206	0.224	0.079	0.715***	0.630***	-0.167	0.141	1	-0.494***	-0.054	-0.354*
Oxidized GSH	-0.068	-0.01	0.091	0.204	0.133	0.311*	0.105	0.401**	-0.442**	-0.276	0.835***	0.194	-0.494***	1	0.193	0.504***
3-Chloro-tyrosine	-0.020	-0.049	0.183	0.178	0.333*	0.035	-0.064	0.307*	0.113	0.026	0.211	0.257	-0.054	0.193	1	0.239
3-Nitro-tyrosine	0.024	-0.095	0.371**	0.108	0.274	0.002	-0.115	0.246	-0.356*	-0.155	0.431**	0.316*	-0.354*	0.504***	0.239	1

B - Correlation of clinical and biochemical parameters in Healthy Obese and Unhealthy Obese participants

	BMIz	WC	SBP	DBP	HR	Insulin	Glucose	HOMA-IR	HOMA-B	LDL	HDL	Triglycerides	Total cholesterol	NEFA	Glycerol	Lactate	CRP	Leptin	Adiponectin
BMIz	1	0.836***	0.374*	0.244	0.241	0.707***	0.060	0.692	0.540***	0.377*	-0.599***	0.314*	0.207	0.214	0.378*	0.220	0.473**	0.712***	-0.490***
WC	0.836***	1	0.430**	0.241	0.156	0.736***	-0.012	0.705***	0.606***	0.417**	-0.554***	0.442**	0.255	0.127	0.389*	0.219	0.542***	0.803***	-0.597***
SBP	0.374*	0.430**	1	0.557***	0.317*	0.484***	0.104	0.458**	0.445**	0.252	-0.125	0.190	0.264	-0.0245	0.043	0.362*	0.241	0.428**	-0.310*
DBP	0.244	0.241	0.557***	1	0.326*	0.218	0.081	0.228	0.243	0.208	-0.258	0.214	0.119	0.069	0.200	0.183	0.344*	0.191	-0.224
HR	0.241	0.156	0.317*	0.326*	1	0.177	0.344*	0.233	0.056	0.304	-0.203	0.239	0.255	0.075	-0.039	0.358*	-0.079	0.075	-0.120
Insulin	0.707***	0.736***	0.484***	0.218	0.177	1	0.0006	0.971***	0.847***	0.266	-0.447**	0.402**	0.165	-0.0306	0.264	0.425**	0.358*	0.694***	-0.682***
Glucose	0.060	-0.012	0.104	0.081	0.344*	0.0006	1	0.179	-0.412**	-0.047	-0.091	-0.086	-0.147	-0.089	-0.105	0.233	-0.212	-0.133	0.205
HOMA-IR	0.692	0.705***	0.458**	0.228	0.233	0.971***	0.179	1	0.722***	0.277	-0.471**	0.377*	0.149	0.003	0.248	0.444**	0.280	0.593***	-0.641***
HOMA-B	0.540***	0.606***	0.445**	0.243	0.056	0.847***	-0.412**	0.722***	1	0.298	-0.264	0.278	0.278	-0.021	0.259	0.344*	0.418**	0.663***	-0.670***
LDL	0.377*	0.417**	0.252	0.208	0.304	0.266	-0.047	0.277	0.298	1	-0.376*	0.562***	0.911***	0.304	0.400**	0.214	0.237	0.270	-0.193
HDL	-0.599***	-0.554***	-0.125	-0.258	-0.203	-0.447**	-0.091	(-)0.471**	-0.264	(-)0.376*	1	-0.442**	-0.028	0.007	-0.078	-0.037	-0.457**	-0.329*	0.368*
Triglycerides	0.314*	0.442**	0.190	0.214	0.239	0.402**	-0.086	0.377*	0.278	0.562***	-0.442**	1	0.484**	0.185	0.450**	0.302	0.238	0.385*	-0.333*
Total cholesterol	0.207	0.255	0.264	0.119	0.255	0.165	-0.147	0.149	0.278	0.911***	-0.028	0.484**	1	0.360*	0.404**	0.180	0.060	0.211	-0.064
NEFA	0.214	0.127	-0.0245	0.069	0.075	-0.0306	-0.089	0.003	-0.021	0.304	0.007	0.185	0.360*	1	0.580***	0.053	-0.004	0.099	-0.042
Glycerol	0.378*	0.389*	0.043	0.200	-0.039	0.264	-0.105	0.248	0.259	0.400**	-0.078	0.450**	0.404**	0.580***	1	0.276	0.174	0.358*	-0.396*
Lactate	0.220	0.219	0.362*	0.183	0.358*	0.425**	0.233	0.444**	0.344*	0.214	-0.037	0.302	0.180	0.053	0.276	1	-0.117	0.263	-0.242
CRP	0.473**	0.542***	0.241	0.344*	-0.079	0.358*	-0.212	0.280	0.418**	0.237	-0.457**	0.238	0.060	-0.004	0.174	-0.117	1	0.456**	-0.321*
Leptin	0.712***	0.803***	0.428**	0.191	0.075	0.694***	-0.133	0.593***	0.663***	0.270	-0.329*	0.385*	0.211	0.099	0.358*	0.263	0.456**	1	-0.553***
Adiponectin	-0.490***	-0.597***	-0.310*	-0.224	-0.12	-0.682***	0.205	-0.641***	-0.670***	-0.193	0.368*	-0.333*	-0.064	-0.042	-0.396*	-0.242	-0.321*	-0.553***	1
Methionine	-0.049	-0.085	-0.034	-0.091	-0.035	-0.054	-0.064	-0.037	-0.084	-0.085	0.066	0.144	0.007	0.028	-0.013	-0.113	-0.096	-0.023	0.116
SAM	0.013	-0.120	0.060	-0.010	0.026	-0.052	0.046	-0.036	-0.144	0.039	-0.029	0.101	0.051	0.041	-0.019	-0.105	0.036	-0.225	0.272
SAH	0.017	0.138	0.124	0.004	-0.101	0.162	0.210	0.165	0.091	-0.054	0.118	-0.142	-0.083	-0.193	-0.123	0.207	0.176	0.067	0.036
Adenosine	-0.082	-0.057	-0.099	-0.001	0.058	-0.012	0.047	-0.029	0.060	-0.003	0.073	-0.096	-0.028	-0.113	-0.118	0.117	0.084	-0.165	-0.012
Total homocysteine	-0.100	0.058	-0.083	-0.171	0.082	0.049	0.120	0.024	0.036	-0.033	-0.017	-0.121	-0.088	-0.339*	-0.287	0.044	0.129	-0.148	-0.030
Total Cysteine	0.147	0.059	0.096	-0.054	0.036	0.057	0.208	0.077	-0.151	0.059	-0.146	0.032	-0.011	-0.177	-0.209	-0.084	-0.051	-0.049	0.210
Free Cysteine	0.107	0.181	0.075	-0.056	-0.021	0.025	0.145	0.061	-0.071	0.283	0.0003	0.162	0.289	0.229	0.089	-0.036	0.038	0.126	0.159
Cystine	-0.053	0.145	0.041	-0.111	0.149	0.043	0.153	0.058	-0.028	0.176	0.217	0.154	0.245	0.179	0.004	0.088	0.036	0.086	0.038
Total Reduced GSH	0.024	-0.0001	-0.050	-0.120	-0.053	-0.008	-0.110	-0.002	-0.108	0.056	-0.046	0.068	0.083	0.069	-0.064	-0.222	-0.124	-0.012	0.111
Free Reduced GSH	-0.065	0.009	0.039	-0.090	-0.001	-0.062	0.129	-0.038	-0.204	0.005	0.008	-0.026	0.019	0.018	-0.139	-0.168	-0.011	-0.018	0.254
GSSG	-0.191	-0.142	-0.082	0.028	0.005	-0.163	0.218	-0.205	-0.192	-0.191	0.355*	-0.113	-0.100	-0.049	-0.036	0.163	-0.116	-0.038	0.324*
Cystinyl glycine	-0.146	-0.165	-0.059	-0.152	0.056	-0.101	0.436	-0.015	-0.315*	-0.103	0.068	0.008	-0.115	-0.010	-0.019	0.200	-0.008	-0.202	0.254
Total γ-Glutamyl-Cysteine	-0.028	-0.018	0.012	0.081	-0.065	-0.022	0.006	0.016	-0.049	0.073	-0.255	-0.087	0.009	-0.132	-0.301	-0.361*	0.059	-0.178	0.144
Oxidized GSH	-0.152	-0.129	-0.065	0.059	0.03	-0.131	0.156	-0.183	-0.116	-0.185	0.336*	-0.046	-0.099	-0.080	-0.018	0.227	-0.065	-0.065	0.226
3-Chloro-tyrosine	0.081	0.171	-0.021	-0.125	0.018	0.053	-0.041	0.016	0.010	0.018	-0.001	-0.041	-0.012	-0.039	-0.003	-0.124	0.070	0.219	-0.197
3-Nitro-tyrosine	-0.141	-0.123	-0.077	-0.001	-0.005	-0.025	0.123	-0.007	0.031	-0.046	0.065	0.083	-0.073	-0.171	0.054	0.238	0.119	-0.049	-0.096

	Methionine	SAM	SAH	Adenosine	Total homocysteine	Total Cysteine	Free Cysteine	Cystine	Total Reduced GSH	Free Reduced GSH	GSSG	Cystinyl glycine	Total γ-Glutamyl-Cysteine	Oxidized GSH	3-Chloro-tyrosine	3-Nitro-tyrosine
BMIz	-0.049	0.013	0.017	-0.082	-0.100	0.147	0.107	-0.053	0.024	-0.065	-0.191	-0.146	-0.028	-0.152	0.081	-0.141
WC	-0.085	-0.120	0.138	-0.057	0.058	0.059	0.181	0.145	-0.0001	0.009	-0.142	-0.165	-0.018	-0.129	0.171	-0.123
SBP	-0.034	0.060	0.124	-0.099	-0.083	0.096	0.075	0.041	-0.050	0.039	-0.082	-0.059	0.012	-0.065	-0.021	-0.077
DBP	-0.091	-0.010	0.004	-0.001	-0.171	-0.054	-0.056	-0.111	-0.120	-0.090	0.028	-0.152	0.081	0.059	-0.125	-0.001
HR	-0.035	0.026	-0.101	0.058	0.082	0.036	-0.021	0.149	-0.053	-0.001	0.005	0.056	-0.065	0.03	0.018	-0.005
Insulin	-0.054	-0.052	0.162	-0.012	0.049	0.057	0.025	0.043	-0.008	-0.062	-0.163	-0.101	-0.022	-0.131	0.053	-0.025
Glucose	-0.064	0.046	0.210	0.047	0.120	0.208	0.145	0.153	-0.110	0.129	0.218	0.436	0.006	0.156	-0.041	0.123
HOMA-IR	-0.037	-0.036	0.165	-0.029	0.024	0.077	0.061	0.058	-0.002	-0.038	-0.205	-0.015	0.016	-0.183	0.016	-0.007
HOMA-B	-0.084	-0.144	0.091	0.060	0.036	-0.151	-0.071	-0.028	-0.108	-0.204	-0.192	-0.315*	-0.049	-0.116	0.010	0.031
LDL	-0.085	0.039	-0.054	-0.003	-0.033	0.059	0.283	0.176	0.056	0.005	-0.191	-0.103	0.073	-0.185	0.018	-0.046
HDL	0.066	-0.029	0.118	0.073	-0.017	-0.146	0.0003	0.217	-0.046	0.008	0.355*	0.068	-0.255	0.336*	-0.001	0.065
Triglycerides	0.144	0.101	-0.142	-0.096	-0.121	0.032	0.162	0.154	0.068	-0.026	-0.113	0.008	-0.087	-0.046	-0.041	0.083
Total cholesterol	0.007	0.051	-0.083	-0.028	-0.088	-0.011	0.289	0.245	0.083	0.019	-0.100	-0.115	0.009	-0.099	-0.012	-0.073
NEFA	0.028	0.041	-0.193	-0.113	-0.339*	-0.177	0.229	0.179	0.069	0.018	-0.049	-0.010	-0.132	-0.080	-0.039	-0.171
Glycerol	-0.013	-0.019	-0.123	-0.118	-0.287	-0.209	0.089	0.004	-0.064	-0.139	-0.036	-0.019	-0.301	-0.018	-0.003	0.054
Lactate	-0.113	-0.105	0.207	0.117	0.044	-0.084	-0.036	0.088	-0.222	-0.168	0.163	0.200	-0.361*	0.227	-0.124	0.238
CRP	-0.096	0.036	0.176	0.084	0.129	-0.051	0.038	0.036	-0.124	-0.011	-0.116	-0.008	0.059	-0.065	0.070	0.119
Leptin	-0.023	-0.225	0.067	-0.165	-0.148	-0.049	0.126	0.086	-0.012	-0.018	-0.038	-0.202	-0.178	-0.065	0.219	-0.049
Adiponectin	0.116	0.272	0.036	-0.012	-0.030	0.210	0.159	0.038	0.111	0.254	0.324*	0.254	0.144	0.226	-0.197	-0.096
Methionine	1	0.618***	-0.114	0.006	-0.184	0.532***	0.354*	0.043	0.500***	0.458**	0.010	0.238	0.398**	-0.226	-0.301*	-0.251
SAM	0.618***	1	0.008	0.191	-0.126	0.713	0.38	0.039	0.254	0.437	-0.015	0.213	0.335	-0.135	-0.509	-0.286
SAH	-0.114	0.008	1	0.077	0.613	-0.026	0.042	0.117	-0.069	0.233	0.216	0.177	0.145	0.075	0.116	0.256
Adenosine	0.006	0.191	0.077	1	0.405**	0.147	0.154	0.321*	0.056	0.176	0.282	0.287	0.105	0.198	-0.01	0.13
Total homocysteine	-0.184	-0.126	0.613	0.405**	1	-0.032	-0.06	0.215	-0.014	0.127	0.133	0.265	0.096	0.101	0.265	0.309
Total Cysteine	0.532***	0.713	-0.026	0.147	-0.032	1	0.520***	0.198	0.282	0.434**	0.118	0.280	0.312*	-0.045	-0.332*	-0.257
Free Cysteine	0.354*	0.38	0.042	0.154	-0.06	0.520***	1	0.618***	0.326*	0.457**	0.020	0.385**	0.169	-0.155	-0.265	-0.25
Cystine	0.043	0.039	0.117	0.321*	0.215	0.198	0.618***	1	0.081	0.248	0.386**	0.363*	-0.151	0.285	0.245	0.115
Total Reduced GSH	0.500***	0.254	-0.069	0.056	-0.014	0.282	0.326*	0.081	1	0.665***	-0.008	0.252	0.571***	-0.377*	-0.031	-0.439**
Free Reduced GSH	0.458**	0.437	0.233	0.176	0.127	0.434**	0.457**	0.248	0.665***	1	0.232	0.448**	0.561***	-0.295	-0.066	-0.324*
GSSG	0.010	-0.015	0.216	0.282	0.133	0.118	0.020	0.386**	-0.008	0.232	1	0.295	-0.200	0.807***	0.151	0.388**
Cystinyl glycine	0.238	0.213	0.177	0.287	0.265	0.280	0.385**	0.363*	0.252	0.448**	0.295	1	0.151	0.065	-0.049	0.135
Total γ-Glutamyl-Cysteine	0.398**	0.335	0.145	0.105	0.096	0.312*	0.169	-0.151	0.571***	0.561***	-0.200	0.151	1	-0.528***	-0.213	-0.352*
Oxidized GSH	-0.226	-0.135	0.075	0.198	0.101	-0.045	-0.155	0.285	-0.377*	-0.295	0.807***	0.065	-0.528***	1	0.129	0.520***
3-Chloro-tyrosine	-0.301*	-0.509	0.116	-0.01	0.265	-0.332*	-0.265	0.245	-0.031	-0.066	0.151	-0.049	-0.213	0.129	1	0.114
3-Nitro-tyrosine	-0.251	-0.286	0.256	0.13	0.309	-0.257	-0.25	0.115	-0.439**	-0.324*	0.388**	0.135	-0.352*	0.520***	0.114	1

Legend

BMIz	BMI *z* -score
WC	Waist Circumference
SBP	Systolic Blood Pressure
DBP	Diastolic Blood Pressure
HR	Heart Rate
LDL	Low-Density Lipoprotein
HDL	High-Density Lipoprotein
NEFA	Non-Esterified Fatty Acid:
CRP	C-Reactive Protein
SAM	S-Adenosylmethyonine
SAH	S-Adenosylhomocysteine
GSH	Reduce Glutathione
GSSG	Oxidized Glutathione

The analysis was performed using the Spearman's correlation (*rho*) and the *p*- value was considered significant:

* p<0.05

** p<0.01

*** p<0.001
