# Supplementary material for: Redox Imbalance and Methylation Disturbances in Early Childhood Obesity
Source: Oxid Med Cell Longev. 2021 Aug 17;2021:2207125. doi: 10.1155/2021/2207125 (PMC8387800; doi:10.1155/2021/2207125)
Supplement: Supplementary 2 — Supplementary Table 2: complete list of differentially methylated positions (DMPs). [file 2207125.f2.pdf]

DMP	Chromosome	Position	Genes	Δβ	Log_2 Fold Change	FDR
**cg06905516**	8	23022704	TNFRSF10D	-0.0658172	-0.118573078386117	0.0406335022523768
**cg08608222**	4	1795499	FGFR3;FGFR3;FGFR3	0.0394691	0.974745939210874	0.0406335022523768
**cg00941856**	10	101674581	DNMBP	-0.111603	-0.203924257833641	0.0406335022523768
**cg24345551**	6	33360652	KIFC1	-0.0843615	-0.169964842922027	0.0406335022523768
**cg04798796**	10	88855483	GLUD1;FAM35A	0.0128459	0.78019703680129	0.0406335022523768
**cg23638556**	13	111010230	COL4A2	-0.0352772	-0.0550357484555059	0.0406335022523768
**cg21183329**	9	124976219	LHX6;LHX6	0.0138929	0.74410393169284	0.0406335022523768
**cg21545248**	5	149402498	HMGXB3	-0.118147	-0.21719840047772	0.0406335022523768
**cg14774480**	11	102653516	WTAPP1	-0.0418546	-0.0654491768029718	0.0406335022523768
**cg12792397**	16	30703104		0.0183593	0.0277728147568009	0.0406335022523768
**cg06680214**	6	33422417	ZBTB9;ZBTB9	-0.00219952	-0.381958984086785	0.0406335022523768
**cg25025968**	11	4116056	RRM1;RRM1	0.016294	0.719573700587054	0.0406335022523768
**cg22624255**	19	19779476	ZNF101	0.0310168	1.09645945215441	0.0406335022523768
**cg22496450**	13	61191016		-0.0978919	-0.219409006045832	0.0406335022523768
**cg19793131**	11	96209225	JRKL-AS1	-0.059185	-0.100001142046094	0.0406335022523768
**cg20839991**	6	33088941	HLA-DPB2	-0.0333946	-0.0517411832043036	0.0406335022523768
**cg26328741**	16	2021947	TBL3	0.022945	0.79535118403304	0.0406335022523768
**cg25261059**	19	44031185	ETHE1	0.0651899	0.359116864511103	0.0406335022523768
**cg11908852**	1	38397237	INPP5B	0.0118427	0.6256883641268	0.0406335022523768
**cg01039133**	7	105752333	SYPL1;SYPL1	0.00909396	0.489729674847109	0.0406335022523768
**cg11483359**	6	19712715		-0.122321	-0.225732875760143	0.0406335022523768
**cg00430138**	11	11688731		-0.0464161	-0.078429240479446	0.0406335022523768
**cg17309592**	3	49591741	BSN	0.063166	0.656666673987389	0.0406335022523768
**cg03804985**	9	130159694	SLC2A8	0.0218871	0.957584345105923	0.0406335022523768
**cg19367466**	12	123717773	C12orf65;C12orf65	0.0239812	0.645296872926284	0.0406335022523768
**cg25195309**	1	225766155	ENAH;ENAH	-0.079411	-0.157638423092724	0.0406335022523768
**cg27461942**	13	100229148	LINC01039	-0.0875736	-0.166330640866549	0.0406335022523768
**cg13985868**	9	116102304	WDR31;WDR31	0.0320005	0.901413470591093	0.0406335022523768
**cg04982432**	6	38306180	BTBD9;BTBD9;BTBD9;BTBD9	-0.0823567	-0.151053215855891	0.0406335022523768
**cg26836121**	4	68388804	CENPC	-0.0492055	-0.0818722905008148	0.0406335022523768
**cg05919975**	12	57148848		-0.124588	-0.250471131538006	0.0406335022523768
**cg01395424**	5	1277474	TERT;TERT	0.0260448	0.0419079803954786	0.0406335022523768
**cg19690486**	19	14610252		-0.0805528	-0.144023830857933	0.0406335022523768
**cg22118655**	22	37823656	ELFN2	0.0533956	0.406558154153351	0.0415761721086483
**cg09693311**	14	54936086		-0.113309	-0.213021346732731	0.043769606288123
**cg19816107**	7	33101939	NT5C3;NT5C3	0.019855	0.537673887517361	0.043769606288123
**cg22735125**	5	49725470	EMB	-0.0469106	-0.0944772066336053	0.043769606288123
**cg26619035**	7	87936923	STEAP4	-0.0302796	-0.04642786995791	0.043769606288123
**cg01958008**	3	128597156	ACAD9	-0.0734059	-0.130324043830873	0.043769606288123
**cg06619047**	16	8772199	ABAT	-0.0438772	-0.0710378272646915	0.043769606288123
**cg19485344**	16	53412025	LOC102723373	-0.0995825	-0.210052229134053	0.043769606288123
**cg16328610**	6	83777335	DOPEY1	0.0232185	0.568984409845287	0.043769606288123
**cg17691292**	8	95654019	ESRP1;ESRP1;ESRP1;ESRP1;ESRP1	0.0169181	0.887744460855561	0.043769606288123
**cg01068252**	9	128114429	GAPVD1;GAPVD1;GAPVD1;GAPVD1	-0.061445	-0.104004747005541	0.043769606288123
**cg09544878**	5	133747733	CDKN2AIPNL	0.0150824	0.595688483793804	0.043769606288123
**cg12922647**	15	31396316	TRPM1	-0.0459696	-0.0740164846463773	0.043769606288123
**cg00966078**	1	23079320	EPHB2;EPHB2	-0.0717216	-0.119471672795221	0.043769606288123
**cg11784214**	17	19479935	SLC47A1	-0.0493608	-0.0826485677275705	0.043769606288123
**cg08558652**	16	48389438	LOC100507577;MIR548AE2;MIR5095	-0.0648793	-0.119332688869588	0.0440437062548208
**cg03754076**	1	232766254		0.0120227	0.883691752896239	0.0443669184970106
**cg12022339**	19	52692933	PPP2R1A;PPP2R1A	0.00728751	0.596344457004661	0.0443669184970106
**cg06444855**	5	38819991	OSMR-AS1	-0.129331	-0.251621936761016	0.0447999812308717
**cg18141295**	1	193144639	CDC73	-0.0581567	-0.102891227021625	0.044983718150736
**cg27190839**	11	33097035	LOC283267	-0.0825291	-0.145795379258764	0.046223009450485
**cg09556666**	15	72759065		-0.130132	-0.221196855567574	0.046223009450485
**cg13828275**	20	32972747	ITCH;ITCH;ITCH	-0.104588	-0.203791900141478	0.046223009450485
**cg07397958**	15	49476141	GALK2;GALK2;GALK2;GALK2	-0.0984144	-0.196665845869217	0.046223009450485
**cg01954686**	7	150885654	ASB10;ASB10;ASB10	-0.0878873	-0.203971896025394	0.046223009450485
**cg16203711**	3	169491183	MYNN;MYNN	0.0333015	1.24546553253385	0.046223009450485
**cg19870125**	12	129604022	TMEM132D	-0.0774623	-0.129662360254565	0.046223009450485
**cg26654766**	10	13343607	PHYH	-0.0619288	-0.1256361516786	0.046223009450485
**cg11473272**	3	125146553		-0.0332096	-0.0535671573005014	0.0469518271538882
**cg10400226**	19	8325369	CERS4	-0.0889155	-0.172782903719894	0.046991736038697
**cg15403961**	4	173905761	GALNTL6	-0.0976913	-0.211795241982064	0.046991736038697
**cg03365985**	4	42399484	SHISA3	0.00778769	0.677606960549186	0.046991736038697
**cg18749273**	15	68569993	FEM1B	0.0211876	0.686438817129089	0.046991736038697
**cg04783204**	6	44191600	SLC29A1;SLC29A1;SLC29A1;SLC29A1;SLC29A1;SLC29A1	0.0166506	0.661884182897757	0.046991736038697
**cg23727374**	20	30946786	ASXL1;ASXL1	0.0259131	0.829473915468712	0.046991736038697
**cg26332016**	12	117348108	FBXW8;FBXW8	-0.0935664	-0.168735547779607	0.046991736038697
**cg04697629**	1	221871655		0.00667726	0.00983533882842333	0.047418340765503
**cg00495931**	20	43545321	PABPC1L	0.00975951	0.0144618167571059	0.047418340765503
**cg24390275**	6	56860507	BEND6	-0.0507518	-0.082357266421499	0.047418340765503
**cg15609150**	5	179197801	MAML1	-0.127225	-0.314322405942136	0.0475625159781643
**cg10351795**	3	133744113	SLCO2A1	-0.0582335	-0.101351763105253	0.0475625159781643
**cg15539395**	19	59086703	MGC2752	0.0111455	0.444479205196505	0.0475625159781643
**cg21787516**	7	105018109	SRPK2	-0.0585389	-0.121459271970044	0.0475625159781643
**cg14595269**	7	151216272	RHEB	0.0624945	0.459211656415842	0.0501968659474078
**cg24808111**	7	148889824		-0.0930476	-0.163425674175079	0.0502645183457041
**cg14819132**	17	17495032	PEMT	0.0110168	0.552637080591458	0.0503267269163535
**cg03425878**	16	67038709	CES4A;CES4A;CES4A;CES4A;CES4A;CES4A	0.0075249	0.011103343569635	0.051809519065246
**cg02871554**	18	21170124		-0.0730601	-0.148204424576289	0.051809519065246

**cg01897092**	17	34057222	RASL10B	-0.0929732	-0.182672713771587	0.051809519065246
**cg05118364**	16	3533812	NAT15;NAT15;NAT15	-0.0846287	-0.190323684358396	0.0518992411265714
**cg07218663**	6	146350618	GRM1;GRM1	0.0215767	0.759816205989132	0.0518992411265714
**cg14870958**	14	73029842	RGS6	-0.0169623	-0.0262293277588816	0.0518992411265714
**cg06817090**	1	1334334	CCNL2;CCNL2;CCNL2;LOC148413;CCNL2	0.00788647	0.658423581555615	0.0518992411265714
**cg21197273**	3	11760785	VGLL4;VGLL4	0.00674041	0.329675701578179	0.0518992411265714
**cg19926395**	7	150865207	GBX1	0.0131627	0.756565955490245	0.0518992411265714
**cg24736951**	3	171114259	TNIK;TNIK;TNIK;TNIK;TNIK;TNIK;TNIK;TNIK;TNIK	-0.0327439	-0.0513963795834923	0.0518992411265714
**cg24743229**	1	110577591	FAM40A	0.0149933	0.610187961362712	0.0518992411265714
**cg00552822**	15	22368443	LOC727924;OR4M2	-0.0467413	-0.0811544306242111	0.0518992411265714
**cg11069461**	7	64646466	INTS4L1	-0.0602762	-0.103756172770022	0.0518992411265714
**cg17381040**	19	36102347	HAUS5	-0.0438344	-0.0675052757877374	0.0518992411265714
**cg14028272**	1	229533222		-0.0426454	-0.0676282831023415	0.0518992411265714
**cg16420045**	15	65350816	RASL12;RASL12	0.0125497	0.018931894521605	0.0518992411265714
**cg10148231**	7	849153		-0.0837718	-0.157466745738593	0.0518992411265714
**cg25019777**	4	53525403	USP46;USP46	0.0638011	0.747612200717906	0.0518992411265714
**cg06942121**	16	2040985	SYNGR3	0.0133021	0.352113195237665	0.0518992411265714
**cg07437492**	1	240434057	FMN2;FMN2	-0.0795417	-0.137991784328019	0.0518992411265714
**cg03436558**	16	75657345	ADAT1	0.0156625	0.670138522872142	0.0518992411265714
**cg13441599**	7	102715243	ARMC10;ARMC10;ARMC10;ARMC10;ARMC10;ARMC10;FBXL13;FBXL13;FBXL13;FBXL	0.0143272	0.707199146685249	0.0521912828346582
**cg00765721**	2	159314180	CCDC148;CCDC148;PKP4;PKP4	0.0197356	0.847271540952859	0.0523427652535012
**cg25188141**	2	128633711	AMMECR1L	-0.0449683	-0.0726934465720912	0.0523427652535012
**cg11913696**	14	77932535	AHSA1	-0.0401746	-0.06272643096699	0.0530636463671279
**cg04214520**	3	195615359	TNK2;TNK2	0.00926496	0.0141591576252101	0.0535932816111014
**cg26077228**	17	80408536	C17orf62;C17orf62;C17orf62	0.020084	0.57835457517611	0.0540431130494622
**ch.7.53629068R**	7	53661574		0.0157414	0.574218083468763	0.0540431130494622
**cg23911661**	1	45674993		-0.0631038	-0.107431194794865	0.0548512093997217
**cg22829819**	16	69332313	SNTB2	-0.0251278	-0.0387431379226169	0.0553078981147217
**cg14438445**	2	39221176	1 SOS	-0.0805308	-0.147591525871211	0.055813348309455
**cg26219201**	19	54585100	TARM1	-0.03537	-0.0573306766347431	0.055813348309455
**cg23169308**	11	118881590	CCDC84;CCDC84;CCDC84;CCDC84	-0.0480654	-0.0779793156481695	0.055813348309455
**cg22541808**	17	40687226	NAGLU	-0.0954657	-0.199091471098774	0.055813348309455
**cg13062138**	5	84374644		-0.0619955	-0.111361583050029	0.055813348309455
**cg14763763**	22	24968468	SNRPD3	-0.0388004	-0.060877816398849	0.055813348309455
**cg16268848**	2	109744586	SH3RF3;LOC100287216	0.0105337	0.537819359257178	0.055813348309455
**cg26750487**	1	207818449	CR1L	0.0173059	0.514371026201175	0.055813348309455
**cg27506538**	17	66588055	FAM20A;FAM20A	-0.09743	-0.187269246506453	0.055813348309455
**cg09471916**	16	3131034		-0.029647	-0.047122895019374	0.055813348309455
**cg13255896**	16	81352678	GAN	-0.069034	-0.12444963085618	0.055813348309455
**cg09033276**	3	195959363	SLC51A	0.00838916	0.0124007059740016	0.055813348309455
**cg23687258**	6	106808749		0.0275197	0.609720799061538	0.055813348309455
**cg10588706**	1	246305649	SMYD3;SMYD3	-0.0645656	-0.102507258072371	0.055813348309455
**cg20320896**	7	144534283	TPK1;TPK1	-0.0345839	-0.0544971708448443	0.055813348309455
**cg18887033**	2	7005947	CMPK2	0.0648362	0.378340898658906	0.055813348309455
**cg23330385**	2	187385911		-0.0504405	-0.082993378432526	0.055813348309455
**cg22012693**	8	139919163	COL22A1	-0.123828	-0.239281194342031	0.055813348309455
**cg09692037**	2	16502224		0.00487629	0.00718098190072974	0.055813348309455
**cg27222589**	12	52968762	KRT74	-0.0522376	-0.0859733717005815	0.055813348309455
**cg06069179**	17	33288421	CCT6B;CCT6B;ZNF830	0.00863542	0.861337257139569	0.055813348309455
**cg22890950**	10	122216135	PPAPDC1A	0.0309575	1.03127730117504	0.055813348309455
**cg09093477**	5	34839189	TTC23L	0.010227	0.508915841176316	0.055813348309455
**cg18792956**	3	34200625		-0.0600788	-0.123588531442679	0.055813348309455
**cg08877624**	8	141521446	CHRAC1;CHRAC1;CHRAC1	0.042238	0.455131570473645	0.055813348309455
**cg00730348**	13	51482465	RNASEH2B;RNASEH2B	-0.070874	-0.133622260750994	0.055813348309455
**cg19522644**	18	8717905	KIAA0802	0.0190401	0.0288183961717389	0.055813348309455
**cg09621945**	8	28463758		-0.0835957	-0.147817735356274	0.055813348309455
**cg26187342**	17	65713858	NOL11	0.0712932	0.375895822034881	0.0559831905624135
**cg08395298**	4	185827243		-0.0978363	-0.200887195938918	0.0559831905624135
**cg15910486**	5	142783621	NR3C1;NR3C1;NR3C1;NR3C1;NR3C1;NR3C1;NR3C1	0.02354	0.426344268044438	0.0559831905624135
**cg00665045**	13	106158958	DAOA-AS1	-0.0471648	-0.0767215919000765	0.0559831905624135
**cg07576222**	15	67357975	SMAD3	0.0344758	0.422248401839587	0.0559831905624135
**cg01572334**	3	110790939	PVRL3;PVRL3;PVRL3	0.0125951	0.657405463127045	0.0559831905624135
**cg11081703**	12	108909056	FICD;FICD	0.0191671	0.557785526129723	0.0559831905624135
**cg27133310**	3	146263336	PLSCR1	-0.0775021	-0.155572325606891	0.0559831905624135
**cg25221988**	16	1355400		-0.0635851	-0.110957098198338	0.0559831905624135
**cg24829631**	1	19639165	PQLC2;PQLC2;PQLC2;AKR7A2;PQLC2;PQLC2;PQLC2	0.0247533	0.764576472714612	0.0559831905624135
**cg27233071**	2	108964526		-0.110408	-0.287470836989028	0.0559831905624135
**cg01866220**	16	28969554	MIR4517;NFATC2IP	-0.0667816	-0.114494766671256	0.0559831905624135
**cg03795316**	10	12110955	DHTKD1;DHTKD1	0.0193955	0.936741597213522	0.0559831905624135
**cg07490276**	14	70233797	SFRS5;SFRS5;LOC100289511	0.0112441	0.313883441808425	0.0559831905624135
**cg00538011**	15	90838172		-0.0185722	-0.0283914846492332	0.0559861534840211
**cg08276976**	15	39411127		-0.107957	-0.219596349971089	0.0559861534840211
**cg01739401**	12	51208362	ATF1	-0.092756	-0.165894002593466	0.0559861534840211
**cg11899080**	17	64455086	PRKCA	-0.0675417	-0.115111713254451	0.0559861534840211
**cg03790378**	11	47278831	NR1H3;NR1H3;NR1H3	-0.0206605	-0.0307661054909767	0.0559861534840211
**cg23162960**	5	175875209	FAF2	0.00646354	0.673689128968056	0.0559861534840211
**cg10309250**	17	42218412	C17orf53;C17orf53	-0.0614571	-0.120505086421667	0.0559861534840211
**cg13255559**	1	195950882		-0.0710974	-0.132940044647617	0.0559861534840211
**cg08220548**	18	76160443		-0.0522116	-0.0845241873178414	0.0559861534840211
**cg20154431**	8	99053241		-0.0782299	-0.151207623236585	0.0559861534840211
**cg02956320**	2	169643050	NOSTRIN;NOSTRIN	-0.0732048	-0.136584361306329	0.0559861534840211
**cg09907201**	12	56237315	MMP19	-0.0772043	-0.138938635593102	0.0559861534840211

**cg01341130**	15	76033361	DNM1P35	-0.079231	-0.167513972963776	0.0559861534840211
**cg00108459**	10	71123414	HK1;HK1;HK1;HK1;HK1	-0.0681939	-0.12684526669541	0.0559861534840211
**cg07801516**	19	37154552	ZNF461	-0.100201	-0.296999147055265	0.0559861534840211
**cg07031794**	2	26568949	SELI;GPR113	0.0422561	0.418574728012646	0.0559861534840211
**cg16192320**	1	206680748	RASSF5;RASSF5	0.0335402	0.452700818756738	0.0559861534840211
**cg22592142**	15	45409170	DUOXA2	0.0293893	0.616817913138339	0.0559861534840211
**cg08968899**	8	98961821	MATN2;MATN2	-0.03825	-0.0642354844113241	0.0559861534840211
**cg00214165**	5	7869652	MTRR;FASTKD3;MTRR	0.0259222	0.617252421617214	0.0559861534840211
**cg03932842**	7	65703474	TPST1	-0.0639981	-0.127581848572873	0.0559861534840211
**cg19733916**	14	89730642	FOXN3;FOXN3	-0.0656685	-0.135941820180429	0.0559861534840211
**cg05961166**	21	26864304		-0.0781734	-0.133027134682021	0.0559861534840211
**cg15944824**	17	40981088		-0.0818012	-0.184848502141435	0.0559861534840211
**cg05064347**	21	44395663	PKNOX1;PKNOX1	0.0161528	0.993587504895866	0.0563145121749649
**cg03926815**	3	13114681	IQSEC1	0.052254	0.193044722906818	0.0572868736734894
**cg15137369**	8	105601862	LRP12;LRP12	0.0417737	0.769453792753036	0.0572868736734894
**cg09466312**	4	580474		-0.0537069	-0.0888681790125446	0.0572868736734894
**cg14084272**	19	21654965		-0.0418435	-0.0656111977576899	0.0572868736734894
**cg11212901**	17	22020759		-0.055798	-0.097512181749258	0.0572868736734894
**cg23137531**	13	113622708	MCF2L	0.0100786	0.618366978673968	0.0572868736734894
**cg25950438**	14	34077154	NPAS3;NPAS3;NPAS3;NPAS3	-0.11711	-0.266786021306068	0.0572868736734894
**cg25601661**	11	72525334	ATG16L2	0.0413164	0.451617929244009	0.0572868736734894
**cg10084513**	3	122745685	SEMA5B	0.0123589	0.453920361553204	0.0572868736734894
**cg20630971**	6	159308131		-0.0644269	-0.116343624591644	0.0572868736734894
**cg21051843**	1	211572088	LINC00467	-0.0395697	-0.0664063532873141	0.0572868736734894
**cg10998122**	11	64008466	FKBP2;FKBP2;FKBP2;FKBP2	0.0245015	0.656283876772511	0.0572868736734894
**cg06360875**	11	22210253		-0.0729352	-0.130297182369274	0.0572868736734894
**cg08759723**	12	92027881		-0.0302957	-0.0468642490998552	0.0572868736734894
**cg02338410**	2	201763187	NIF3L1;NIF3L1;NIF3L1;NIF3L1	-0.0714224	-0.134044703312335	0.0572868736734894
**cg08926493**	8	55224461		-0.0834109	-0.143967132189412	0.0572868736734894
**cg25133757**	16	23699709	PLK1	-0.0283159	-0.0431774492361052	0.0572868736734894
**cg07231398**	14	106671426		-0.0798193	-0.165086995900561	0.0572868736734894
**cg15730068**	4	164394686	TKTL2	-0.031713	-0.0507374129267868	0.0572868736734894
**cg07570055**	2	233387088		0.0282292	0.726409712228598	0.0572868736734894
**cg01968242**	8	98787927	LAPTM4B;LAPTM4B	0.00940685	0.586594126021433	0.0572868736734894
**cg21434114**	18	3450282	TGIF1;TGIF1;TGIF1;TGIF1;TGIF1;TGIF1;TGIF1;TGIF1	0.0114361	0.493880400298997	0.0572868736734894
**cg06053850**	11	76510871		-0.0923927	-0.214695995802536	0.0572868736734894
**cg00868299**	9	92306213	UNQ6494	-0.0576747	-0.0944968190714708	0.0572868736734894
**cg27361617**	21	32470484		-0.0773972	-0.146996677296586	0.0572868736734894
**cg01320780**	8	22298453	PPP3CC	0.0133771	0.489587767704275	0.0572868736734894
**cg05781820**	11	65090854		-0.056122	-0.102254050733824	0.0572868736734894
**cg08150885**	12	106751428	POLR3B;POLR3B	0.00457615	0.351692989621085	0.0572868736734894
**cg26011210**	12	122125276		0.0423985	0.753624581929061	0.0572868736734894
**cg16559448**	5	179870052		-0.0865249	-0.18039597338438	0.0576655229937819
**cg23273364**	2	198364778	HSPE1;HSPE1;HSPD1;HSPD1	0.0274053	1.04798103486208	0.0582213687880469
**cg01065278**	1	9491741		-0.058609	-0.0980094395562806	0.0583086963318465
**cg14853169**	1	11113080		0.0679488	0.680335664495851	0.0585466095117296
**cg09870152**	1	171103714		-0.0917219	-0.249045633082101	0.0585466095117296
**cg00295948**	9	124261807	GGTA1	0.021646	0.373596405593771	0.0585466095117296
**cg09419983**	3	148847273	HPS3	0.0158378	0.77101330968801	0.0585466095117296
**cg18614025**	7	8008335	GLCCI1	0.00539701	0.550174032587653	0.0585466095117296
**cg23052020**	6	106960170	AIM1;AIM1	0.00848893	0.613042783675979	0.0585466095117296
**cg24289912**	10	129845739	PTPRE;PTPRE	0.00929646	0.54145860058691	0.0585466095117296
**cg27487046**	1	860613	SAMD11	0.00627432	0.569496353907217	0.0590698588315815
**cg02775882**	14	103210570		-0.0635624	-0.109015558470477	0.0590698588315815
**cg23887623**	15	91516823	PRC1;PRC1;PRC1	-0.0208621	-0.0312216941908148	0.0590698588315815
**cg00094875**	19	52597141	ZNF841	-0.0709819	-0.13849933196035	0.0590698588315815
**cg24962528**	5	177042842		-0.031059	-0.0474615882526214	0.0590698588315815
**cg03778373**	5	37378360	WDR70	-0.0574808	-0.0958585810082764	0.0590698588315815
**cg11517950**	20	42417382		-0.102964	-0.196851709235779	0.0590698588315815
**cg09902272**	22	41312942	XPNPEP3	-0.0740474	-0.127042959490653	0.0590698588315815
**cg21875532**	6	42109999	C6orf132	0.0277932	0.535112907324578	0.0590698588315815
**cg19991512**	15	44250851	FRMD5	-0.0761428	-0.202358134506238	0.0590698588315815
**cg21883042**	12	62997433	C12orf61;MIRLET7I	0.00793136	0.593537973673215	0.0590698588315815
**cg24080119**	19	45644694		0.0118379	0.0179166767240935	0.0590736506874234
**cg15541743**	4	3226527	HTT	0.00692507	0.010262902684241	0.0590736506874234
**cg06215939**	16	1755402	MAPK8IP3;MAPK8IP3	0.0653356	0.498130948193865	0.0590736506874234
**cg12290615**	6	71419788	SMAP1;SMAP1;SMAP1;SMAP1	-0.171948	-0.419744913077603	0.0590736506874234
**cg25104555**	10	16562998	C1QL3	0.0166443	0.84325669751052	0.0590736506874234
**cg20113500**	12	113773013	SLC24A6	0.0198791	0.49012150147729	0.0590736506874234
**cg27619817**	20	46832141		-0.0569653	-0.102916136260171	0.0590736506874234
**cg24602243**	5	14144425	TRIO	0.0227782	0.76598546812917	0.0590736506874234
**cg10206070**	11	67791338	ALDH3B1;ALDH3B1;ALDH3B1;ALDH3B1;ALDH3B1	-0.0646408	-0.116679797155084	0.0590736506874234
**cg18961533**	19	19302816	RFXANK;RFXANK;RFXANK;RFXANK;MEF2BNB;MEF2BNB;MEF2BNB-MEF2B;MEF2BN	0.0371958	0.467407161681932	0.0590736506874234
**cg08739385**	1	38201818	EPHA10	-0.101834	-0.18679233281253	0.0590736506874234
**cg15789385**	12	121907156	KDM2B;KDM2B	-0.099559	-0.214913651940593	0.0590736506874234
**cg25720688**	7	129295884	NRF1;NRF1;NRF1;NRF1	-0.0680516	-0.158306536178189	0.0592064708780651
**cg08941355**	7	27133106	HOXA1;HOXA1	-0.0848774	-0.17678297880615	0.0592064708780651
**cg16136840**	5	95997740	CAST;CAST;CAST;CAST;CAST	0.0370151	0.737971614156598	0.0592064708780651
**cg04139300**	20	40247070	CHD6;CHD6	0.0202726	0.786132542205494	0.0592064708780651
**cg00402311**	8	62282815	CLVS1	-0.140382	-0.279606653920519	0.0592064708780651
**cg12800150**	6	34216964	C6orf1;C6orf1;C6orf1	0.00855134	0.380341481219333	0.0592064708780651
**cg16947339**	16	30546198	ZNF747;ZNF747;ZNF747;ZNF747;ZNF747;ZNF747;ZNF747;ZNF747	0.0711946	1.49085637289442	0.0592064708780651

**cg25617568**	6	42277458	TRERF1;TRERF1	-0.0456295	-0.0799727490616643	0.0592064708780651
**cg26353328**	8	42010408	AP3M2;AP3M2	0.0146824	0.762366115140259	0.0592064708780651
**cg09357979**	4	25915643	C4orf52	0.029024	0.649940184091031	0.0592064708780651
**cg22855052**	12	6982535	SPSB2;SPSB2	0.0347824	0.51818059133792	0.0592064708780651
**cg02635865**	20	56885090	RAB22A;PPP4R1L	0.0417628	1.03762530406868	0.0592064708780651
**cg05016940**	14	53242413	GNPNAT1	-0.0643016	-0.107259369123023	0.0592064708780651
**cg06818627**	12	109536430	UNG;UNG	0.00936084	0.574606904226052	0.0592064708780651
**cg20840778**	17	79842807		-0.0468573	-0.0789396033678405	0.0592064708780651
**cg02606566**	1	93913524	FNBP1L;FNBP1L;FNBP1L	0.0110081	0.474923858775655	0.0592064708780651
**cg16870607**	2	219246302	SLC11A1	-0.0782657	-0.167790493780942	0.0592064708780651
**cg07463138**	15	83478369	WHAMM	0.00921612	0.598179451837082	0.0592064708780651
**cg04373646**	9	37484887	POLR1E;POLR1E	-0.0416564	-0.0672721129340991	0.05945973035251
**cg15128638**	3	124493484	ITGB5	-0.0692351	-0.125840480071182	0.05945973035251
**cg00656411**	7	140773422		0.0363735	0.924423836350291	0.05945973035251
**cg18571419**	4	187026278		0.0138643	0.353377627791481	0.05945973035251
**cg20710049**	10	22875490	PIP4K2A	-0.0592663	-0.0971172410829588	0.0597624091056663
**cg19409381**	4	83351505	HNRPDL;ENOPH1;HNRPDL	0.011771	0.615554395208228	0.0597624091056663
**cg12659851**	15	51988940	SCG3;SCG3	-0.091969	-0.189399114421771	0.0597624091056663
**cg24025893**	10	134756164	C10orf93	0.0488721	0.371463551267324	0.0597624091056663
**cg20036711**	13	21622707	LATS2	-0.0446717	-0.0718860626233614	0.0597624091056663
**cg16316472**	4	77997182	CCNI	0.0571518	0.897079791814894	0.0597624091056663
**cg08823305**	20	3451391	ATRN;ATRN;ATRN	0.0151967	0.708973312628139	0.0597624091056663
**cg13320890**	6	131186742	EPB41L2;EPB41L2;EPB41L2;EPB41L2;EPB41L2	-0.00785123	-0.0116873729796305	0.0598677893691087
**cg15482976**	2	174828973	SP3;SP3;SP3	0.0449751	0.509539288894447	0.0602094701420674
**cg25927259**	18	59857553	KIAA1468	-0.0459493	-0.0812055842355996	0.0604118995132999
**cg00789416**	14	101404296	SNORD113-5	-0.0192645	-0.0295144210910498	0.0606380399775694
**cg10442442**	2	127792200		-0.0362008	-0.0629044536578241	0.0606380399775694
**cg04356926**	3	98241413	CLDND1;CLDND1;CLDND1;CLDND1;CLDND1;CLDND1;CLDND1	0.0105104	0.679162522756574	0.0607265780570152
**cg21597684**	2	197457400	HECW2	0.0243919	0.79284259323085	0.0607265780570152
**cg15440854**	11	115041104		0.00265046	0.231611204633289	0.0607265780570152
**cg11575295**	7	158250911	PTPRN2;PTPRN2;PTPRN2	0.0801688	0.728764699360552	0.0607265780570152
**cg15331792**	1	45396742	EIF2B3;EIF2B3;EIF2B3	-0.0648126	-0.111570200727944	0.0607265780570152
**cg04049141**	16	20821573	LOC81691;LOC81691	-0.0389442	-0.0648518808757196	0.0607265780570152
**cg21942282**	11	64868530	VPS51;VPS51	-0.114124	-0.251769124809461	0.0607265780570152
**cg00449068**	6	107403532	BEND3	-0.073657	-0.134394509399797	0.0607265780570152
**cg16754853**	15	52868720		-0.0948652	-0.189807231410676	0.0607265780570152
**cg16415939**	1	236083208		-0.069	-0.160615695481897	0.0607265780570152
**cg04815242**	22	45330752	PHF21B;PHF21B	0.0101871	0.0152886263144311	0.0607265780570152
**cg13272676**	11	9404868	IPO7	-0.0806449	-0.142829700962754	0.0607265780570152
**cg18406718**	4	102269374	PPP3CA;PPP3CA;PPP3CA	0.0103213	0.593950074679615	0.0607265780570152
**cg21380365**	6	27865980		-0.0378429	-0.064300493177377	0.0607265780570152
**cg20509869**	2	220375605		0.0992099	0.9307915135266	0.0607265780570152
**cg02006147**	2	74734710	PCGF1	0.017485	0.875250926425979	0.0607265780570152
**cg00986191**	5	178419655	GRM6	-0.0383762	-0.0602786156597933	0.060827577018538
**cg24247472**	17	46908838	CALCOCO2	0.0275907	0.806705685160305	0.060827577018538
**cg20450471**	6	28584061		0.052707	0.476323735195727	0.060827577018538
**cg02662491**	3	165574116		-0.0460616	-0.0756247868068144	0.0614273661093166
**cg19943799**	20	40222546	CHD6	-0.0648263	-0.105771831693132	0.0614273661093166
**cg17643023**	18	55833162	NEDD4L;NEDD4L;NEDD4L;NEDD4L;NEDD4L;NEDD4L;NEDD4L;NEDD4L	-0.0531524	-0.0939954274240381	0.0614273661093166
**cg22983329**	22	48721504		-0.0911419	-0.175034382417776	0.0614273661093166
**cg23561934**	2	121199920		0.0292857	0.536942107603484	0.0614273661093166
**cg09431544**	19	14629187	DNAJB1;DNAJB1	0.0499302	0.342320746571312	0.0614273661093166
**cg07765536**	15	95647020		-0.104571	-0.325963720438853	0.0615371723654592
**cg04529860**	19	39900650		0.00772321	0.681721934718533	0.0615371723654592
**cg07264679**	12	7657396	CD163;CD163	-0.011904	-0.0176257216733763	0.0615371723654592
**cg14540546**	19	35738122	LSR;LSR;LSR;LSR;LSR	-0.0620919	-0.108522682916358	0.0615371723654592
**cg22218536**	3	40518595	ZNF619;ZNF619;ZNF619;ZNF619;ZNF619;ZNF619	0.0229823	0.605612492571369	0.0615371723654592
**cg07090025**	1	1510437	SSU72	0.017242	0.632690546647694	0.0615371723654592
**cg21959890**	10	94068044	MARCH5	-0.0611376	-0.105918098320094	0.0615371723654592
**cg07697561**	5	140998635	DIAPH1;DIAPH1	0.0224325	0.677662331460088	0.0615418159917198
**cg24809299**	20	23331479	NXT1;NXT1	0.015735	0.759195675991474	0.0615805888647651
**cg23279878**	1	43464711		-0.0732938	-0.119419630209819	0.0615805888647651
**cg04330631**	6	42018203	TAF8	0.0382713	0.731293710442021	0.0615805888647651
**cg09189978**	22	38240632	ANKRD54;MIR658	0.0477546	0.316745658965131	0.0615805888647651
**cg17155610**	1	150382400	RPRD2	-0.0664483	-0.155793507118404	0.0615805888647651
**cg25305397**	19	13816206		-0.0271587	-0.0427722831365354	0.0615805888647651
**cg18834652**	11	94276072	FUT4	-0.0410101	-0.0778705467207683	0.0615805888647651
**cg20054983**	11	124496361	TBRG1;TBRG1	0.00420474	0.00624974413311849	0.0615805888647651
**cg03835313**	7	27137427	HOTAIRM1;HOTAIRM1	-0.0828359	-0.193065046941273	0.0615805888647651
**cg25621735**	2	230933934	SLC16A14	-0.070467	-0.135780397983663	0.0615805888647651
**cg07163173**	2	67624373	ETAA1	0.0218845	0.844238503428439	0.0615805888647651
**cg26743529**	10	29134817	C10orf126	-0.09138	-0.194981973751015	0.0615805888647651
**cg23217542**	15	65958740	DENND4A;DENND4A	-0.0724072	-0.13975208364853	0.0615805888647651
**cg14294444**	9	128003255	HSPA5	0.00258769	0.316150650228386	0.0615805888647651
**cg18572609**	3	136150391	STAG1	-0.0361366	-0.0577354687186095	0.0615805888647651
**cg02400449**	22	38857584		0.00913851	0.51913276573753	0.0615805888647651
**cg03969724**	1	6114997	KCNAB2;KCNAB2	-0.105223	-0.223452703936744	0.0615805888647651
**cg00385596**	11	76088726	PRKRIR	-0.012873	-0.0192169461794712	0.0615805888647651
**cg06869501**	19	21541044	ZNF738	-0.0456296	-0.0856101686697788	0.0615805888647651
**cg15160573**	1	28908642	SNORA16A;SNHG12	0.0141655	0.546277230644772	0.0615805888647651
**cg03355286**	12	20962423	SLCO1B3	-0.102645	-0.240502047597891	0.0615805888647651
**cg01626918**	12	75978785		-0.0541841	-0.094112690154776	0.0615805888647651

**cg07835289**	1	236030215	LYST;LYST	0.0254648	0.494377554501753	0.0615805888647651
**cg06339248**	11	57435803	ZDHHC5	0.0321551	1.07049594376483	0.0618218480558689
**cg14550101**	1	155247361	HCN3	0.006041	0.473896035793199	0.0622827562307827
**cg06183295**	3	108855390		-0.0773594	-0.155931999803372	0.0622827562307827
**cg15662608**	15	88247247		-0.0925302	-0.16914784585083	0.0623363912408582
**cg19197830**	2	170681574	METTL5	0.0174719	0.470928764297447	0.0623363912408582
**cg16578267**	1	937048	HES4;HES4	0.0203597	0.427381230628676	0.0623363912408582
**cg14336526**	11	30367246		-0.0472233	-0.0771379867816901	0.0623363912408582
**cg02463478**	16	30798048	ZNF629	0.00895012	0.515955851552336	0.0623363912408582
**cg02761778**	16	69643871	NFAT5;NFAT5;NFAT5;NFAT5;NFAT5	-0.0697791	-0.132105086937518	0.0623363912408582
**cg23171573**	15	41099257	ZFYVE19;DNAJC17	0.0316871	0.555342526545468	0.0623363912408582
**cg09704553**	12	27167052	TM7SF3	0.0427366	1.02636735118041	0.0623363912408582
**cg09414262**	22	19594239		-0.0700842	-0.122094624906562	0.0623363912408582
**cg18330222**	15	100831929	ADAMTS17	-0.0660202	-0.122121861524955	0.0623363912408582
**cg05249419**	15	78855388		-0.0652454	-0.11735162974594	0.0623363912408582
**cg25380713**	14	69927647	SLC39A9;SLC39A9;SLC39A9;SLC39A9;SLC39A9	-0.0242119	-0.0370474632317422	0.0623363912408582
**cg15619333**	12	133759653	ZNF268;ZNF268;ZNF268;ZNF268;ZNF268;ZNF268;ZNF268;ZNF268;ZNF268	-0.113064	-0.202075581886034	0.0623363912408582
**cg00250391**	11	93850000		-0.0487262	-0.0844331726692717	0.0623363912408582
**cg16117070**	2	20547854		-0.0580299	-0.135170718642343	0.0623363912408582
**cg20878463**	15	58358758	ALDH1A2;ALDH1A2;ALDH1A2	0.0391605	0.557022757666626	0.0623363912408582
**cg03053914**	1	173837504	SNORD75;SNORD74;GAS5;ZBTB37;ZBTB37;ZBTB37;ZBTB37	0.0531258	0.718292162084865	0.0623363912408582
**cg17615565**	11	11454265	GALNT18	0.00398016	0.00582609562810245	0.0623363912408582
**cg17217228**	14	63203565	KCNH5;KCNH5	-0.0748201	-0.166719586734686	0.0623363912408582
**cg12126869**	10	346505	DIP2C	-0.0427008	-0.0683349719457728	0.0623363912408582
**cg17390355**	22	30228436	ASCC2;ASCC2	-0.0239137	-0.0366581283759943	0.0623363912408582
**cg23528353**	9	35605358	TESK1;TESK1	0.0443949	0.516876448595069	0.0623363912408582
**ch.14.1499103R**	14	93412334	ITPK1;ITPK1;ITPK1	0.0131298	1.00472934704947	0.0623363912408582
**cg00380172**	6	148663585	SASH1	0.0196525	0.450047095322357	0.0623363912408582
**cg26258631**	2	190648605	PMS1;ORMDL1;ORMDL1;PMS1;PMS1	0.00690641	0.516397307215803	0.0623363912408582
**cg19571034**	11	125462736	STT3A	0.0204544	0.721853534293137	0.0623363912408582
**cg05075708**	6	32940440	BRD2;BRD2	0.00935526	0.670069922213361	0.0623363912408582
**cg01794932**	1	1209696	UBE2J2;UBE2J2;UBE2J2;UBE2J2	0.0162833	0.832328888245868	0.0623363912408582
**cg17819963**	10	135191943	PAOX;PAOX;PAOX	0.0176928	0.529037838161361	0.0626020306422343
**cg13109045**	14	105218947	SIVA1;SIVA1	0.0230246	0.925466337422788	0.0627142914522418
**cg05947505**	10	102800464	SFXN3	-0.0614374	-0.113760866657757	0.0627142914522418
**cg06142946**	11	17373124	DKFZp686O24166	0.0030447	0.310066616245669	0.0627142914522418
**cg18281016**	19	58790371	ZNF8;ZNF8	0.0237097	0.508437581725871	0.0627142914522418
**cg05065230**	3	49395807	GPX1;GPX1	0.0117197	0.650534173882929	0.0627142914522418
**cg09706833**	21	46824938	COL18A1	0.0441286	0.302312610626543	0.0627142914522418
**cg11865519**	15	101163830	ASB7;ASB7	-0.0265727	-0.0422017769457813	0.0627142914522418
**cg08989903**	1	24952794		-0.104994	-0.206386468371954	0.0627142914522418
**cg20280386**	10	99080417	FRAT1;FRAT1	0.021886	0.870816129646476	0.0627142914522418
**cg12639796**	1	32479246	KHDRBS1;KHDRBS1;KHDRBS1;KHDRBS1	0.0139864	1.40283964725664	0.0627142914522418
**cg00603717**	13	46039090	COG3;COG3	0.0292975	0.579886927891005	0.0627142914522418
**cg11359770**	2	26304137	RAB10	-0.0735431	-0.129190663649272	0.0628258293549384
**cg02474542**	11	72300891	PDE2A;PDE2A;PDE2A;PDE2A	0.008757	0.457541444812571	0.0629014080171654
**cg19742870**	9	71860887	TJP2;TJP2;TJP2;TJP2;TJP2;TJP2	-0.0857317	-0.15135920849825	0.0629014080171654
**cg16201674**	13	95364586	SOX21	0.0403307	0.548720854448031	0.0629014080171654
**cg18457685**	6	29760659	HCG4	0.0390255	0.976994982838922	0.0629014080171654
**cg01879257**	20	40513291		-0.0268267	-0.0409841035497993	0.0629014080171654
**cg05804856**	10	127715083	ADAM12;ADAM12	-0.0816788	-0.202533901620495	0.0629014080171654
**cg13192053**	8	60062434		-0.136257	-0.367375421274856	0.0629014080171654
**cg12090014**	1	182462043	RGSL1	0.0274402	0.0434373481496855	0.0629014080171654
**cg23053573**	1	26662567	AIM1L	0.00761081	0.337838405883455	0.0629014080171654
**cg03216991**	17	2415620	METT10D	0.0701359	0.272857633244329	0.0629014080171654
**cg23415810**	12	53920129	ATF7;ATF7;ATF7	-0.0772261	-0.129877449510882	0.0629014080171654
**cg03363567**	17	57642929	DHX40;DHX40;DHX40;DHX40	-0.00178017	-0.33543368966639	0.0629014080171654
**cg05348421**	17	1359962	CRK;CRK	0.0838292	0.484686515224238	0.0629160702151938
**cg19889264**	15	75018700	CYP1A1	0.0266173	0.576009779255354	0.0629160702151938
**cg03537567**	4	25375829		-0.0716623	-0.136608829718778	0.0629160702151938
**cg06318239**	2	234921750	TRPM8	-0.0148427	-0.0221963936969107	0.0629160702151938
**cg00279778**	22	45102452	PRR5;PRR5;PRR5;PRR5-ARHGAP8;PRR5;PRR5	-0.0672269	-0.140961945688283	0.0629160702151938
**cg15701794**	10	77156421		0.0213439	0.536986648997934	0.0629160702151938
**cg06674436**	5	1278864	TERT;TERT	0.00172153	0.00250492027090461	0.0633352380262148
**cg05587970**	11	61187174	CPSF7;CPSF7;CPSF7	-0.0690431	-0.124864169918003	0.0637961556823956
**cg09507567**	10	124027408		-0.0711497	-0.180372421226922	0.0637961556823956
**cg05082095**	1	155099843	EFNA1;EFNA1	0.026794	0.7460762723568	0.0637961556823956
**cg08035151**	6	31772251	2,00 LSM	-0.064149	-0.105479164502134	0.0637961556823956
**cg09125080**	5	71687192		-0.05139	-0.0852100695082129	0.0637961556823956
**cg21215416**	11	77532152	AAMDC;RSF1	0.0220654	0.659151956413864	0.0637961556823956
**cg17357614**	10	26965487		-0.0442607	-0.071754973240842	0.0637961556823956
**cg04120002**	20	51712963	TSHZ2	-0.0994753	-0.191898082205134	0.0637961556823956
**cg01609171**	16	127997	MPG;MPG;MPG	0.00904743	0.622360647962198	0.0637961556823956
**cg08270825**	9	125561317	OR1K1	-0.117115	-0.264893309038985	0.0637961556823956
**cg20573956**	3	112957882	BOC;BOC;BOC	-0.0808567	-0.157603228557427	0.0637961556823956
**cg07855129**	1	90287374	LRRC8D;LRRC8D	0.017677	0.421986461379683	0.0637961556823956
**cg08452838**	10	34451339	PARD3;PARD3;PARD3;PARD3;PARD3;PARD3;PARD3;PARD3	-0.038357	-0.0640829631844589	0.0637961556823956
**cg17795164**	2	153032738	STAM2	0.00704447	0.507205506669514	0.0637961556823956
**cg19730147**	8	124253706	C8orf76	0.0184468	0.545908073181726	0.0637961556823956
**cg22851420**	1	40149650	HPCAL4	0.0500983	0.673074516423237	0.0637961556823956
**cg04233747**	5	145214971	PRELID2;PRELID2;PRELID2	0.0348826	0.748688605080674	0.0637961556823956
**cg11638071**	17	56405967	BZRAP1;BZRAP1;BZRAP1;BZRAP1	0.0066043	0.444020224669512	0.0637961556823956

**cg24866077**	1	226119646		0.015917	0.0251540674342917	0.0637961556823956
**cg12196367**	3	183892577	AP2M1;AP2M1	0.0401013	0.553524488987423	0.0637961556823956
**cg26214765**	6	166797748	BRP44L	-0.0965817	-0.213145954695888	0.0637961556823956
**cg05561955**	22	24951987	GUCD1;GUCD1;GUCD1;GUCD1;GUCD1;SNRPD3;SNRPD3;GUCD1;GUCD1;GUCD1;GU	0.0193195	0.919508156076826	0.0637961556823956
**cg02048065**	10	60757288		0.0112935	0.0170105216199023	0.0637961556823956
**cg16861047**	1	116518978	SLC22A15	0.0172981	0.639477504263023	0.0637961556823956
**cg12108527**	1	43233151	LEPRE1;C1orf50;LEPRE1	0.0177094	0.625530609227367	0.0637961556823956
**cg09726328**	1	63833127	ALG6	0.0276473	0.833799017404371	0.0637961556823956
**cg13390224**	22	38287268		-0.0586187	-0.106276717844442	0.0637961556823956
**cg07669147**	19	36134735	ETV2	0.0289722	0.587817905029017	0.0637961556823956
**cg21654404**	12	118814431	SUDS3;SUDS3	0.0460932	0.528392729913499	0.0637961556823956
**cg00729314**	1	47082382	MOB3C;MOB3C	0.0180434	0.523822059639172	0.0637961556823956
**cg14174336**	16	67208654	NOL3	0.0433093	0.48672843945862	0.0637961556823956
**cg25924827**	3	155860278	KCNAB1;KCNAB1	-0.0940914	-0.167544913256282	0.0637961556823956
**cg27343380**	7	158617671	ESYT2	-0.0127735	-0.0191235495833764	0.0637961556823956
**cg00452522**	1	246887291	SCCPDH	0.0128574	0.781340721089639	0.0637961556823956
**cg17858851**	16	67427618	TPPP3;TPPP3	0.0306518	0.8191669176536	0.0637961556823956
**cg19112344**	20	23427172		-0.0712109	-0.151189988836518	0.0637961556823956
**cg16632433**	5	180649677	TRIM41;TRIM41	0.0156779	0.644167248327708	0.0637961556823956
**cg00321115**	13	28942568	FLT1;FLT1	-0.0953072	-0.19355536768909	0.0637961556823956
**cg14962383**	17	65318223		-0.0436988	-0.0822523543677304	0.0637961556823956
**cg05697637**	4	42659358	ATP8A1;ATP8A1	0.0197184	0.815731726188963	0.0637961556823956
**cg27075724**	21	18985387	BTG3;BTG3	0.00596025	0.450099854846573	0.0637961556823956
**cg11372563**	19	27737627		-0.0485438	-0.0821318595530706	0.0637961556823956
**cg02820613**	16	30538074	ZNF768	0.0160007	0.508407646773399	0.0637961556823956
**cg16241861**	14	103995231	TRMT61A	0.0463358	0.667323041549783	0.0637961556823956
**cg12995897**	2	196372688		-0.0590321	-0.0987282683806388	0.0637961556823956
**cg23254908**	16	50279922		0.0255645	0.429127103639789	0.0637961556823956
**cg09357935**	11	20626264	SLC6A5	0.015449	0.442559945175715	0.0637961556823956
**cg09208919**	9	326599	DOCK8;DOCK8;DOCK8	-0.072743	-0.128868503950133	0.0637961556823956
**cg15277305**	7	105042987		-0.0192365	-0.0289947702554326	0.0637961556823956
**cg04686763**	11	64655583		0.0360285	0.51898260935587	0.0637961556823956
**cg13518530**	19	13030150	SYCE2	0.0512127	1.08073639078491	0.0637961556823956
**cg06450908**	2	24433251	ITSN2;ITSN2	-0.0410518	-0.068719843138923	0.0637961556823956
**cg03947203**	11	118987153	C2CD2L	-0.0158315	-0.0236624908332038	0.0637961556823956
**cg04288257**	7	157361639	PTPRN2;PTPRN2;PTPRN2;PTPRN2;PTPRN2;PTPRN2;PTPRN2;PTPRN2;PTPRN2;PTPR	0.00361362	0.243201709897544	0.0637961556823956
**cg13091133**	4	42190340		-0.0716296	-0.132106668240776	0.0637961556823956
**cg12867853**	22	44351301	SAMM50;SAMM50	0.0195848	0.517815064697836	0.0637961556823956
**cg22048274**	6	31853428	EHMT2;EHMT2	-0.0664483	-0.117129215534112	0.0637961556823956
**cg20008357**	6	32145745	RNF5P1;RNF5;AGPAT1	0.025511	0.461747796382273	0.0637961556823956
**cg07046142**	8	72628609		-0.0568216	-0.109037936036972	0.0637961556823956
**cg21311736**	6	16126437		-0.0529183	-0.111502721254653	0.0637961556823956
**cg07261133**	14	23923449		-0.0991035	-0.196971241835006	0.0637961556823956
**cg07049977**	2	37458536	CEBPZ;C2orf56;C2orf56	0.0280239	0.756550558992576	0.0637961556823956
**cg06293195**	22	36878654	TXN2	-0.0777068	-0.146586515911594	0.0637961556823956
**cg15778457**	7	155165295		0.013946	0.535854708723183	0.0637961556823956
**cg10335659**	2	99757356	C2orf15;TSGA10;TSGA10	0.0202171	0.816801549327057	0.0637961556823956
**cg26536189**	1	13840494		0.0115701	0.588921448929265	0.0637961556823956
**cg22844531**	3	47422418	PTPN23;PTPN23	0.0205334	0.783661372253816	0.0637961556823956
**cg17308959**	3	15643111	HACL1;HACL1;BTD	0.0106341	0.524130177141522	0.0637961556823956
**cg24770927**	14	24911599	SDR39U1	0.0072899	0.459594973968431	0.0637961556823956
**cg06746074**	3	87276268	CHMP2B	0.0152325	0.39356162405823	0.0637961556823956
**cg20744217**	11	67211017	CORO1B;CORO1B	0.0753031	0.47078920861115	0.0637961556823956
**cg08580214**	9	127624539	RPL35	0.0154877	0.870322045106702	0.0638028490249762
**cg12523403**	19	12779890	MORG1;MORG1;C19orf56;MORG1	0.0363288	0.957083925978011	0.0638028490249762
**cg20925075**	11	66515238	C11orf80;C11orf80	-0.0637313	-0.118433048838103	0.0638028490249762
**cg14696775**	14	74497016	CCDC176	-0.142991	-0.333323922151667	0.0638028490249762
**cg22269416**	6	32068240	TNXB	-0.0366199	-0.0588223048527268	0.0638028490249762
**cg00203463**	9	140150331	COBRA1	0.0377323	1.00595288059274	0.0638028490249762
**cg10387007**	14	77248049	VASH1	-0.0511015	-0.0965725556256452	0.0638028490249762
**cg07230192**	7	33018114	FKBP9;FKBP9;FKBP9	-0.0231699	-0.0358299950049635	0.0638028490249762
**cg26077214**	1	84258662	LOC101927587	-0.0807604	-0.156898761401605	0.0638028490249762
**cg13492103**	1	75596827	LHX8	0.0105721	0.488496729611718	0.0638028490249762
**cg06525537**	9	112970566	C9orf152	-0.0272569	-0.0425771277081747	0.0638028490249762
**cg15339796**	20	373038	TRIB3;TRIB3;TRIB3;TRIB3;TRIB3;TRIB3	-0.0948107	-0.178059785088695	0.0638028490249762
**cg16684937**	11	695322	TMEM80;TMEM80;TMEM80;TMEM80;DEAF1;DEAF1;DEAF1;DEAF1	0.0175361	0.858499628018986	0.0638028490249762
**cg14815005**	22	22222162	MAPK1;MAPK1	0.0239576	0.915395181510354	0.0638028490249762
**cg05921832**	16	74819124		-0.0701714	-0.150179667655342	0.0638028490249762
**cg14337526**	13	98086635	RAP2A;RAP2A	0.0560143	0.523666520076877	0.0638028490249762
**cg26427417**	12	124192819	TCTN2;TCTN2	-0.0517775	-0.10549462394258	0.0638198881746216
**cg18605066**	19	3029158	TLE2;TLE2;TLE2;TLE2;TLE2;TLE2	0.0407905	0.645558075381615	0.0638198881746216
**cg19838074**	2	242149865	ANO7	-0.0187589	-0.0283301875525582	0.0638198881746216
**cg06931542**	6	149867387	PPIL4	0.0171001	0.953295328718306	0.0638198881746216
**cg05363350**	1	46727745	RAD54L;RAD54L	-0.062305	-0.12239506919	0.0638198881746216
**cg16903437**	5	138556295		-0.0795973	-0.151904273589053	0.0638198881746216
**cg08864829**	12	49050903	SNORA2A;C12orf41	-0.0875391	-0.197732693877263	0.0638198881746216
**cg12629012**	4	57803754		-0.0506366	-0.0901517811962359	0.0638198881746216
**cg06530991**	1	202898906		-0.0599005	-0.121162344941632	0.0638198881746216
**cg22754654**	19	14197848	C19orf67	-0.0833777	-0.142131217112275	0.0638198881746216
**cg07970433**	10	64582099		-0.014772	-0.0222366748335053	0.0638198881746216
**cg05319477**	5	56391430		-0.0485892	-0.0814039753527676	0.0638198881746216
**cg19775406**	17	5390252	MIS12;MIS12;DERL2	0.0103292	0.513940955991078	0.0638198881746216

**cg07616922**	11	34049237		-0.0240793	-0.0369175763994513	0.0638198881746216
**cg20774490**	9	132565255	TOR1B	0.0261634	0.660631881947593	0.0638198881746216
**cg21547976**	19	1206451	STK11;STK11	0.0156429	0.784196317488346	0.0638198881746216
**cg03590216**	20	5931285	MCM8;MCM8;TRMT6	0.0220232	0.568961010098438	0.0638198881746216
**cg07370464**	17	43394456	MAP3K14	0.0313162	0.53398434761725	0.0638198881746216
**cg01643110**	3	47619726	CSPG5;CSPG5;CSPG5;CSPG5;CSPG5	0.013995	0.528559188398338	0.0638198881746216
**cg25540078**	4	144102007		-0.0628506	-0.121186448802502	0.0638198881746216
**cg15332162**	1	224370698	DEGS1	0.041956	0.814264324343054	0.0638198881746216
**cg27346562**	17	46166752	CBX1;CBX1	-0.0264477	-0.040943996888782	0.0638198881746216
**cg13765002**	6	144385134	PLAGL1;PLAGL1;PLAGL1;PLAGL1;PLAGL1;PLAGL1;PLAGL1;PLAGL1;PLAGL1	0.0111133	0.746521041381334	0.0638198881746216
**cg12032191**	1	46152434	TMEM69;GPBP1L1	0.0081279	0.551394224156435	0.0638198881746216
**cg08207256**	19	10828848	DNM2;DNM2;DNM2;DNM2;DNM2;DNM2;DNM2;MIR638;DNM2	0.0278363	0.523450565158612	0.0638198881746216
**cg13812249**	9	71999962	FAM189A2;FAM189A2	-0.0761952	-0.171135172425229	0.0638198881746216
**cg24082320**	19	2328980	SPPL2B;SPPL2B;LSM7	0.0101392	0.429478684759344	0.0638198881746216
**cg17588904**	1	114493165	HIPK1;HIPK1;HIPK1	-0.130798	-0.287251078744532	0.0638198881746216
**cg25022687**	10	118032987	GFRA1;GFRA1;GFRA1;GFRA1	0.0105673	0.460248673509923	0.0638198881746216
**cg22850304**	1	39325639	RRAGC	0.01111	0.62976248056508	0.0638198881746216
**cg11562147**	6	36303570	C6orf222	-0.0273782	-0.0443737197563988	0.0638198881746216
**cg15346191**	2	242743588	GAL3ST2	0.028339	0.866763263628455	0.0638198881746216
**cg04371440**	12	112819495		0.00842127	0.535377513230685	0.0638198881746216
**cg04921619**	12	7000534		0.0118504	0.460782441530269	0.0638198881746216
**cg18264753**	6	138188625	TNFAIP3;TNFAIP3	0.0201452	0.800150303503658	0.0638198881746216
**cg04253876**	2	30453758	LBH	0.025793	0.670122789077451	0.0638198881746216
**cg06837242**	1	19578761	MRTO4;KIAA0090	0.0307467	0.741592275720288	0.0638198881746216
**cg22361604**	1	61435148		-0.0756368	-0.141450013624474	0.0638198881746216
**cg27485605**	12	96430600	LTA4H;LTA4H;LTA4H	-0.0980579	-0.21218642806632	0.0638198881746216
**cg12055421**	2	74685521	WBP1	0.00397289	0.365814775785985	0.0638198881746216
**cg20725035**	3	148709344	GYG1	0.054214	0.608839905086403	0.0638198881746216
**cg13599271**	3	58291905	RPP14;RPP14	0.0299244	0.663075202016128	0.0638198881746216
**cg14939446**	16	2571015	AMDHD2;AMDHD2	0.00644167	0.487185528240149	0.0638198881746216
**cg02892271**	8	42120927	LOC101929897	-0.0131247	-0.0196934478763763	0.0638198881746216
**cg14212045**	5	142179386	ARHGAP26;ARHGAP26	-0.0599093	-0.103425554179631	0.0638198881746216
**cg20326786**	1	176912031	ASTN1;ASTN1	-0.10888	-0.250576004039036	0.0638198881746216
**cg26320000**	2	129902340		-0.0562036	-0.0931939328403634	0.0638198881746216
**cg03980304**	16	27325000	IL4R;IL4R	0.00835801	0.593882029317171	0.0638198881746216
**cg24860356**	20	33624140	TRPC4AP;TRPC4AP	-0.0637421	-0.111949100854468	0.0638198881746216
**cg02254698**	7	87508528	DBF4	-0.0580303	-0.0924338911038442	0.0638198881746216
**cg14665812**	5	170880235	FGF18	-0.0243247	-0.0392281803142874	0.0638198881746216
**cg14559709**	1	234785604	LOC101927787	-0.0500188	-0.0788522237786281	0.0638198881746216
**cg18374625**	19	50004152	MIR150	0.0169717	0.502447375734477	0.0638198881746216
**cg06194885**	3	138150379		-0.0646466	-0.112536282112282	0.0638198881746216
**cg08314795**	20	42450922		-0.0758583	-0.138081153906915	0.0638486631192083
**cg24078554**	1	120182239	ZNF697	-0.0617158	-0.109803842766001	0.0638486631192083
**cg05719672**	15	91446576	MAN2A2	0.0253343	0.734221691123991	0.0640482042019922
**cg00113951**	12	121078090	CABP1	0.00892935	0.33861777053317	0.0641166877450315
**cg05890550**	17	2699706	RAP1GAP2;RAP1GAP2	0.00999376	0.664322518307905	0.0641203665745313
**cg13950603**	19	7795619	CLEC4G	0.019434	0.453922884789687	0.0642851491443453
**cg20947164**	10	124105011		-0.0198983	-0.029914288114485	0.0645256869037719
**cg21341492**	1	9836431	CLSTN1;CLSTN1;CLSTN1	-0.0905523	-0.18078546915667	0.0646954670925009
**cg16438841**	12	132149724		-0.0271332	-0.0409579277479921	0.0648343760594094
**cg00673963**	1	100731856	RTCD1;RTCD1;RTCD1;RTCD1	0.00644696	0.443979869694123	0.0648343760594094
**cg07636362**	8	79717261	IL7;IL7	0.0125302	0.610083861494778	0.0648343760594094
**cg08517455**	2	99439997	C2orf55	-0.0192496	-0.0297580869171712	0.0648343760594094
**cg25867545**	3	182880776	LAMP3	0.0424769	0.455634103906536	0.0648343760594094
**cg08531746**	13	113512905	ATP11A;ATP11A	-0.0487321	-0.0778102944545666	0.0648343760594094
**cg11732949**	10	7993238	TAF3	-0.0639877	-0.106434900839473	0.0648343760594094
**cg07233389**	9	112085534		-0.0601974	-0.101951486072205	0.0648343760594094
**cg10059324**	1	7884824	PER3	-0.0697186	-0.138390304825217	0.0648343760594094
**cg13872879**	19	51071324	LRRC4B	0.0253191	0.642048703415259	0.0648343760594094
**cg14049549**	12	132156010		-0.0670614	-0.118922055498333	0.0648343760594094
**cg06984528**	2	82062958		-0.0818923	-0.16151215200971	0.0648343760594094
**cg02063360**	14	105452560	C14orf79	0.0166429	0.495192704775618	0.0648343760594094
**cg27568321**	4	113152709	AP1AR;AP1AR	0.0315105	0.938460101734212	0.0648343760594094
**cg14925091**	13	113721521	MCF2L;MCF2L	-0.0497991	-0.0907748527860833	0.0648343760594094
**cg23016347**	10	75170966	ANXA7;ANXA7	-0.0572879	-0.116297149076041	0.0648343760594094
**cg07500799**	7	127650747	SND1	0.0142742	0.0218323750887338	0.0648343760594094
**cg17102910**	7	1126249	GPER;C7orf50;C7orf50;GPER;C7orf50;GPER	-0.0499642	-0.110831642296786	0.0648343760594094
**cg01167366**	10	12742170	CAMK1D;CAMK1D	-0.0349313	-0.0551432246967248	0.0648343760594094
**cg16494260**	14	55967277		-0.0335065	-0.0531230619947757	0.0648343760594094
**cg12064008**	1	32827730	LOC100128071;LOC100128071;TSSK3	0.0124122	0.836972608092615	0.0648343760594094
**cg01918066**	20	43159964	PKIG;PKIG;PKIG;PKIG	-0.0611691	-0.103382662917855	0.0648343760594094
**cg23300001**	10	134628152		-0.0279493	-0.0453242943940102	0.0648343760594094
**cg16693127**	10	62493129	ANK3;ANK3	0.0302836	0.642273627317725	0.0648343760594094
**cg10194899**	6	43248849	TTBK1	-0.116097	-0.223700208901231	0.0648343760594094
**cg21647833**	17	57184330	TRIM37;TRIM37	0.00908776	0.623015208407843	0.0648343760594094
**cg12142685**	10	12391596	CAMK1D;CAMK1D;CAMK1D;CAMK1D	0.0488211	0.341559374663839	0.0648343760594094
**cg21396456**	3	43021214	FAM198A	0.0134607	0.461375266296268	0.0648343760594094
**cg18780740**	6	80516964	C6orf7	-0.0397678	-0.0648091976386578	0.0651479690567066
**cg24467825**	6	30302667	TRIM39;TRIM39	-0.0896621	-0.175729408630665	0.0651577145239516
**cg01169772**	5	41904236	C5orf51	0.00447064	0.355825617936927	0.0651577145239516
**cg00181527**	9	135754163	AK8;AK8;C9orf9	0.0140459	0.643102941836349	0.0651577145239516
**cg08101859**	4	56280173	TMEM165;TMEM165	-0.0487716	-0.0816889367005565	0.0651577145239516

**cg03770187**	1	247979766	OR14A16	-0.0698373	-0.165570996884996	0.0651577145239516
**cg14264457**	18	52679522		-0.0550925	-0.0927886757936148	0.0651577145239516
**cg04947856**	2	239112553	ILKAP	0.00562793	0.48718065177299	0.0651577145239516
**cg21576095**	17	72586279	CD300LD;C17orf77;C17orf77	-0.0966843	-0.206797794983897	0.0651577145239516
**cg16174680**	8	41504078	NKX6-3	0.00374581	0.350879218451131	0.0651577145239516
**cg19744051**	5	55294284		-0.0952469	-0.185776889023513	0.0651577145239516
**cg04517323**	8	98788873	LAPTM4B	0.0336988	0.494811858905731	0.0651577145239516
**cg17748061**	4	11368007		-0.0123674	-0.0189120813142202	0.0651577145239516
**cg06002947**	16	28936713	RABEP2	0.0256892	0.737068337710918	0.0651577145239516
**cg08649501**	3	41997107	ULK4	-0.0196755	-0.0303475405577021	0.0651577145239516
**cg23702073**	1	36621802	MAP7D1;MAP7D1;MAP7D1;MAP7D1;MAP7D1;MAP7D1;TRAPPC3	0.0390835	0.397833489777629	0.0651577145239516
**cg17480615**	3	3137289	IL5RA;IL5RA;IL5RA;IL5RA;IL5RA;IL5RA;IL5RA	-0.0325451	-0.0511275196506931	0.0651577145239516
**cg16516670**	8	71382800	LOC101926892	-0.0754466	-0.125278216078251	0.0651577145239516
**cg16162324**	12	104852047	CHST11	0.00614505	0.439920938918579	0.0651577145239516
**cg07232475**	15	44069664	ELL3	0.00610087	0.526711229533145	0.0652094004001671
**cg22486192**	16	29848547	MVP;MVP;MVP;MVP	-0.0785948	-0.156417883502449	0.0652094004001671
**cg00047843**	12	83321412	TMTC2	-0.0881619	-0.180619753075834	0.0652094004001671
**cg13070817**	2	204861567		-0.0486798	-0.100278602399761	0.0652094004001671
**cg13148818**	2	85956418		0.0168652	0.178984893768791	0.0652094004001671
**cg25150333**	12	132195268	SFRS8	0.0197311	0.660939984645658	0.0652094004001671
**cg24799451**	11	134093757	NCAPD3;VPS26B	0.00546344	0.481844426519193	0.0652094004001671
**cg17103081**	4	22514419	GPR125	-0.0663631	-0.111430554091274	0.0652094004001671
**cg05814555**	1	62902599	USP1;USP1;USP1;USP1	0.0121553	0.463821415907693	0.0652094004001671
**cg15573783**	19	45927056	ERCC1;ERCC1;ERCC1;ERCC1;ERCC1	0.0277155	0.258304783477381	0.0652094004001671
**cg07143898**	10	98945300	SLIT1	0.0190158	0.737166264855784	0.0652094004001671
**cg17985418**	17	66031785	KPNA2	0.0206371	1.03839687079326	0.0652094004001671
**cg03450784**	1	228358760	IBA57	0.00909407	0.0135440997777652	0.0652094004001671
**cg06086148**	3	128399777		0.0172908	0.966117951213305	0.0652094004001671
**cg20719132**	19	56028425	SSC5D	0.016079	0.449739076101465	0.0652094004001671
**cg07844072**	19	1372272	MUM1;MUM1	-0.0563869	-0.0907049266358928	0.0652094004001671
**cg08697280**	20	36322294	CTNNBL1;CTNNBL1	0.0177721	0.535063766414878	0.0652094004001671
**cg12532831**	4	144230446		-0.0602921	-0.106037555472037	0.0652094004001671
**cg24920997**	3	192109830	FGF12;FGF12	-0.0860804	-0.20076879733906	0.0652094004001671
**cg19763809**	2	98703475	VWA3B	0.0298374	0.402075561637214	0.0652094004001671
**cg24681048**	20	25604516	ZNF337-AS1;ZNF337-AS1;ZNF337-AS1;NANP	0.0273047	0.622344363243982	0.0652094004001671
**cg00210473**	6	41341942		0.0125438	0.480720585073841	0.0652094004001671
**cg09554951**	11	2890725	KCNQ1DN	0.0290764	0.559266416524886	0.0652094004001671
**cg14090374**	1	85667233	SYDE2	0.0132492	0.472712373975146	0.0652094004001671
**cg14541281**	16	22321484	POLR3E;POLR3E;POLR3E;POLR3E;POLR3E;POLR3E	-0.0472542	-0.0796460743371714	0.0652094004001671
**cg07475973**	22	42306951		0.00557833	0.395702471226999	0.0652094004001671
**cg02973875**	3	126196376		-0.0813028	-0.141792219498226	0.0652094004001671
**cg02112893**	17	29718165	RAB11FIP4	0.0284344	0.465956358816795	0.0652094004001671
**cg15744637**	11	809911	RPLP2	0.0366198	0.903126909380253	0.0652094004001671
**ch.1.198578402R**	1	200311779		0.00489731	0.398559779138434	0.0652094004001671
**cg15700924**	7	139244174		-0.0210618	-0.0321406574225035	0.0652094004001671
**cg17796333**	6	52511342		-0.0149464	-0.0224596424265915	0.0652094004001671
**cg11621243**	20	20200773	CFAP61	-0.0741439	-0.128595989600617	0.0652094004001671
**cg20078939**	11	33060711	TCP11L1;TCP11L1	0.0135202	0.699661756951133	0.0652094004001671
**cg25444556**	5	153990114		0.00564994	0.508578851769046	0.0652094004001671
**cg24172570**	7	27561178		-0.0469667	-0.0817263399554602	0.0652094004001671
**cg08823985**	6	137366545	IL20RA	-0.0908203	-0.266230545677179	0.0654026339937296
**cg11973877**	1	52195423	OSBPL9;OSBPL9;OSBPL9;OSBPL9;OSBPL9;OSBPL9;OSBPL9	0.00926232	0.547591395420158	0.0655503107967182
**cg09826550**	4	47839283	CORIN	0.0102061	0.58298979949485	0.0655503107967182
**cg04108296**	1	167691246	MPZL1;MPZL1;MPZL1;MPZL1;MPZL1;MPZL1	0.0159242	0.507364748287883	0.0655936653150758
**cg02120513**	5	36722752		-0.0414744	-0.0721708979541832	0.0657883333179143
**cg05174943**	10	134513773	INPP5A	-0.0190437	-0.0289772694704869	0.0657883333179143
**cg18092614**	17	40729792	PSMC3IP;PSMC3IP;PSMC3IP;PSMC3IP;PSMC3IP;PSMC3IP;PSMC3IP;PSMC3IP;PSMC	0.0361091	0.611997648958867	0.0657883333179143
**cg04231929**	9	93556220		-0.0896076	-0.209388460128029	0.0659412720310244
**cg20297940**	1	14746121		-0.0503674	-0.0866316016232431	0.0659412720310244
**cg18785467**	6	88412083	NCRNA00120;AKIRIN2	0.0392611	0.544835372355626	0.0659412720310244
**cg01337508**	17	40540572	STAT3;STAT3;STAT3	0.0107816	0.480424235742401	0.0659412720310244
**cg25320496**	2	130876007	POTEF	-0.107667	-0.264185861045224	0.0659412720310244
**cg08314899**	12	102045493	MYBPC1;MYBPC1;MYBPC1;MYBPC1;MYBPC1;MYBPC1;MYBPC1;MYBPC1;MYBPC1;M	-0.0585806	-0.101635820641524	0.0659412720310244
**cg15794350**	12	56395187	SUOX;SUOX;SUOX	-0.0476823	-0.0843587082856408	0.0659412720310244
**cg27452651**	22	50312357	ALG12;CRELD2;CRELD2;CRELD2;CRELD2	0.017997	0.547055439471786	0.0659412720310244
**cg17790113**	3	125652101	ALG1L	-0.0189499	-0.0290823711366943	0.0659412720310244
**cg01438737**	20	42086396	SFRS6	0.0278108	1.14427033891715	0.0659412720310244
**cg14797322**	12	58165899	FAM119B;METTL1;METTL1;METTL1;METTL1;FAM119B	0.0154812	1.28454544363917	0.0659412720310244
**cg24595041**	2	9761870	YWHAQ	-0.0586556	-0.121974992885069	0.0659412720310244
**cg04728296**	4	166414646	CPE	-0.0333519	-0.367200785486134	0.0659412720310244
**cg20469575**	4	169122189		-0.113616	-0.215307713282418	0.0659412720310244
**cg24126592**	11	46389363	DGKZ;DGKZ;DGKZ;DGKZ	0.0527542	0.624237417326482	0.0659412720310244
**cg06466360**	18	749231	YES1	-0.0523583	-0.0927575612330321	0.0659412720310244
**cg16976547**	15	91433530	FES;FES;FES;FES	-0.0353304	-0.0561871104437334	0.0659412720310244
**cg25974903**	1	65211057	RAVER2	0.0112255	0.479746537373149	0.0659412720310244
**cg11874143**	3	138065751	MRAS	-0.0665966	-0.158163835077923	0.0660260871819099
**cg26894684**	13	52378339	DHRS12;DHRS12	0.014579	0.800516642182949	0.0660431357896831
**cg05158631**	6	43862455	LINC01512	-0.0256036	-0.0405336888650117	0.0660431357896831
**cg26471385**	21	43299747	PRDM15;PRDM15;PRDM15;PRDM15;PRDM15	0.0144673	0.610126967879684	0.0660431357896831
**cg19034733**	8	130096484		-0.0371837	-0.0601080869844593	0.0660431357896831
**cg14033917**	1	32609664	KPNA6	-0.0773353	-0.143263486343489	0.0661490926086976
**cg23664545**	17	74070257	GALR2	0.0425443	0.504219587703997	0.0661490926086976

**cg25992738**	7	152589017		-0.078651	-0.163853664306983	0.0661490926086976
**cg22120988**	12	65515350	WIF1	0.0259843	0.835081703773288	0.0661490926086976
**cg25348336**	2	39003850	GEMIN6	-0.0634628	-0.139071793271966	0.0661490926086976
**cg02181968**	12	39836347	KIF21A	0.0209423	0.4259353237836	0.0661490926086976
**cg02402209**	4	152249812		-0.0938889	-0.178857127323233	0.0661490926086976
**cg07691152**	12	103355123		0.0486774	0.376808370987826	0.0661490926086976
**cg23454177**	16	67970396	PSMB10	0.0141861	0.89934394879734	0.0661490926086976
**cg21753290**	20	37376903	ACTR5	0.0092375	0.746942385692137	0.0661868360705664
**cg11644394**	7	144148615		-0.118111	-0.244881827795912	0.0661868360705664
**cg27484621**	18	6788200		-0.019483	-0.0295778373718991	0.0661868360705664
**cg03580256**	1	78149279	ZZZ3;ZZZ3	0.0220957	0.381684666113491	0.0661868360705664
**cg11555752**	3	187925787	LPP	-0.0146395	-0.0221426921486004	0.0661868360705664
**cg09453280**	14	35403910		-0.0538564	-0.0856147183223715	0.0661868360705664
**cg05702448**	1	167905194	DCAF6;BRP44;BRP44;BRP44;DCAF6;BRP44	0.0417355	1.01364336907873	0.0661868360705664
**cg13027404**	15	53095984		0.0245252	0.784194577061812	0.0661868360705664
**cg01334211**	8	141729014	PTK2;PTK2;PTK2	-0.0774735	-0.17907950513204	0.0663898045527364
**cg13916146**	15	63248230		-0.0583237	-0.100317834464066	0.0663898045527364
**cg25202793**	8	129829827		0.0743718	0.134574371616318	0.0663898045527364
**cg17087373**	10	82212706	TSPAN14;TSPAN14	-0.0738093	-0.134818511083352	0.0664628725553255
**cg25192948**	1	32479490	KHDRBS1;KHDRBS1	0.0280339	0.567957299019665	0.0664628725553255
**cg17891820**	4	55524400	KIT;KIT	0.0102474	0.545270756457071	0.0664628725553255
**cg08216131**	20	2637503	NOP56;SNORD57;NOP56	0.0134433	0.0204176034509982	0.0664628725553255
**cg21302194**	4	155412348	DCHS2;DCHS2	0.0349949	0.707996772204644	0.0664628725553255
**cg03195060**	15	74334206	PML;PML;PML;PML	0.00838194	0.0124230971012386	0.0664628725553255
**cg18153578**	19	38893604	FAM98C	0.00929316	0.52173210577385	0.0664628725553255
**cg27052090**	14	54939059		-0.0621383	-0.10661152516315	0.0664628725553255
**cg03710333**	4	1722958	TMEM129;TACC3;TMEM129;TMEM129;TMEM129	0.00890594	0.438634761534602	0.0664838944298731
**cg16855870**	2	886764		-0.0367075	-0.0581332705383853	0.0666128737106594
**cg21646199**	1	116381654	NHLH2;NHLH2	0.0306331	0.812722276007753	0.0666652379364637
**cg25342861**	17	45177598		0.0196743	0.588557896110531	0.0666655228457385
**cg03922588**	11	62473871	GNG3;BSCL2;BSCL2;BSCL2;HNRNPUL2-BSCL2;BSCL2	0.0366306	0.667536985579221	0.0666655228457385
**cg25506386**	3	63849756	THOC7;ATXN7;ATXN7	0.0274603	0.741032989353084	0.0666655228457385
**cg01030973**	1	2574695		-0.00794304	-0.367825768432081	0.0666655228457385
**cg05415131**	11	58940866	DTX4	0.0161662	0.536945532878599	0.0666655228457385
**cg01624301**	9	128469583	MAPKAP1;MAPKAP1;MAPKAP1;MAPKAP1;MAPKAP1;MAPKAP1	0.0135492	0.41260120602019	0.0666655228457385
**cg01579535**	12	54447292	HOXC4;HOXC4	0.0349119	0.655390081150558	0.0666655228457385
**cg24695441**	1	16751686	SPATA21	-0.0720143	-0.144202488924831	0.0666655228457385
**cg03458099**	2	223532694		-0.0508228	-0.0836523324846008	0.0666655228457385
**cg05110693**	16	71928225	IST1;IST1;IST1;IST1;IST1;IST1	0.0296421	0.532394403591275	0.0666655228457385
**cg25531497**	10	25465219	GPR158;LOC100128811	0.0151848	0.548247224816937	0.0666655228457385
**cg04293275**	10	9710767		-0.0625361	-0.105285466599518	0.0667384182260743
**cg23126234**	2	229549575		-0.025976	-0.0410429808465107	0.0667384182260743
**cg26261342**	12	56512029	ZC3H10;ZC3H10	0.00791586	0.405898968950705	0.0667401829846008
**cg21144673**	22	42017783	PPPDE2;XRCC6	0.0109167	0.682870904583575	0.0667401829846008
**cg04258637**	8	100907546	COX6C	-0.0651819	-0.18936742821514	0.0667401829846008
**cg02654704**	16	74685448	RFWD3	-0.0645434	-0.120823653883946	0.0667401829846008
**cg08677672**	12	124891822	NCOR2;NCOR2;NCOR2	0.0146458	0.0224811258718943	0.0667401829846008
**cg19656847**	11	75996257		-0.0628894	-0.109192569856681	0.0667401829846008
**cg11351687**	6	170863423	PSMB1;TBP	0.0295069	0.762155123697487	0.0667401829846008
**cg06497893**	4	152454119	FAM160A1	-0.077181	-0.157849813933024	0.0667401829846008
**cg25370859**	12	107712557	BTBD11;BTBD11	0.0165048	0.737893631015884	0.0667401829846008
**cg03758181**	10	102989325	FLJ41350;LBX1	0.0117322	0.387474561943846	0.0667401829846008
**cg20118422**	15	64455210	PPIB;PPIB	0.0545486	0.699531580451291	0.0667401829846008
**cg10007520**	16	88658109	ZC3H18;ZC3H18	-0.0960955	-0.208661663550529	0.0667401829846008
**cg14069192**	9	99381052	CDC14B;CDC14B	0.0199868	0.369733209362439	0.0667401829846008
**cg22340644**	7	75368344	HIP1	0.00812151	0.494559271270775	0.0667401829846008
**cg16618685**	16	23608722	NDUFAB1	-0.0737672	-0.124192346586474	0.0667401829846008
**cg27072387**	2	33171699	LTBP1	0.00833402	0.507248483134157	0.0667401829846008
**cg18331855**	17	1131781		0.0277704	0.679807548501624	0.0667401829846008
**cg22489991**	6	11383195	NEDD9;NEDD9	-0.0898996	-0.170678605305438	0.0667401829846008
**cg14719675**	2	25142882	ADCY3	0.0197358	0.475800999674804	0.0667401829846008
**cg10932242**	14	100659592		0.0217799	1.15157032343142	0.0667401829846008
**cg26653326**	8	124742606	ANXA13;ANXA13	-0.124359	-0.276655350261752	0.0668638661234761
**cg08104527**	18	13217953	C18orf1;C18orf1	0.0161083	0.355005153130846	0.0669305281337343
**cg16632132**	16	1122873	LOC146336	0.040199	0.649009570646493	0.0669929898153562
**cg23294576**	4	89128206	ABCG2	-0.079682	-0.162619380151936	0.0670918661730173
**cg01430633**	9	138987312	NACC2	0.0407259	0.81621312912713	0.0670918661730173
**cg21955154**	4	40999951	APBB2;APBB2;APBB2	-0.0610445	-0.15562056166921	0.0672382154742153
**cg21442773**	1	236029828	LYST	0.0186753	0.862712341856214	0.0673722200276347
**cg03554573**	11	111155021	C11orf53	0.0761961	0.271203745384014	0.0673722200276347
**cg13608561**	12	68039127		-0.00616156	-0.00910854410134222	0.0673722200276347
**cg08844364**	6	108850201		0.00806831	0.0119525157018139	0.0673722200276347
**cg26589485**	17	1838439	RTN4RL1	-0.01872	-0.0285102968769093	0.0673722200276347
**cg15067515**	12	49667670		-0.0473166	-0.0784004114853347	0.0673722200276347
**cg01919034**	15	69741819		-0.099886	-0.204032278831079	0.0673722200276347
**cg22944187**	12	128306001		-0.0594492	-0.0959732587125408	0.0674981221322804
**cg20684937**	5	12661875		-0.0416046	-0.0641673706237992	0.0674981221322804
**cg00561610**	3	52480253	SEMA3G	0.00865477	0.0128215351147261	0.0674981221322804
**cg04725029**	1	15850746	CASP9;CASP9;CASP9;CASP9	0.0186687	0.539788590720692	0.0674981221322804
**cg13974753**	5	149553071	CDX1	-0.0130167	-0.0194950122693138	0.0676818262290003
**cg07576016**	5	1523261	LPCAT1	0.0154165	0.72098387232332	0.0676939125229721
**cg24634568**	1	43250769		0.0795442	0.616146638289077	0.0676939125229721

**cg15924509**	1	2322543	RER1;MORN1	0.0081002	0.385113784340493	0.0676939125229721
**cg10884662**	15	75871802	PTPN9	0.0388406	0.713801807073141	0.0676939125229721
**cg14832349**	16	54301124	LOC100996338	-0.117309	-0.228800020059811	0.0676939125229721
**cg26325497**	19	54199538	MIR525	-0.121804	-0.434835675413966	0.0676939125229721
**cg20819397**	9	123964132	RAB14;RAB14	0.0155343	0.793614066711042	0.0676939125229721
**cg21325609**	15	90645961	IDH2;IDH2	0.00940004	0.50362442770184	0.0677792193279821
**cg04193484**	12	57620856		0.0187291	0.631442802911594	0.0679131757592796
**cg14397361**	9	140149997	COBRA1	0.0464092	1.86275053013731	0.0679131757592796
**cg05118743**	7	36013332		-0.0296357	-0.0471633898779721	0.0679131757592796
**cg22346963**	1	172247606	DNM3;DNM3	-0.0764813	-0.161761733001979	0.0679131757592796
**cg10585421**	8	52850164		-0.0585772	-0.119620198552922	0.0679131757592796
**cg24619626**	15	25380133	PAR1	-0.0198069	-0.0299677311168329	0.0679131757592796
**cg00661205**	13	42400902	KIAA0564;KIAA0564	-0.0537476	-0.101416748230367	0.0679131757592796
**cg15288815**	8	74659085	STAU2;STAU2;STAU2;STAU2;STAU2;STAU2;STAU2;STAU2;STAU2;STAU2;STAU2;STA	0.00688114	0.626238705440777	0.0679225427004522
**cg15834198**	2	65701984		-0.0272748	-0.0421752846767146	0.0680264516709495
**cg22932101**	14	71815816		-0.113009	-0.254664391395558	0.0680555650389075
**cg17026475**	15	37391480	MEIS2;MEIS2;MEIS2;MEIS2;MEIS2;MEIS2;MEIS2;MEIS2;MEIS2;MEIS2	0.0114949	0.670500229142117	0.068135787766705
**cg02568904**	19	36606206	TBCB;TBCB;TBCB;POLR2I	0.0285316	0.725565119142183	0.0682610117226496
**cg21045612**	9	17134839	CNTLN;CNTLN;CNTLN;CNTLN	0.0267191	0.578398687214591	0.0682710808114525
**cg13023786**	15	79606973	TMED3;TMED3;TMED3	0.00485113	0.00712809767445071	0.0683829546751411
**cg05435065**	7	75544030	POR	0.0232064	0.971863494070089	0.0683829546751411
**cg03119031**	6	31620547	BAT3;BAT3;BAT3;BAT3	0.00539473	0.417957333411673	0.0686618404415626
**cg18228326**	1	17446068	PADI2	0.00535572	0.360465727343792	0.068825152040529
**cg24654547**	16	68057165	DUS2L;DDX28;DDX28	0.0801211	1.59762538018473	0.068825152040529
**cg19350162**	12	124927552	NCOR2;NCOR2	-0.0361134	-0.0577976526267136	0.068825152040529
**cg04581285**	16	24682924	LINC01567	-0.0578514	-0.1024754114988	0.068825152040529
**cg24529484**	19	47933421	SLC8A2	0.0167611	0.470417053481473	0.068825152040529
**cg00221445**	8	74183964		-0.0392332	-0.0611677225412433	0.068825152040529
**cg09336406**	2	20068906		0.0200486	0.60305963078358	0.068825152040529
**cg12928759**	2	74756470	HTRA2;AUP1;HTRA2	0.0282419	0.557838571327992	0.068825152040529
**cg24159214**	3	50402751	CACNA2D2;CACNA2D2	0.0178771	0.427387653273019	0.068825152040529
**cg15427004**	3	38179858	ACAA1;ACAA1;ACAA1;MYD88	0.0100488	0.492674273285443	0.068825152040529
**cg19325000**	2	74710102	CCDC142;CCDC142;TTC31;TTC31	0.00945089	0.752825898719212	0.068825152040529
**cg09868336**	3	68981866	FAM19A4;FAM19A4	0.0335389	0.566566739088038	0.068825152040529
**cg11227817**	11	133716095	SPATA19;SPATA19	-0.0371399	-0.0582228061554492	0.068825152040529
**cg24356725**	4	1340288	KIAA1530	0.0100549	0.490637109857105	0.068825152040529
**cg13482308**	9	37002685	PAX5	0.0292463	0.704030128842211	0.068825152040529
**cg00680003**	6	30523449	GNL1;PRR3;PRR3	0.0375622	0.548565403805705	0.0688574585918995
**cg04704021**	7	100942858		0.034448	0.524544745519798	0.0688574585918995
**cg20974817**	16	89789246	ZNF276;ZNF276;ZNF276;ZNF276;ZNF276;ZNF276;ZNF276;ZNF276;ZNF276;ZNF276;ZN	0.0172568	1.09493594582349	0.0689533268497513
**cg05373655**	1	228660732		-0.0543392	-0.132854734858933	0.0689639948460279
**cg09718295**	6	160182856	ACAT2	0.0245779	0.826662953709992	0.069124782163179
**cg09039698**	16	30007384	HIRIP3;INO80E;HIRIP3	0.0081088	0.553085776371742	0.0692049703646304
**cg16406226**	20	42086458	SFRS6	0.0290504	0.506625376419407	0.0692288869536783
**cg01706621**	15	88247258		-0.0607077	-0.100583133167584	0.0692288869536783
**cg27022615**	11	70049105	FADD	0.0120178	0.68621313813335	0.0692561608659109
**cg20137918**	1	52388141	RAB3B	-0.0546464	-0.0888374443167739	0.0692561608659109
**cg13137376**	6	30712221	IER3	0.0332586	0.543056800558439	0.0692561608659109
**cg24714100**	6	30290603	HCG18;HCG18	-0.096206	-0.224224641526472	0.0692561608659109
**cg20964023**	14	91976739	SMEK1	0.0134801	0.497413742163714	0.0692561608659109
**cg02641288**	5	1883011	IRX4	0.00803118	0.492895025293279	0.0692561608659109
**cg00731917**	18	60263973		0.0122251	0.433683677050834	0.0692561608659109
**cg02986878**	9	117275370		-0.0751748	-0.135623665856737	0.0693509726914498
**cg11825411**	7	50631741	DDC	-0.0105446	-0.0158400268623194	0.0693509726914498
**cg10825881**	15	52202509	TMOD3	-0.058409	-0.104606622684285	0.0693509726914498
**cg26220419**	11	107880052	CUL5;CUL5	0.0113388	1.28175759888744	0.0693509726914498
**cg04905448**	2	69002063	ARHGAP25;ARHGAP25;ARHGAP25;ARHGAP25;ARHGAP25;ARHGAP25	0.00927822	0.674067907218933	0.0693509726914498
**cg07995179**	6	85484287		0.00864154	0.514481263165439	0.0693509726914498
**cg12662767**	14	75421955	PGF;PGF;PGF;PGF;PGF;PGF	0.00824612	0.452671372167894	0.0693958169019876
**cg15119683**	6	38533031	BTBD9;BTBD9;BTBD9;BTBD9	-0.0937123	-0.181444548728506	0.0694216674076264
**cg10830758**	10	95462430	C10orf4	0.0128775	0.663971368229475	0.0695020477439938
**cg15363887**	11	57414557	YPEL4	0.0416918	0.427968322678043	0.0695070059736163
**cg12171249**	7	11606963	THSD7A	-0.0686292	-0.16384789115108	0.0695207673697441
**cg03480336**	21	33800475	EVA1C;EVA1C;EVA1C	-0.0283903	-0.0439254787173863	0.0698413931085226
**cg00529697**	5	75013131	C5orf37;C5orf37	0.0431308	0.549096938240806	0.0698413931085226
**cg21294812**	6	132722770	MOXD1	0.0114735	0.619699660142433	0.0698413931085226
**cg08886046**	2	130691738	LOC389033	0.0956877	0.525160982547562	0.0698413931085226
**cg24182293**	3	193852998	HES1	0.0181238	0.360335670578433	0.0698413931085226
**cg18753296**	6	31630923	BAT4	-0.0196436	-0.0305931726424344	0.0698413931085226
**cg00597931**	8	142179697	DENND3	-0.0160966	-0.0240872037031929	0.0698413931085226
**cg05862114**	7	130132259	MEST;MEST;MESTIT1;MEST	0.0599482	0.318030270554114	0.0698413931085226
**cg17053871**	20	34043114	CEP250	0.0228153	0.637233156770247	0.0698413931085226
**cg11059266**	4	48782139	FRYL;FRYL	0.0373426	0.440988748437561	0.0698413931085226
**cg24059612**	19	54585167	TARM1	-0.0598222	-0.0995865756832643	0.0698413931085226
**cg17117277**	19	3822126	ZFR2	0.0127871	0.79943715311292	0.0698413931085226
**cg10507649**	21	43916595	RSPH1	0.0148768	0.48146894460905	0.0698413931085226
**cg25044026**	6	84418700	SNAP91;SNAP91;SNAP91;SNAP91;SNAP91;SNAP91;SNAP91	0.023455	0.401170126499314	0.0698413931085226
**cg19175585**	16	71392493	CALB2;CALB2;CALB2	0.00589759	0.39878600832016	0.0698413931085226
**cg02081018**	21	38445169	PIGP;PIGP;TTC3;PIGP	0.0162754	0.458734723964053	0.0698413931085226
**cg10858909**	4	99850288	EIF4E;EIF4E;EIF4E;EIF4E;EIF4E	0.0046494	0.44863626650379	0.0698413931085226
**cg21183638**	22	31701338		-0.0779385	-0.159734882689133	0.0698413931085226
**cg15809122**	22	23487482	RAB36	0.0127073	0.495904593242237	0.0698413931085226

**cg05170515**	14	24899109	CBLN3;KHNYN	0.0369155	0.631253919376183	0.0698413931085226
**cg17434634**	19	49522954		0.0625021	0.323194549247236	0.0698413931085226
**cg23057485**	8	134052341	SLA;TG;SLA;SLA	0.0172633	0.0272331220070082	0.0698413931085226
**cg03823108**	20	1099106	PSMF1;PSMF1	0.0148494	0.755591259548756	0.0698413931085226
**cg13868290**	16	19089964	COQ7;COQ7	-0.0795518	-0.138956662101638	0.0698413931085226
**cg06579459**	6	28263113	PGBD1;PGBD1	-0.0920644	-0.207852238723064	0.0698413931085226
**cg05865746**	17	2699553	RAP1GAP2;RAP1GAP2	0.0313676	0.885839031406083	0.0698413931085226
**cg06186057**	6	28186447	LOC222699	0.0103569	0.640634648988125	0.0698413931085226
**cg05871997**	6	56819623	BEND6;DST	0.00751811	0.596703030421526	0.0698413931085226
**cg04027443**	8	1169032		-0.0641812	-0.120224871160338	0.0698413931085226
**cg08125682**	16	11038455	CLEC16A;CLEC16A	0.0324865	0.367469249278897	0.0698413931085226
**cg10541587**	4	106067761	TET2;TET2	0.0178092	0.564057581704308	0.0698413931085226
**cg03504384**	1	87380170	SEP15;SEP15;HS2ST1;HS2ST1	0.00729401	0.558832551420904	0.0698413931085226
**cg17236617**	1	6454202	ACOT7	0.00790324	0.559599714002028	0.0698413931085226
**cg20434586**	6	43237511	TTBK1	0.0435669	0.716452177040798	0.0698413931085226
**cg19479828**	5	177555469		-0.0291903	-0.045385617250589	0.0698413931085226
**cg22252462**	6	31622318	APOM	-0.0760357	-0.149327898231629	0.0698413931085226
**cg01459748**	11	113817020	HTR3B	-0.0821816	-0.176195477535708	0.0698413931085226
**cg18177613**	9	135359969	C9orf171;C9orf171	-0.062404	-0.125138064522526	0.0698413931085226
**cg20225569**	20	5931325	TRMT6;TRMT6;MCM8;MCM8;MCM8;MCM8;MCM8;MCM8;MCM8;MCM8;MCM8;MCM8	0.00436967	0.495949524407889	0.0698413931085226
**cg17327377**	14	67009641	GPHN;GPHN	-0.0623657	-0.111378057752158	0.0698413931085226
**cg13842237**	21	47064079		0.0131766	0.525077759524715	0.0698413931085226
**cg07987587**	22	42486991	NDUFA6	0.018362	0.786279603450031	0.0698413931085226
**cg15049439**	19	7167445	INSR;INSR	-0.0481138	-0.0753302247976512	0.0698413931085226
**cg06174476**	9	98645616	ERCC6L2;ERCC6L2	-0.0584218	-0.0968430468579208	0.0698413931085226
**cg16208491**	6	4021748	PRPF4B	0.010376	0.490016658358887	0.0698413931085226
**cg09242721**	3	3168535	TRNT1	0.0421559	0.676937471561504	0.0698413931085226
**cg26126181**	5	6347990		-0.0209342	-0.031562945501443	0.0698413931085226
**cg26016402**	5	7851674	C5orf49	-0.0389219	-0.0666996257054573	0.0698413931085226
**cg19233405**	1	154988721	ZBTB7B	0.0226905	0.399852399125438	0.0698413931085226
**cg18481087**	3	160474064	PPM1L;PPM1L	0.030746	0.492797169247581	0.0698413931085226
**cg14975881**	19	54389945	PRKCG	-0.0811385	-0.169548326951636	0.0698413931085226
**cg01457699**	8	100511131	VPS13B;VPS13B	-0.0683881	-0.385476313963684	0.0698413931085226
**cg22711405**	12	54379726	HOXC10;HOXC-AS3	0.0550108	0.28373042283531	0.0698413931085226
**cg16796215**	11	62372128	EML3;EML3;EML3	-0.0814223	-0.154540188488106	0.0698413931085226
**cg01669394**	2	201181269	SPATS2L;SPATS2L;SPATS2L;SPATS2L;SPATS2L;SPATS2L;SPATS2L	0.00151787	0.00221021340781325	0.0698413931085226
**cg12798675**	14	102171582		-0.0898525	-0.195976780630804	0.0698413931085226
**cg19855563**	17	79253533	SLC38A10;SLC38A10	-0.0238517	-0.0370919301943086	0.0698413931085226
**cg17616545**	5	154403647		-0.0789722	-0.166781405794859	0.0698465658529427
**cg15112848**	11	35000144	PDHX;PDHX;PDHX	0.0167298	0.0256156347055744	0.0698465658529427
**cg14021572**	4	105416420		0.0102693	0.31886250359028	0.0698465658529427
**cg12594290**	6	11972963		-0.0328625	-0.0549472732750475	0.0698465658529427
**cg27210354**	5	177051706	LOC202181	-0.0267078	-0.0430552083030048	0.0698465658529427
**cg06007323**	4	6784769	KIAA0232;KIAA0232	0.0245833	0.69920077071713	0.0698465658529427
**cg14642671**	17	48450604	EME1;MRPL27;EME1;EME1;EME1	0.00576583	0.478880720404483	0.0698465658529427
**cg19782148**	3	105971299		-0.0475092	-0.0748989848376592	0.0698465658529427
**cg18019150**	7	6452872	DAGLB;DAGLB	0.0178776	0.0273640269814808	0.0698465658529427
**cg00177496**	3	197283127	BDH1;BDH1;BDH1	0.0103644	0.650495294134149	0.0698465658529427
**cg22033857**	8	58947178	FAM110B	0.0153149	0.023372387813928	0.0698465658529427
**cg06449018**	20	55553840		-0.00679228	-0.00997490718490126	0.0698465658529427
**cg10865856**	6	30043061	RNF39;RNF39	0.00964634	0.583619430708727	0.0698465658529427
**cg11162385**	20	25604740	NANP	0.05241	1.15639382545983	0.0698465658529427
**cg19461907**	19	17336467	OCEL1	-0.0785546	-0.162230862372157	0.0698465658529427
**cg10880709**	4	1283506	MAEA;MAEA;MAEA;MAEA;MAEA	0.0143576	0.921842625743294	0.0698465658529427
**cg01923061**	10	50821068	CHAT;CHAT;CHAT;CHAT;CHAT;CHAT;CHAT	0.00590914	0.338679433140545	0.0698465658529427
**cg19598832**	19	1646448	TCF3;TCF3	0.0437975	0.0720391140758435	0.0698465658529427
**cg15834355**	12	54442075	HOXC4	-0.0434302	-0.0764448455897819	0.0698465658529427
**cg23116115**	4	1004261	FGFRL1;FGFRL1	0.0237759	0.361003408761951	0.0698465658529427
**cg26842801**	5	55070008	DDX4;DDX4;DDX4;DDX4	-0.0585652	-0.101022401349629	0.0698465658529427
**cg21196575**	13	95245226	TGDS	-0.062434	-0.13401142079083	0.0698465658529427
**cg18652367**	5	180017689	SCGB3A1	0.013896	0.775532095882685	0.0698465658529427
**cg13102693**	2	220094685	ANKZF1;ANKZF1;ANKZF1;ATG9A;ATG9A;ATG9A	0.0210551	1.22039279954065	0.0698465658529427
**cg25068445**	9	139780417	TRAF2	0.0104808	0.455165787412867	0.0698465658529427
**cg03897156**	20	54967330	CSTF1;AURKA;AURKA;AURKA;AURKA;CSTF1;AURKA;AURKA;AURKA;AURKA;AURKA	0.0093325	0.510838481999686	0.0698465658529427
**cg26931154**	3	87347249		-0.0552169	-0.519634594707325	0.0698465658529427
**cg07312150**	11	61585346	FADS1;FADS2;FADS2	-0.0891608	-0.169326699636176	0.0698465658529427
**cg07783291**	19	33178679		-0.0620109	-0.14885821565765	0.0698465658529427
**cg19747686**	9	95155516	OGN;OGN;CENPP;CENPP;OGN;OGN	-0.0418793	-0.0686585303351603	0.0698465658529427
**cg09748306**	16	28005435	GSG1L	-0.0606201	-0.123279964466073	0.0698465658529427
**cg05344692**	16	68544404		0.0465836	0.437760387580751	0.0698465658529427
**cg15538009**	14	51304083		-0.061505	-0.110979357372593	0.0698465658529427
**cg25874642**	12	50898251	DIP2B	-0.0153456	-0.0232568355561353	0.0698465658529427
**cg06816419**	14	52324109		0.0163559	0.0255140454114446	0.0698465658529427
**cg22091798**	11	62446605	UBXN1	0.0148272	0.493258285825554	0.0698465658529427
**cg11296531**	14	96343010	TUNAR	0.00933302	0.538682920665319	0.0698465658529427
**cg08599085**	8	134909737		-0.0106991	-0.0159942152377308	0.0698465658529427
**cg14924567**	1	109203837	HENMT1;HENMT1;HENMT1	0.0151045	0.604072812364409	0.0698465658529427
**cg14496314**	3	61754981	PTPRG	-0.0704132	-0.134523049794577	0.0698465658529427
**cg14159373**	19	1696132		-0.0786201	-0.175799227796821	0.0698465658529427
**cg15791348**	19	1354663	MUM1	0.00924498	0.593145802793815	0.0698465658529427
**cg20146387**	14	20919009	OSGEP	-0.0779964	-0.144829963242775	0.0698465658529427
**cg07326768**	15	86337453	KLHL25	0.0120265	0.607424543280187	0.0698465658529427

**cg26175410**	17	74233903	RNF157	-0.0202907	-0.0305491679259575	0.0698465658529427
**cg19120943**	2	113304419	POLR1B;POLR1B;POLR1B;POLR1B;POLR1B;POLR1B;POLR1B	-0.0627494	-0.114893840389393	0.0698465658529427
**cg19948014**	21	33032656	SOD1	0.0065562	0.551876613321316	0.0698465658529427
**cg18590436**	5	159582684		-0.0298833	-0.0461271162428214	0.0698465658529427
**cg26213885**	1	88675834		-0.0169038	-0.0263495156835324	0.0698465658529427
**cg26095478**	22	46377526		-0.0678056	-0.123937180161904	0.0698465658529427
**cg24863821**	10	3793247		-0.0128338	-0.019431152077021	0.0698465658529427
**cg27380774**	11	1000560	AP2A2	-0.0235567	-0.0365569525791144	0.0698465658529427
**cg16895948**	1	145610875	POLR3C;POLR3C;RNF115	0.0125308	0.910476005474599	0.0698465658529427
**cg21694044**	22	41215540	SLC25A17	0.0337799	0.356285627744798	0.0698465658529427
**cg08164950**	6	147981771		-0.0312777	-0.0483289087414644	0.0698465658529427
**cg00994643**	8	23082826	TNFRSF10A	0.0168936	0.784023726389372	0.0698465658529427
**cg25960393**	8	9106559		-0.0976028	-0.520849470489707	0.0698465658529427
**cg13173369**	1	32444348		-0.0285244	-0.0467436392800985	0.0698465658529427
**cg25942155**	20	2607828	TMC2	-0.0121865	-0.0182994206022411	0.0698465658529427
**cg06070291**	15	44828899	EIF3J	0.0386422	0.871203717104843	0.0698465658529427
**cg09753043**	11	43596920		0.0156151	0.489485721447394	0.0698465658529427
**cg18289490**	20	48532070	SPATA2;SPATA2	0.0525901	0.899503126304331	0.0698465658529427
**cg12194493**	4	493061	PIGG;ZNF721;PIGG;PIGG;PIGG;ZNF721	0.00484296	0.390896765553131	0.0698465658529427
**cg11989968**	2	173421429	PDK1;PDK1;PDK1;PDK1	0.00793331	0.62196614418815	0.0698465658529427
**cg12789522**	15	78423758	CIB2;CIB2	0.0294098	0.450542196222334	0.0698465658529427
**cg06316818**	15	83306789	CPEB1;CPEB1	-0.0515781	-0.0826188661110486	0.0698656661582179
**cg10483909**	1	243418637	CEP170;CEP170;CEP170;SDCCAG8;CEP170;CEP170;CEP170	0.00563946	0.343912413988546	0.0698656661582179
**cg09870066**	2	68546685	CNRIP1;CNRIP1;CNRIP1;CNRIP1	0.0251553	0.554688962033358	0.0698656661582179
**cg13471915**	1	162531167	UAP1	0.00650095	0.365706009454905	0.0698656661582179
**cg01114186**	6	31940183	STK19;STK19;STK19;DOM3Z	0.0129503	0.596390213262971	0.0698656661582179
**cg24554839**	1	166124903	MIR921;FAM78B	-0.0186429	-0.029865228175759	0.0698656661582179
**cg14792270**	11	85955165	EED;EED	-0.0444832	-0.155144302977824	0.0698656661582179
**cg10319399**	8	145018285	PLEC1;PLEC1;PLEC1;PLEC1;PLEC1;PLEC1	0.023048	0.647783904676687	0.0698656661582179
**ch.2.157204871R**	2	157496625		0.0109292	0.386869091163178	0.0698656661582179
**cg02625641**	15	59041175	ADAM10	0.0442595	0.931903413038198	0.0698656661582179
**cg01771876**	2	11886613	LPIN1	0.0242658	0.729643425856769	0.0698656661582179
**cg17938619**	18	8929488		-0.0429504	-0.0695309794259912	0.0698656661582179
**cg22837406**	14	76618409	C14orf118;C14orf118	0.0246656	0.486244586802427	0.0698656661582179
**cg01579019**	8	27695516	PBK	0.00978815	0.497516790145951	0.0698656661582179
**cg17864958**	1	224370909	DEGS1;DEGS1	0.044987	0.557143748203732	0.0698656661582179
**cg13529288**	7	100209880	MOSPD3;MOSPD3;MOSPD3;MOSPD3	0.0110005	0.692175081776973	0.0698656661582179
**cg27365485**	11	64126452	RPS6KA4;RPS6KA4	0.026834	0.765039727677897	0.0698812827786955
**cg02676434**	3	38066785	PLCD1;PLCD1;PLCD1	0.00430861	0.382352013515795	0.069933977738841
**cg05949385**	1	199984216		-0.0953656	-0.182742827361973	0.0703791515614276
**cg18663063**	5	68710907	MARVELD2;MARVELD2	0.0396169	0.480489590292583	0.0703791515614276
**cg23525438**	6	17016226		0.0977455	0.60453010276933	0.0705201853386366
**cg06014792**	17	7382760	ZBTB4;ZBTB4	0.0185336	0.617312555674958	0.0705201853386366
**cg11990813**	17	2603739	KIAA0664	-0.034898	-0.0550739769114628	0.0707051197797108
**cg22111723**	13	21872664		0.0196094	0.920862944377972	0.0707051197797108
**cg10151248**	11	66639567	PC;PC;PC	-0.0278134	-0.0430429552043742	0.070755072675609
**cg20997773**	11	117667841	DSCAML1	0.0155901	0.478624042642867	0.0707707474335027
**cg22238304**	2	206981412	LOC100329109	-0.0570367	-0.122952157528604	0.0707707474335027
**cg23105839**	10	135050004	VENTX	0.0207225	0.842648463882748	0.0707707474335027
**cg02239805**	7	114562316	MDFIC;MDFIC;MDFIC;MDFIC;MDFIC;MDFIC	0.0392744	0.557417713504868	0.0707707474335027
**cg01216311**	17	7025714		-0.0669433	-0.438671125866982	0.0707707474335027
**cg01337968**	7	129355101	NRF1;NRF1;NRF1;NRF1	-0.0376147	-0.0584057326485407	0.0707707474335027
**cg05665069**	3	119883253		-0.0158786	-0.0238812756240304	0.0707707474335027
**cg02729011**	16	699500	WDR90	0.0228263	0.62762758322207	0.0707707474335027
**cg04210100**	2	9614471	IAH1	0.0210803	0.690230249999299	0.0707707474335027
**cg04111478**	11	1991677		0.0705047	0.425941334234999	0.0707707474335027
**cg04330730**	1	48472619		-0.0612519	-0.137474558500878	0.0707707474335027
**cg19774733**	9	37422586	GRHPR	0.0295388	0.860020129994756	0.0707707474335027
**cg13778778**	20	35808076	C20orf132;RPN2;RPN2;C20orf132;C20orf132	0.0208204	0.828758300183899	0.0707707474335027
**cg22476645**	16	15150025	NTAN1	0.0122546	0.466217257500362	0.0707707474335027
**cg19496491**	11	12695499	TEAD1	0.0160433	0.894008366304978	0.0707707474335027
**cg07828939**	11	3021385		0.0128664	0.0193565148993785	0.0707707474335027
**cg27275821**	1	89144331	PKN2-AS1	-0.0714497	-0.12588410148372	0.0707707474335027
**cg04100564**	12	112189172	ACAD10;ACAD10	-0.0687675	-0.120352453845238	0.0707707474335027
**cg11895632**	16	69042429	TANGO6	-0.0428722	-0.0672063207553237	0.0707707474335027
**cg17395226**	5	156887490	NIPAL4	0.00654903	0.322699819960425	0.0707707474335027
**cg27026509**	1	36348944	EIF2C1;EIF2C1	0.0205239	0.667790270486458	0.0707707474335027
**cg16722016**	12	90119262		-0.0635798	-0.106299889411578	0.0707707474335027
**cg16267901**	12	58023160	B4GALNT1;B4GALNT1;B4GALNT1	-0.072248	-0.147467592783699	0.0707707474335027
**cg13307023**	16	1359278	UBE2I;UBE2I;UBE2I;UBE2I	0.00420466	0.325218724318322	0.0707707474335027
**cg17115258**	15	102192853	TM2D3;TM2D3	0.00640854	0.405650921232712	0.0707707474335027
**cg23382141**	6	73976708	KHDC1	-0.0444658	-0.0716511214297538	0.0707707474335027
**cg14697246**	3	48487981	ATRIP;ATRIP	0.00728843	0.395045069979816	0.0707707474335027
**cg02328440**	1	235813839	GNG4;GNG4;GNG4	0.0116789	0.452286045292904	0.0707707474335027
**cg25312546**	19	4307208	FSD1	-0.0680868	-0.129274539558278	0.0707707474335027
**cg05835573**	2	159650648	DAPL1	-0.0694917	-0.115993493364808	0.0707707474335027
**cg01431319**	10	51595391	TIMM23;TIMM23;TIMM23;TIMM23B;TIMM23B;TIMM23B	-0.03468	-0.0562783726960634	0.0707707474335027
**cg00422716**	7	123484985	HYAL4	-0.0544499	-0.0983016040849037	0.0707707474335027
**cg15817341**	5	150538450	ANXA6	-0.0415419	-0.0666779382349045	0.0707707474335027
**cg12074084**	1	45140087	C1orf228;TMEM53;TMEM53	0.0298123	0.50090855314915	0.0707883108423024
**cg05702927**	2	204206071	ABI2;ABI2;ABI2;ABI2	-0.105763	-0.245126577572922	0.0710937980996139
**cg24647363**	6	143857972		0.0167026	0.581030736689269	0.0710967962909368

**cg13064658**	1	212003989	LPGAT1;LPGAT1	0.0399179	0.458487850163733	0.0710967962909368
**cg01353139**	8	124170018		0.0126148	0.457718374402177	0.0710967962909368
**cg23397741**	17	35014588		0.0185459	0.587545312586327	0.0710967962909368
**cg25249300**	2	54483341	TSPYL6;ACYP2;TSPYL6	-0.00474265	-0.00696604941619033	0.0710967962909368
**cg23007574**	19	41942070	ATP5SL;ATP5SL;ATP5SL;ATP5SL;ATP5SL;ATP5SL;ATP5SL	-0.0405207	-0.065797802796558	0.0710967962909368
**cg26286479**	12	69924501	FRS2;FRS2;FRS2;FRS2;FRS2;FRS2;FRS2;FRS2	-0.0273228	-0.0412442757846175	0.0710967962909368
**cg01101619**	6	4088409	C6orf201;C6orf201;C6orf201	-0.0143423	-0.021267353945835	0.0710967962909368
**cg23651826**	11	115630531		0.0416057	0.422629237706695	0.0710967962909368
**cg15890173**	5	74532862	ANKRD31	0.0111636	0.574711398765725	0.0710967962909368
**cg01646610**	19	19976784	ZNF253;ZNF253	0.0128517	0.410537691341028	0.0710967962909368
**cg06417885**	8	47108318		-0.0566014	-0.102898923125476	0.0710967962909368
**cg02943305**	7	31685368	CCDC129	-0.141898	-0.395620139514931	0.0710967962909368
**cg12323760**	1	40544720	PPT1;PPT1	-0.0850975	-0.170418438040574	0.0710967962909368
**cg19987349**	8	98993142	MATN2;MATN2	-0.0975016	-0.191523726875244	0.0710967962909368
**cg16788538**	7	72972107	BCL7B	0.0193403	0.763440749226576	0.0710967962909368
**cg20460852**	8	23082822	TNFRSF10A	0.0382863	0.92579980607176	0.0710967962909368
**cg09852920**	17	48277216	COL1A1	0.0343744	1.24604186720731	0.0710967962909368
**cg24576206**	22	25865846		-0.0273912	-0.0427719208511121	0.0710967962909368
**cg14704270**	22	50639024	SELO	0.0468055	0.609815783590264	0.0710967962909368
**cg19966981**	17	71564318	SDK2	-0.0285985	-0.0447280259990286	0.0710967962909368
**cg08358233**	10	48634114		-0.0403256	-0.0654035967441556	0.0710967962909368
**cg06504337**	12	123465174	ARL6IP4;ARL6IP4;ARL6IP4;ARL6IP4;ARL6IP4;ARL6IP4;ARL6IP4;ARL6IP4;ARL6IP4	0.0156072	0.50672458117272	0.0710967962909368
**cg05525867**	15	75661090	MAN2C1	0.0129362	0.881498550831165	0.0710967962909368
**cg20550383**	14	104313914	PPP1R13B;PPP1R13B;LINC00637	0.0265094	0.442720400816279	0.0710967962909368
**cg07751641**	5	134826244		0.0231945	0.768016914339213	0.0710967962909368
**cg12001457**	2	50163732	NRXN1;NRXN1;NRXN1	-0.0309652	-0.0486108145441262	0.0710967962909368
**cg02717630**	16	46865118	C16orf87	0.0245133	0.653203850732497	0.0710967962909368
**cg21076259**	11	35161345	CD44;CD44;CD44;CD44;CD44	0.00681637	0.34278417244506	0.0710967962909368
**cg00468356**	7	138176808	TRIM24;TRIM24	-0.0478337	-0.0764079497734727	0.0710967962909368
**cg20647962**	15	41136443	SPINT1;SPINT1;SPINT1	0.0228898	0.518989143876452	0.0710967962909368
**cg05302416**	11	2466037	KCNQ1	0.0222925	0.525464821839923	0.0710967962909368
**cg23312248**	12	123465515	ARL6IP4;ARL6IP4;ARL6IP4;ARL6IP4	0.0196383	0.550765504113736	0.0710967962909368
**cg00460807**	3	45731126	SACM1L	0.0237026	0.814695132381025	0.0710967962909368
**cg17448575**	15	40762993	CHST14	0.01404	0.659883871397479	0.0710967962909368
**cg06635202**	2	8165386		-0.0114457	-0.0170251061642496	0.0710967962909368
**cg26157020**	9	129240339	MVB12B	-0.0514936	-0.0915200037484988	0.0710967962909368
**cg09092901**	2	33049964	LINC00486	-0.0637822	-0.126158582804558	0.0712834664990888
**cg12271759**	2	40468548	SLC8A1;SLC8A1;SLC8A1;SLC8A1	-0.0484016	-0.0835981617290261	0.0713479161220902
**cg22355691**	19	12739144	ZNF791	-0.080166	-0.141131684732491	0.0713479161220902
**ch.7.109945386F**	7	110158150		0.0265003	0.470969512103851	0.0713985471131724
**cg07102665**	20	6210894		-0.0345425	-0.0564080811319779	0.0714921875435001
**cg12214665**	6	138188557	TNFAIP3	0.0175645	0.75515702796533	0.0714921875435001
**cg24934063**	7	121944173	FEZF1;FEZF1	0.0230515	0.339876855722695	0.0714921875435001
**cg17307811**	19	55916316	UBE2S	-0.074769	-0.144346715519751	0.0714921875435001
**cg07136338**	17	33814819	SLFN12L	0.0115719	0.481347394944446	0.0715237813221254
**cg20136584**	1	204121503	ETNK2	0.00865915	0.384808611562647	0.0715237813221254
**cg02921604**	19	54297098	NLRP12;NLRP12	-0.0471388	-0.0812222112422541	0.0715237813221254
**cg04878489**	6	43139942	SRF;SRF	0.0278975	0.534637696434597	0.0715237813221254
**cg24238564**	11	65779343	CST6	0.0288897	0.683123740502286	0.0715237813221254
**cg09881019**	19	45250987	BCL3	0.0463173	0.316630338254275	0.0715237813221254
**cg02507889**	18	76754902	SALL3	-0.0372495	-0.0631329553847701	0.0715237813221254
**ch.3.52786R**	3	3083388	CNTN4;CNTN4;CNTN4	0.0180971	0.27026268553803	0.0715371904069492
**cg04576025**	5	1386550		0.033262	1.15653768039061	0.0715371904069492
**cg25048344**	2	119914337	C1QL2	0.0125769	0.504910956654604	0.0717463943073639
**cg22834172**	17	37774349		0.0163155	0.593830610405995	0.0718102503016278
**cg09395034**	5	137801102	EGR1	0.0180389	0.533305832294327	0.0718102503016278
**cg24516890**	3	133293153	CDV3;CDV3;CDV3	0.0108353	0.44412567797567	0.0718102503016278
**cg02556655**	11	63975026	FERMT3;FERMT3	0.00906978	0.608737539697052	0.0718102503016278
**ch.19.683370F**	19	16239180	RAB8A	0.0280189	0.736434564972849	0.0718102503016278
**cg04379122**	10	130119293		-0.0620698	-0.113905236184627	0.0718258046962064
**cg26147668**	7	130081239	TSGA14	-0.0579189	-0.712952847408517	0.0718583329213108
**cg03814148**	4	113223849	ALPK1;ALPK1;ALPK1	0.0273369	0.0467306891009954	0.07186224295106
**cg20972078**	2	230320638	DNER	-0.0272535	-0.0418448546247008	0.07186224295106
**cg21950196**	1	228327428	GUK1;GUK1	0.0395742	0.227793729206285	0.07186224295106
**cg02938205**	3	49236810	CCDC36;CCDC36	0.0442754	0.644631019865415	0.07186224295106
**cg20110633**	19	44302906	LYPD5;LYPD5	0.0342252	0.793044937296685	0.07186224295106
**cg21367769**	6	25651194	SCGN	-0.0851737	-0.149802438405562	0.07186224295106
**cg09658066**	6	35995354	MAPK14;MAPK14;MAPK14;MAPK14	0.0360564	0.473860385010342	0.07186224295106
**cg18936327**	1	161060281	PVRL4	0.00744137	0.0111276950891701	0.07186224295106
**cg08740284**	15	40987165	RAD51;RAD51-AS1;RAD51;RAD51;RAD51	0.0180624	0.412864197071407	0.07186224295106
**cg26042512**	8	19117049		-0.0131378	-0.0196819639656028	0.07186224295106
**cg09238470**	16	30007069	HIRIP3;INO80E;HIRIP3	0.0073575	0.678950745155611	0.071963909760912
**cg21992350**	6	80657193	ELOVL4;ELOVL4	0.0136586	0.486493151065684	0.0720342832593362
**cg10756051**	2	198365397	HSPD1;HSPE1;HSPD1	0.00447031	0.3114214703404	0.0720342832593362
**cg24564491**	21	46238804	SUMO3	-0.0305167	-0.0489897573400001	0.0720842714624691
**cg25081106**	13	88324169	SLITRK5	0.0127584	0.354432339424024	0.0720842714624691
**cg10929299**	14	50334860		0.017405	0.562864116006389	0.0720842714624691
**cg26069562**	6	29691981	HLA-F;HLA-F;HLA-F	0.00573482	0.371291452738891	0.0720842714624691
**cg09653046**	4	129730880	JADE1;JADE1;JADE1;JADE1;JADE1;JADE1;JADE1;JADE1	0.0125111	0.564271259606026	0.0720842714624691
**cg09209510**	14	100288389	EML1;EML1	0.0066749	0.327161209201973	0.0720842714624691
**cg03987003**	1	145516092	GNRHR2;PEX11B	0.0378882	0.470087096683984	0.0720842714624691
**cg22802174**	1	40995949	ZNF684	-0.0626622	-0.13696457120092	0.0721278245126002

**cg03319894**	8	81402617	ZBTB10;ZBTB10	-0.109528	-0.198451054310256	0.0721278245126002
**cg26240353**	12	96429271	LTA4H	0.0036136	0.266689272920426	0.0721749871830667
**cg17170305**	20	49342101		-0.035263	-0.0543476124672553	0.0721749871830667
**cg09846903**	8	123657237		-0.0070028	-0.0104655578019956	0.0721749871830667
**cg04681368**	19	42806184	PRR19;PAFAH1B3;PAFAH1B3;PAFAH1B3	0.0366302	1.42364127895522	0.0721749871830667
**cg02523617**	4	39699380	UBE2K;UBE2K;UBE2K	0.0223182	0.72907861098882	0.0721749871830667
**cg25328184**	5	110406414	TSLP	0.00748944	0.457045494799244	0.0721749871830667
**cg03086376**	16	1876114	HAGH;HAGH;FAHD1;FAHD1;FAHD1	0.02091	0.86562070365507	0.0721749871830667
**cg27183602**	4	52709200	DCUN1D4;DCUN1D4	0.00983745	0.601814874191105	0.0722472957206911
**cg14214262**	12	114843188	TBX5;TBX5;TBX5;TBX5	0.00818135	0.449224747774739	0.0722599275543869
**cg27346037**	6	106442129		0.0470046	0.53431781109869	0.0723709909571975
**cg26155979**	10	105110866	PCGF6;PCGF6;PCGF6;PCGF6	0.0486541	0.259386852283159	0.0723709909571975
**cg00606861**	18	19321110	MIB1	0.0172324	0.691179174274416	0.0723709909571975
**cg00776427**	4	85258738		-0.0705519	-0.130777514129209	0.0724258073641366
**cg00531182**	17	49412314	LOC101927274	-0.0704233	-0.130509853965674	0.072603219095295
**cg08220120**	5	138727711	LOC389333;LOC389333	0.164059	0.617676621258641	0.0726408587661753
**cg11803801**	5	172299559	ERGIC1	-0.0216083	-0.0335866521416338	0.0726408587661753
**cg21153648**	16	30394785	SEPT1;ZNF48;ZNF48	-0.0349613	-0.0572250490404156	0.0726408587661753
**cg04548722**	16	4844753	LOC440335;LOC440335;LOC440335	-0.0467562	-0.106303315692303	0.0726408587661753
**cg06013872**	4	72203823	SLC4A4;SLC4A4;SLC4A4	-0.062822	-0.107681116817411	0.0726408587661753
**cg15724937**	17	17598080	RAI1	0.0195288	0.619566904548184	0.0726408587661753
**cg10038250**	14	104561218	ASPG	0.033862	0.79191252361856	0.0726408587661753
**cg13134407**	3	176535538	LINC01209	-0.0390411	-0.0630375724335979	0.0726408587661753
**cg07065803**	11	45921557	MAPK8IP1	0.040907	0.334602098179751	0.0726408587661753
**cg03995399**	2	217741840		-0.0623666	-0.117706516042441	0.0726408587661753
**cg10620501**	8	37620046	PROSC	0.0401712	0.887029607263204	0.0726408587661753
**cg27559630**	5	134241181	PCBD2	0.00480532	0.598182742935561	0.0726408587661753
**cg05037488**	6	33395495	SYNGAP1	0.0361048	0.341443667969028	0.0726408587661753
**cg17211077**	3	151647132		-0.0663492	-0.13656081685363	0.0726408587661753
**cg01061025**	1	212209225	INTS7;DTL;DTL	0.00969844	0.552401409586898	0.0726408587661753
**cg10440681**	16	89939442	TCF25	0.0163788	0.766479180544143	0.0726408587661753
**cg24382801**	6	37665291	MDGA1;MDGA1	0.028632	0.918932369946077	0.0726408587661753
**cg15340431**	15	86040528	AKAP13;AKAP13	0.00749879	0.0113330054164108	0.0726408587661753
**cg26642667**	7	127292389	SND1;SND1	0.0642768	0.770760404608225	0.0726408587661753
**cg05771398**	17	66031830	KPNA2	0.00558922	0.562098998626833	0.0726408587661753
**cg01407202**	17	78194138	SLC26A11;SLC26A11;SLC26A11;SLC26A11;SGSH;SGSH	0.0272117	0.690909485403958	0.0726408587661753
**cg13680864**	10	17686119	STAM	0.0192277	0.582556090018448	0.0726409387642839
**cg16771716**	11	65667296	FOSL1	0.0122539	0.549248438006756	0.0726409387642839
**cg22425802**	12	65174396		0.0119994	0.694438021369895	0.0726409387642839
**cg25102782**	2	179316089	PRKRA;DFNB59;PRKRA;PRKRA;MIR548N	0.00738351	0.462932432627295	0.0726409387642839
**cg15443043**	6	87922248	ZNF292	-0.0259896	-0.0410801533397932	0.0726409387642839
**cg09532284**	4	16084247	PROM1;PROM1	0.0270742	0.63622010332054	0.0726409387642839
**cg19796514**	17	45266588	CDC27;CDC27;CDC27;CDC27;CDC27;CDC27;CDC27	0.0232084	0.411885334083663	0.0726409387642839
**cg00395990**	11	119054921	PDZD3;PDZD3	-0.0773133	-0.141819937679917	0.0726409387642839
**cg16306898**	1	1475675	C1orf70	0.0125376	1.0453823335651	0.0726409387642839
**cg11401820**	15	82425284	EFTUD1;EFTUD1	-0.00909232	-0.0136571929888501	0.0726409387642839
**cg00763768**	14	99947408	SETD3;CCNK;CCNK;SETD3	0.006442	0.753953364584004	0.0726409387642839
**cg16656180**	10	97912335	ZNF518A;ZNF518A;ZNF518A;ZNF518A	-0.0437732	-0.0810391700567013	0.0726409387642839
**cg11302666**	17	41564564	DHX8;DHX8	-0.0832169	-0.149208660346575	0.0726409387642839
**cg21354059**	1	143272904		-0.0541533	-0.112453071551005	0.0726409387642839
**cg09552297**	3	186158082		-0.0382898	-0.059938558532617	0.0726409387642839
**cg09724271**	2	144969767	GTDC1;GTDC1;GTDC1;GTDC1;GTDC1;GTDC1;GTDC1	-0.0641099	-0.10772695781881	0.0726409387642839
**cg16327326**	16	1662895	IFT140	0.0475988	1.00070472026429	0.0726409387642839
**cg08740524**	12	54520017	LOC400043	0.0155274	0.55553380347812	0.0726409387642839
**cg26778477**	19	46295880	DMWD	0.0260285	0.798919928136497	0.0726409387642839
**cg00914033**	10	81664698		0.0279013	0.617609592593727	0.0726409387642839
**cg22751046**	5	125799448	GRAMD3;GRAMD3;GRAMD3;GRAMD3;GRAMD3	-0.0749698	-0.132880320928189	0.0726498340916917
**cg12526923**	19	17439208	ANO8	0.0305716	0.479911267603461	0.0726498340916917
**cg04202122**	2	29116540	WDR43	-0.0425322	-0.0687868079258978	0.0726498340916917
**cg04391610**	2	109223537	LIMS1;LIMS1;LIMS1;LIMS1	-0.0656413	-0.118583097126316	0.0726574627309568
**cg23319790**	19	54215487	MIR519D;MIR517A	-0.0523341	-0.0871394460908687	0.0726574627309568
**cg10478879**	12	31814672	METTL20;METTL20;METTL20	-0.0507817	-0.088198341823183	0.0726574627309568
**cg06517913**	3	121724385	ILDR1;ILDR1;ILDR1	-0.0455824	-0.0816950595412211	0.0726574627309568
**cg14056065**	6	31562236	NCR3;NCR3;NCR3	-0.0611499	-0.0985279916756513	0.0726574627309568
**cg23691516**	1	9661251	TMEM201;TMEM201	-0.00579025	-0.00848978925940311	0.0726574627309568
**cg09136101**	17	4403322	SPNS2	0.0056967	0.295606234283089	0.0726574627309568
**cg25494966**	17	56912681	PPM1E;PPM1E	-0.0442668	-0.0724311443420408	0.0726595614683484
**cg21963178**	10	75571738	NDST2	0.0509128	0.488391892692551	0.0728512394755248
**cg06874758**	1	29508727	SFRS4	0.0103737	0.46707061543123	0.0728512394755248
**cg03501393**	16	69567173		-0.0677745	-0.133616555990246	0.0728512394755248
**cg24939483**	10	31360234		-0.0108995	-0.016327892240982	0.0728512394755248
**cg18895189**	3	172166270	GHSR;GHSR	0.052657	0.486229291459461	0.0728512394755248
**cg02571579**	2	85361441	TCF7L1;TCF7L1	0.0371092	0.708656218422029	0.0728512394755248
**cg08262876**	10	102757026	LZTS2	-0.066368	-0.47835147992966	0.0728512394755248
**cg06727060**	16	30709711	SRCAP	0.0374531	0.391019777182221	0.0728512394755248
**cg04609307**	1	8378356		0.0163201	0.436642302575704	0.0728512394755248
**cg02240671**	3	52149713	POC1A;POC1A;POC1A	-0.0796943	-0.179426147969523	0.0728512394755248
**cg24706124**	1	154173755	C1orf189	-0.0728285	-0.125458003859134	0.0728512394755248
**cg07614980**	6	169744149		-0.0636589	-0.123222628311418	0.0728512394755248
**cg01515806**	1	36235799	CLSPN	0.00875571	0.596418197698342	0.0728512394755248
**cg00044440**	5	138671449		-0.0526135	-0.0824964979007803	0.0728512394755248
**cg20009183**	2	242675096	D2HGDH;D2HGDH;D2HGDH	0.0460371	1.54607507928281	0.0731825828954341

**cg21088281**	4	176922637	GPM6A;GPM6A	0.0111784	0.614874537214264	0.0732545130155182
**cg15399785**	17	17942665	ATPAF2;C17orf39;C17orf39	0.0165913	0.674600619285679	0.073275557879141
**cg15770599**	3	32547795		-0.0291722	-0.0449016258515672	0.073275557879141
**cg17963944**	7	100303258	POP7	0.00861145	0.470644104852912	0.073275557879141
**cg14569576**	9	102585669	NR4A3;NR4A3;NR4A3	-0.002159	-0.243939887523546	0.073275557879141
**cg12786012**	1	12602511		-0.00578504	-0.00852965288731592	0.073275557879141
**cg13943052**	15	40987167	RAD51;RAD51;RAD51;RAD51	0.0173292	0.65896883358755	0.073275557879141
**cg03695166**	10	5506703		-0.0641452	-0.105071671150702	0.073275557879141
**cg16053580**	19	51568863	KLK13	0.0424951	0.350104553926719	0.073275557879141
**cg21137022**	6	52853558	GSTA4	-0.00330473	-0.00483685796856592	0.073275557879141
**cg15918766**	12	53662405	ESPL1	0.00947461	0.450505904913095	0.073275557879141
**cg06789519**	13	112553904		-0.0379296	-0.0617967114434863	0.073275557879141
**cg24950571**	5	10441620	ROPN1L	0.00438426	0.339841760756228	0.073275557879141
**cg16684608**	1	153758749		-0.111914	-0.192213608200201	0.073275557879141
**cg05470082**	5	131832498		0.00870513	0.601049437127525	0.073275557879141
**cg16200007**	2	105946567	TGFBRAP1;TGFBRAP1	0.016813	0.811772131085856	0.073275557879141
**cg24272452**	20	49322897		-0.0429821	-0.0735930270257855	0.073275557879141
**cg17832605**	3	160048980	IFT80;IFT80;IFT80	-0.0238086	-0.0366451770593985	0.073275557879141
**cg16665383**	8	96258496	C8orf37	-0.0967727	-0.181751470190903	0.073275557879141
**cg22715837**	7	113723162		0.0195818	0.525772515948703	0.073275557879141
**cg01531665**	9	33473681	NOL6;SUGT1P1;NOL6	0.00642468	0.454611960324654	0.073275557879141
**cg26971783**	15	77857997		0.0133356	0.0201576845896764	0.0732810414830824
**cg17120588**	5	159911089	MIR146A	-0.0407049	-0.0665266563146791	0.0732810414830824
**cg27084712**	3	42977845		-0.0651541	-0.117182202032663	0.0733074210941224
**cg17611765**	17	46703945	HOXB9	0.0132047	0.762227948802435	0.0733528094360592
**cg04283882**	4	926361	TMEM175;GAK	0.00470444	0.548131803720666	0.0733728185261988
**cg05545777**	5	101119128		0.0916585	0.310083339000172	0.0733728185261988
**cg24098561**	5	175197036		-0.0498247	-0.0869765533745052	0.0733728185261988
**cg24713343**	1	52082692	OSBPL9;OSBPL9;OSBPL9;OSBPL9	0.0333671	0.64784972129524	0.0733824346145668
**cg06058597**	2	10829993	NOL10;NOL10	0.0106776	0.376852434450034	0.0733824346145668
**cg22473973**	10	133794911	BNIP3	0.0118976	0.436833725667046	0.0733824346145668
**cg01616682**	11	15095017	CALCB	0.0420689	0.356224709927024	0.0733824346145668
**cg08714389**	14	81398052	CEP128	-0.0247696	-0.0396098929022929	0.0733824346145668
**cg01349853**	3	36986697	TRANK1	0.0177039	0.847360081601727	0.0733824346145668
**cg17161202**	8	141903388	PTK2;PTK2;PTK2	-0.0721124	-0.163548539544455	0.0733824346145668
**cg22986271**	3	45730975	SACM1L	0.0301621	0.867267816055615	0.0734657106065612
**cg15057214**	11	74699344	NEU3	-0.0718668	-0.140826162963607	0.0734917443124597
**cg20979061**	19	49939949	SLC17A7	0.0365767	0.205181323657187	0.0735585772557059
**cg16078523**	3	50738135	DOCK3	-0.138944	-0.296945654395322	0.0735585772557059
**cg19091715**	1	203562914		-0.0726869	-0.156317004885373	0.0735699070566771
**cg02512267**	5	43120873	ZNF131;ZNF131;ZNF131;ZNF131	0.00857932	0.606880134991808	0.0735699070566771
**cg17564183**	6	28888885	TRIM27	-0.016894	-0.0257586698676802	0.0735699070566771
**cg00472445**	6	101329307	ASCC3;ASCC3	0.0260532	0.654975508720795	0.0735699070566771
**cg08757142**	12	66036823	LOC100507065;LOC100507065;LOC100507065;LOC100507065	-0.0844032	-0.147076018706001	0.0735699070566771
**cg19062489**	19	15662114	CYP4F22	0.0276827	0.68007368842487	0.0735699070566771
**cg14741153**	10	95753348	PLCE1	-0.0265626	-0.243599721873226	0.0735699070566771
**cg04238274**	7	53879377		-0.040327	-0.063807678438462	0.0735699070566771
**cg15502903**	22	17566297	IL17RA	0.0126083	0.578830995675992	0.0735699070566771
**cg22513166**	1	212476869	PPP2R5A;PPP2R5A	-0.108318	-0.370883902373074	0.0735699070566771
**cg25205329**	19	2329794	LSM7;SPPL2B;SPPL2B	0.010831	0.0169515705720327	0.0735699070566771
**cg17606996**	2	171572472	LINC01124;LOC101926913;SP5	0.0229161	0.569721186371671	0.0735699070566771
**cg15702823**	6	10838819		0.00976837	0.563857060787114	0.0735699070566771
**cg18826743**	17	36900631	PCGF2	-0.0640902	-0.114667050645599	0.0738424746781187
**cg18022777**	2	30144651	ALK	0.0285807	0.9706065462989	0.0738424746781187
**cg10502508**	14	76449243	TGFB3	0.0122813	0.518661722681612	0.0738424746781187
**cg00349599**	17	17184369	COPS3	0.00889579	0.521820983130728	0.0738424746781187
**cg13118536**	22	41215559	SLC25A17	0.02125	0.386600328779085	0.0738424746781187
**cg00056074**	2	131485150		0.017004	0.396855358164782	0.0738424746781187
**cg01296482**	15	45798233	SLC30A4	-0.0402978	-0.0646620529428543	0.0738424746781187
**cg04757074**	12	67663012	CAND1	0.0084058	0.571302276420937	0.0738424746781187
**cg26827737**	2	37552543		0.00426063	0.338862080998804	0.0738424746781187
**cg14988142**	1	229643771	NUP133	0.0145632	0.583670809957699	0.0739018872965345
**cg12133425**	1	247494926	ZNF496;ZNF496	0.0232422	0.920229955214448	0.0740119959070531
**cg13742532**	3	42850848	CCBP2	-0.0780698	-0.148101619468772	0.0742315942615147
**cg18036763**	22	45404910	PHF21B;PHF21B	0.0912877	1.23484815522732	0.0743398242742805
**cg21229979**	6	163746175	LOC285796	0.035585	0.427482102334717	0.0743398242742805
**cg07547404**	15	40933758	CASC5;CASC5	-0.0746965	-0.141933045135935	0.0744442726833354
**cg21839284**	1	54359146	DIO1;DIO1;DIO1;DIO1	-0.0893469	-0.175698796351187	0.0744442726833354
**cg04227633**	14	102904606	TECPR2	0.0114708	0.371074591415766	0.0744442726833354
**cg18637847**	3	193788929		0.0141369	0.472777387821475	0.0745809265647803
**cg22511564**	16	46809514		-0.0501296	-0.0858270710166308	0.0745809265647803
**cg14638209**	2	10260280		0.0143344	0.407945460866636	0.0745809265647803
**cg17069349**	3	52311846	WDR82	0.0192973	0.750073381538996	0.0745809265647803
**cg01558923**	1	218520959	TGFB2;TGFB2	0.027688	0.632747607287817	0.0746165544993359
**cg25833238**	6	153304325	FBXO5;FBXO5	0.0157177	0.748996416941287	0.0746165544993359
**cg11116013**	10	134973794	KNDC1	0.0450674	0.56499622849691	0.0746212954715123
**cg14145635**	6	20201350	MBOAT1;MBOAT1	-0.0228471	-0.035354972196629	0.0746212954715123
**cg23058863**	14	24610866	FAM158A	0.0113813	0.676945120390387	0.0746212954715123
**cg02489006**	11	47207577	PACSIN3;PACSIN3;PACSIN3	0.00823268	0.349047386692095	0.0746212954715123
**cg14817448**	3	125069389	ZNF148	-0.0993099	-0.203030240399723	0.0746212954715123
**cg01202012**	22	47248847	TBC1D22A;TBC1D22A;TBC1D22A;TBC1D22A;TBC1D22A	0.0129057	0.0205757380806188	0.0746212954715123
**cg14346123**	10	5237038		-0.042959	-0.0743438320866207	0.0746502722943117

**cg01608030**	19	1605680	UQCR	0.046679	1.00004176324486	0.0746502722943117
**cg00021028**	21	45209230	RRP1	0.0169219	0.557019943499449	0.0747311141696513
**cg07661923**	11	6502690	FXC1;FXC1;ARFIP2	0.0117278	0.987820793141496	0.0747311141696513
**cg24509398**	1	28416532	EYA3	-0.092603	-0.19249210441199	0.0747311141696513
**cg16226020**	17	6839697		-0.0692337	-0.13867306285205	0.0747311141696513
**cg17726566**	1	230943956		-0.038827	-0.0623703888515044	0.0747311141696513
**cg18182148**	1	92952533	GFI1;GFI1	0.052283	0.355967671006249	0.0747311141696513
**cg08115387**	20	709194		0.0332578	1.16599845065827	0.0747311141696513
**cg13155599**	4	120221765	C4orf3;C4orf3;C4orf3	0.0193213	1.16202069037837	0.0747775171995083
**cg10798292**	1	202976329	TMEM183B;TMEM183A	0.0085773	0.418784812208974	0.0747775171995083
**cg05129802**	16	85517587		-0.0117661	-0.017493763916595	0.0747775171995083
**cg26601289**	1	247267644	ZNF669;ZNF669;ZNF669;ZNF669	0.0380937	0.392644403367296	0.0747879026788693
**cg03209127**	1	235668099	B3GALNT2	0.0134892	0.736309083467768	0.074851991693778
**cg23937586**	15	40574925		0.00656436	0.38494546129199	0.0748963656338971
**cg06686789**	8	20041853	SLC18A1;SLC18A1;SLC18A1;SLC18A1	-0.0915215	-0.182635074239918	0.0748963656338971
**cg27423247**	8	123783955		-0.0817403	-0.151035764914505	0.0748963656338971
**cg07406704**	16	23592608	NDUFAB1	-0.0533684	-0.0899121675508751	0.0748963656338971
**cg00875152**	5	42772539	CCDC152	-0.0686298	-0.131324436017358	0.0748963656338971
**cg22717779**	9	125808911	RABGAP1	-0.0324316	-0.0533415546889419	0.0748963656338971
**cg25882056**	22	50311056	ALG12;CRELD2;CRELD2	-0.0506399	-0.0864084701970342	0.0750204302424032
**cg16145701**	1	114472499	HIPK1;HIPK1	0.0312793	0.600384696408301	0.0751757896712282
**cg05183487**	16	89992959	TUBB3	0.0464432	0.989197501884308	0.0751757896712282
**cg05834551**	20	44798891		-0.0426427	-0.0803089588186962	0.0751757896712282
**cg23472502**	6	42420509	TRERF1	0.0166662	0.614939915058011	0.0752543218238762
**cg26860209**	12	15474441	PTPRO;PTPRO	-0.0406823	-0.0680988927270633	0.0752543218238762
**cg27323134**	8	86089330	E2F5;E2F5	0.0541216	0.396376608082612	0.0752543218238762
**cg24069815**	14	45557069	PRPF39	-0.0511107	-0.106292608753548	0.0752543218238762
**cg23124755**	9	33447476	SUGT1P1;AQP3	0.0125481	0.326303172142253	0.0753048559745674
**cg15648832**	5	135322425		-0.0503894	-0.08409368234851	0.0753324135000421
**cg03227570**	1	17766306	RCC2;RCC2	0.0313437	0.35705844331682	0.0753324135000421
**cg04153495**	16	25078095		0.0134672	0.366905926388654	0.0753629505566011
**cg25022925**	5	126112600	LMNB1	0.036541	0.682056397903901	0.0754491539219829
**cg25013978**	5	36149258	MIR580;LMBRD2	-0.0747699	-0.129341780360313	0.0754491539219829
**cg17921886**	15	72109718	NR2E3	-0.042811	-0.0766483945172812	0.075455696829907
**cg02972188**	12	58165945	METTL1;FAM119B;METTL1;FAM119B	0.0171907	0.673646151260807	0.0754590195528433
**cg14630164**	4	153462842		-0.0654917	-0.110648255015754	0.0754891862827452
**cg17977649**	6	46743572		-0.0569407	-0.10562466657776	0.0755130313905189
**cg24341498**	9	137217390	RXRA	0.0457879	1.79958392279548	0.0755547756611606
**cg06089948**	11	70251259	CTTN;CTTN;CTTN	-0.0109737	-0.0163711093929285	0.0755547756611606
**cg01441014**	4	2278810	ZFYVE28	0.0103472	0.0154844777880254	0.0755547756611606
**cg14580115**	11	86141757		-0.0629009	-0.128881569924228	0.0755547756611606
**cg08655589**	3	14444175	SLC6A6;SLC6A6;SLC6A6;SLC6A6;SLC6A6;SLC6A6	0.0724813	1.24087372940102	0.0755547756611606
**cg03103375**	9	128329580	MAPKAP1;MAPKAP1;MAPKAP1;MAPKAP1;MAPKAP1;MAPKAP1	-0.00739827	-0.010899569988482	0.0755547756611606
**cg23374762**	18	54305440	TXNL1;TXNL1	0.0117265	0.520137819815523	0.0755547756611606
**cg26529712**	19	35399253		-0.0476217	-0.139728408951141	0.0755547756611606
**cg20559736**	19	45174671	CEACAM19;CEACAM19	-0.0874128	-0.183321229939336	0.0755547756611606
**cg05892167**	3	79350387	ROBO1	-0.0599864	-0.116699181803275	0.0755547756611606
**cg00582820**	3	125314599	OSBPL11	0.0186084	0.895815683035885	0.0755547756611606
**cg22613880**	17	66453826	WIPI1;PRKAR1A	0.0206473	1.12628026133241	0.0755547756611606
**cg21151769**	20	30946476	ASXL1;ASXL1;ASXL1;ASXL1	0.038882	0.38110765675093	0.0755547756611606
**cg05445097**	10	79627932	DLG5	-0.0812319	-0.137967978327179	0.0755547756611606
**cg14963970**	5	59980902	DEPDC1B;DEPDC1B	0.00552545	0.00818594569004492	0.0755547756611606
**cg25330093**	12	51420190	SLC11A2;SLC11A2;SLC11A2;SLC11A2;SLC11A2;SLC11A2;SLC11A2;SLC11A2	0.01102	0.483213562168003	0.0755547756611606
**cg00461149**	7	157452656	PTPRN2;PTPRN2;PTPRN2	-0.0578309	-0.143418935976074	0.0755547756611606
**cg14392168**	7	99766665	GAL3ST4	-0.0694147	-0.153798785754712	0.0755547756611606
**cg02508567**	2	85361184	TCF7L1	0.0354961	1.00672034441176	0.0755547756611606
**cg00395210**	12	48592465		0.0106175	0.579812838500296	0.0755547756611606
**cg25540028**	13	49001184	LPAR6;LPAR6;RB1	-0.0676404	-0.109336551020308	0.0755547756611606
**cg11856542**	4	37245707	KIAA1239	0.0220564	0.525119156261398	0.0755547756611606
**cg24659836**	9	119882507	ASTN2	-0.0692073	-0.525902588574964	0.0755547756611606
**cg01943813**	6	24495061	ALDH5A1;ALDH5A1	0.0266727	0.448977337083946	0.0755547756611606
**cg25391332**	1	32411791		-0.0198845	-0.0303914529937535	0.0755547756611606
**cg20791669**	3	190232025	IL1RAP;IL1RAP;IL1RAP;IL1RAP;IL1RAP;IL1RAP	0.00937258	0.611313369217183	0.0755547756611606
**cg12769138**	2	168150203		0.00511142	0.405390597143081	0.0755547756611606
**cg11648030**	12	47605172	PCED1B-AS1;PCED1B;PCED1B	-0.0700094	-0.139727270584474	0.0755807973880237
**cg11070274**	8	9106610		-0.097521	-0.605041673956292	0.0755807973880237
**cg10197405**	16	31148406	PRSS8	-0.0608125	-0.134827533351558	0.0755807973880237
**cg03623378**	20	13201670	ISM1	0.0103847	0.617368375631928	0.0755807973880237
**cg14853783**	8	55688267		-0.0138601	-0.0206920356149596	0.0755807973880237
**cg08894050**	16	2273202	E4F1;E4F1;E4F1	0.0183753	0.472716118234695	0.0755807973880237
**cg14131755**	10	50606043		0.0229133	0.624464607443508	0.0755807973880237
**cg25312481**	1	10459146	PGD;PGD	0.0318655	0.663796706712831	0.0755807973880237
**cg01694698**	10	96312660	HELLS;HELLS;HELLS;HELLS;HELLS;HELLS;HELLS;HELLS;HELLS;HELLS	-0.0294743	-0.0458346466627985	0.0755807973880237
**cg07658503**	7	30722177	CRHR2	0.00646038	0.285737950739999	0.0755807973880237
**cg05542295**	17	55162876	AKAP1	0.0188469	0.597571569108361	0.0755807973880237
**cg16257334**	6	30881842	GTF2H4;VARS2;VARS2;VARS2	0.0219256	0.6990931956126	0.0755861310347516
**cg03182917**	2	176944601		0.033085	0.42031637172223	0.0755992312357927
**cg26687726**	19	47220339	PRKD2;PRKD2;PRKD2;PRKD2;PRKD2;PRKD2	0.00506621	0.405075676912934	0.0756530220924628
**cg17880703**	19	2900938	ZNF57;ZNF57	0.0103379	0.67166298596279	0.0756530220924628
**cg27618671**	17	19483043		0.0459308	0.433618471022609	0.0756530220924628
**cg21912093**	14	73704842	PAPLN	0.0322462	0.753852509377461	0.0756530220924628
**cg18151270**	13	53401282		-0.0198344	-0.0308115184476529	0.0756885304971628

**cg03237412**	15	44069622	ELL3	0.0262035	0.674874380979218	0.0756885304971628
**cg00952573**	6	18210046	KDM1B	-0.0918092	-0.207831174137925	0.0756885304971628
**cg05802477**	12	132629385	DDX51;NOC4L	0.010836	0.635672609611886	0.0757385158533335
**cg17943520**	21	40817850	MIR6508;SH3BGR;SH3BGR	0.018551	0.488564710205008	0.0757556832333133
**cg14572091**	9	136537981	SARDH;SARDH	-0.0175285	-0.0262660344661009	0.0758034296655427
**cg17854641**	19	51126772	SYT3;SYT3;SYT3	-0.0472783	-0.0804008451853602	0.075888486798537
**cg02815282**	11	67141135	LOC100130987;CLCF1;CLCF1;CLCF1	0.0178197	1.43328264323462	0.075888486798537
**cg00282877**	10	135265980	LOC619207	-0.0642984	-0.121002811994715	0.075888486798537
**cg02598978**	16	20817844	LOC81691;ERI2;LOC81691;LOC81691;ERI2;LOC81691	0.0260396	0.690361716953546	0.075888486798537
**cg15470309**	16	80905554	PRCAT47;PRCAT47	-0.0444787	-0.0793091374810189	0.075888486798537
**cg23968650**	6	114384629	HS3ST5	-0.0757027	-0.151206897026386	0.075888486798537
**cg13022888**	14	82000246	SEL1L	0.0294086	0.411604347564561	0.075888486798537
**cg25597760**	17	77766120		0.021415	0.375460438139725	0.075888486798537
**cg19934703**	1	17332367	ATP13A2;ATP13A2;ATP13A2	-0.06279	-0.117710129042877	0.075888486798537
**cg05237745**	3	187431255	LOC100131635	-0.0244657	-0.037643014706826	0.075888486798537
**cg23095642**	1	52081938	OSBPL9;OSBPL9;OSBPL9;OSBPL9;OSBPL9	-0.0537599	-0.0854291092323883	0.075888486798537
**cg01008057**	7	79764881	GNAI1;GNAI1	0.0208374	0.760766262367257	0.075888486798537
**cg14864519**	15	38991650	C15orf53	0.00617138	0.00911201604636388	0.075888486798537
**cg27001137**	13	113455344	ATP11A;ATP11A	-0.0497961	-0.0938665071382011	0.075888486798537
**cg18075930**	7	30615805		-0.0519094	-0.0948502241221929	0.075888486798537
**cg06338016**	3	196218811	RNF168	0.00941793	0.0142164023815485	0.075888486798537
**cg24964061**	15	35264425		-0.0112683	-0.0168696931634793	0.075888486798537
**cg08531864**	20	35841852	RPN2;RPN2	-0.0420355	-0.0678725609763426	0.075888486798537
**cg03183872**	20	3140552	FASTKD5;UBOX5;UBOX5	0.0482393	1.10409239918456	0.075888486798537
**cg11438280**	2	219586076	TTLL4	-0.0572163	-0.0964238097927752	0.075888486798537
**cg12155930**	12	49744981	DNAJC22;DNAJC22	-0.0216953	-0.0330603608256133	0.075888486798537
**cg23302636**	14	55281026		-0.0402833	-0.0786929334414996	0.075888486798537
**cg00999623**	1	108743186	SLC25A24	0.0349881	0.845370993830586	0.075888486798537
**cg15543284**	2	54785307	SPTBN1;SPTBN1	0.0418953	0.726698194295663	0.075888486798537
**cg20105087**	20	44542136	PLTP;PLTP	-0.0742497	-0.157313102456712	0.075888486798537
**cg09597829**	5	37575993	WDR70	-0.0952249	-0.174137904514931	0.075888486798537
**cg08819256**	2	129109442		-0.0470773	-0.0914230053923309	0.075888486798537
**cg22397944**	11	237014	PSMD13;PSMD13;SIRT3;PSMD13;PSMD13;SIRT3	0.0455423	0.572372983232388	0.075888486798537
**cg23492789**	2	72520940	EXOC6B	-0.0869898	-0.148788696811393	0.075888486798537
**cg06930255**	3	66344474	SLC25A26;SLC25A26;SLC25A26	-0.067236	-0.164163339872024	0.075888486798537
**cg18429863**	8	73449684	KCNB2;KCNB2	0.0152946	0.622081063322024	0.075888486798537
**cg26869617**	16	2111094	TSC2;TSC2;TSC2	0.0115196	0.0179366250626754	0.075888486798537
**cg11415852**	10	97201765	SORBS1;SORBS1;SORBS1;SORBS1;SORBS1;SORBS1;SORBS1	-0.041328	-0.0638874871852503	0.075888486798537
**cg25437757**	8	42625222	CHRNA6;CHRNA6	-0.0790434	-0.159418861657665	0.075888486798537
**cg04767484**	15	76629104	ISL2	0.0162562	0.457612527498189	0.075888486798537
**cg00997411**	1	205290916	NUAK2	0.0279482	0.818731570439229	0.075888486798537
**cg10399696**	17	80231560	CSNK1D;CSNK1D;CSNK1D;CSNK1D	0.014044	0.666091387263358	0.075888486798537
**cg08214808**	11	45922166	MAPK8IP1	0.0732335	0.531885409446455	0.075888486798537
**cg09266901**	12	124118336	EIF2B1;GTF2H3	0.0183754	0.484587810445005	0.075888486798537
**cg09097421**	1	22815899	ZBTB40;ZBTB40	0.0170758	0.0272517014672751	0.075888486798537
**cg05153861**	7	100492700	ACHE;ACHE	0.00625019	0.374105597606181	0.075888486798537
**cg13828397**	11	121477655	SORL1	-0.0100293	-0.0151373233804783	0.075888486798537
**cg03329500**	22	44205978	EFCAB6;EFCAB6	-0.037208	-0.0614065473468633	0.075888486798537
**cg07886529**	2	232571463		0.0180906	1.15168441922434	0.075888486798537
**cg17382214**	12	110718450	ATP2A2;ATP2A2	0.00886503	0.506962557480052	0.075888486798537
**cg16257101**	14	58863000	TOMM20L	0.0274577	0.496466964977716	0.075888486798537
**cg25938596**	8	104240157	BAALC;BAALC	-0.0350689	-0.0558100863946674	0.075888486798537
**cg07229135**	19	28204254		-0.0760324	-0.147098519101631	0.0758953027078097
**cg10093972**	3	10436176	MIR885;ATP2B2;ATP2B2	-0.0547184	-0.0999564476108598	0.0759848099952965
**cg25996141**	3	52211370		0.0102592	0.0156466862734694	0.0759848099952965
**cg04887335**	3	196044632	TCTEX1D2	0.0523902	1.12780875352805	0.0761074931379137
**cg12632487**	1	6786300	LOC100505887;LOC100505887	0.0694131	0.153704821572688	0.0761074931379137
**cg13604132**	4	75023629	MTHFD2L	0.0288134	0.665688359649744	0.0761817626240994
**cg06567972**	6	7643349		-0.0558385	-0.116273130293779	0.0764247335283911
**cg19014302**	19	18303893	MPV17L2	0.017302	1.143957215761	0.0764867033301317
**cg02899788**	1	47133675	ATPAF1;ATPAF1	0.0339228	0.484967994173029	0.0764888070110629
**cg19135230**	8	103136319	NCALD;NCALD;NCALD;NCALD;NCALD;NCALD;NCALD	0.0131128	0.623402427242037	0.0764888070110629
**cg06319822**	16	215960	HBM	0.0155057	0.702651357092054	0.0764888070110629
**cg19926434**	14	66975296	GPHN;GPHN	0.0123239	0.709812483943524	0.0764888070110629
**cg13756059**	17	40428538	STAT5B	0.0150642	0.478647447556369	0.0764888070110629
**cg05207611**	12	133333251	ANKLE2	-0.0459728	-0.0761925253429043	0.0764888070110629
**cg12590791**	6	89931408	GABRR1	-0.0648996	-0.119662774608763	0.0764888070110629
**cg04034539**	11	62439802	C11orf48;C11orf83	0.0205209	0.707015026814927	0.0768853325894975
**cg00126575**	10	25239164	PRTFDC1;PRTFDC1	-0.0391634	-0.0612718411603119	0.0768940654202772
**cg08555160**	16	69166339	CHTF8;CHTF8;CIRH1A;CHTF8;CHTF8	0.0330375	0.485129516499155	0.0769246609613108
**cg09193923**	2	120517123	PTPN4	0.00522305	0.53368522790142	0.0770055732749119
**cg00936689**	9	134465556	RAPGEF1;RAPGEF1;RAPGEF1	-0.0333449	-0.0556013245465492	0.0770055732749119
**cg02944076**	1	201778100	NAV1;NAV1	-0.0123592	-0.0184253496280966	0.0770055732749119
**cg26279152**	13	20356101	PSPC1;PSPC1	0.0105757	0.873291414959141	0.0770055732749119
**cg08800530**	5	175788722	KIAA1191;KIAA1191;KIAA1191;KIAA1191;KIAA1191;KIAA1191	0.0428534	1.30327385725631	0.0770055732749119
**cg24800930**	14	24454176	DHRS4L2;DHRS4L2;DHRS4L2	-0.026334	-0.0406380690466567	0.0770055732749119
**cg15587693**	2	239228886	TRAF3IP1;TRAF3IP1	0.0235185	0.781826222719076	0.0770055732749119
**cg21308809**	17	61614347	KCNH6;KCNH6	-0.0542427	-0.091125023885363	0.0770055732749119
**cg03974764**	1	117009075	LOC101929023;LOC101929023	0.0181069	0.0291002295479343	0.0770055732749119
**cg01746873**	13	113344027	ATP11A;ATP11A	0.0239557	0.750188451763481	0.0770055732749119
**cg04018214**	12	91572199	DCN;DCN;DCN;DCN;DCN;DCN	-0.0160624	-0.02416269148264	0.0770055732749119
**cg08114812**	1	41445105	CTPS;CTPS	0.0170671	0.513866830574932	0.0770055732749119

**cg05643051**	21	44527804	U2AF1;U2AF1;U2AF1	0.0287102	0.571914429003568	0.0770055732749119
**cg06890950**	4	81382711	C4orf22	-0.0610376	-0.12195559197837	0.0770055732749119
**cg09580409**	4	183065519	MGC45800	0.0461238	0.419984667521932	0.0770055732749119
**cg00127353**	6	112210969		0.00402094	0.00589907695292168	0.0770055732749119
**cg18610261**	2	37899776	CDC42EP3	0.0142557	0.447260325294555	0.0770142185851406
**cg27597069**	6	37321822	RNF8;RNF8;RNF8;RNF8	0.0316649	0.925438729740042	0.0770227125661909
**cg03062417**	8	67868321	TCF24	-0.0655636	-0.13034872433949	0.0770227125661909
**cg11610739**	17	40075306	ACLY;ACLY	0.00778149	0.700042606292204	0.0770227125661909
**cg21843128**	16	68678687	CDH3;CDH3	0.0199446	0.653394040194136	0.077026756825532
**cg16466335**	1	63833149	ALG6	0.0188914	0.690282276550466	0.077026756825532
**cg21776419**	5	81118892		-0.0513556	-0.112652699367612	0.077026756825532
**cg02750935**	5	145214981	PRELID2;PRELID2;PRELID2	0.0133849	0.575080878160511	0.0770562797754422
**cg14707886**	3	98577791	DCBLD2	-0.0429132	-0.0699856645251101	0.0770581122415928
**cg16430332**	2	61389173	C2orf74;C2orf74	0.0132615	0.0203199499364874	0.0771642703234815
**cg03738868**	7	100281714	GIGYF1	-0.0111004	-0.0164559385831764	0.0772004263667292
**cg06105778**	5	176830667	F12	0.0107909	0.740659466061409	0.0772004263667292
**cg20053381**	2	220363572	GMPPA;GMPPA	0.0184216	0.881978597748728	0.0773401707744442
**cg04434653**	18	33767451	MOCOS	0.0350972	0.640011381850165	0.0774574731309749
**cg17937222**	12	122018562	KDM2B;KDM2B	0.0193272	0.709474067259993	0.0776658803867226
**cg13101398**	20	52824678	PFDN4	0.0177348	0.567780754706201	0.0776763358570821
**cg01274995**	1	165472019	LOC400794	-0.0545205	-0.10022877076769	0.0777016245513136
**cg00869249**	10	977800	LOC101927762;LARP4B	0.0432619	0.650184567677418	0.0777055549405616
**cg23834228**	11	73358657	PLEKHB1;PLEKHB1;PLEKHB1;PLEKHB1;PLEKHB1;PLEKHB1;PLEKHB1	0.010479	0.470217542789991	0.0778333043404734
**cg02874321**	11	94166952	MRE11A;MRE11A	-0.082463	-0.163455340521004	0.0778333043404734
**cg26533397**	19	52795316	ZNF766	-0.0488359	-0.0849739997187733	0.0778681764554711
**cg08528519**	14	100659610		0.0140224	0.39574331500791	0.0778681764554711
**cg02096656**	7	139025181	C7orf55	0.0332052	0.457200787665835	0.0778681764554711
**cg00210204**	6	10748101	TMEM14B;TMEM14B;TMEM14B;TMEM14B	0.01251	0.793406605089158	0.0778681764554711
**cg12244205**	6	28972937	ZNF311;ZNF311	-0.0690689	-0.157027140339232	0.0778681764554711
**cg17745339**	5	176755199		-0.0573554	-0.10862509194907	0.0778681764554711
**cg02587124**	6	34626797	C6orf106;C6orf106	-0.0413863	-0.0673474763606805	0.0778681764554711
**cg23961333**	1	55516078	PCSK9;PCSK9	0.0131578	0.0198011441040823	0.0778681764554711
**cg09986736**	11	10472465	AMPD3;AMPD3	0.0336545	0.407408384366914	0.0778681764554711
**cg13884288**	22	38004832	GGA1;GGA1;GGA1;GGA1;GGA1;GGA1;GGA1	0.0170738	0.681256806316826	0.0778681764554711
**cg07990044**	5	77590720	AP3B1;AP3B1	0.0107717	0.591650936487104	0.0778681764554711
**cg11640907**	14	91757787	CCDC88C	0.00724173	0.0108226435116735	0.0779211342335577
**cg25133212**	6	31928014	RDBP;SKIV2L	-0.0380219	-0.0610434541103606	0.0779373069687235
**cg02807377**	4	141567436	TBC1D9	-0.0658051	-0.131476475256683	0.0779373069687235
**cg05817661**	11	93517837	MED17	0.00588516	0.361937289822334	0.0779373069687235
**cg19755108**	5	176434079	UIMC1	0.0729241	0.179335187632181	0.077955282178589
**cg17129645**	2	26395833	FAM59B	0.01818	0.682580824334352	0.077955282178589
**cg13036971**	20	31319259	COMMD7;COMMD7	-0.0283238	-0.0440287251716169	0.077955282178589
**cg06623486**	1	162531613	UAP1	0.0261192	0.61842826197214	0.077955282178589
**cg21387238**	1	155829263	SYT11	0.0325426	1.14841513998409	0.077955282178589
**cg14527983**	20	44562104	PCIF1	0.00214782	0.0031369797974909	0.077955282178589
**cg11022537**	4	177738806		-0.0537389	-0.0871647564100362	0.077955282178589
**cg06886078**	10	126480477	METTL10	0.0107832	0.545117766650757	0.0779679144360258
**cg20553782**	10	104403435	TRIM8	0.0109447	0.659825303650099	0.0779679144360258
**cg10987882**	20	3996459	RNF24;RNF24;RNF24	0.0351098	0.245131037464273	0.0779679144360258
**cg01011432**	20	60982814	CABLES2	0.0149063	0.589786825061271	0.0779679144360258
**cg06027057**	14	90849959		0.0170903	0.525278452671243	0.0779679144360258
**cg18272878**	9	128510407	PBX3;PBX3;PBX3;PBX3	0.021442	0.664828786431924	0.0779679144360258
**cg25007781**	14	105963610	C14orf80;C14orf80;C14orf80	0.00485859	0.00711104432922571	0.0779679144360258
**cg12364031**	7	20692147	ABCB5;ABCB5;ABCB5;ABCB5	-0.0593781	-0.103137870396698	0.0779679144360258
**cg00966077**	16	1401335	C16orf42;GNPTG	0.0227214	0.844605630917246	0.0779679144360258
**cg24809382**	15	55582033	RAB27A	0.0122196	0.873167635448733	0.0779679144360258
**cg26612088**	17	74722779	JMJD6;C17orf95;JMJD6;JMJD6;JMJD6	0.0318187	0.968890282740137	0.0779679144360258
**cg04972341**	11	72354329	PDE2A;PDE2A;PDE2A;PDE2A	0.0229062	0.455612158708333	0.0779679144360258
**cg19117777**	5	109024969	MAN2A1	0.0155011	0.44784432568597	0.0779679144360258
**cg17852224**	22	51042761	MAPK8IP2;MAPK8IP2	0.03682	0.246170719149787	0.0779679144360258
**cg05557255**	11	2422385	TSSC4	0.0326657	0.953402478906351	0.0779679144360258
**cg04318006**	8	121823643	SNTB1	0.0194663	0.615689829271994	0.0779679144360258
**cg22827465**	19	3094947	GNA11	0.0285018	0.720802128595582	0.0779679144360258
**cg01555791**	17	1012288	ABR;ABR;ABR	0.0278374	0.667142697975841	0.0779679144360258
**cg11412876**	6	112194702	FYN	0.0427354	0.634835029452607	0.0779679144360258
**cg24470929**	8	98791446	LAPTM4B	-0.0766258	-0.161246681586487	0.0779679144360258
**cg08313638**	10	12110577	DHTKD1	-0.0193658	-0.585636306200596	0.0779679144360258
**cg21808250**	12	133757947	ZNF268;ZNF268;ZNF268;ZNF268;ZNF268;ZNF268;ZNF268;ZNF268;ZNF268	0.0209004	0.364251156560433	0.0779679144360258
**cg08791234**	6	33267781	TAPBP;TAPBP;RGL2;RGL2	0.00719489	0.396597839304682	0.0779679144360258
**cg24182275**	14	51297392	NIN;NIN;NIN;NIN	0.0173344	0.819038685128838	0.0779679144360258
**cg21608489**	19	42637137	POU2F2	0.00677272	0.464202305658414	0.0779679144360258
**cg04878955**	1	38100435	RSPO1;RSPO1	0.0072355	0.331036376155198	0.0779679144360258
**cg23591039**	1	225814239	ENAH;ENAH	-0.0274426	-0.044506755492213	0.0779679144360258
**cg06579481**	7	104621597		-0.110885	-0.206738850457434	0.0779679144360258
**cg25764824**	22	42085083	NHP2L1;C22orf46	0.018311	0.843017946043869	0.0779679144360258
**cg09387970**	11	55433005	OR4C6	-0.0489743	-0.0779050557320132	0.0779679144360258
**cg14557534**	3	27763581	EOMES	0.0140722	0.558906980241942	0.0779679144360258
**cg07941301**	6	42928277	GNMT	0.014665	0.739753596927313	0.0779679144360258
**cg16888080**	5	148676852	AFAP1L1;AFAP1L1	-0.0138306	-0.0207394049665926	0.0779679144360258
**cg24796645**	4	55745557		-0.0696119	-0.120039053501163	0.0779679144360258
**cg09500317**	16	23652388	DCTN5;PALB2	0.0159826	0.523717806247088	0.0779679144360258
**cg17662034**	8	74207518	RDH10;RDH10	0.0376835	1.00461356477721	0.0779679144360258

**cg14489346**	4	81105371	PRDM8	0.0222412	0.439169178251065	0.0779679144360258
**cg04603176**	11	118306465	MLL	0.0103841	0.424446369464991	0.0779679144360258
**cg14181092**	5	122371896	PPIC	0.0128938	0.523142009931298	0.0779679144360258
**cg27569414**	22	21161673	PI4KA	0.0138763	0.0211349851914967	0.0779679144360258
**cg15645079**	20	3140569	FASTKD5;UBOX5;UBOX5	0.01528	0.826047658045068	0.0779679144360258
**cg23783653**	6	27059850		0.0432123	0.753776783978274	0.0779679144360258
**cg01317569**	5	60140824	ELOVL7;ELOVL7	-0.0082492	-0.0122946699637202	0.0779679144360258
**cg20902958**	2	129075203	HS6ST1	0.00397394	0.283614687914955	0.0779679144360258
**cg19513374**	6	28962971	ZNF311	-0.0564522	-0.09630504913515	0.0779679144360258
**cg11365360**	3	184279509	EPHB3	0.0119941	0.595951889677285	0.0779679144360258
**cg22584339**	13	49150952		-0.0381696	-0.0609987147321446	0.0779679144360258
**cg25739700**	17	5393371	MIS12	-0.0549351	-0.0963874478751384	0.0779679144360258
**cg10658459**	5	72747197		0.0116637	0.420211809598551	0.0779679144360258
**cg18017240**	12	128365675	FLJ37505	0.0102307	0.015638590840714	0.0779679144360258
**cg05741182**	16	72127884	DHX38;TXNL4B;TXNL4B;DHX38;TXNL4B	0.00455214	0.326587720679592	0.0779679144360258
**cg24210426**	18	12780803		-0.0975362	-0.19955352622268	0.0779679144360258
**cg05461270**	6	28574608		-0.00831376	-0.179528255978487	0.0779679144360258
**cg23287661**	1	153395100	S100A7A	0.040805	0.389951123418937	0.0779679144360258
**cg13841901**	1	45805975	MUTYH;TOE1;MUTYH;MUTYH;MUTYH;MUTYH;MUTYH;MUTYH;MUTYH;MUTYH	0.0247933	1.10478315963147	0.0779914655351465
**cg02346492**	17	9143174	NTN1	0.0147546	0.522102659221528	0.0781084662956358
**cg22128985**	1	2089384	PRKCZ;PRKCZ;PRKCZ	-0.0187564	-0.0292889922549697	0.0781084662956358
**cg02373061**	3	163021252	LINC01192	-0.0533776	-0.0894034233671449	0.0783140361858285
**cg02552216**	4	184426031	ING2	0.0134721	0.477916985427039	0.0783191788051293
**cg13873127**	2	201828507	ORC2;ORC2	0.00620225	0.388789506707314	0.0783501615562852
**cg12624667**	3	36986555	TRANK1	0.00664564	0.897504376909005	0.0783624933302863
**cg19469297**	7	45151440	TBRG4;TBRG4;TBRG4	0.0145784	0.486289890977333	0.0783906349567055
**cg00163171**	12	31963379		-0.0312553	-0.0498049320890218	0.0783906349567055
**cg08478074**	1	110613397	ALX3	0.00329828	0.288239942757898	0.0783906349567055
**cg07346676**	11	60681596	TMEM109;TMEM109	0.0245078	1.12724917314138	0.0783906349567055
**cg00577109**	19	59074507	MZF1;MZF1;LOC100131691	0.0195351	0.378048930516359	0.0783906349567055
**cg00379528**	12	125350693		-0.0455496	-0.0869801356010696	0.0783906349567055
**cg27563716**	4	184365897	CDKN2AIP;CDKN2AIP	0.0324755	0.55930726941796	0.0783906349567055
**cg00544901**	19	49999417	RPS11	0.0259829	0.775176679739297	0.0783906349567055
**cg06268875**	18	11147385	FAM38B	0.0684062	0.303240895053734	0.0783906349567055
**cg03314080**	2	178129903	NFE2L2;NFE2L2;NFE2L2	0.0188746	0.923118024763533	0.0783906349567055
**cg21445603**	9	21503744	MIR31HG	0.00549546	0.00808899395358362	0.0783906349567055
**cg19136868**	11	560685	RASSF7;RASSF7;RASSF7;C11orf35	0.0361161	0.747827395644983	0.0783906349567055
**cg06221861**	7	100425075	EPHB4;EPHB4	0.0418494	0.381844945246819	0.0783906349567055
**cg09686236**	2	96011915	KCNIP3;KCNIP3	0.0301674	0.769108130078009	0.0783906349567055
**cg26250870**	19	22234980	ZNF257	-0.0483168	-0.0850881194791477	0.0783906349567055
**cg14943002**	11	47256035	DDB2	-0.0462022	-0.0773595725356598	0.0783906349567055
**cg06962746**	22	45016487		0.0142392	0.730572382366572	0.0783906349567055
**cg15540144**	2	99553141	C2orf55	0.0219742	0.341909934264476	0.0783906349567055
**cg24158363**	17	73401717	GRB2;GRB2;GRB2;GRB2	0.0512469	0.427509830198556	0.0783906349567055
**cg26535435**	19	47290586	SLC1A5;SLC1A5	0.0141981	0.586562133146478	0.0783906349567055
**cg24234117**	13	32885661	ZAR1L	0.0552089	0.100895017764388	0.0783906349567055
**cg08774561**	22	37263475	NCF4;NCF4	0.0134685	0.0203459773034113	0.0783906349567055
**cg23473955**	17	27893407	ABHD15	0.00841892	0.669572456469529	0.0783906349567055
**cg01773831**	3	156392830	TIPARP;LOC100287227	0.00654589	0.484485043625883	0.0783906349567055
**cg08267442**	15	91473386	UNC45A	0.0281057	0.658596895777448	0.0783906349567055
**cg25702652**	2	164204628		-0.0357966	-0.0584909010130146	0.0783906349567055
**cg09889194**	4	7661729	SORCS2	-0.0360977	-0.0611009065055891	0.0783906349567055
**cg09708488**	8	21882013	NPM2	0.015439	0.388516918074337	0.0783906349567055
**cg18682873**	2	46769828	RHOQ	0.028759	0.601226587240533	0.078562303147328
**cg08436643**	17	73257657	MRPS7;GGA3;GGA3	-0.0017369	-0.266330491646832	0.078562303147328
**cg01017689**	5	76376266	SNORA47;ZBED3	0.00985278	0.01505544339779	0.078562303147328
**cg20338600**	5	98110229	RGMB	0.0165507	0.495159183081476	0.078562303147328
**cg09985075**	2	86743517	CHMP3;CHMP3;CHMP3;CHMP3;RNF103-CHMP3	-0.0619459	-0.102684744993809	0.078562303147328
**cg14905382**	1	218055074		-0.0172627	-0.025631464608287	0.0785792415349582
**cg10682850**	16	26658168		-0.0294907	-0.0470668538112292	0.0785974049806064
**cg14565835**	1	174368960	RABGAP1L	-0.0497699	-0.093159034020641	0.0785974049806064
**cg03803784**	12	51157550	ATF1	0.0352498	0.94485527628722	0.0786301045888898
**cg02746014**	19	54959839	LENG8-AS1;LENG8	0.010245	0.458255062866538	0.0786388456284924
**cg14863978**	7	150864961	GBX1	0.0360057	0.226410458312565	0.0786388456284924
**cg17844160**	5	53606416	15,00 ARL	0.0280335	0.861369468057776	0.0786388456284924
**cg11980791**	9	131084797	COQ4;COQ4;TRUB2	0.0310773	1.28067330510208	0.0786388456284924
**cg19568834**	20	34252926	CPNE1;CPNE1;RBM12;CPNE1;CPNE1;CPNE1;RBM12;CPNE1;CPNE1	0.026093	1.01820884740127	0.0786388456284924
**cg05191454**	6	42016435	CCND3;CCND3;CCND3;CCND3	0.00974541	0.459266694401555	0.0786388456284924
**cg20955833**	6	33980895		-0.0362687	-0.0561949163204445	0.0786388456284924
**cg13878775**	2	242041740	MTERFD2;MTERFD2;MTERFD2;MTERFD2;MTERFD2	0.0104004	0.394784412922534	0.0786388456284924
**cg26752655**	20	23331282	NXT1	0.0117095	0.651738473525767	0.079005326842492
**cg15881168**	17	28916490	LRRC37BP1	-0.018459	-0.0280238136055751	0.0790678959059095
**cg17354220**	2	83743984		-0.0465061	-0.143617915029186	0.0791485597184903
**cg04956944**	2	112899297	FBLN7;FBLN7	-0.0192477	-0.0291803842412299	0.0791485597184903
**cg24895834**	10	6622425	PRKCQ	0.0323071	0.776644642450465	0.0791485597184903
**cg14651518**	3	50313693	SEMA3B;SEMA3B	0.00719059	0.608810573714006	0.0791485597184903
**cg01495361**	20	31369590	DNMT3B;DNMT3B;DNMT3B;DNMT3B;DNMT3B;DNMT3B	-0.0551239	-0.0887057845279697	0.0791485597184903
**cg24849789**	12	57061323	PTGES3;PTGES3;PTGES3;PTGES3;PTGES3;PTGES3;PTGES3	-0.0634105	-0.109519033668125	0.0791485597184903
**cg11817804**	7	36427191	KIAA0895	-0.046376	-0.0750686209006172	0.0791485597184903
**cg14844341**	5	139089549		0.00889431	0.442368499252963	0.0791485597184903
**cg26717786**	17	79139685	AATK;LOC388428	0.0315497	0.782095282846567	0.0791485597184903
**cg18451256**	2	128284158	IWS1	0.0252153	1.12334150737653	0.0791485597184903

**cg18294715**	7	71211027		-0.0724543	-0.137806837508119	0.0791485597184903
**cg27497049**	8	94712646	FAM92A1	0.0304775	0.612069479495024	0.0793305813717542
**cg27150186**	21	26979856	MRPL39;MRPL39	0.0103836	0.480261893521552	0.0793305813717542
**cg06338429**	13	46689293		-0.0422292	-0.0699082848775485	0.0793305813717542
**cg24131747**	14	68141730	VTI1B	0.032694	1.34855498852687	0.0793305813717542
**cg16882609**	11	695345	TMEM80;TMEM80;TMEM80;TMEM80;DEAF1;DEAF1;DEAF1;DEAF1	0.0137997	0.624265423399774	0.0793305813717542
**cg13792528**	6	28834507		0.011916	0.598955973407073	0.0793305813717542
**cg18845801**	7	16793338	TSPAN13	0.00810593	0.886387062805729	0.0793305813717542
**cg25261191**	7	101847096	CUX1;CUX1;CUX1	-0.0174071	-0.0262482018451987	0.0793305813717542
**cg13956095**	17	81037419	METRNL	0.04387	0.378543919199463	0.0793305813717542
**cg12419067**	3	9975156	IL17RC;CRELD1;CRELD1;CRELD1;IL17RC;IL17RC	0.0240952	0.652072518197849	0.0793378679602681
**cg04044418**	16	89789362	ZNF276;ZNF276	0.0197135	0.613247333218089	0.0793378679602681
**cg06813578**	15	51386254	TNFAIP8L3	0.0114091	0.288831640443127	0.0793378679602681
**cg23151295**	14	76796070		0.0135534	0.400669756877421	0.0793378679602681
**cg02708544**	5	17376892		-0.0585688	-0.101671716157013	0.0793378679602681
**cg18848478**	1	113933423	MAGI3;MAGI3	0.0225022	0.737061475001158	0.0793378679602681
**cg26976187**	5	176944204	DDX41	0.023549	0.761510611972036	0.0793378679602681
**cg00673286**	15	83349613	AP3B2	0.0172086	0.599942477180094	0.0793378679602681
**cg22526051**	8	145743825	RECQL4;LRRC14	0.0153557	0.58626317119619	0.0793378679602681
**cg13128139**	2	27651391	NRBP1	0.00884718	0.550205619301235	0.0793378679602681
**cg16113772**	6	2972824	SERPINB6;SERPINB6;SERPINB6;SERPINB6;SERPINB6;SERPINB6	-0.0610608	-0.116186932854472	0.0793378679602681
**cg05157272**	1	246862256		-0.0576417	-0.109346800744728	0.0793378679602681
**cg22844232**	3	193776047		0.058106	0.761972563805077	0.0793378679602681
**cg00840257**	16	8963031	CARHSP1;CARHSP1	0.0337928	0.99206144599782	0.0793378679602681
**cg08378505**	17	73127297	NT5C	0.0145702	0.773142263271903	0.0793378679602681
**cg14499653**	1	24740357	STPG1;STPG1;STPG1;STPG1	0.0118049	0.408982277919345	0.0793378679602681
**cg09506172**	19	28331274		-0.0296985	-0.0526011745237426	0.0793378679602681
**cg00744495**	9	138710327	CAMSAP1	-0.0191009	-0.02909111436708	0.0793378679602681
**cg21251197**	9	140514016	EHMT1;C9orf37;EHMT1	0.00505812	0.456276090595058	0.0793378679602681
**cg06911002**	2	160408618	BAZ2B;BAZ2B	-0.0712466	-0.129726416744101	0.0793378679602681
**cg05271080**	4	41986520	DCAF4L1;DCAF4L1	-0.0700341	-0.149312913990677	0.0793378679602681
**cg27656398**	12	123451108	ABCB9;ABCB9;ABCB9	0.021718	0.40837785152673	0.0793378679602681
**cg16637556**	19	41104184	LTBP4;LTBP4	0.0303564	0.440208913434411	0.0793378679602681
**cg15781375**	2	27651918	NRBP1	0.0312344	0.510465973413684	0.0793378679602681
**cg11199490**	12	53564794	CSAD;CSAD	-0.0378473	-0.060976337556189	0.0793378679602681
**cg05141005**	1	175070412	TNN	-0.0328492	-0.0651450751307536	0.0793378679602681
**cg12235622**	1	43856473	C1orf84;MED8;MED8	-0.0106569	-0.0159976805234325	0.0793378679602681
**cg04623019**	1	115034522	TRIM33;TRIM33	-0.0131968	-0.0204761410738422	0.0793378679602681
**cg16022195**	10	19000784		-0.0735753	-0.145363688937047	0.0793378679602681
**cg26549029**	12	133286883	PGAM5;PGAM5;PGAM5	0.0115361	0.65710570638353	0.0793378679602681
**cg12757037**	2	113321746	POLR1B;POLR1B;POLR1B;POLR1B;POLR1B;POLR1B;POLR1B	-0.0361837	-0.0560194802195172	0.0793378679602681
**cg02580528**	1	183441376	SMG7;SMG7;SMG7	0.0116277	0.624152827450393	0.0793378679602681
**cg08095532**	15	78831288	PSMA4;PSMA4;PSMA4	0.00909853	0.0135944415104358	0.0793378679602681
**cg27362998**	10	73376966	CDH23;CDH23;CDH23;CDH23;CDH23;CDH23;CDH23;CDH23;CDH23	0.00903256	0.0134199738222831	0.0793378679602681
**cg26715814**	19	7529580	ARHGEF18;ARHGEF18;ARHGEF18;ARHGEF18	0.0105449	0.0161651484299687	0.0793378679602681
**cg13751231**	13	103058039		-0.0292776	-0.0464476736982833	0.0793378679602681
**cg14472484**	1	184517082	C1orf21	-0.00937866	-0.0139105266737349	0.0793378679602681
**cg27530136**	1	156782996	SH2D2A;SH2D2A;SH2D2A;SH2D2A;SH2D2A	-0.0694506	-0.191445670485549	0.0793378679602681
**cg03865418**	9	79057167	GCNT1;GCNT1	-0.0688788	-0.147721198395178	0.079340596170282
**cg06028533**	10	16478910	PTER;PTER	0.0105994	0.431277627310459	0.079340596170282
**cg00164041**	4	89378199	HERC5	0.0053682	0.418421038443845	0.079340596170282
**cg12339478**	20	44573621	PCIF1	-0.0175064	-0.0263402749899082	0.079340596170282
**cg19497734**	4	144257218	GAB1;GAB1	0.0123056	0.553379808552762	0.079340596170282
**cg02495519**	16	20911570	DCUN1D3;LYRM1;LYRM1;LYRM1;LYRM1	0.030625	0.628198069771184	0.079340596170282
**cg27555944**	3	49056208	DALRD3;DALRD3	0.0211143	0.633159292144349	0.079340596170282
**cg06889339**	6	168197625	C6orf123	0.0502204	0.942195408726288	0.079340596170282
**cg17014378**	9	136025100	RALGDS	0.0171257	0.459632711339667	0.079340596170282
**cg06104657**	2	74734831	PCGF1	0.0065611	0.420139998045527	0.079340596170282
**cg14178882**	21	38128940	HLCS;HLCS;HLCS	0.00965807	0.0144612727151965	0.079340596170282
**cg21737173**	9	16870822	BNC2	0.0296259	0.592241409695166	0.0793950033231131
**cg20852378**	4	184680264		-0.0729902	-0.15179966062843	0.0793950033231131
**cg08522087**	5	14871910	ANKH	0.0266289	1.17995422560284	0.0793950033231131
**cg20971970**	12	57632495	NDUFA4L2	0.021934	0.630810487105509	0.0793950033231131
**cg01640456**	4	141075565	MAML3	0.00816077	0.378162475450975	0.0793950033231131
**cg20387272**	8	144640401	GSDMD;GSDMD	0.0128089	0.697234785092243	0.0793950033231131
**cg25584761**	2	16410140		-0.0126409	-0.0188105434373659	0.0793950033231131
**cg18119529**	19	48775014	ZNF114	0.00390244	0.455808110085945	0.0793950033231131
**cg01436700**	2	105953902	C2orf49	0.0179832	0.551161907584893	0.079411595111465
**cg00526953**	16	1535814		0.0939968	0.200601216855317	0.079472069825999
**cg03538717**	3	79068441	ROBO1;ROBO1;ROBO1;ROBO1;ROBO1;ROBO1;ROBO1	0.00908176	0.488227886626017	0.0796531557145924
**cg08781042**	12	48166839	SLC48A1	0.00528021	0.411369295520528	0.0799043081031149
**cg22736789**	7	138561753	KIAA1549;KIAA1549	0.00527919	0.00781634195715019	0.0799043081031149
**cg03648789**	2	38304037	CYP1B1	0.0187318	0.624641321517619	0.080036094059226
**cg16236108**	4	84457579	AGPAT9	0.0194576	0.74111280338375	0.0801095170482245
**cg11288647**	14	95727390	CLMN	-0.038574	-0.0706844092278623	0.0802433827886744
**cg13260280**	8	27063108		-0.0666738	-0.122490451329967	0.0803625254972515
**cg12866551**	10	20019641		-0.018526	-0.0285666727327626	0.0803625254972515
**cg00826610**	9	37800654	DCAF10	0.0213516	0.676964314352443	0.0803625254972515
**cg12528637**	15	40987266	RAD51-AS1;RAD51;RAD51;RAD51;RAD51	0.0282344	0.753248375412799	0.0807297440978179
**cg10904699**	11	123301490		0.0350276	0.65625490039188	0.0807297440978179
**cg04547425**	17	73043127	ATP5H;KCTD2;ATP5H	0.0103649	0.676700136605759	0.0807297440978179
**cg02497785**	7	48494608	ABCA13	0.101222	0.177060050549182	0.0807297440978179

**cg11111696**	10	31320775	ZNF438;ZNF438;ZNF438;ZNF438;ZNF438;ZNF438;ZNF438;ZNF438;ZNF438;ZNF438;ZN	0.0145922	0.547121554289	0.0807297440978179
**cg06002370**	2	228029452	COL4A3;COL4A3;COL4A3;COL4A3;COL4A4	0.00812234	0.348593999600026	0.0807297440978179
**cg15199800**	15	44069791	ELL3	0.0128703	0.339729945902741	0.0807297440978179
**cg05311383**	10	116049324	VWA2	-0.0235569	-0.0356775618324506	0.0807297440978179
**cg09457057**	1	151398452	POGZ;POGZ;POGZ;POGZ;POGZ	-0.0766733	-0.177103350838547	0.0807297440978179
**cg00090767**	14	65569376	MAX;MAX;MAX;MAX;MAX;MAX	0.0234185	0.836797010712398	0.0807297440978179
**cg17270690**	20	48844509		-0.0506302	-0.0839576414467497	0.0807297440978179
**cg25962774**	5	176831364	F12	0.0183294	0.551351335977147	0.0807297440978179
**cg18436249**	20	56299915		-0.0404643	-0.0682236375294134	0.0807297440978179
**cg15871991**	2	10829907	NOL10	0.0107153	0.450143867662026	0.0807297440978179
**cg17389928**	3	37807439	ITGA9	-0.0089631	-0.0133428751729016	0.0807297440978179
**cg10651583**	12	57941069	DCTN2	0.0732371	0.722504022311926	0.0807297440978179
**cg19889626**	8	26047996		0.0464423	0.58583891013954	0.0807297440978179
**cg22226568**	15	41059847	GCHFR	-0.0181989	-0.0288883257112681	0.0807297440978179
**cg11432034**	3	13520913	HDAC11;HDAC11	0.0144753	0.716136100001832	0.0807297440978179
**cg19250027**	7	107642472	LAMB1	0.019126	0.506137934632658	0.0807297440978179
**cg21649051**	1	85514340	MCOLN3	0.0156981	0.435297025204633	0.0807297440978179
**cg08587797**	10	102988852	LBX1;FLJ41350	0.0395545	0.378806211107624	0.0807297440978179
**cg22223655**	18	67872902	RTTN;RTTN	0.0154438	0.965548126805229	0.0807297440978179
**cg03782202**	2	176971820	HOXD11	0.0667328	0.312574821376418	0.0807297440978179
**cg09901100**	7	134867116		-0.048601	-0.0826273717360087	0.0807297440978179
**cg07262328**	11	35160709	CD44;CD44;CD44;CD44;CD44;CD44;CD44;CD44;CD44	0.0321391	0.877732142301723	0.0807297440978179
**cg10308275**	12	93708517	LOC643339	0.00358933	0.0052888079162601	0.0807297440978179
**cg00990358**	1	40996360	ZNF684	-0.045962	-0.0973494023620873	0.0807297440978179
**cg19180542**	10	43047893	ZNF37B	0.00860406	0.675401189011363	0.0807297440978179
**cg20706599**	15	90777202	CIB1;C15orf58;CIB1	0.0338621	0.605020081841173	0.0807297440978179
**cg22376704**	19	4760016		0.0343745	1.09808269554301	0.0807297440978179
**cg09486567**	20	18038510	OVOL2;OVOL2	0.00810057	0.508261423433471	0.0807297440978179
**cg16513467**	10	28822500	WAC;WAC;WAC;WAC;WAC	0.0340968	0.765827786849627	0.0807297440978179
**cg12821203**	22	42149459	MEI1	-0.0482777	-0.0780244508205041	0.0807297440978179
**cg24473265**	12	113584176		-0.0823537	-0.187663934219042	0.0807573062614043
**cg11111835**	2	38529283	ATL2;ATL2;ATL2	-0.0279541	-0.0450602716547609	0.0807573062614043
**cg15924127**	14	91640473	C14orf159;C14orf159;C14orf159;C14orf159;C14orf159;C14orf159;C14orf159	-0.0516287	-0.0819897236547793	0.0807744883761898
**cg17654257**	1	13930520	PDPN;PDPN;PDPN;PDPN	-0.0437226	-0.104715059871599	0.0807744883761898
**cg07076175**	8	53478053	FAM150A	0.049098	0.318409075153631	0.0807744883761898
**cg21878902**	19	13068309	GADD45GIP1	0.0658724	0.654663612230039	0.0807744883761898
**cg07665510**	17	55952063	CUEDC1	0.0166566	0.53561745934673	0.0808095206902759
**cg07730946**	4	185743727	ACSL1	-0.0322287	-0.0510900432927536	0.0808095206902759
**cg19509894**	19	8070923	ELAVL1	0.0162454	0.741852770212649	0.0808600725575999
**cg25423888**	10	102286179	NDUFB8	0.0215164	0.0341569815911762	0.0808600725575999
**cg19761211**	16	70415365	ST3GAL2	0.0326032	1.51039827728758	0.0808600725575999
**cg18384926**	15	45722598	C15orf48;C15orf48	0.0279215	0.449359531402572	0.0808600725575999
**cg00275645**	21	38640049	DSCR3	0.0165733	0.708830462962549	0.0808600725575999
**cg14743352**	8	145734503	MFSD3	0.0230658	0.707598796017934	0.0808600725575999
**cg05165559**	20	62037758	KCNQ2;KCNQ2;KCNQ2;KCNQ2	-0.0193474	-0.0293099477567465	0.0808600725575999
**cg09948076**	15	99645088	SYNM;SYNM	-0.00405804	-0.30562325874844	0.0808600725575999
**cg03251249**	10	69645012	SIRT1;SIRT1;SIRT1	0.00819729	0.498237233511229	0.0808600725575999
**cg04878000**	1	32237923		0.0359974	0.72484106483811	0.0808600725575999
**cg22894518**	16	17837987		-0.0486384	-0.084046350221475	0.0808600725575999
**cg11688874**	10	28822482	WAC;WAC;WAC;WAC;WAC	0.0165637	0.600034186689835	0.0808600725575999
**cg07105333**	17	27277048	PHF12;PHF12	0.0097803	0.62845604912639	0.0808600725575999
**cg02562419**	6	159049626	TMEM181	-0.0526098	-0.0889098218624297	0.0808600725575999
**cg08678514**	20	61340340	NTSR1;NTSR1	0.037944	0.561910574803249	0.0808600725575999
**cg17921248**	17	64298993	PRKCA	0.0178095	0.716700831933378	0.0808600725575999
**cg14400631**	11	14666512	PDE3B;PSMA1	0.0111851	0.50527358171658	0.0808600725575999
**cg19604738**	6	170599678	DLL1;DLL1;FAM120B	0.0132733	0.927726922926769	0.0808600725575999
**cg10060589**	22	41116056		0.00554444	0.00817193262351322	0.0808600725575999
**cg24167897**	15	63796719	USP3	0.0158307	0.732046663942437	0.0808600916942534
**cg01998014**	16	81545502	CMIP;CMIP	-0.0732678	-0.148193638616952	0.080930998598066
**cg04603954**	4	163786477		-0.0335568	-0.0518881628710964	0.080930998598066
**cg24369983**	1	78448632		-0.0179422	-0.0276729855604056	0.0810286807936586
**cg21041620**	12	49076286	KANSL2	0.00807299	0.452256469871956	0.0810286807936586
**cg06424550**	10	12808422	CAMK1D;CAMK1D	-0.0423719	-0.0701926976658863	0.0810748459393256
**cg27125093**	19	3179828	S1PR4	0.0247788	0.770330482791338	0.0811525274541725
**cg15675646**	13	111669202		-0.0335447	-0.053244676367654	0.0811525274541725
**cg21901807**	2	96987482		0.0111985	0.549930467954317	0.0811525274541725
**cg24456235**	5	52152755	ITGA1	-0.0433503	-0.0703485948198396	0.0811525274541725
**cg20448053**	17	8192352	SLC25A35;RANGRF	0.00755087	0.672862872083404	0.0811525274541725
**cg26152888**	4	48343623	SLAIN2;SLAIN2	0.00726317	0.418198530246417	0.0811525274541725
**cg05533552**	11	34074141	CAPRIN1;CAPRIN1	0.0324472	0.815792218206255	0.0811525274541725
**cg16762000**	1	44911569	RNF220	-0.0620456	-0.110657954453324	0.0811525274541725
**cg03470837**	9	101251353	GABBR2	-0.0876102	-0.174517153207053	0.0811525274541725
**cg26128898**	15	68315767		-0.083202	-0.157894032347734	0.0811525274541725
**cg18096290**	6	131070434		-0.0381378	-0.0609532908589994	0.0811525274541725
**cg25873335**	4	52709051	DCUN1D4;DCUN1D4;DCUN1D4;DCUN1D4	0.0200882	0.724838279375315	0.0811525274541725
**cg21119962**	1	246670217	SMYD3	0.00660116	0.499798790872504	0.0811525274541725
**cg05236418**	5	177581052	NHP2;NHP2	0.0114239	0.557272949282854	0.0811525274541725
**cg24513433**	18	47088234	LIPG	0.0413287	0.396800634419973	0.0811525274541725
**cg13460980**	2	55746328	CCDC104	0.0631446	0.384895259385792	0.0812232057403117
**cg20288129**	3	122745632	SEMA5B	0.0357767	0.887709124070612	0.0812232057403117
**cg20629986**	3	69639223		-0.046738	-0.0994687313027338	0.0812486345695656
**cg06019865**	20	30795840	PLAGL2;POFUT1;POFUT1	0.011518	0.447973455387817	0.081281342815783

**cg01390564**	16	31162282	PRSS36	-0.0565979	-0.156914949837984	0.081281342815783
**cg20199354**	16	3070259	TNFRSF12A	0.0354783	0.296197551185417	0.081281342815783
**cg20882291**	8	97506160	SDC2;SDC2	0.0364115	1.17682668254829	0.081281342815783
**cg04240504**	6	109330951	SESN1;SESN1;SESN1	0.0287033	0.574879525292247	0.081281342815783
**cg05731183**	15	68132796		0.00538438	0.476262489679977	0.0813644945458804
**cg03617693**	3	136751559		0.0177865	0.515901336677896	0.0813644945458804
**cg11525941**	5	87971950	LOC645323;LOC645323	0.026874	0.6118199705262	0.0813644945458804
**cg27408262**	10	126706831	CTBP2;CTBP2;CTBP2	-0.0761446	-0.145586659332559	0.0814415238184259
**cg24883333**	1	25071513	CLIC4	0.0127476	0.587485558277382	0.0814415238184259
**cg14804102**	16	89339261	ANKRD11;ANKRD11;ANKRD11	-0.0281457	-0.0436051576575561	0.0814415238184259
**cg24280449**	16	55690309	SLC6A2;SLC6A2;SLC6A2	0.0322991	0.615428181520352	0.0814415238184259
**cg06520520**	22	46594254	PPARA;PPARA;PPARA;PPARA	0.0107748	0.0161555524880397	0.0814415238184259
**cg16648632**	6	6003584	NRN1	0.0434475	0.619448228939364	0.08167041962779
**cg02231834**	16	11560831	LOC101927131	-0.0336043	-0.053179494135463	0.0816908409525518
**cg11482290**	12	51136416	DIP2B	-0.0284444	-0.0451826590081795	0.0816908409525518
**cg00448889**	15	60714492	NARG2;NARG2	-0.0812434	-0.156937318625814	0.0817246500291242
**cg08803415**	20	30466863	TTLL9	-0.0804697	-0.169303680293586	0.0817246500291242
**cg12511277**	11	134633148		-0.0159383	-0.0240374096514543	0.0817246500291242
**cg13650856**	2	63005412	EHBP1;EHBP1;EHBP1;EHBP1	-0.0362583	-0.056437290984992	0.0817351082821855
**cg16736300**	19	8579199	ZNF414;ZNF414	0.016242	0.834521368709003	0.0817543124009382
**cg09831611**	2	54261700		-0.0550777	-0.101923117050635	0.0817543124009382
**cg13302166**	17	8534048	MYH10	0.0230257	0.470429874690153	0.0817543124009382
**cg17826675**	3	156392703	TIPARP-AS1;TIPARP;TIPARP	0.040632	1.02280230950137	0.0817543124009382
**cg12858212**	16	4451467	CORO7-PAM16;CORO7;CORO7;CORO7	-0.0131147	-0.0202521576993327	0.0817543124009382
**cg10954363**	3	112930438	BOC	0.0104477	0.611561793156546	0.0817543124009382
**cg15577706**	17	33763575	SLFN13	-0.0339716	-0.0534379453737258	0.0817543124009382
**cg15741083**	13	21099541	CRYL1	0.0224043	0.96528096797583	0.0817543124009382
**cg04827646**	14	72971119	RGS6;RGS6;RGS6;RGS6;RGS6;RGS6;RGS6;RGS6;RGS6;RGS6	-0.0412257	-0.0669600715502142	0.0817543124009382
**cg20549229**	8	98655248	MTDH	-0.0455093	-0.0789166532206119	0.0817543124009382
**cg23346408**	10	81001957	ZMIZ1	0.0239336	0.456913311694689	0.0817543124009382
**cg06442378**	10	34587378	PARD3;PARD3;PARD3;PARD3;PARD3;PARD3;PARD3;PARD3	-0.0750186	-0.152268848951066	0.0817543124009382
**cg13151361**	2	179298300	PRKRA;MIR548N;PRKRA;PRKRA	-0.0966551	-0.183721388010715	0.0817543124009382
**cg16303510**	6	14394735		-0.0122786	-0.0185582197506712	0.0817543124009382
**cg04241305**	5	137689053	KDM3B	0.0125197	0.457165623014015	0.0817543124009382
**cg05151395**	17	8192371	SLC25A35;RANGRF	0.0158581	0.491614816375493	0.0817543124009382
**cg17589325**	1	201924583	TIMM17A	0.040507	0.754258418892012	0.0817543124009382
**cg05226454**	10	725877	DIP2C	0.00979737	0.0148754511356934	0.0817543124009382
**cg01882095**	19	17420128	DDA1	0.0137901	0.928030609419805	0.0817543124009382
**cg05090972**	16	69788427	NOB1	0.00602102	0.39760373615222	0.0817543124009382
**cg09818930**	16	31106315	VKORC1;VKORC1	0.0265928	0.925179190944276	0.0817543124009382
**cg10058885**	22	38673051		-0.0888124	-0.174192013820255	0.0817543124009382
**cg17736252**	1	78273462	FAM73A	-0.0481229	-0.0895940817638817	0.0817543124009382
**cg10698424**	9	114423570	GNG10;DNAJC25-GNG10	0.00665252	0.48054967539005	0.0817543124009382
**cg18100653**	7	155089030	INSIG1;INSIG1;INSIG1	0.0215638	0.734742324536004	0.0817543124009382
**cg05630523**	19	12886518	HOOK2;HOOK2	0.0131603	0.720255997144754	0.0817543124009382
**cg01979888**	12	6665424	IFFO1;IFFO1	0.013765	0.480339263719533	0.0817543124009382
**cg17749261**	3	156392701	TIPARP-AS1;TIPARP;TIPARP	0.0943698	2.21118344560835	0.0817543124009382
**cg03498383**	16	68002044	SLC12A4;SLC12A4;SLC12A4;SLC12A4	0.00564202	0.274420855553987	0.0817758765894366
**cg20607106**	1	95538550	ALG14	0.018435	0.49722880321711	0.0819650163810877
**cg24697311**	18	77712348	PQLC1;PQLC1;PQLC1	0.0107678	0.458341897965572	0.0819650163810877
**cg17681277**	16	3070264	TNFRSF12A	0.0544559	0.358903794002637	0.0820047408072486
**cg06455032**	19	45251911	BCL3	0.0409016	0.275176524265218	0.0820047408072486
**cg20942198**	3	196014751	PCYT1A	0.00753867	0.494092269260155	0.0820047408072486
**cg04888730**	16	3712318	TRAP1;TRAP1	0.0161035	0.024884888399446	0.0820047408072486
**cg22732549**	8	8820073		-0.0698961	-0.122779427944503	0.0820047408072486
**cg22051338**	21	34989581	CRYZL1	-0.0406275	-0.067579021569009	0.0820455234964
**cg13499468**	1	23494459	LUZP1;LUZP1	0.038204	0.394319920792089	0.0820894269120116
**cg07301329**	16	85648263	KIAA0182;KIAA0182	0.0477726	0.350271054625089	0.0820894269120116
**cg05590233**	17	109545	RPH3AL	-0.0391479	-0.0639228602978652	0.0820894269120116
**cg01109763**	4	3387281	RGS12;RGS12;RGS12	0.0298177	0.731985158195133	0.0821019867366648
**cg05303981**	9	21995305	CDKN2A;CDKN2B-AS1;CDKN2B-AS1;CDKN2B-AS1;CDKN2B-AS1;CDKN2B-AS1;CDKN	0.0287836	0.598861551798119	0.0821019867366648
**cg11384654**	12	43041781	LOC101927058	-0.0696553	-0.139898117023822	0.0821932952287104
**cg06705772**	11	62439523	C11orf48;C11orf83	0.00655605	0.449729081709688	0.0821932952287104
**cg05787120**	3	50365854	TUSC2	0.0157629	0.691519253389506	0.0821932952287104
**cg09832197**	9	97007732		-0.0745395	-0.143175661900673	0.0822454053551294
**cg23604584**	13	50367114	KPNA3	0.0175593	0.676105778246371	0.0822719801133613
**cg16767327**	2	29331293	CLIP4	-0.048735	-0.0802158823320895	0.0823199235122547
**cg10723075**	20	20693064	RALGAPA2	0.0108272	0.495989316935868	0.0823867859668522
**cg18780259**	19	19006582	GDF1;LASS1;LASS1	0.0414045	0.612373709707421	0.0823867859668522
**cg07249919**	5	53813391	SNX18;SNX18;SNX18	0.0161937	0.323497660198889	0.0823867859668522
**cg05797914**	5	110702850	CAMK4	-0.109757	-0.227903181313591	0.0823867859668522
**cg00558174**	5	14872551	ANKH	0.0123721	0.530371771419631	0.0823867859668522
**cg08077890**	18	157838	USP14;USP14	-0.0798154	-0.143233768537799	0.0823867859668522
**cg09856869**	1	33286822	S100PBP;S100PBP	-0.067439	-0.159057408791195	0.0823867859668522
**cg21605986**	5	175788725	KIAA1191;KIAA1191;KIAA1191;KIAA1191;KIAA1191;KIAA1191	0.033842	1.22543803201557	0.0823867859668522
**cg20506184**	10	7453329	SFMBT2;SFMBT2	0.0102988	0.52758315271208	0.0823867859668522
**cg02745211**	3	132757106	TMEM108;TMEM108	0.00503896	0.313374997624898	0.0823867859668522
**cg02240315**	1	41707901	SCMH1;SCMH1;SCMH1;SCMH1	0.0126592	0.438692364880103	0.0823867859668522
**cg18885865**	14	59655267	DAAM1	0.0135727	0.715450089261765	0.0823867859668522
**cg08396677**	2	136288699	ZRANB3;R3HDM1;ZRANB3	0.00486294	0.291054875711546	0.0823867859668522
**cg17386899**	15	79603965	TMED3	0.0269044	0.642515317938987	0.0823867859668522
**cg07803781**	1	11333577	UBIAD1;UBIAD1	0.00667931	0.536975027923937	0.0823867859668522

**cg07654559**	2	74710618	TTC31;CCDC142;TTC31	0.0463904	0.557126735083305	0.0823867859668522
**cg08139833**	11	67798212	NDUFS8	0.00354854	0.416327193773097	0.0823867859668522
**cg22748698**	17	7146447	GABARAP	0.0127312	0.432853628826418	0.0823867859668522
**cg22841635**	14	69951305	FLJ44817	0.0211427	0.362378511579842	0.0823867859668522
**cg22801675**	5	175971121		0.0122575	0.51620317708674	0.0823867859668522
**cg13298932**	1	155100169	EFNA1;EFNA1	0.0251105	0.799362195893556	0.0823867859668522
**cg03629096**	14	73915638	NUMB;NUMB;NUMB;NUMB	-0.0802815	-0.144128968971492	0.0823867859668522
**cg23346107**	14	69864717	ERH;SLC39A9	0.0117562	0.47451074441975	0.0823867859668522
**cg11144753**	14	103740065		0.028068	0.689621819392766	0.0823867859668522
**cg14013133**	1	43887850	KIAA0467	0.0123269	0.0186455009805289	0.0823867859668522
**cg22858733**	6	35995359	MAPK14;MAPK14;MAPK14;MAPK14	0.00758358	0.535702859247489	0.0823867859668522
**cg14354168**	6	1619134		-0.0544584	-0.104567402427658	0.0823867859668522
**cg26494252**	19	15083916	SLC1A6	-0.0519934	-0.119480967983199	0.0823867859668522
**cg04129754**	19	28660027		-0.0748483	-0.188592691118932	0.0823867859668522
**cg27060912**	19	6517005		0.0168872	0.459322433816302	0.0823867859668522
**cg07691761**	5	131892711	RAD50;RAD50;RAD50;RAD50	0.00823551	0.382860969052017	0.0823867859668522
**cg11704396**	3	48723701	NCKIPSD;NCKIPSD	0.0288791	0.960254012517783	0.0823867859668522
**cg01601097**	1	229364737		-0.0499741	-0.0891757921967299	0.0823867859668522
**cg24671245**	10	76585406	MYST4	0.0214917	0.494148775879606	0.0823867859668522
**cg13117590**	6	3981622		-0.0262221	-0.0405777832468137	0.0823867859668522
**cg07045469**	7	48494579	ABCA13	0.0733667	0.118990820062115	0.0823867859668522
**cg27102864**	16	58535418	NDRG4;NDRG4;NDRG4	0.017118	0.559439911672481	0.0825116137994541
**cg00906147**	4	39979791	PDS5A;PDS5A;PDS5A	0.00812562	0.380071879115992	0.0825996943841617
**cg12562042**	16	19098052		0.00852627	0.407302566158156	0.0825996943841617
**cg22322834**	12	50135138	TMBIM6;TMBIM6	0.00922347	0.378572715658719	0.0825996943841617
**cg14819616**	10	128839969	DOCK1;DOCK1	-0.0666964	-0.125609968760473	0.0825996943841617
**cg00803088**	10	43600706	RET;RET	0.0571401	2.1024312454709	0.0825996943841617
**cg25123308**	2	113300087	POLR1B;POLR1B	0.014847	0.525715806360374	0.0825996943841617
**cg05079543**	19	42388414	ARHGEF1;ARHGEF1;ARHGEF1	0.0258908	0.679653326079312	0.0825996943841617
**cg01119259**	14	44977304	FSCB	-0.0728687	-0.140126618094556	0.0825996943841617
**cg10624480**	1	153936631	SLC39A1	0.00301585	0.393008518535928	0.0825996943841617
**cg26064774**	4	6666009		0.078392	0.654286787449608	0.0825996943841617
**cg11701951**	12	51477122	CSRNP2;CSRNP2	0.00824779	0.460824224853452	0.0825996943841617
**cg22942210**	14	69654470		-0.0498874	-0.0831544373723812	0.0825996943841617
**cg17618987**	22	16190030		-0.058866	-0.10921598212481	0.0825996943841617
**cg26314507**	15	25902323		-0.039793	-0.0630016128053776	0.0825996943841617
**cg00502972**	17	6543355	TXNDC17;KIAA0753	0.0343233	0.661773325733138	0.0825996943841617
**cg08136599**	6	8042158	EEF1E1-BLOC1S5;BLOC1S5;BLOC1S5;BLOC1S5-TXNDC5	-0.0549408	-0.0996047854342244	0.0825996943841617
**cg17372361**	14	61612138		-0.0112754	-0.016768963429607	0.0825996943841617
**cg00227490**	11	9336016	TMEM41B;TMEM41B;TMEM41B;TMEM41B;TMEM41B	0.00776388	0.50295224226309	0.0825996943841617
**cg20477448**	4	13656704		0.00661765	0.00977039759548299	0.0825996943841617
**cg15896339**	1	876249	SAMD11	0.0142242	0.570273716917833	0.0825996943841617
**cg01326942**	6	90596921		-0.0496602	-0.0813975338386198	0.0825996943841617
**cg07889826**	3	50127152	RBM5	0.014004	0.47891711206763	0.0825996943841617
**cg24346110**	15	83407523	ACTG1P17	-0.0846955	-0.158397765872212	0.0825996943841617
**cg14107485**	8	27950471	ELP3;ELP3;ELP3;ELP3;ELP3;ELP3	0.0211102	0.715465482188239	0.0825996943841617
**cg17944940**	17	73285520	SLC25A19;SLC25A19;SLC25A19;SLC25A19;SLC25A19;SLC25A19	0.0337685	1.12313108043454	0.0825996943841617
**cg05062612**	12	108523409	WSCD2	0.00504195	0.346478818759469	0.0825996943841617
**cg08410109**	2	101179395	PDCL3	0.0185361	0.688865715086566	0.0825996943841617
**cg03652211**	12	108736538		-0.0339535	-0.0592433557664198	0.0825996943841617
**cg02688348**	11	57103429	SSRP1	0.0451579	1.0669581988487	0.0825996943841617
**cg17353057**	21	46294185	PTTG1IP	0.018078	0.814876158427757	0.0825996943841617
**cg07640747**	10	93392870	PPP1R3C	0.0157911	0.481092103283054	0.0825996943841617
**cg00689580**	6	10420798	TFAP2A	0.045967	0.742214872348575	0.0825996943841617
**cg21231262**	9	122521448		-0.0663171	-0.182753494545701	0.0825996943841617
**cg00805320**	16	68057225	DUS2L;DUS2L;DDX28;DDX28	0.0171988	0.970912547529664	0.0825996943841617
**cg27325395**	9	99089615	SLC35D2;SLC35D2;SLC35D2	-0.0384912	-0.061647998977324	0.0825996943841617
**cg13339291**	17	80155868	CCDC57	-0.0595542	-0.107863844457028	0.0825996943841617
**cg08818195**	12	133066505	FBRSL1	0.0445303	0.488336393268662	0.0825996943841617
**cg14264747**	3	133383753		-0.0211415	-0.0335494769496024	0.0825996943841617
**cg27223543**	1	45098125	RNF220	0.0195343	0.565996291426639	0.0825996943841617
**cg02795515**	17	37381273	STAC2	0.0148687	0.542569822830144	0.0825996943841617
**cg09838326**	8	30629816		-0.0213192	-0.0342231648619619	0.0825996943841617
**cg05080815**	19	35379312		-0.0140925	-0.0212619290501054	0.0825996943841617
**cg24202654**	2	171674567	GAD1;GAD1	0.0158655	0.354505465971129	0.0825996943841617
**cg20788115**	15	75636870		-0.0495199	-0.0791204560562389	0.0825996943841617
**cg15020726**	15	31196382	MTMR15;MTMR15;MTMR15;MTMR15	0.0090955	0.462825609623584	0.0825996943841617
**cg15495920**	1	65778729	DNAJC6	-0.0232107	-0.0359620191696452	0.0825996943841617
**cg00872998**	9	74820402	GDA;GDA;GDA;GDA	-0.093149	-0.214274964586273	0.0825996943841617
**cg21484680**	2	27593328	EIF2B4;EIF2B4;SNX17;EIF2B4	0.0262032	0.753047096794773	0.0825996943841617
**cg17814109**	5	1386363		0.0248838	0.561887006517678	0.0826207889194387
**cg07102345**	9	111696472	IKBKAP;IKBKAP;FAM206A	0.0228074	0.481231766218665	0.082629327055183
**cg04553410**	7	150864885	GBX1	0.0242052	0.899281693104227	0.082629327055183
**cg13717023**	5	53606639	15,00 ARL	0.0245353	0.859248430386943	0.082629327055183
**cg23451324**	19	11708147	ZNF627	0.00763783	0.480320323634346	0.082629327055183
**cg26329992**	4	56719469	EXOC1;EXOC1;EXOC1	-0.00561562	-0.00822928896105215	0.0827147122842582
**cg18677773**	5	168018169		-0.0767063	-0.16113727079145	0.0827226567216581
**cg26260369**	14	68141723	VTI1B	0.0459022	1.28734823142486	0.0827226567216581
**cg26672794**	1	25257131	RUNX3;RUNX3	0.0422505	0.467989449741244	0.0827226567216581
**cg13414679**	11	70243843	CTTN;CTTN	0.0321062	0.653643548611843	0.0827226567216581
**cg14580567**	4	145567271	HHIP;HHIP	0.0169041	0.456349202527035	0.0827226567216581
**cg15246686**	9	72715990	MAMDC2;MAMDC2	-0.0220539	-0.0336214862024681	0.0827226567216581

**cg05361503**	16	4475729	DNAJA3;DNAJA3;DNAJA3	0.0203365	0.964367557322025	0.0827226567216581
**cg06362482**	8	16021835	MSR1;MSR1;MSR1	-0.0406533	-0.361574541049669	0.0827226567216581
**cg19566272**	6	139695824	CITED2;CITED2;CITED2	0.00736344	0.613585117151741	0.0827226567216581
**cg17772342**	5	134871686	NEUROG1	0.0584517	0.334191584425774	0.0827226567216581
**cg03631461**	5	179089038		-0.00700001	-0.0103779107154385	0.0827226567216581
**cg18013408**	7	100487659	UFSP1;ACHE;ACHE	0.0193266	0.328929969406111	0.0827226567216581
**cg11218415**	8	104030731		-0.0535996	-0.0878979430975534	0.0827226567216581
**cg19756512**	11	35068439		-0.0321618	-0.0504238633431439	0.0827226567216581
**cg18012350**	15	74753669	UBL7;UBL7	0.0192544	0.632433276773893	0.0827226567216581
**cg04847795**	1	70820736	HHLA3;ANKRD13C;HHLA3;HHLA3;HHLA3	0.025508	0.901395933473746	0.0827226567216581
**cg09591886**	16	22333264	POLR3E	0.00672594	0.0100511707316762	0.0827226567216581
**cg05203855**	19	7994445	TIMM44	0.0173048	0.375055211464623	0.0827226567216581
**cg22084302**	13	20246166	MPHOSPH8	0.00357821	0.00522875833072869	0.0827226567216581
**cg13789219**	13	99438910		-0.0224605	-0.0342209974195565	0.0827226567216581
**cg04294412**	4	7045720	CCDC96;TADA2B	0.0284111	1.02138056875953	0.0827226567216581
**cg21526572**	10	133850592		0.0368748	0.430197330149547	0.0827226567216581
**cg16533336**	7	70765583	WBSCR17	-0.0389594	-0.0699915207325286	0.0827226567216581
**cg09111258**	17	46114933	COPZ2;MIR152	0.0261496	0.650423618550959	0.0827226567216581
**cg08611430**	5	137801033	EGR1	0.00954145	0.475832761082719	0.0827226567216581
**cg21381136**	1	8445818	RERE;RERE;RERE	-0.0496645	-0.0867873992600977	0.0827226567216581
**cg04242710**	2	201729573	CLK1;CLK1;CLK1;CLK1	0.00846506	0.459852045228263	0.0827226567216581
**cg09432409**	5	66540037		-0.0444966	-0.079149948389083	0.0827226567216581
**cg24964915**	3	196676228	PIGZ	-0.00343297	-0.00514744072614754	0.0827672357731161
**cg11948381**	11	47574185	CUGBP1	0.0101686	0.551828287527866	0.0828165505546208
**cg20906495**	6	150238016	RAET1G	-0.0124432	-0.0186115740640604	0.0828165505546208
**cg23721088**	10	80955256	ZMIZ1	0.0314327	0.659191906637322	0.0828165505546208
**cg07771804**	19	2268130	OAZ1;OAZ1	-0.0293611	-0.0451694844165123	0.0828165505546208
**cg02457826**	20	30310733	BCL2L1;BCL2L1	0.0174219	0.554132472611083	0.0828165505546208
**cg25742745**	6	30689865	TUBB	0.0238511	0.408504619095484	0.0828165505546208
**cg24790837**	7	138838713	TTC26;TTC26;TTC26;TTC26;TTC26	-0.0751173	-0.172908168312305	0.0829472074408977
**cg02463426**	19	16683387	SLC35E1	0.0288036	0.661635532738922	0.0829472074408977
**cg12934646**	6	159466962	TAGAP;TAGAP;TAGAP	-0.0172336	-0.0260066413583102	0.0829472074408977
**cg21239269**	8	77592757	ZFHX4;ZFHX4-AS1	-0.0308249	-0.0515013439002157	0.0829878902353803
**cg04558100**	2	73521030	EGR4	0.00495357	0.36156198218457	0.0830056722905755
**cg10671572**	6	150071503	PCMT1	-0.0715477	-0.447011663681616	0.083013465099289
**cg26375263**	14	76843202	ESRRB	0.0133844	0.531982752534643	0.0830275998867893
**cg02620189**	19	40927363		0.0115332	0.511227994799562	0.0830275998867893
**cg02447122**	2	25142523	ADCY3	0.0228618	0.607951903886269	0.0830275998867893
**cg09987128**	9	84602238	SPATA31D1	-0.0600265	-0.106648214293578	0.0830275998867893
**cg05881034**	3	42623675	SEC22C;SEC22C	0.035026	0.865388230581578	0.0830275998867893
**cg22159483**	17	1588198	PRPF8	0.0340283	0.562912268450687	0.0830275998867893
**cg11208039**	11	19138753	ZDHHC13;ZDHHC13;ZDHHC13;ZDHHC13	0.0207132	0.760499235619559	0.0830275998867893
**cg02052142**	14	60794866		0.0120448	0.459255403827944	0.0830275998867893
**cg12991306**	8	27941405	C8orf80	-0.0682108	-0.157644472302921	0.0830275998867893
**cg26646953**	17	17183928	COPS3;COPS3;COPS3	0.00595722	0.398861152352408	0.0830275998867893
**cg19280901**	2	173443071	PDK1;PDK1;PDK1;PDK1	-0.0605119	-0.101062726213287	0.0830275998867893
**cg03621258**	6	31613356	BAG6;BAG6;BAG6;BAG6;BAG6;BAG6	0.0135789	0.0205475987483601	0.0830275998867893
**cg07018260**	11	10536864	RNF141	-0.0805562	-0.157089415935475	0.0830275998867893
**cg11651785**	8	38244580	LETM2;LETM2;LETM2;LETM2;LETM2;LETM2	0.0232411	0.541056303982293	0.0830275998867893
**cg06179586**	6	29969539	NCRNA00171	-0.0389152	-0.0619979127947179	0.0830275998867893
**cg04414295**	14	102606318	WDR20;WDR20;HSP90AA1;WDR20;WDR20;WDR20	0.0298423	0.985467245323967	0.0830715021864001
**cg16252691**	12	94498285		-0.0807518	-0.163863410265057	0.0830760503717604
**cg14787483**	22	39052528	LOC646851;LOC646851;CBY1;CBY1	0.0056473	0.444407843165318	0.0830760503717604
**cg06783524**	2	208460831	CREB1;CREB1;METTL21A	-0.0413281	-0.0650338883331018	0.0830760503717604
**cg05141147**	12	113903386	LHX5	0.0156698	0.516893135867065	0.0830760503717604
**cg12570942**	2	242626270	DTYMK;DTYMK;DTYMK;DTYMK;DTYMK	0.00975274	0.40908647927806	0.0830760503717604
**cg10819560**	15	63774509		-0.0709449	-0.149636781063621	0.0830760503717604
**cg01301842**	5	156815746	CYFIP2;CYFIP2;CYFIP2;CYFIP2	-0.0467203	-0.0845666775214475	0.0830760503717604
**cg08344341**	5	36151993	LMBRD2;SKP2;SKP2;LMBRD2	0.0100576	0.550848585119506	0.0830760503717604
**cg08311403**	11	124735215	ROBO3	0.00443218	0.326536332048526	0.0830760503717604
**cg12128610**	7	66057395		0.00924661	0.521415237518467	0.0830760503717604
**cg13286692**	2	3137500		-0.0444516	-0.0722423532429183	0.0830760503717604
**cg20481419**	12	79850430	MIR5692B	-0.0679656	-0.119387159138368	0.0830760503717604
**cg12109968**	20	50383676	ATP9A	0.0295197	0.59372797272614	0.0830760503717604
**cg01188936**	12	80084361	PAWR	0.0104067	0.788364165616603	0.0830760503717604
**cg27386413**	2	3473947	TRAPPC12	0.00476681	0.00705228143736225	0.0830760503717604
**cg12266151**	20	33291892	TP53INP2	0.0102399	0.387300026935974	0.0830760503717604
**cg17829401**	3	9975317	CRELD1;CRELD1;CRELD1	0.0130554	0.453803383718048	0.0830903758532303
**cg19582614**	12	4381894	CCND2	0.0154002	0.374105874276336	0.0830903758532303
**cg11220663**	2	70994863	ADD2;ADD2;ADD2;ADD2;ADD2	0.0898115	0.822145579819572	0.0831928480211502
**cg26519799**	19	55897819	RPL28;RPL28;RPL28;RPL28;RPL28	0.00585738	0.600817947779366	0.0831928480211502
**cg09418792**	9	123555852	FBXW2;LOC100288842	0.019941	0.740651580163056	0.0833421938001392
**cg09526912**	2	45237431	SIX2	0.033338	0.448192801034516	0.0833561423430311
**cg04361145**	9	123964183	RAB14;RAB14	0.0276563	1.11964557425822	0.0833664672878077
**cg03783420**	9	36400446	RNF38;RNF38;RNF38;RNF38;RNF38	0.00806	0.452800498351718	0.0833725400522609
**cg20773005**	16	79436422		0.0168042	0.0259246556035678	0.0833725400522609
**cg04342230**	11	8986449	TMEM9B;TMEM9B;TMEM9B;TMEM9B;TMEM9B-AS1	0.0329448	0.942757496668362	0.0833725400522609
**cg01216412**	12	110011396	MMAB;MVK;MVK	0.00783003	0.456236872315906	0.0833725400522609
**cg05516012**	11	73019501	ARHGEF17	0.00973739	0.681477517213723	0.0833725400522609
**cg17262065**	7	39653641		-0.0606388	-0.111294436870692	0.0833725400522609
**cg22034943**	11	65383239	PCNXL3	0.0177889	0.919770321386434	0.0833725400522609
**cg22820285**	1	55008241		0.00858018	0.441056385103066	0.0833725400522609

**cg26574964**	11	117049906	SIDT2	0.0248764	0.389294691298255	0.0833725400522609
**cg24069674**	6	27230661		-0.0217953	-0.0330170684622633	0.0833725400522609
**cg18040305**	6	64281886	PTP4A1	0.00691951	0.477329707510734	0.0833725400522609
**cg12591986**	16	14368115		-0.0580962	-0.102349935812084	0.0833725400522609
**cg20630240**	16	66771590	DYNC1LI2;DYNC1LI2	0.00931224	0.0140219801217293	0.0833725400522609
**cg17301114**	2	72971072	EXOC6B	-0.0375654	-0.0604222276059503	0.0833725400522609
**cg03920061**	10	21598708		-0.0354589	-0.055629912935175	0.0833725400522609
**cg19301602**	3	46855194		-0.0953141	-0.182507551676389	0.0833725400522609
**cg24799909**	5	145724482		0.0248505	0.761710023737104	0.0833725400522609
**cg25879102**	1	38600119		0.00812857	0.419940934853229	0.0833725400522609
**cg16974433**	11	35198159	CD44;CD44;CD44;CD44;CD44;CD44;CD44	-0.00538291	-0.00791601126277003	0.0833725400522609
**cg09127400**	6	30712331	IER3	0.0100624	0.645549736307309	0.0833725400522609
**cg15463803**	4	113436765	NEUROG2	0.018642	0.516724748156769	0.0833725400522609
**cg03920887**	1	205616632		-0.0293076	-0.0454097575094518	0.0833725400522609
**cg09699605**	12	57023789	BAZ2A	0.0403977	1.27392211495586	0.0833725400522609
**cg12822972**	9	33524289	ANKRD18B	0.0499308	0.545183129446102	0.0833725400522609
**cg20165111**	11	77300425	AQP11	0.0140728	0.851354817841712	0.0833725400522609
**cg10922289**	11	62648720	SLC3A2;SLC3A2;SLC3A2;SLC3A2;SLC3A2;SLC3A2	0.0128911	0.453417577992804	0.0833725400522609
**cg01353464**	3	38180116	ACAA1;ACAA1;ACAA1;MYD88;MYD88	0.0476781	0.42379841776588	0.0833725400522609
**cg16659151**	3	136233959	STAG1	-0.0687218	-0.136858822984607	0.0833725400522609
**cg24856732**	22	50963486	SCO2;SCO2;SCO2;SCO2	0.0225488	0.688270665879336	0.0833725400522609
**cg01654862**	3	159480947	SCHIP1	0.00951961	0.59252504686737	0.0833725400522609
**cg07767421**	16	5059086	SEC14L5	-0.0381349	-0.0631956051001044	0.0833725400522609
**cg17597744**	8	53627099	RB1CC1;RB1CC1	0.0412609	0.874964355258341	0.0833725400522609
**cg00124488**	6	31509762	BAT1;BAT1;SNORD84;BAT1;BAT1	0.0152234	0.502115817990528	0.0833725400522609
**cg19377127**	22	41033281	MKL1	0.0111326	0.448944997787091	0.0833725400522609
**cg26925343**	16	28857904	TUFM	0.0258541	0.645017166759124	0.0833725400522609
**cg07625039**	5	153817206	SAP30L-AS1	-0.077594	-0.145162369183246	0.0833725400522609
**cg16162734**	2	149543956	EPC2	-0.0240513	-0.0369194026991435	0.0833725400522609
**cg22281380**	7	50861467	GRB10	0.0109443	0.3408480039869	0.0833725400522609
**cg08443851**	16	85646773	KIAA0182;KIAA0182	0.0410547	0.801261216225541	0.0833725400522609
**cg05402641**	2	180899188		-0.0536365	-0.0902786115361921	0.0833725400522609
**cg11675409**	15	44083712	SERF2	-0.0470495	-0.0831325428654862	0.0833725400522609
**cg14438736**	3	171617543	TMEM212-AS1	-0.0360007	-0.0659015298315747	0.0833725400522609
**cg16182055**	5	137879011	ETF1	0.0159507	0.536771260245784	0.0833725400522609
**cg07283152**	11	8284997	LMO1;LMO1	0.0377396	0.565877301407896	0.0833725400522609
**cg06936006**	7	133074606	EXOC4;EXOC4	-0.0373305	-0.0593156225458457	0.0833725400522609
**cg10059378**	16	4817345	ZNF500	0.0166122	0.653701272830046	0.0833725400522609
**cg02641865**	1	40595336		-0.0674273	-0.114684516454897	0.0833725400522609
**cg18560247**	4	78071590		-0.0583263	-0.0975655510917974	0.0833725400522609
**cg19609713**	12	6558051	CD27;CD27-AS1	-0.031589	-0.0514744624909787	0.0833725400522609
**cg03766306**	1	90461091	LOC492303;ZNF326;ZNF326	0.0102273	0.495931977454571	0.0833725400522609
**cg24390820**	12	131189380		-0.0347968	-0.0600532665865005	0.0833725400522609
**cg12225467**	1	40372098		-0.0315278	-0.0555217861498529	0.0833725400522609
**cg03602124**	1	113258101	PPM1J	0.00985216	0.496148320993016	0.0833725400522609
**cg11698099**	16	67695462	PARD6A;ACD;ACD;PARD6A;ACD	0.0219023	0.371679613298036	0.0833725400522609
**cg23272668**	11	11863629	USP47;USP47	0.00818363	0.453163522984503	0.0833725400522609
**cg09847415**	22	40633982	TNRC6B;TNRC6B;TNRC6B	0.00601837	0.00885840270841351	0.0833725400522609
**cg03410961**	6	391680	IRF4	0.0293469	0.431897875610589	0.0833725400522609
**cg02962095**	1	145590829	NUDT17;POLR3C;POLR3C;NBPF20;NBPF10;NBPF10	-0.0679011	-0.121639139102579	0.0833725400522609
**cg19516041**	4	125633907	ANKRD50;ANKRD50	0.0479679	0.436488972741971	0.0833725400522609
**cg14163444**	7	99595348		0.0183614	0.429474427292951	0.0833725400522609
**cg06898080**	12	111807837	FAM109A;FAM109A;FAM109A	-0.0389715	-0.0686193344742079	0.0833725400522609
**cg27556913**	4	1341059	KIAA1530	0.00743212	0.400474738205833	0.0833725400522609
**cg06006168**	12	53893448	TARBP2;TARBP2;MAP3K12;MAP3K12	0.0437873	0.490755020098989	0.0833725400522609
**cg09397900**	16	2070528	NPW	0.0117193	0.635725189959481	0.0833725400522609
**cg07226281**	16	1756622	MAPK8IP3;MAPK8IP3	0.0180733	0.731329622844781	0.0833725400522609
**cg24180066**	19	56632673	ZNF787	0.0188708	0.534560116036689	0.0833725400522609
**cg22323350**	5	65222292	ERBB2IP;ERBB2IP;ERBB2IP;ERBB2IP;ERBB2IP;ERBB2IP	0.0305969	0.598020075794159	0.0833725400522609
**cg26085714**	1	245133458	LOC101928068;EFCAB2;EFCAB2;EFCAB2;EFCAB2	0.0346068	0.311466688694982	0.0833725400522609
**cg01637377**	17	8567313		-0.045466	-0.0782325039390296	0.0833725400522609
**cg17172090**	6	2926650		-0.0486456	-0.105488035111673	0.0833725400522609
**cg12234226**	6	90932450	BACH2;BACH2	-0.0719529	-0.170262264468707	0.0833725400522609
**cg04429367**	13	113939322		-0.0231369	-0.0357570145110977	0.0833725400522609
**cg16713009**	19	44223436	IRGC	-0.011817	-0.0177729466182903	0.0833725400522609
**cg11564042**	3	150479915	SIAH2	0.0453938	0.599585050658647	0.0833725400522609
**cg15586420**	15	90294517	MESP1;MESP1	0.0320407	0.578660332685478	0.0833725400522609
**cg11276193**	17	33570022	SLFN5	0.00405065	0.348497958548859	0.0833725400522609
**cg06206471**	16	4364052		0.00786357	0.357934797517731	0.0833725400522609
**cg23310839**	18	19180999	ESCO1	0.0137582	0.513019416254461	0.0833725400522609
**cg18772071**	4	89618324	NAP1L5;HERC3;NAP1L5	0.0475084	0.14191549250715	0.0833725400522609
**cg15012981**	1	11741009	MAD2L2;MAD2L2	0.0284272	1.36979192096249	0.0833725400522609
**cg21572957**	3	195564748		-0.0307498	-0.0486843633998462	0.0833725400522609
**cg19398269**	6	28678460		-0.0178915	-0.0513568222309907	0.0833725400522609
**cg08563487**	20	2633027	MIR1292;NOP56;NOP56	0.0244304	0.930312733035727	0.0833725400522609
**cg18221711**	8	103963195	AZIN1-AS1;AZIN1-AS1	-0.0168011	-0.0252225020504621	0.0833725400522609
**cg10457504**	17	19281708	MAPK7;MAPK7;MAPK7;MAPK7	0.016734	0.342564415680892	0.0833725400522609
**cg16524332**	4	3075655	HTT	0.0190139	0.626461447590942	0.0833725400522609
**cg27619163**	17	7982806	ALOX12B	0.0438988	1.47398467306146	0.0833725400522609
**cg15337463**	20	5930963	TRMT6;MCM8;MCM8;MCM8;MCM8;MCM8;TRMT6;TRMT6	0.00664117	0.472729789534546	0.0833725400522609
**cg00521887**	18	39535162	PIK3C3	0.00848368	0.401682394537633	0.0833725400522609
**cg08658318**	21	46351329		0.0111574	0.628944415192806	0.0833725400522609

**cg12900061**	15	43199409	TTBK2	-0.0590267	-0.100700830697853	0.0833725400522609
**cg26673151**	17	37887607	C17orf37	-0.0594203	-0.101298186464133	0.0833725400522609
**cg22652733**	10	74452328	MCU;MCU;MCU;MCU	0.0240286	0.618372104171829	0.0833725400522609
**cg11716640**	1	113933411	MAGI3;MAGI3	0.0252324	0.638488556012157	0.0833725400522609
**cg11187916**	1	145562715	ANKRD35	0.0232791	0.398500674001206	0.0833725400522609
**cg16676952**	20	62612301	SAMD10;PRPF6	0.00519287	0.419352289187401	0.0833725400522609
**cg08268585**	5	34839194	TTC23L	0.00731758	0.491125988590222	0.0833725400522609
**cg10625705**	20	307320	SOX12	0.0189134	0.421383061857904	0.0833725400522609
**cg19850545**	22	41347463	RBX1	0.0253571	0.754544200663347	0.0833725400522609
**cg13240253**	3	71245529	FOXP1	-0.0614645	-0.10344410795125	0.0833725400522609
**cg08125574**	4	186058542		-0.0165553	-0.0249982865485147	0.0833725400522609
**cg08889009**	15	59157123		0.0652604	0.293072599021368	0.0833725400522609
**cg01181053**	11	57425124	CLP1;CLP1	0.00824547	0.543685028518982	0.0833725400522609
**cg12608603**	14	91926636	PPP4R3A;PPP4R3A	-0.0510828	-0.0882495798576423	0.0833725400522609
**cg08694699**	16	68270282	ESRP2	0.0210494	0.526826674351028	0.0833725400522609
**cg04369964**	11	63997586	DNAJC4	0.0122899	0.323518293140393	0.0833725400522609
**cg02294570**	12	122362615	WDR66	-0.0580236	-0.159934729171523	0.0833725400522609
**cg05711145**	16	84224633	ADAD2;ADAD2	-0.0174531	-0.0273953148825945	0.0833725400522609
**cg12811797**	3	156392549	TIPARP-AS1;TIPARP;TIPARP;TIPARP	0.0301031	0.711724984193679	0.0833725400522609
**cg13486309**	1	155329113	ASH1L	-0.0867555	-0.16659806458086	0.0833725400522609
**cg18976974**	8	103990325		-0.0567376	-0.0951867048336709	0.0833725400522609
**cg23091741**	1	226111643	PYCR2	0.0263246	0.71679840567618	0.0833725400522609
**cg00730780**	10	30316187	KIAA1462	0.016088	0.939499930569006	0.0833725400522609
**cg07776847**	4	140201065	C4orf49	0.00445435	0.336372342822438	0.0833725400522609
**cg11124980**	7	105925837	NAMPT	0.0173671	0.679055786570676	0.0833725400522609
**cg13373321**	12	49076048	C12orf41	0.00873496	0.751097734377905	0.0833725400522609
**cg07961283**	21	36261057	RUNX1;RUNX1;RUNX1	0.0309447	1.00532089791503	0.0833725400522609
**cg06703062**	1	31191648	MATN1	0.0380348	1.74751298937554	0.0833725400522609
**cg15685834**	1	18970180	PAX7;PAX7;PAX7	0.0110904	0.399868968217708	0.0833725400522609
**cg22008625**	8	10588386	SOX7	0.00884163	0.371629712560382	0.0833725400522609
**cg03213505**	9	139025831		0.0325334	0.571192578400538	0.0833725400522609
**cg07697635**	5	77656183	SCAMP1	0.00645489	0.584779340821793	0.0833725400522609
**cg05995220**	20	55964998	RBM38;RBM38	0.0299379	0.319630483552036	0.0833725400522609
**cg14846380**	12	125333322	SCARB1;SCARB1	0.00316544	0.00465349083505806	0.0833725400522609
**cg13324603**	17	74733680	MFSD11;MIR636;SRSF2;SRSF2;SRSF2;MFSD11;MFSD11;MFSD11;MFSD11;MFSD11;M	0.0761916	1.73469821846795	0.0833725400522609
**cg13530292**	19	42721476	DEDD2	0.00797382	0.556375314213312	0.0833725400522609
**cg02999208**	15	42183840	SPTBN5	-0.0507754	-0.0941834490789406	0.0833725400522609
**cg22535773**	10	98461202	PIK3AP1	-0.0564105	-0.111056189027515	0.0833725400522609
**cg12899157**	18	3452302	TGIF1;TGIF1;TGIF1;TGIF1;TGIF1;TGIF1;TGIF1;TGIF1	0.0163455	0.69277129872911	0.0833725400522609
**cg14855607**	14	74706891	VSX2	0.0276689	0.337519671635716	0.0833725400522609
**cg02215960**	3	50607131	HEMK1	0.0164404	0.828652184831434	0.0833725400522609
**cg24356321**	1	186344702	TPR;C1orf27;MIR548F1;C1orf27;C1orf27	0.0245213	0.689746230996153	0.0833725400522609
**cg12915591**	11	43965360	LOC387763;LOC387763	0.010486	0.778995200362352	0.0833725400522609
**cg01061575**	22	45098367	PRR5-ARHGAP8;PRR5;PRR5;PRR5;PRR5;PRR5	0.0172111	0.410923867451485	0.0833734077776146
**cg11736539**	6	36838237	C6orf89;PPIL1	-0.0197076	-0.0296536794648711	0.0834162432682024
**cg24729481**	1	43833600	ELOVL1;ELOVL1	0.0175255	0.760215402032836	0.0834162432682024
**cg15991309**	17	80291688	SECTM1;SECTM1	0.0155124	0.601268647288977	0.0834162432682024
**cg04938152**	17	17888990	LRRC48;LRRC48;LRRC48;LRRC48	-0.0441344	-0.0829441897190523	0.0834360043272571
**cg04924415**	12	100661456	SCYL2;DEPDC4	0.0433909	0.592545775873789	0.0834360043272571
**cg10949583**	2	179343740	FKBP7;FKBP7;PLEKHA3;MIR548N	0.00869655	0.459577241803645	0.0834360043272571
**cg10679714**	8	145691133	KIFC2;CYHR1;CYHR1;CYHR1	0.0550479	0.294409867650591	0.0834360043272571
**cg07207490**	11	118492463	PHLDB1;PHLDB1;PHLDB1	-0.0524529	-0.14342533729686	0.0834360043272571
**cg03045021**	9	131644678	LRRC8A;LRRC8A;LRRC8A;CCBL1;CCBL1;CCBL1;CCBL1;CCBL1	0.012535	0.578585815255556	0.0834892971239766
**cg17426237**	10	51463156		-0.0403205	-0.0675961844558074	0.0835887417406499
**cg09130192**	2	10161320		-0.072839	-0.137944927221701	0.0835887417406499
**cg04095923**	21	37839286	CLDN14;CLDN14;CLDN14;CLDN14;CLDN14	-0.00916844	-0.013554743884011	0.0835887417406499
**cg02744670**	4	2470688	RNF4;RNF4;RNF4	0.0821127	1.15101203586061	0.0836395973810918
**cg16015797**	17	73021048		-0.0560811	-0.0939266009026822	0.0836395973810918
**cg06138422**	12	133338013	ANKLE2	0.0247259	0.627887346606607	0.083646584198182
**cg12345609**	1	110165150	AMPD2;AMPD2;AMPD2;AMPD2;AMPD2	0.0120763	0.0184782027750917	0.083646584198182
**cg19669078**	3	42543564	VIPR1;VIPR1;VIPR1;VIPR1;VIPR1	0.0662763	1.09102470815522	0.083646584198182
**cg06952910**	4	122744652	CCNA2	0.0176548	0.880854294010458	0.0836609040083184
**cg06677332**	11	34074341	CAPRIN1;CAPRIN1	0.0113836	0.43720283392758	0.0837705860438025
**cg26363634**	1	110162485	AMPD2;AMPD2;AMPD2	0.00909933	0.519598059526681	0.0838091144349962
**cg03785281**	13	41241501	FOXO1	0.0223375	0.985933096273204	0.0838117917806502
**cg01801368**	6	33281322	TAPBP;TAPBP;TAPBP	0.0132752	0.573443008494097	0.0838507173041068
**cg26969288**	1	200379157	ZNF281;ZNF281;ZNF281;ZNF281;ZNF281;ZNF281	0.0278536	1.00610953778486	0.0838840447910916
**cg02768671**	6	169470822		-0.0391941	-0.0625771090795596	0.0838840447910916
**cg24185998**	5	176966237	FAM193B;FAM193B	-0.0530857	-0.0939262213901789	0.0838840447910916
**cg25798987**	10	73157222	CDH23;CDH23	0.0243414	0.613182884068121	0.0838840447910916
**cg18562663**	3	187455516	BCL6;BCL6	0.0114194	0.461380203729008	0.0838840447910916
**cg07031532**	15	64995684	OAZ2	0.0231399	0.862993791231587	0.0838840447910916
**cg00334349**	7	127928611		-0.0587361	-0.0969514939965768	0.0838840447910916
**cg24769167**	19	1205715	STK11	0.00801077	0.715531306777569	0.0838840447910916
**cg10783797**	7	150930536	CHPF2;CHPF2	0.0368857	0.702817402040935	0.0839041611329158
**cg12553947**	5	114486604	TRIM36;TRIM36;TRIM36	-0.0238952	-0.043879725477827	0.0839041611329158
**cg07124148**	8	103876083	AZIN1-AS1;AZIN1-AS1;AZIN1;AZIN1;AZIN1;AZIN1;AZIN1;AZIN1	0.0120733	0.903234594465224	0.0839041611329158
**cg08495027**	22	31364830	MORC2;TUG1	0.0115402	0.313021105191562	0.0839041611329158
**cg11902278**	10	29976135	SVIL	-0.0511763	-0.0891753033892618	0.0839903740877992
**cg14913644**	19	11530065	RGL3;RGL3	0.0133908	0.431208047176713	0.0839903740877992
**cg07851057**	5	70751686	BDP1;BDP1	0.0342026	0.847180108786429	0.0839903740877992
**cg17004038**	12	109532235	ALKBH2;ALKBH2;ALKBH2;ALKBH2;ALKBH2	-0.0317473	-0.0526482920982228	0.0839903740877992

**cg18585246**	8	1041929		-0.0250456	-0.039203691725455	0.0839903740877992
**cg07483245**	15	23034598	NIPA2;NIPA2;NIPA2;NIPA2	0.0232644	0.538552204177276	0.0839903740877992
**cg02908709**	3	111578238	PHLDB2;PHLDB2;PHLDB2;PHLDB2;PHLDB2;PHLDB2	0.0103663	0.723113188958304	0.0839903740877992
**cg11487977**	19	9879695	ZNF846	0.00689817	0.453544684595543	0.0839903740877992
**cg02386983**	2	86669112	KDM3A;KDM3A	0.0159127	0.646635534409444	0.0839903740877992
**cg12118798**	7	155170624		0.0147269	0.40561711789387	0.0839903740877992
**cg26095266**	2	10262787	RRM2;RRM2	0.0196987	0.61583360012115	0.0839903740877992
**cg16195284**	14	24112375	DHRS2;DHRS2;DHRS2;DHRS2	-0.0468715	-0.0958575475711064	0.0839903740877992
**cg04265971**	4	2470698	RNF4	0.0265606	1.37616161531596	0.0839903740877992
**cg03438090**	2	239143093		0.0178874	0.710600350307186	0.0839903740877992
**cg27245656**	18	48572727	SMAD4	-0.0702727	-0.130668036315451	0.0839903740877992
**cg05267587**	17	29233478	C17orf42	0.00568593	0.475340737252469	0.0839903740877992
**cg13190306**	11	18415923	LDHA;LDHA;LDHA;LDHA;LDHA	0.00948639	0.483846839892065	0.0839903740877992
**cg14998651**	2	73430289	NOTO	0.0126717	0.490746204372189	0.0839903740877992
**cg21778743**	2	24397871	FAM228A	0.0968341	0.678542131224148	0.0839903740877992
**cg17742155**	9	117350088	ATP6V1G1;ATP6V1G1	0.00672984	0.511269410818003	0.0839903740877992
**cg06485000**	17	78881721	RPTOR;RPTOR	0.0101815	0.0153908617372161	0.0839903740877992
**cg06349981**	2	10443745	HPCAL1;HPCAL1	0.046019	0.911222740029476	0.0839903740877992
**cg25869267**	1	164319588		-0.0665066	-0.120147617988513	0.0839903740877992
**cg21498653**	3	55521852	WNT5A	0.00663123	0.516488949783199	0.0839903740877992
**cg17886847**	14	23045367	DAD1	-0.0208915	-0.031991412992349	0.0839903740877992
**cg26662703**	1	196856446	CFHR4;CFHR4;CFHR4	-0.0452774	-0.0861309348619261	0.0839903740877992
**cg13017229**	16	58663923	CNOT1;CNOT1;CNOT1;CNOT1	0.0151568	0.782608446579742	0.0839903740877992
**cg07102474**	17	61499248	TANC2	0.0114883	0.0171709829549236	0.0839903740877992
**cg19831351**	5	153589239	GALNT10	-0.0383838	-0.0623664000084203	0.0839903740877992
**cg00834319**	14	64970752	ZBTB25;ZBTB1;ZBTB1	0.0163815	0.752915360429391	0.0839903740877992
**cg07172280**	9	140009455	DPP7	0.00571618	0.482903636609586	0.0839903740877992
**cg07904175**	19	16911472	NWD1;NWD1	-0.0278351	-0.0428866057300234	0.0839903740877992
**cg26791384**	16	77246057	SYCE1L	0.0259896	0.789634819227007	0.0839903740877992
**cg19526740**	19	58142253		-0.0434507	-0.095755126194666	0.0839903740877992
**cg23256829**	10	134229731	PWWP2B;PWWP2B	-0.0148224	-0.0222893593430425	0.0839903740877992
**cg03509671**	3	101497876	FAM55C;FAM55C	0.034198	1.29127137731632	0.0839903740877992
**cg13353015**	1	34547254	CSMD2	-0.0365372	-0.0644073235751115	0.0839903740877992
**cg17760982**	11	13740479	FAR1	-0.0696517	-0.129205218891225	0.0839903740877992
**cg06753949**	19	15334309		0.042828	0.958751521890454	0.0839903740877992
**cg02039732**	3	73039895		-0.0446432	-0.073495766430748	0.0839903740877992
**cg22751438**	9	115333218	KIAA1958;KIAA1958;KIAA1958	-0.0757002	-0.163734432811055	0.0839903740877992
**cg18809729**	11	1302981	TOLLIP	0.00334702	0.004885061618778	0.0839903740877992
**cg18490813**	6	7625602		-0.0592932	-0.106090410093516	0.0839903740877992
**cg00523590**	1	213123698	VASH2;VASH2;VASH2;VASH2	0.0148448	0.594855082668019	0.0839903740877992
**cg10865444**	21	43136563	C21orf129;NCRNA00112	0.00628661	0.00981437682035521	0.0839903740877992
**cg04823492**	10	97471301	ENTPD1	-0.0755024	-0.146208654826359	0.0839903740877992
**cg25707994**	7	157129685	DNAJB6;DNAJB6	0.037893	0.98024329818952	0.0839903740877992
**cg19268708**	1	2837518		-0.0392097	-0.0686726956917393	0.0839903740877992
**cg11576513**	6	114663692		0.0370017	1.40462291864273	0.0839903740877992
**cg05065765**	3	38206519	OXSR1	0.0158687	0.632186132198928	0.0839903740877992
**cg23697417**	20	982991	RSPO4;RSPO4	0.0204068	0.927642150846237	0.0839903740877992
**cg02717503**	7	157213736		0.0571536	0.0945352499908809	0.0839903740877992
**cg20127188**	22	21996426	SDF2L1	0.0228381	0.982622428966665	0.0839903740877992
**cg15233711**	11	13299315	ARNTL;ARNTL;ARNTL	0.0133494	0.415788115648002	0.0839903740877992
**cg15935527**	18	74843715	MBP;MBP	0.00976965	0.455120149237877	0.0839903740877992
**cg04737820**	4	2010918	NELFA;NELFA	0.0308793	0.597771466730294	0.0839903740877992
**cg07086074**	19	58049756	ZNF549;ZNF549	-0.032112	-0.049280223014192	0.0839903740877992
**cg10621559**	9	131038338	SWI5;GOLGA2	0.0236805	1.02869384551871	0.0839903740877992
**cg21010566**	7	105925537	NAMPT;NAMPT	0.00551169	0.37953562977184	0.0839903740877992
**cg24294159**	1	150947566	LASS2;LASS2	0.0457155	1.22615668864335	0.0839903740877992
**cg13250541**	2	27435095	SLC5A6;SLC5A6;ATRAID;ATRAID;ATRAID;SLC5A6	0.0216713	1.01259565184609	0.0839903740877992
**cg00713252**	1	9188947	GPR157	0.0112546	0.437140051800659	0.0839903740877992
**cg24823920**	20	9287042	PLCB4;PLCB4;PLCB4	-0.0356224	-0.0580700564045792	0.0839903740877992
**cg14283194**	7	91763737	CYP51A1;CYP51A1;CYP51A1	0.0245913	1.03433035865741	0.0840028948184367
**cg02563827**	1	8003721	TNFRSF9	-0.0529311	-0.0852224923711474	0.0841102063847659
**cg08249556**	21	30365104	RNF160	0.0142259	0.498383919646073	0.0841102063847659
**cg13047843**	7	101386679		0.0168501	0.95900118786769	0.0841102063847659
**cg19118037**	17	6332115	AIPL1;AIPL1;AIPL1;AIPL1;AIPL1;AIPL1;AIPL1;AIPL1	-0.0542965	-0.108208603922138	0.0841102063847659
**cg16396954**	14	72398856	RGS6	0.0424897	0.501860250397312	0.0841102063847659
**cg12492289**	21	47269180	PCBP3;PCBP3	-0.0830225	-0.177524976637053	0.0841102063847659
**cg06243263**	6	84981475		-0.0302175	-0.0482976512833736	0.0841102063847659
**cg24260662**	2	133281441	GPR39	-0.0498689	-0.0927652338189243	0.084133541414546
**cg01807303**	11	73495319		-0.080926	-0.23833358130163	0.084133541414546
**cg06717492**	7	65447339	GUSB	0.0283527	0.716080594546715	0.084133541414546
**cg18556005**	15	41952440	MGA;MGA	0.00800368	0.345430549992357	0.0842323360157694
**cg00580022**	22	17639482	CECR4;CECR5;CECR5;CECR4	0.0344722	0.597881079013016	0.0842323360157694
**cg22169206**	19	56136215	ZNF784	0.00573005	0.296697693694097	0.0842323360157694
**cg10432104**	9	137967290	OLFM1;OLFM1;OLFM1;OLFM1	0.0169003	0.507596041012502	0.0843818716779792
**cg14459319**	4	144206376		-0.0735792	-0.153692321030872	0.0843818716779792
**cg19926630**	4	1401854		0.0069985	0.334083020605987	0.0843818716779792
**cg21173351**	5	4135244		-0.0922159	-0.156380534020476	0.0843818716779792
**cg18127410**	11	9113184	SCUBE2;SCUBE2	0.0547202	0.798977769319846	0.0843818716779792
**cg03947261**	12	64515314	SRGAP1	-0.0559914	-0.0995744963925408	0.0843818716779792
**cg14230859**	2	157291402	GPD2;GPD2	-0.0638872	-0.114038715159106	0.0843818716779792
**cg16997101**	1	21864590	ALPL;ALPL;ALPL	-0.0631718	-0.123273504867793	0.0844064739243959
**cg11520985**	6	7313314	SSR1	0.0184634	0.827375292067798	0.0844064739243959

**cg00809477**	1	10898189		-0.0126971	-0.0193746282656137	0.0844064739243959
**cg06161720**	14	35099471	SNX6;SNX6	0.0175459	0.480430871542096	0.0844064739243959
**cg04382250**	20	5730330	C20orf196	-0.0717772	-0.171701692843536	0.0844064739243959
**cg26541587**	10	43950885	ZNF487	0.0218544	0.740752130196303	0.0844064739243959
**cg18073970**	1	184006765	GLT25D2;GLT25D2	0.0137443	0.410905468617892	0.0844064739243959
**cg02460223**	1	8398664	SLC45A1	-0.0251411	-0.0389405834882281	0.0844122492667772
**cg12754733**	19	11485436	C19orf39	0.00741673	0.387716692912154	0.0844442875910541
**cg06391985**	9	139304722	SDCCAG3;SDCCAG3;SDCCAG3;PMPCA;PMPCA;PMPCA	0.0196457	0.642644907836835	0.0844442875910541
**cg11195494**	3	46923433	PTH1R	0.0359731	0.522811179430646	0.0844790895055961
**cg26978822**	16	56622779	MT3	-0.0187593	-0.0287230689325452	0.0844790895055961
**cg01035038**	14	88459686	GALC;GALC;GALC;GALC	0.00743631	0.351590824885826	0.0844790895055961
**cg17001032**	1	33386151		-0.0455034	-0.0881086884039936	0.0844790895055961
**cg02510388**	3	182511451	ATP11B;ATP11B	0.00911881	0.424299290597567	0.0845276769472141
**cg15016513**	10	126150256	LHPP;LHPP	0.018503	0.670785676005993	0.0845468821586279
**cg07279963**	3	61546526	PTPRG	0.0266332	0.740931972501924	0.084612062780541
**cg21800309**	6	99797222	C6orf168	0.0129441	0.545006007399293	0.084612062780541
**cg20312087**	8	145754193	MGC70857	0.00769617	0.335601422615079	0.084612062780541
**cg24476709**	4	74089886	ANKRD17;ANKRD17;ANKRD17	-0.0448623	-0.0873810491211875	0.084612062780541
**cg16289299**	16	89939807	TCF25	0.00396404	0.366665280112697	0.084612062780541
**cg05766943**	6	107394007	BEND3	-0.0243576	-0.0374268604849545	0.084612062780541
**cg08300346**	11	113932106	ZBTB16;ZBTB16	0.0165044	0.358572797213731	0.084612062780541
**cg05253577**	5	167719262	WWC1;WWC1;WWC1	0.00417743	0.328398961996521	0.084612062780541
**cg24585451**	6	112684233		-0.0204647	-0.0312385861225429	0.084612062780541
**cg23962478**	22	50354086	PIM3	0.0183197	0.653703245515113	0.084612062780541
**cg11784395**	2	202946199	KIAA2012	-0.0574172	-0.111900320228939	0.084612062780541
**cg08244522**	20	23030533	THBD	0.0119093	0.446274528149962	0.084612062780541
**cg03057182**	1	46252470		-0.0759782	-0.125988592026013	0.084612062780541
**cg03820581**	1	25944147	MAN1C1;MAN1C1;MAN1C1;MAN1C1	0.0112926	0.531855887844112	0.084612062780541
**cg12740316**	8	68644694	CPA6	-0.0274258	-0.0433316540860784	0.084612062780541
**cg06447154**	15	93674560		-0.0571786	-0.109350983515377	0.084612062780541
**cg05356738**	10	8096319	FLJ45983;GATA3;GATA3;FLJ45983	0.0133379	0.632360647514776	0.084612062780541
**cg20567537**	19	10698078	AP1M2;AP1M2	-0.0376503	-0.394218459571251	0.084612062780541
**cg09665900**	20	45849713	ZMYND8;ZMYND8;ZMYND8;ZMYND8;ZMYND8;ZMYND8;ZMYND8;ZMYND8;ZMYND8;Z	-0.029756	-0.0484304651774986	0.084612062780541
**cg04474988**	10	131770171		0.066916	0.236596333645287	0.084612062780541
**cg02969418**	8	120780847	TAF2	-0.0473318	-0.0904442035293742	0.084612062780541
**cg04239375**	9	107526778	NIPSNAP3B	0.016262	0.386207476383573	0.084612062780541
**cg24855292**	13	25080267	PARP4	-0.0170347	-0.0259330574193259	0.084612062780541
**cg02926965**	6	30523975	PRR3;PRR3;GNL1	0.0224874	0.476221693512877	0.084612062780541
**cg15608397**	2	223163573	PAX3;PAX3;PAX3;PAX3;PAX3;PAX3;PAX3;PAX3;PAX3;CCDC140;PAX3;PAX3;PAX3;PA	0.0579477	0.361040213932168	0.084612062780541
**cg01927148**	5	36200909	NADK2;NADK2;NADK2;NADK2	-0.0664799	-0.108308518606888	0.084612062780541
**cg10773309**	1	32229411	BAI2	0.0180218	0.407597442232927	0.084612062780541
**cg11352190**	12	45270000	NELL2;NELL2;NELL2;NELL2;NELL2;NELL2	0.0341482	0.789096761653178	0.084612062780541
**cg05469759**	16	58498456	NDRG4;NDRG4;NDRG4	0.0224971	0.533861268326939	0.084612062780541
**cg13819586**	14	24685205	MDP1;MDP1	0.0356594	0.843643865796301	0.084612062780541
**cg16218089**	1	150516304		0.00384908	0.00564164197975366	0.084612062780541
**cg17463277**	3	179791243		-0.0350909	-0.069713266734958	0.084612062780541
**cg26291818**	8	61429582	RAB2A;RAB2A	0.0421891	0.529084579379651	0.084612062780541
**cg17730500**	16	23569012	UBFD1;UBFD1;EARS2;EARS2;EARS2	0.0105348	0.621957248428384	0.084612062780541
**cg13906078**	7	105241469		-0.0563254	-0.230333767369068	0.084612062780541
**cg06662991**	6	3456581	SLC22A23	0.0138548	0.544105589562598	0.084612062780541
**cg12615557**	1	109046245		-0.16327	-0.403730308010828	0.084612062780541
**cg08446989**	11	66036511	RAB1B	0.0240212	0.709469171763452	0.084612062780541
**cg11636714**	8	42128950	IKBKB;IKBKB	0.0386098	0.5780364757342	0.084612062780541
**cg27054251**	1	104068093	RNPC3;LOC101928436;LOC101928436	0.0066324	0.435406595246232	0.084612062780541
**cg13701769**	13	111915506	ARHGEF7;ARHGEF7;ARHGEF7;ARHGEF7;ARHGEF7	0.0156381	0.0237421223220364	0.084612062780541
**cg23942310**	10	46117671	ZFAND4;ZFAND4;ZFAND4;ZFAND4	-0.045121	-0.0734671638646208	0.084612062780541
**cg04550070**	11	73694480	UCP2	0.0108922	0.565193399472936	0.084612062780541
**cg02081019**	5	92930813		0.0137681	0.642829822274765	0.084612062780541
**cg05098590**	10	111767379	ADD3;ADD3;ADD3	0.00508518	0.438615729004483	0.084612062780541
**cg06039108**	2	557490		-0.0117428	-0.0185115768223936	0.084612062780541
**cg07113322**	16	58034858	C16orf57;ZNF319	0.0267417	0.587439236987856	0.084612062780541
**cg15914307**	5	91624891		-0.0827757	-0.208528792122103	0.084612062780541
**cg01794812**	8	146027063	ZNF517	-0.055958	-0.109429543680838	0.084612062780541
**cg14988083**	4	1804492	FGFR3;FGFR3;FGFR3	-0.079641	-0.157481197463002	0.084612062780541
**cg24538029**	5	170846083	FGF18	0.00802501	0.501701794523441	0.084612062780541
**cg08068407**	19	4491713	HDGF2;HDGF2	0.00873718	0.013090284777088	0.084612062780541
**cg12683602**	17	1532220	SLC43A2	0.00627519	0.41189355191967	0.084612062780541
**cg12920602**	16	31084954	ZNF646;ZNF668;ZNF668	0.0101819	0.355204661218905	0.084612062780541
**cg01463565**	17	8926158	NTN1	0.012857	0.402183381208282	0.084612062780541
**cg17516809**	12	127547994		-0.0111562	-0.0165692770669588	0.084612062780541
**cg11799172**	10	31607441	ZEB1;ZEB1;ZEB1;LOC220930;ZEB1	0.00777836	0.562596763756322	0.084612062780541
**cg17959324**	19	42747065	GSK3A	0.00698789	0.675781473336339	0.084612062780541
**cg19086488**	1	43752185	C1orf210;C1orf210	-0.0665737	-0.120663694338681	0.084612062780541
**cg04383836**	17	9559049	USP43	-0.088791	-0.17322356463893	0.0846161741542491
**cg21742381**	1	151065206	GABPB2	-0.0283726	-0.0432474582783523	0.0846161741542491
**cg18770660**	11	65082070	CDC42EP2	0.0110639	0.564811697322316	0.0846161741542491
**cg14445171**	9	131419910	WDR34	-0.0943937	-0.168775124887524	0.084664026312105
**cg09156097**	5	138729283	LOC389333	0.0577811	0.909047564515433	0.084664026312105
**cg01635061**	6	33160234	COL11A2;COL11A2;COL11A2;COL11A2;COL11A2;COL11A2;COL11A2;COL11A2	0.0123493	0.414747303652874	0.0847201632052088
**cg16806919**	7	129705337		-0.0623861	-0.11543448741538	0.0847356131376682
**cg12575313**	21	33765297	URB1;C21orf119;URB1	0.0152798	0.536461800558244	0.0847356131376682
**cg01963240**	14	54421117	BMP4;BMP4;BMP4	0.0205567	0.810441671043231	0.0847356131376682

**cg11998173**	5	107007752	EFNA5	0.00686885	0.400671541518439	0.0847356131376682
**cg00300090**	3	4345167	SETMAR;SETMAR	0.044758	0.711077413594933	0.0847356131376682
**cg10577070**	17	17942837	GID4;ATPAF2	0.0209063	0.646127103670753	0.0847356131376682
**cg05277991**	3	69788295	MITF	-0.00432936	-0.242128685234001	0.0847356131376682
**cg09962379**	8	37928432		-0.0263059	-0.0428306920647438	0.0847356131376682
**cg02339888**	1	67862336	IL12RB2	-0.0579882	-0.137666014787818	0.0847356131376682
**cg08340583**	4	178302630		-0.0326929	-0.0515710411527625	0.0847356131376682
**cg06658816**	1	9242956		0.0146881	0.591824712047512	0.0847356131376682
**cg07279281**	3	46735319	ALS2CL	0.00851242	0.520694683325202	0.0847356131376682
**cg18987683**	3	160283058	KPNA4	0.0130201	1.04715822353622	0.0847356131376682
**cg00750934**	14	74263737	LOC100506476	0.00939825	0.0141692116458203	0.0847356131376682
**cg05587736**	16	56780751	NUP93	-0.0214897	-0.0328875960781537	0.0847356131376682
**cg02815785**	14	37063429		-0.0161102	-0.0246229964552876	0.0847356131376682
**cg19271031**	3	187722915		0.00878585	0.0129725452877342	0.0847356131376682
**cg02978503**	7	48144090	UPP1;UPP1;UPP1;UPP1;UPP1;UPP1	-0.0134704	-0.0206483514749948	0.0847356131376682
**cg26365938**	1	24645953	GRHL3;GRHL3	0.00874729	0.338287482589174	0.0847356131376682
**cg24351167**	6	12749567	PHACTR1	0.0222847	0.681171391954102	0.0847356131376682
**cg16031872**	3	142297641	ATR;ATR	0.00679491	0.437141123323782	0.0847356131376682
**cg24109980**	10	50818707	SLC18A3;SLC18A3;CHAT	0.0402809	0.781615798803201	0.0847356131376682
**cg08420415**	22	38477802	SLC16A8	0.0258848	0.47703187354563	0.0847356131376682
**cg01505700**	17	4853232	ENO3;ENO3;PFN1	0.00880318	0.569372087237164	0.084785309378375
**cg12426293**	18	8612540	RAB12	0.0120186	0.0181199471451331	0.084785309378375
**cg21707136**	11	2421773	TSSC4;TSSC4;TSSC4;TSSC4;TSSC4;TSSC4;TSSC4;TSSC4	0.0325146	1.16359844791259	0.084785309378375
**cg19188060**	3	184017239	PSMD2	0.0218841	0.522587035980064	0.084785309378375
**cg08595804**	13	112712424		0.0224692	0.58670196927976	0.084785309378375
**cg25112966**	3	72792066		-0.067358	-0.118991794863527	0.084785309378375
**cg18735356**	1	112298505	DDX20;DDX20;LOC101928718;FAM212B	0.0154145	0.570102716750337	0.084785309378375
**cg16246698**	12	133263907	PXMP2;POLE;POLE	0.0197624	0.672184523610034	0.084785309378375
**cg20037470**	17	30185765	C17orf79	0.0133569	0.249864629156349	0.084785309378375
**cg08304120**	11	65381096	MAP3K11	0.0153468	0.819629966305313	0.084785309378375
**cg27591595**	17	57732823	CLTC;CLTC	-0.0740377	-0.1591174361721	0.084785309378375
**cg11656992**	17	79818741	P4HB	0.0232591	0.759685030434842	0.084785309378375
**cg01031146**	3	184081222	POLR2H;POLR2H	0.00373198	0.369798064437289	0.084785309378375
**cg02854791**	7	157776477	PTPRN2;PTPRN2;PTPRN2;PTPRN2;PTPRN2	0.0179471	0.030230230306956	0.084785309378375
**cg21068576**	19	57875295	TRAPPC2P1;ZNF547	0.0344636	0.356714114053026	0.084785309378375
**cg23610453**	10	79470966		0.0348559	0.482699371468834	0.084785309378375
**cg16149238**	2	23608001	KLHL29	0.014991	0.58226696639567	0.084785309378375
**cg20307885**	7	27130090		-0.0467287	-0.33753103990088	0.084785309378375
**cg18330340**	2	24307512	TP53I3;TP53I3;TP53I3;TP53I3;FAM228B;FAM228B	0.0123467	0.661945959899942	0.084785309378375
**cg02083546**	12	118477739	WSB2	-0.0251245	-0.0394787667458502	0.084785309378375
**cg01840281**	5	75977657	IQGAP2;IQGAP2;IQGAP2;IQGAP2	-0.0543155	-0.103480835306149	0.084785309378375
**cg04084600**	2	235016302		-0.0887487	-0.196803131752561	0.084785309378375
**cg06196453**	2	109335786	RANBP2	0.0240839	0.709728411509363	0.084785309378375
**cg20349687**	2	88927127	EIF2AK3	0.0084648	0.552399187339018	0.084785309378375
**cg26946821**	17	35292390		0.00885395	0.559140117567075	0.084785309378375
**cg12998265**	3	156392708	TIPARP-AS1;TIPARP;TIPARP	0.0286218	0.524239602069331	0.084785309378375
**cg11204311**	1	235293522	TOMM20	-0.0560364	-0.126793387475278	0.084785309378375
**cg02057463**	21	42789922		-0.0634933	-0.125723716317831	0.084785309378375
**cg11631334**	3	196730272	MFI2	0.0297694	0.496061107187885	0.084785309378375
**cg22209616**	15	68569728	FEM1B	0.008903	0.441413212058786	0.084785309378375
**cg12250496**	4	62066787		0.0176925	0.57203090940559	0.084785309378375
**cg06638463**	19	10530633	PDE4A	0.0339881	0.694266043456175	0.0848574265845052
**cg01085347**	1	119870666		0.0233212	0.6139453748129	0.084901181557511
**cg13865656**	10	92757657		-0.0555869	-0.0932009820079002	0.084901181557511
**cg06779945**	6	138188320	TNFAIP3	0.00596483	0.528806216089364	0.084901181557511
**cg11028747**	7	102104916	LRWD1;ALKBH4	0.00497174	0.379015860040477	0.084901181557511
**cg14277044**	17	1890733	RTN4RL1	-0.00352069	-0.00511751657589812	0.084901181557511
**cg17871537**	5	118788128	HSD17B4	0.00736392	0.527172274975102	0.084901181557511
**cg15554087**	1	8404216	SLC45A1	0.0235455	0.037036545411719	0.084901181557511
**cg18975376**	10	28967265	BAMBI	0.0120516	0.328098127097969	0.084901181557511
**cg25047469**	15	90198526	KIF7	0.0428549	0.55929695804996	0.084901181557511
**cg04142690**	10	7830753	KIN;KIN;ATP5C1;ATP5C1	0.00413924	0.334343270111904	0.084901181557511
**cg06567042**	3	197535273	LRCH3	-0.0513621	-0.0885044591046284	0.084901181557511
**cg24270182**	8	9046418		-0.00522964	-0.00769499629810237	0.0849255715218498
**cg10196372**	16	4322417	TFAP4	0.0174328	0.712046735233784	0.0849255715218498
**cg21877498**	5	139944509	APBB3;APBB3;SLC35A4;APBB3;APBB3	0.00996217	0.577753288251197	0.0849255715218498
**cg13223329**	11	133274220	OPCML	-0.0533656	-0.0835676551978196	0.0849255715218498
**cg02938651**	6	855390		-0.00353251	-0.00520726429404765	0.0849255715218498
**cg17537252**	3	56836203	ARHGEF3;ARHGEF3	0.0136309	0.466916076683746	0.0849255715218498
**cg25108914**	11	58826264	LOC283194	0.0389105	0.287975999586716	0.0849255715218498
**cg27430726**	7	30721990	CRHR2;CRHR2;CRHR2;CRHR2;CRHR2;CRHR2;CRHR2;CRHR2	0.011311	0.521767649408167	0.0849255715218498
**cg22300980**	11	57330100	UBE2L6;UBE2L6	-0.0349869	-0.0545198225423526	0.0849255715218498
**cg02523938**	16	34409092		-0.0488614	-0.128653064282963	0.0849255715218498
**cg13307690**	2	25620049	DTNB;DTNB;DTNB;DTNB;DTNB;DTNB;DTNB;DTNB	-0.0340807	-0.0547302632299998	0.0849255715218498
**cg26005766**	6	125475650	TPD52L1;TPD52L1;TPD52L1;TPD52L1;TPD52L1	0.0121424	0.755261674622638	0.0849255715218498
**cg11386686**	1	203274497	BTG2	0.0297698	1.05512549903966	0.0849255715218498
**cg09886619**	8	38253313	LETM2;LETM2;LETM2;LETM2;LETM2	-0.061259	-0.104923449000059	0.0849255715218498
**cg09789536**	1	896226	KLHL17	0.0541413	0.22717425119714	0.0849255715218498
**cg07964213**	3	138014757	ARMC8;TXNDC6	-0.0224892	-0.0343656914261421	0.0849255715218498
**cg25781121**	3	48282641	ZNF589;ZNF589	0.015561	1.06991561905821	0.0849255715218498
**cg20245503**	12	58026705	B4GALNT1;B4GALNT1	0.00603154	0.449689229447217	0.0849255715218498
**cg12947510**	9	119449380	ASTN2;TRIM32;ASTN2;ASTN2;ASTN2;ASTN2;TRIM32	0.00763217	0.324485527368699	0.0849255715218498

**cg25208919**	5	124704131		-0.0368841	-0.0609105098650921	0.0849255715218498
**cg00463109**	1	110722603	SLC6A17	-0.0575138	-0.102437236494354	0.0849255715218498
**cg18208654**	17	41174520	VAT1	0.00789929	0.441885381445395	0.0849255715218498
**cg10441013**	1	160039990	KCNJ10	0.0145335	0.486621023601117	0.0849255715218498
**cg25876975**	15	77363192	TSPAN3;TSPAN3;TSPAN3	0.0571776	0.213949976422538	0.0849255715218498
**cg19656070**	17	3571978	TMEM93;TAX1BP3;TMEM93	0.0217728	0.676160169864188	0.0849255715218498
**cg01526745**	17	32448038	ASIC2	-0.0627376	-0.110739687924328	0.0849255715218498
**cg23048358**	17	9940652	GAS7;GAS7	-0.00611654	-0.00897088920547826	0.0849255715218498
**cg09630385**	10	88506688		-0.0379275	-0.0625005200942491	0.0849255715218498
**cg16507755**	21	36164414	RUNX1;RUNX1	-0.019841	-0.0308505724982541	0.0849656803197816
**cg15644003**	11	63438952	ATL3;ATL3	0.0122008	0.51202650694677	0.0849656803197816
**cg13645565**	16	66959579	RRAD;RRAD	0.0450487	0.716126643870319	0.0849970960961894
**cg03067068**	16	19729774	C16orf88;IQCK	0.0159206	0.505992677198746	0.0849970960961894
**cg22480244**	1	220445929	RAB3GAP2	0.00720055	0.49004323421868	0.0849970960961894
**cg13932954**	1	25255805	RUNX3;RUNX3	0.0227532	0.775045108269114	0.0850317240491275
**cg13946335**	3	20383419	LOC101927829	-0.0304023	-0.0474578600020399	0.0850317240491275
**cg24407859**	22	43411083		0.0267052	0.652280788147366	0.0850317240491275
**cg12761536**	22	38245354	EIF3L;EIF3L	0.00513018	0.446372415315109	0.0850569729187668
**cg10477989**	11	68039601	C11orf24	0.0476399	0.621254780927399	0.0850877653672594
**cg05787952**	16	734667	JMJD8	0.0367921	0.562178927770991	0.0850877653672594
**cg12320972**	18	19192160	SNRPD1;SNRPD1	0.0110868	0.47136859062651	0.085130804986105
**cg18436128**	14	65439236	RAB15	0.0104338	0.6030620810418	0.085130804986105
**cg02330195**	10	73342047	CDH23;CDH23;CDH23;CDH23;CDH23	-0.0530588	-0.117521154603781	0.085130804986105
**cg14510105**	16	18812794	ARL6IP1;ARL6IP1	0.0105248	0.611411543760673	0.085130804986105
**cg13628367**	7	48429659	ABCA13	-0.0561567	-0.598594925518236	0.085130804986105
**cg19270118**	1	154553118		-0.0389814	-0.0638498094506196	0.085130804986105
**cg13701855**	19	52532420	ZNF614	-0.0414902	-0.178433484793812	0.085130804986105
**cg19135690**	11	86620552		-0.0198457	-0.0304665542542186	0.085130804986105
**cg27200833**	15	74421909	ISLR2;LOC283731;ISLR2;ISLR2;ISLR2;ISLR2	0.0188493	0.701394603634314	0.085130804986105
**cg23433430**	4	41362520	LIMCH1;LIMCH1;LIMCH1;LIMCH1;LIMCH1	0.00656069	0.563411287425692	0.085130804986105
**cg05391643**	1	27214630	GPN2	-0.06376	-0.119308349193221	0.085130804986105
**cg04347624**	3	101498377	FAM55C;FAM55C;FAM55C	0.0380975	0.942307785515412	0.085130804986105
**cg21206054**	10	51827272	FAM21A;FAM21A;FAM21EP	-0.0433977	-0.541449718827203	0.085130804986105
**cg27199414**	22	40901737	MKL1;MKL1;MKL1	-0.0706681	-0.186211329441971	0.085130804986105
**cg18685243**	12	58015069	SLC26A10	0.021916	0.954528100290109	0.085130804986105
**cg00259882**	11	70049130	FADD	0.0112942	0.540162536419463	0.085130804986105
**cg12668711**	17	48624393	SPATA20;SPATA20;SPATA20	0.01293	0.749019792807006	0.085130804986105
**cg12025821**	8	145133465	EXOSC4	0.0218552	0.57225280323658	0.085130804986105
**cg18467502**	16	57144407	CPNE2	-0.0479318	-0.0872025370005481	0.085130804986105
**cg04496233**	11	64863659	C11orf2	0.0117914	0.43187085076404	0.085130804986105
**cg06671069**	10	91174283	IFIT5	0.0197773	0.630349250429534	0.0851709366001437
**cg18609779**	4	37455499	C4orf19	0.0375313	0.685666450236602	0.085190293948191
**cg19190632**	10	97022649	PDLIM1	-0.0708312	-0.146639488070325	0.085190293948191
**cg03865041**	16	71842666	AP1G1;AP1G1;AP1G1;AP1G1	0.0605445	0.654869194792888	0.085190293948191
**cg03600868**	2	54826020	SPTBN1;SPTBN1	-0.0717462	-0.137817762033358	0.085190293948191
**cg09174009**	1	27216796	GPN2;GPN2	0.00437665	0.631283506725758	0.085190293948191
**cg14849727**	4	56719908	EXOC1;EXOC1;EXOC1;EXOC1;EXOC1;EXOC1	0.0159345	0.800293500524267	0.0852126565551805
**cg00076353**	20	442752	TBC1D20	0.0182954	0.62266737711325	0.0852126565551805
**cg12996305**	17	2295923	MNT	0.0405428	0.658840157486968	0.0852126565551805
**cg14408412**	7	111776496	DOCK4	0.00649472	0.00958055085890182	0.0852126565551805
**cg13199784**	9	136283271	REXO4;REXO4;REXO4;REXO4;REXO4;REXO4;REXO4;ADAMTS13	0.0195613	0.66130462879414	0.0852367956617704
**cg06058618**	1	155057452	EFNA3	0.0172349	0.47535742126852	0.0852718775188369
**cg05751059**	17	79848657	ALYREF;ANAPC11;ANAPC11;ANAPC11;ANAPC11;ANAPC11;ANAPC11;ANAPC11;ANAI	0.0367175	1.60816369011996	0.0853069333427688
**cg06067302**	22	40766481	SGSM3	0.0100293	0.84675374122985	0.085323384220766
**cg02520077**	1	156163116	SLC25A44;SLC25A44;SLC25A44;SLC25A44;SLC25A44	-0.0327732	-0.0589756200327238	0.085323384220766
**cg22395811**	4	164945455	MARCH1	-0.0491493	-0.0920634056012912	0.0853534947117769
**cg09149379**	9	131038181	SWI5;GOLGA2	0.0479517	1.80421152156068	0.0853534947117769
**cg19377812**	7	130353647	TSGA13;TSGA13;COPG2;COPG2	0.0498273	0.51444703287057	0.08536060172747
**cg18985971**	1	45265788	PLK3	0.0135785	0.457452794963261	0.08536060172747
**cg27343747**	9	130548282	CDK9;MIR2861	0.0419201	0.355764107411106	0.08536060172747
**cg07189338**	6	41040461	NFYA;NFYA;OARD1	0.0331173	1.03920367265374	0.08536060172747
**cg16294310**	22	50629110	TRABD	0.0245054	0.664637333454024	0.08536060172747
**cg26174797**	2	27363557	C2orf53	-0.072699	-0.159247679920262	0.08536060172747
**cg12383580**	17	48167096	ITGA3;ITGA3	-0.0220512	-0.0350619471097303	0.08536060172747
**cg01636413**	13	25321336		0.0176837	0.434503461494158	0.08536060172747
**cg27265499**	16	85699474	KIAA0182;KIAA0182	0.00757639	0.0111880453021659	0.08536060172747
**cg04117076**	5	88991074		-0.102811	-0.245734681389713	0.08536060172747
**cg00866986**	17	17942403	ATPAF2;ATPAF2;C17orf39	0.0107885	0.464652199985333	0.0853830660820548
**cg14231674**	9	35657560	CCDC107	0.0337256	0.24649071967797	0.0853859782826372
**cg04450857**	10	119296756	EMX2OS	0.0170111	0.458566180077479	0.0853859782826372
**cg01265653**	11	96190436		-0.0931282	-0.175258644964937	0.0853859782826372
**cg05430257**	12	12218620		-0.0449105	-0.0799647245426283	0.0853859782826372
**cg25846336**	15	60293554		-0.054481	-0.28656878617789	0.0853859782826372
**cg21415305**	3	9851855	TTLL3	0.0374695	0.613069963139213	0.0853859782826372
**cg23318713**	10	75670697	PLAU;C10orf55;PLAU	0.0138882	0.65829746128766	0.0853859782826372
**cg03497182**	2	76998734	LRRTM4;LRRTM4	-0.0360549	-0.0594458022508987	0.0853859782826372
**cg11962201**	20	33680799	TRPC4AP;TRPC4AP	0.0199037	0.578462414050975	0.0853859782826372
**cg01043997**	6	30689219	TUBB	0.0400131	0.410492491654461	0.0853859782826372
**cg00299070**	1	237946534	RYR2	-0.0337146	-0.0521843925907607	0.0853920702246093
**cg18320648**	2	152266336	RIF1	0.0520092	0.38142561602339	0.0854298564611327
**cg17840051**	19	12150028		-0.107915	-0.212232607623093	0.0854298564611327
**cg20037575**	15	96886839		0.0373574	0.36961371610807	0.0854298564611327

**cg10247061**	13	53065411	LOC220115	-0.0716379	-0.174060916310077	0.0854298564611327
**cg15967188**	4	2470700	RNF4	0.0226155	1.2420523299089	0.0854298564611327
**cg00223135**	15	43799245	TP53BP1	-0.0304165	-0.0525155543049668	0.0854298564611327
**cg04891921**	3	134514173	EPHB1	0.0201048	0.466244539751808	0.0854298564611327
**cg12042864**	7	99066260		-0.0453119	-0.0746857981595283	0.0854298564611327
**cg01998755**	7	99698536	MCM7;AP4M1;MCM7;MCM7	0.0296746	0.951901681845784	0.0854298564611327
**cg10719673**	19	40933298	SERTAD1	-0.0640193	-0.111877677422505	0.085444585709005
**cg01218180**	6	168079427		0.0270118	0.620586081511525	0.085444585709005
**cg19788417**	20	30027638	DEFB123	-0.093645	-0.209844530037269	0.0854591640587902
**cg17015033**	8	37620039	PROSC	0.0108422	0.653984446801714	0.0854591640587902
**cg23192120**	11	45921134	MAPK8IP1	0.0321883	0.611812248216262	0.0854598537623731
**cg19864539**	22	25747688	LRP5L;LRP5L	0.00237323	0.00346620129428112	0.0855190481114364
**cg04576568**	2	220377822	ACCN4;ACCN4	0.0122578	0.463260710219146	0.0855192750488044
**cg18391694**	1	43250900		0.0275801	0.41333319358724	0.0855192750488044
**cg06915098**	2	220384302	ASIC4	-0.0588583	-0.111495528050491	0.0855192750488044
**cg07141215**	16	4308070	TFAP4	-0.0457699	-0.0741629142802773	0.0855192750488044
**cg09348431**	8	144898112	SCRIB;SCRIB	0.0302988	0.744519773421304	0.0855192750488044
**cg21543987**	7	1272486	UNCX	0.0343296	1.34338411397281	0.0855192750488044
**cg15157403**	1	234880737		-0.0680902	-0.173202116574668	0.0855192750488044
**cg09440905**	17	20094629	SPECC1;SPECC1;SPECC1;SPECC1;SPECC1;SPECC1	-0.0581646	-0.117435999331006	0.0855192750488044
**cg16506959**	4	83404135		-0.0487162	-0.0832627802324026	0.0855192750488044
**cg14158601**	17	8055831	PER1	0.016016	0.553042779958306	0.0855192750488044
**cg15555727**	8	144692268	PYCRL	0.00622087	0.518986246868206	0.0855192750488044
**cg09324514**	16	58498710	NDRG4;NDRG4;NDRG4	0.0319918	0.434305177532388	0.0855192750488044
**cg00479770**	11	111289721	LOC100132078	-0.0342881	-0.0540541456025405	0.0855192750488044
**cg23130076**	12	70759920	KCNMB4	0.00499475	0.434372277575667	0.0855192750488044
**cg16015488**	9	35707914	TLN1	-0.032132	-0.0501843887392895	0.0855192750488044
**cg22395573**	17	79830134	ARHGDIA	0.00676045	0.405206527804799	0.0855192750488044
**cg06551828**	2	242141868	ANO7	0.00718933	0.0109548898389657	0.0855192750488044
**cg25791620**	17	1472984		-0.0125148	-0.0194420071504617	0.0855192750488044
**cg20467418**	4	3297777		-0.0153421	-0.0231737934240408	0.0855192750488044
**cg21687827**	9	130890548	PTGES2;PTGES2-AS1;PTGES2;PTGES2;PTGES2;PTGES2	0.0745665	1.60600038492757	0.0855192750488044
**cg25952423**	2	48542335	FOXN2	0.0139118	0.409520984666328	0.0855192750488044
**cg26224725**	7	74489898	WBSCR16	0.0118688	0.629008045240634	0.0855192750488044
**cg15504469**	1	44457406	CCDC24;CCDC24	0.0128146	0.468692139732084	0.0855192750488044
**cg07179556**	14	100663534		-0.0396505	-0.0667912628106074	0.0855192750488044
**cg11928198**	1	151137319	SCNM1;LYSMD1;LYSMD1	-0.0492924	-0.0908732499196398	0.0855192750488044
**cg20349598**	7	1962389	MAD1L1;MAD1L1;MAD1L1	-0.0307137	-0.0533166561340115	0.0855192750488044
**cg17910679**	17	79981754	STRA13;LRRC45	0.0226366	0.469292115663466	0.0855192750488044
**cg03146981**	16	70719277	MTSS1L	0.0302025	0.696700389610499	0.0855192750488044
**cg23242862**	8	65491152	LOC401463	0.0119118	0.501568038122363	0.0855192750488044
**cg00009834**	1	1347671		-0.0316497	-0.0524563488502964	0.0855192750488044
**cg17278864**	17	12877321	RICH2	0.0113967	0.31000285469814	0.0855192750488044
**cg04041707**	5	43018491	LOC648987;LOC648987;LOC648987;LOC648987;LOC648987	0.00568084	0.719512167286785	0.0855192750488044
**cg16845708**	1	22379018	CDC42;CDC42;CDC42	0.0182024	0.810428893829624	0.0855192750488044
**cg17543177**	12	29525010	ERGIC2	-0.0319475	-0.0596369299857999	0.0855192750488044
**cg07247810**	1	220684758		-0.0182733	-0.028853321412045	0.0855192750488044
**cg05224707**	5	171881549	SH3PXD2B	0.0291701	0.4454270435903	0.0855192750488044
**cg14226678**	19	2841332	ZNF555	0.00812312	0.476357307689929	0.0855192750488044
**cg01273393**	1	159996368		-0.0389963	-0.0618435680895882	0.0855192750488044
**cg27575052**	16	29855904	MVP;MVP	0.00769272	0.0114417220173007	0.0855192750488044
**cg15182583**	1	114355080	RSBN1;AP4B1-AS1;RSBN1;RSBN1	0.0160256	1.0448225012379	0.0855192750488044
**cg11193322**	2	129347608		0.011122	0.0172727297438109	0.0855192750488044
**cg01140579**	3	48541502	SHISA5	0.0432258	0.488739296379796	0.0855192750488044
**cg12456794**	3	148523344		-0.0392429	-0.0646579196171596	0.0855192750488044
**cg25984973**	20	62289569	RTEL1;RTEL1	0.0380397	0.853617537576357	0.0855192750488044
**cg14616251**	1	24648696	GRHL3;GRHL3;GRHL3	0.0127311	0.457672304148803	0.0855192750488044
**cg03503802**	22	46933237	CELSR1	0.015688	0.674083441487746	0.0855192750488044
**cg07981845**	10	104154074	NFKB2;NFKB2;NFKB2	0.00535293	0.309422911704373	0.0855192750488044
**cg15404157**	7	140933		-0.0483626	-0.0873389984475167	0.0855192750488044
**cg15831663**	4	131729521		-0.0599234	-0.104806991322195	0.0855192750488044
**cg06596689**	7	139875956	LOC100134229;JHDM1D	0.01475	0.605074300473111	0.0855192750488044
**cg05693527**	3	71114114	FOXP1;FOXP1;FOXP1;FOXP1;FOXP1;FOXP1;FOXP1;FOXP1	0.0546768	0.388607767116064	0.0855192750488044
**cg26079854**	16	52607602	CASC16	-0.063529	-0.122195669474684	0.0855192750488044
**cg04167819**	14	73395839	DCAF4;DCAF4;DCAF4;DCAF4;DCAF4	-0.0293787	-0.0495002914473858	0.0855192750488044
**cg03670816**	5	53606426	15,00 ARL	0.035588	0.28623013465392	0.0855192750488044
**cg08398554**	9	71736409	TJP2	0.0150926	0.914310607426757	0.0855192750488044
**cg13716074**	7	65215894	CCT6P1	0.0115624	0.319346063859078	0.0855192750488044
**cg12073865**	6	90121279	RRAGD	0.0257461	0.669659118125108	0.0855192750488044
**cg27598114**	13	45914733	TPT1;TPT1;TPT1-AS1;TPT1	0.0178793	0.511770998055929	0.0855192750488044
**cg09749205**	2	198311980		-0.0533128	-0.090690717756403	0.0855192750488044
**cg00475427**	9	140019538		-0.0469496	-0.0909821335987334	0.0855192750488044
**cg07364676**	22	29137974	CHEK2;HSCB;CHEK2;CHEK2	0.040764	0.794176880786662	0.0855192750488044
**cg26237050**	14	58754200	PSMA3-AS1;PSMA3-AS1	-0.0683433	-0.17371520762627	0.0855192750488044
**cg14428767**	6	149867359	PPIL4	0.0206455	0.850781774514552	0.0855192750488044
**cg08363518**	19	42905128		-0.0571848	-0.106650766239935	0.0855192750488044
**cg18353563**	3	127390935	ABTB1;PODXL2;ABTB1;ABTB1	0.0144945	0.484521650063692	0.0855192750488044
**cg23867624**	6	1610197	FOXC1	0.00572037	0.392293592233346	0.0855192750488044
**cg14239515**	19	38885242	SPRED3	0.0392934	0.881631436717681	0.0855192750488044
**cg20474201**	10	88228042	WAPAL	-0.0837158	-0.146731180144507	0.0855192750488044
**cg02879471**	6	111135854	CDK19	0.00942727	0.52299091239575	0.0855192750488044
**cg23035058**	10	133982861	JAKMIP3	-0.0302265	-0.0473891289343606	0.0855192750488044

**cg00647258**	22	19435555	C22orf39;C22orf39;C22orf39;C22orf39	0.00409343	0.432619437413634	0.0855192750488044
**cg08809260**	2	177054140	HOXD1	0.0632161	1.04037059804988	0.0855192750488044
**cg13056495**	7	134143249	AKR1B1	0.00521954	0.399986100448287	0.0855192750488044
**cg05956749**	9	34664179	CCL27	0.0109795	0.0164589184262911	0.0855192750488044
**cg14250846**	14	97061527		-0.0148907	-0.0230782273137014	0.0855192750488044
**cg22711111**	6	30139979	TRIM15	0.00912265	0.33185124965481	0.0855192750488044
**cg04112019**	11	2165136	IGF2AS;INS-IGF2;IGF2;IGF2AS	0.0051616	0.310460760901732	0.0855192750488044
**cg14041283**	9	17906338		0.0288142	0.705037109540893	0.0855192750488044
**cg19641554**	16	20912025	LYRM1;LYRM1;DCUN1D3;LYRM1;LYRM1;LYRM1;LYRM1;LYRM1;LYRM1;LYRM1;LYRM	0.00331889	0.47722056613384	0.0855192750488044
**cg21328954**	10	14880024	HSPA14;HSPA14;HSPA14;CDNF	0.0314166	1.07537601242545	0.0855192750488044
**cg11381843**	3	142681401	PAQR9	-0.00871661	-0.0129551859292501	0.0855192750488044
**cg16658737**	19	37064572	ZNF529;ZNF529;ZNF529	0.0335886	0.478510735700769	0.0855192750488044
**cg09949152**	12	118573731	PEBP1	0.0185475	0.913599239852041	0.0855192750488044
**cg05137358**	13	37006127	CCNA1;CCNA1;CCNA1;CCNA1;CCNA1;CCNA1	0.00828286	0.446476773243308	0.0855192750488044
**cg25476221**	12	111807046	FAM109A	0.0111021	0.382689122576026	0.0855192750488044
**cg17663101**	3	49131524	QRICH1;QRICH1	0.0359396	0.940158636946368	0.0855192750488044
**cg13462004**	19	7005350	FLJ25758	-0.036566	-0.0629791167814796	0.0855192750488044
**cg13129662**	17	48227708	PPP1R9B	0.0390236	0.939818379583971	0.0855192750488044
**cg13904199**	9	86594091	HNRNPK;HNRNPK;HNRNPK	0.0074633	0.383295010469337	0.0855192750488044
**cg00993799**	7	104653126	LOC100216545	0.032493	1.50545321653985	0.0855192750488044
**cg14779973**	10	115949613	TDRD1	-0.0103657	-0.0155545142485185	0.0855192750488044
**cg01918627**	1	167326211	POU2F1;POU2F1;POU2F1;POU2F1	-0.0511902	-0.0826432425071193	0.0855192750488044
**cg19517509**	12	120933726	DYNLL1;DYNLL1;DYNLL1	0.0146109	0.403138688286198	0.0855192750488044
**cg14153722**	1	206858799	MAPKAPK2;MAPKAPK2	0.0137662	0.830746944918956	0.0855192750488044
**cg06723057**	16	28549923	NUPR1;NUPR1	-0.0448136	-0.0983614615100035	0.0855192750488044
**cg20324516**	2	3137154		-0.0708698	-0.130592265988802	0.0855192750488044
**cg02203224**	12	123464087	ARL6IP4;ARL6IP4;OGFOD2;ARL6IP4;ARL6IP4	-0.0233822	-0.0363061879877847	0.0855192750488044
**cg01441738**	1	180352217	ACBD6	-0.0527949	-0.106179874110821	0.0855192750488044
**cg23303585**	6	32940054	BRD2;BRD2	0.00597823	0.489981470860512	0.0855192750488044
**cg24644049**	4	85504048	CDS1	0.0385264	0.739823120870868	0.0855192750488044
**cg13455444**	9	131833703	FAM73B	-0.0297547	-0.0477937014439323	0.0855192750488044
**cg01143309**	17	72889783	FADS6	0.0371254	0.164102688496488	0.0855192750488044
**cg22712329**	1	244624555	C1orf101;C1orf101	0.0262803	0.596197941524036	0.0855192750488044
**cg21224049**	4	668623	ATP5I	0.0142149	0.457729983608058	0.0855192750488044
**cg02958169**	17	7184873	SLC2A4	0.0281753	0.667153707724179	0.0855192750488044
**cg02344238**	11	65153777	FRMD8	0.00767457	0.365354681346234	0.0855192750488044
**cg06143886**	13	101142914	PCCA;PCCA;PCCA	-0.0722491	-0.13178894850367	0.0855192750488044
**cg04456437**	3	119217424	C3orf1;C3orf1	0.00645879	0.424299695987105	0.0855192750488044
**cg15437053**	19	34311123		0.0176261	0.655222807152696	0.0855192750488044
**cg05687930**	1	225840508	ENAH;ENAH;ENAH;ENAH	0.00830021	0.373525096041534	0.0855192750488044
**cg06942064**	1	208417143	PLXNA2;PLXNA2	0.0264788	0.632316185199399	0.0855192750488044
**cg02570171**	17	39822195		0.0142025	0.74967896446903	0.0855192750488044
**cg10734849**	2	217215658	MARCH4	-0.0450749	-0.075775282329955	0.0855192750488044
**cg01630422**	19	45510733	RELB	-0.0248097	-0.039360962981449	0.0855192750488044
**cg13569527**	20	33680323	TRPC4AP;TRPC4AP	0.022812	0.575208885674837	0.0855327197115916
**cg00018896**	13	25671476	PABPC3	-0.0451995	-0.0737506496267862	0.0855327197115916
**cg09357831**	7	100214622		-0.0511941	-0.135558845166512	0.0855368273312735
**cg14497781**	8	124169251		0.030301	0.75544997974366	0.0856375391074567
**cg09913544**	22	39269086	CBX6	0.0111	0.684117201152256	0.0856375391074567
**cg08811958**	20	43349788	WISP2	-0.0435381	-0.0685777507658229	0.0856375391074567
**cg02601685**	17	55055297	SCPEP1	0.0075303	0.416314652556925	0.0856375391074567
**cg00894481**	1	15912201	AGMAT	-0.0499029	-0.102800933234037	0.0856450705441419
**cg06752163**	3	170588210	RPL22L1	0.00509817	0.396752663916422	0.0856450705441419
**cg23948080**	14	77227645	VASH1	0.0210837	0.613552285599204	0.0856450705441419
**cg02276747**	7	43909057	URGCP-MRPS24;MRPS24	0.0326749	0.984848775971333	0.0856527675325678
**cg08866144**	6	31371784	MICA	0.0410855	0.271053014335895	0.0856760188358825
**cg12965753**	16	691705	FAM195A	0.0070235	0.362099831694961	0.0856760188358825
**cg13676204**	14	61447818	SLC38A6;TRMT5	0.00328659	0.327376981480651	0.0856760188358825
**cg20877627**	17	79368872		0.00423887	0.36158697309156	0.0856926240155534
**cg10435486**	8	77591419	LOC100192378	-0.0549171	-0.152394604324409	0.0857449281392891
**cg10821925**	12	29533733	ERGIC2	0.0346839	0.770148916957928	0.0857449281392891
**cg03311339**	17	64299562	PRKCA	0.039891	0.512294569542289	0.0857449281392891
**cg23389783**	7	138720575	ZC3HAV1L	0.00665	0.3528370309385	0.0857449281392891
**cg07734253**	16	30194717	CORO1A	0.034964	1.33683384473226	0.0857449281392891
**cg12267222**	1	203296843		0.0145529	0.61833907597285	0.0857449281392891
**cg01153132**	6	119400114	FAM184A;FAM184A	0.0091538	0.470368014161469	0.0857449281392891
**cg06100147**	7	143059260	FAM131B;FAM131B	0.0445315	0.567931600910926	0.0857449281392891
**cg26308874**	10	7478473		0.0088971	0.013265549855482	0.0857449281392891
**cg26000619**	19	2251067	AMH	0.0540911	0.290570626116059	0.0857449281392891
**cg02541778**	8	55382901		0.0578708	0.424923194362796	0.0857449281392891
**cg01757124**	17	76320916		0.00716586	0.622532537683103	0.0857449281392891
**cg23819836**	6	15663134	DTNBP1;DTNBP1;DTNBP1;DTNBP1;DTNBP1;DTNBP1	0.0440301	0.391777990928595	0.0857449281392891
**cg01547051**	7	150777654	FASTK;FASTK	0.0414595	1.26250011351382	0.0857449281392891
**cg10457397**	4	110624915	CASP6;CASP6	0.0208296	0.498002120209624	0.0857449281392891
**cg12065524**	17	29718231	RAB11FIP4	0.0268062	0.382839788665665	0.0857449281392891
**cg25180579**	6	3982635		-0.053241	-0.0909006083822817	0.0857449281392891
**cg21152662**	4	48231543	TEC	-0.0603193	-0.107115314936557	0.0857449281392891
**cg23706268**	13	51484060	RNASEH2B;RNASEH2B;RNASEH2B;RNASEH2B	0.0150147	0.98518438560639	0.0857449281392891
**cg16296880**	4	39367793	RFC1	0.0204342	0.726443693530587	0.0857449281392891
**cg22314900**	2	109150331		0.0109679	0.436402078383206	0.0857449281392891
**cg20346165**	20	39318984	MAFB	0.0252154	0.613108746566854	0.0857449281392891
**cg22265303**	5	77596033		-0.0287076	-0.0476112341917675	0.0857449281392891

**cg04922015**	16	86500429		0.00423456	0.00629741425534387	0.0857449281392891
**cg12695615**	20	61428209	C20orf20	-0.00493352	-0.349871475935404	0.0857449281392891
**cg03159456**	12	125399716	MIR5188;UBC	0.0170374	0.548787804785667	0.0857449281392891
**cg10139614**	16	88878480	APRT;APRT	0.0216632	1.128035866585	0.0857449281392891
**cg07022554**	16	1993481	SEPX1	0.0471292	0.684921598281437	0.0857449281392891
**cg04659689**	7	2903377		0.00454105	0.347633712345431	0.0857449281392891
**cg19929403**	3	50220205	SEMA3F	0.0118719	0.0179274860254007	0.0857449281392891
**cg25126090**	7	4722778	FOXK1	0.00605453	0.384567891366501	0.0857449281392891
**cg24524245**	8	8537351		-0.0728673	-0.154460608088326	0.0857449281392891
**cg20317272**	18	60383672	PHLPP1	0.0123261	0.514284827634439	0.0857449281392891
**cg18656667**	12	14924100	HIST4H4	0.00469558	0.334385655631611	0.0857449281392891
**cg10860647**	11	9385980		0.0190223	0.576682594719942	0.0857449281392891
**cg21379800**	3	130745449	NEK11;ASTE1;ASTE1;NEK11;NEK11	0.00468776	0.311301092789636	0.0857449281392891
**cg25053907**	4	492859	ZNF721;PIGG;PIGG;ZNF721	0.0147701	0.428017745001202	0.0857449281392891
**cg03149593**	3	136988095		-0.0485415	-0.0871362144474621	0.0857449281392891
**cg27416518**	3	190610654		-0.00625549	-0.00931211722897631	0.0857449281392891
**cg24830331**	1	28807088	PHACTR4;PHACTR4	-0.00587404	-0.00876874710982874	0.0857449281392891
**cg19605250**	20	1206806	RAD21L1;RAD21L1	0.0135084	0.483388242518669	0.0857449281392891
**cg12936509**	7	129984292	CPA5;CPA5;CPA5	-0.0599439	-0.110554899933644	0.0857449281392891
**cg21921473**	15	23087656	NIPA1;NIPA1	-0.0401195	-0.0670816043527821	0.0857449281392891
**cg21028326**	22	50683188	TUBGCP6;TUBGCP6	0.0368312	1.28653108380936	0.0857449281392891
**cg00580836**	20	62339176	ARFRP1;ARFRP1;ARFRP1;ARFRP1;ARFRP1;ZGPAT;ZGPAT;ZGPAT;ZGPAT;ARFRP1;A	0.0324763	0.635313665839809	0.0857449281392891
**cg08858239**	8	134569763	ST3GAL1;ST3GAL1	0.00473642	0.00698882186109983	0.0857449281392891
**cg04892409**	1	170501224	GORAB;GORAB;GORAB	0.0326848	0.526288111853717	0.0857449281392891
**cg10189607**	4	184719753		0.0124347	0.406828525738214	0.0857449281392891
**cg03609308**	1	3664084	KIAA0495	0.00457046	0.44529653646194	0.0857449281392891
**cg23415212**	17	37331429	CACNB1	0.027796	0.39559246065974	0.0857449281392891
**cg18427336**	2	69533779		0.00613279	0.576333994737979	0.0857449281392891
**cg03262242**	1	28559476	DNAJC8	0.032941	0.330677944674467	0.0857449281392891
**cg23046897**	19	55851104	SUV420H2	0.0214282	0.522364896444514	0.0857449281392891
**cg11099006**	17	79860645	NPB	0.0153111	0.380167051969718	0.0857449281392891
**cg03396354**	19	46519603	CCDC61	0.0114941	0.593623932974506	0.0857449281392891
**cg00148252**	4	89906159	FAM13A	-0.0432932	-0.0824364641011914	0.0857449281392891
**cg27087150**	7	150756527	SLC4A2;CDK5;CDK5	0.0130187	0.460035773917969	0.0857449281392891
**cg20361600**	2	172967608	DLX2	0.0104148	0.483105214652626	0.0857449281392891
**cg02250071**	6	150465049	PPP1R14C	0.012141	0.537217376285555	0.0857449281392891
**cg15877295**	7	138915876	UBN2	0.00886001	0.366985101495606	0.0857449281392891
**cg11756346**	20	47835881	DDX27;DDX27	0.00708829	0.483255958877586	0.0857449281392891
**cg23662032**	12	107712545	BTBD11;BTBD11	0.0166192	0.592937909176675	0.0857449281392891
**cg22637307**	2	68590998	PLEK	-0.0889567	-0.162079694305693	0.0857449281392891
**cg13912060**	14	68141881	VTI1B	0.0165504	0.549745333754205	0.0857449281392891
**cg13133000**	1	78463620		-0.0615943	-0.117862765095812	0.0857449281392891
**cg14237287**	19	40768217	AKT2;AKT2;AKT2	-0.0182507	-0.0277304627526744	0.0857449281392891
**cg06853376**	5	125937059	PHAX	0.0210585	0.518558974223643	0.0857449281392891
**cg02717770**	15	63136985		0.00823074	0.0122520626374781	0.0857449281392891
**cg14491479**	8	144623711	ZC3H3	0.0284362	0.613241364680025	0.0857449281392891
**cg26809482**	1	16940026	NBPF1	0.0177758	0.602780472300266	0.0857449281392891
**cg10673833**	7	45018849	MYO1G	0.00807491	0.481568406783541	0.0857449281392891
**cg16943083**	18	500817	COLEC12	0.028616	0.427711076791276	0.0857449281392891
**cg04680247**	2	26007561	ASXL2	-0.0200132	-0.0304837726923483	0.0857449281392891
**cg15627380**	17	80656703	RAB40B	0.0319022	1.11320215517378	0.0857449281392891
**cg07675285**	16	27121267		0.0468718	0.375429098715402	0.0857449281392891
**cg16113681**	22	19748925	TBX1;TBX1;TBX1	0.0195806	0.469319358258045	0.0857449281392891
**cg02695969**	19	932813	ARID3A	0.0154691	0.477031731065707	0.0857449281392891
**cg12125605**	3	181514754		-0.0475646	-0.079309934520363	0.0857449281392891
**cg02979871**	9	112402944	PALM2	0.0151053	0.52834945567995	0.0857449281392891
**cg06976096**	3	87138510		0.0420874	0.724677340010122	0.0857449281392891
**cg01082498**	11	68608225	CPT1A;CPT1A	0.0107945	0.425029561576467	0.0857449281392891
**cg07032309**	8	103668104	KLF10	0.0266888	0.882368127176271	0.0857449281392891
**cg08297053**	16	30905110	BCL7C;MIR762	0.0152681	0.599351030574106	0.0857449281392891
**cg26080096**	4	152340180	FAM160A1	-0.0388459	-0.0644702721237496	0.0857449281392891
**cg23620315**	3	13572380		-0.0551734	-0.0879115378090351	0.0857449281392891
**cg17569397**	2	200775850	C2orf69	0.0217682	0.754555147117887	0.0857449281392891
**cg15369512**	17	1012967	ABR;ABR;ABR	0.010118	0.370558845270498	0.0857449281392891
**cg20260663**	12	56211576	ORMDL2;SARNP;SARNP;SARNP	0.0224614	0.818366814882746	0.0857449281392891
**cg09248284**	2	27851818	CCDC121;CCDC121;GPN1;CCDC121;GPN1;GPN1;GPN1;CCDC121;GPN1	0.0294242	0.491961929813473	0.0857449281392891
**cg07388806**	6	7108895	RREB1;RREB1;RREB1;RREB1	0.0050525	0.43967623965243	0.0857449281392891
**cg16389027**	5	137056645	KLHL3;KLHL3;KLHL3	-0.04373	-0.0721366942587397	0.0857449281392891
**cg09077975**	15	87533146	AGBL1	-0.012582	-0.0190693852431337	0.0857449281392891
**cg01355526**	2	232918190	DIS3L2	-0.0300448	-0.048101192073584	0.0857449281392891
**cg16985048**	11	75716813	UVRAG	-0.0327912	-0.0533438342838287	0.0857449281392891
**cg18122310**	12	50236657	BCDIN3D	0.0110567	0.532341953277288	0.0857449281392891
**cg00472898**	16	28847676	ATXN2L;ATXN2L;ATXN2L;ATXN2L;ATXN2L;ATXN2L;ATXN2L	-0.0251222	-0.0398586921340959	0.0857449281392891
**cg11712217**	17	80455631		0.0262475	0.63314976785879	0.0857449281392891
**cg13883202**	3	47422330	PTPN23	0.0211468	0.631079499175718	0.0857449281392891
**cg26369308**	19	41172324	NUMBL	-0.067019	-0.129599243938311	0.0857524769714478
**cg13131061**	2	225268095	FAM124B;FAM124B	-0.0383135	-0.0656492023834379	0.0857524769714478
**cg10970409**	3	42543566	VIPR1	0.0487319	0.737984759189468	0.0857524769714478
**cg16983788**	3	179043854	ZNF639;ZNF639;ZNF639	-0.072998	-0.163096163248515	0.0857524769714478
**cg13067052**	16	2518286		0.00661919	0.513720880783488	0.0857524769714478
**cg10982433**	10	16563821	C1QL3;C1QL3	0.0250415	0.560286541475081	0.0857524769714478
**cg14561071**	8	30769215		0.0185291	0.376604093262937	0.0857524769714478

**cg00564759**	16	29818771	MAZ;MAZ	0.0608427	0.732013815544876	0.0858103807138488
**cg02807964**	11	10814144		0.0276401	0.562267925253505	0.0858103807138488
**cg07849112**	20	36577384		-0.0269032	-0.0449126948938924	0.0858103807138488
**cg23283076**	1	27114325	PIGV	0.00816182	0.350208790873891	0.0858103807138488
**cg11054120**	12	68828311	LOC100507195	-0.0575834	-0.0922776071186648	0.0858103807138488
**cg04410587**	7	156743073	NOM1	0.0240603	0.595535603857988	0.0858103807138488
**cg13412213**	10	119598364		-0.0581758	-0.13355996792799	0.0858103807138488
**cg21397993**	1	243418055	CEP170;SDCCAG8;CEP170;CEP170	0.0383526	0.683658484252521	0.0858103807138488
**cg23203809**	14	81686857	GTF2A1;GTF2A1	-0.0224253	-0.40423710964392	0.0858103807138488
**cg24568646**	21	30446238	CCT8	0.00527515	0.364852809332281	0.0858620832602374
**cg01458961**	4	102269425	PPP3CA;PPP3CA;PPP3CA	0.0569296	0.942332385461137	0.0858620832602374
**cg00474734**	12	53645975	MFSD5;MFSD5;MFSD5	0.0368862	0.405644425744312	0.0859336176360627
**cg10559432**	20	50179543	NFATC2	0.0135275	0.282486692000249	0.0859336902715438
**cg03232937**	18	10599276		-0.0411061	-0.0675413732971352	0.0859602563424653
**cg15778335**	22	43011005	POLDIP3;POLDIP3;RNU12	0.042372	0.370519687475654	0.0859602563424653
**cg26849331**	6	32116216	PRRT1	0.0307444	0.289228278107273	0.085983907179039
**cg07526883**	14	39572542	SEC23A	0.0146001	0.690914598449073	0.0861036694173011
**cg02933679**	1	13910224	PDPN;PDPN	0.0585758	0.396086000096395	0.0861203027284899
**cg05863534**	17	34948351	DHRS11;DHRS11	0.0281105	0.960605323292582	0.0861203027284899
**cg02565863**	14	56584899	PELI2	0.0397121	0.43416892757874	0.0861203027284899
**cg10209391**	1	26633129	UBXN11;UBXN11;UBXN11;UBXN11;UBXN11	0.00650842	0.488262377379844	0.0861203027284899
**cg12365193**	8	144692039	PYCRL	0.0216607	0.608866709109554	0.0861203027284899
**cg19367256**	1	18651998	IGSF21	-0.0220105	-0.0352060123547406	0.0861270842413176
**cg18214701**	17	48172593	PDK2;PDK2;PDK2;PDK2	0.0166268	0.678603460436062	0.0861270842413176
**cg00041084**	5	64398983		0.0071752	0.421459129755645	0.0861270842413176
**cg02398663**	18	45663510		0.0106867	0.604834478161489	0.0861270842413176
**cg24899519**	11	59522528	STX3;STX3	0.00752355	0.848058039568072	0.0861270842413176
**cg17632233**	11	62439667	C11orf48;C11orf83	0.026333	0.92721770676941 8	0.0861270842413176
**cg03548744**	12	110718499	ATP2A2;ATP2A2	0.014768	0.372609478614637	0.0861270842413176
**cg14781686**	10	70287434	SLC25A16	0.0680211	1.08225763434677	0.0861270842413176
**cg02009895**	12	54380101	HOXC10	0.0168279	0.44629926817631	0.0861583160345699
**cg04247218**	16	27561328	GTF3C1;KIAA0556	0.00487895	0.33080417027282	0.0861583160345699
**cg16214654**	2	61697828	USP34;USP34	0.0420314	1.41103249020049	0.0861583160345699
**cg24182603**	12	111843578	SH2B3	0.00676442	0.322431796073833	0.0861583160345699
**cg14661383**	1	228463720	OBSCN;OBSCN	0.00812346	0.391427419588199	0.0861583160345699
**cg26649834**	1	38156051	C1orf109;C1orf109	0.00397872	0.32857066834859	0.0861583160345699
**cg20559561**	12	120972173	RNF10;RNF10	0.00928704	0.618901196361024	0.0861583160345699
**cg15131414**	19	37157847	ZNF461	0.0259965	0.763039115170924	0.0861871565094117
**cg14179433**	15	65809620	DPP8;DPP8;DPP8;DPP8	0.0115606	0.529859880882523	0.0861871565094117
**cg16528891**	1	36169528		-0.0846549	-0.186226090566163	0.0861871565094117
**cg02944953**	19	38544658	SIPA1L3	-0.0411612	-0.0658379159326256	0.0861871565094117
**cg16579225**	1	2322553	RER1;MORN1	0.00781719	0.53148855615036	0.0861871565094117
**cg01915516**	1	3568243	TP73	0.0218979	0.839656753729767	0.0861871565094117
**cg18770186**	8	141521259	CHRAC1;CHRAC1	0.0246205	0.842539240125454	0.0861871565094117
**cg20089744**	3	128372087		-0.022761	-0.0347019702710593	0.0861871565094117
**cg24376776**	10	101297245		0.0260028	0.713034054026896	0.0861871565094117
**cg01706029**	16	27325672	IL4R;IL4R	0.009453	0.400981983405204	0.0861871565094117
**cg06802812**	5	40679165	PTGER4	0.00767352	0.346661430161714	0.0863044940363843
**cg10536369**	2	170335894	BBS5	0.0260319	1.06243981267092	0.0863071052626647
**cg02037503**	14	23540729	ACIN1;ACIN1;ACIN1;ACIN1;ACIN1;ACIN1	0.0692757	0.547588610528149	0.0863071052626647
**cg16747999**	8	95653174	ESRP1;ESRP1;ESRP1;ESRP1;ESRP1	0.0165616	0.693367172017122	0.0863071052626647
**cg16166399**	19	59030753	ZBTB45;ZBTB45	0.00675068	0.420537796260622	0.0863188198756657
**cg23704874**	12	11701293		-0.0397281	-0.0638039593795401	0.0864304769582493
**cg19097648**	5	115177461	ATG12;AP3S1;ATG12	0.0483512	1.27718107615971	0.0864471477515176
**cg21795699**	17	15903687	ZSWIM7;TTC19;ZSWIM7	0.0181202	0.89582705017588	0.0864471477515176
**cg06932451**	19	36869880		0.017996	0.597137366778245	0.0864555261626138
**cg08943344**	17	8374863		0.00989363	0.0147596065277746	0.0864882377859482
**cg01104200**	12	562065		-0.0334807	-0.0539553673131296	0.0864882377859482
**cg05954989**	10	135191962	PAOX;PAOX;PAOX	0.021335	0.965450758502688	0.0864882377859482
**cg07853246**	4	143768134	INPP4B;INPP4B	0.0113501	0.6983981938424	0.0864882377859482
**cg19935040**	17	42432165	FAM171A2	0.0423084	0.256789387467278	0.0865551772613161
**cg25126812**	14	69445989	ACTN1;ACTN1;ACTN1;ACTN1;ACTN1;ACTN1	0.00953321	0.502723651751618	0.0865668030596986
**cg18542078**	19	3184564	NCLN	-0.0393923	-0.0708970351948469	0.0865668030596986
**cg06184899**	14	77842967	SAMD15;TMED8	0.0181474	0.684085481434852	0.0865668030596986
**cg23033866**	17	79670804	MRPL12	0.0179798	0.53086517223639	0.0865984641183482
**cg05686118**	16	57610170	GPR114	-0.0411124	-0.0703520331997304	0.0865984641183482
**cg22165360**	15	64388139	SNX1;SNX1;SNX1	0.00534042	0.333868972313664	0.0865984641183482
**cg00374173**	19	47996733	NAPA;NAPA;NAPA;NAPA-AS1;NAPA;NAPA;NAPA	0.0123243	0.0185665729954898	0.0865984641183482
**cg23741330**	6	116832940	FAM26E;BET3L	-0.0194929	-0.0302241353620876	0.0865984641183482
**cg08742975**	1	204632973	LRRN2;LRRN2	0.0469095	0.0730005199689424	0.0865984641183482
**cg08060454**	3	137487932		0.0271193	0.595624515157898	0.0865984641183482
**cg07377675**	1	62901875	USP1;USP1;USP1	0.00881308	0.533814910135266	0.0865984641183482
**cg22684426**	12	49351253	ARF3	0.00455241	0.307945292585875	0.0865984641183482
**cg12052348**	11	118272271	ATP5L;ATP5L;LOC100131626;LOC100131626;ATP5L	0.0049072	0.306432544122462	0.0865984641183482
**cg01163848**	22	24060637	LOC91316	-0.0382882	-0.0659619541377722	0.0865984641183482
**cg24389730**	17	46114615	MIR152;COPZ2	0.0399214	0.34516935335577	0.0865984641183482
**cg27544871**	12	11654175		-0.0271028	-0.0467210154052516	0.0865984641183482
**cg23358871**	17	78121069	EIF4A3	0.0114824	0.632174352748182	0.0865984641183482
**cg07810106**	5	131630122	SLC22A4	0.0173097	0.648616406181379	0.0865984641183482
**cg22149995**	8	125461772	TRMT12	-0.112667	-0.216500210555074	0.0865984641183482
**cg17906245**	1	213021979	C1orf227	-0.0509095	-0.0820608582030326	0.0865984641183482
**cg20495247**	12	109536079	UNG;UNG;UNG	0.0102328	0.471898661475282	0.0865984641183482

**cg25281743**	17	42403239	SLC25A39;SLC25A39	0.0178578	1.01647594523352	0.0865984641183482
**cg09294055**	16	3314419		0.0176303	1.13896825114143	0.0865984641183482
**cg25868519**	14	75078907	LTBP2;LTBP2	0.0109424	0.570315114957726	0.0865984641183482
**cg16968115**	1	27560829	WDTC1	0.012254	0.951170532602798	0.0865984641183482
**cg08200077**	21	46221949	LINC01424;UBE2G2;UBE2G2;UBE2G2	0.00998569	0.57631710706078	0.0865984641183482
**cg10394354**	17	72857154	GRIN2C;GRIN2C;GRIN2C	0.0256669	0.535845415615799	0.0865984641183482
**cg13187009**	20	55965497	RBM38;RBM38	0.0102351	0.384776944626101	0.0865984641183482
**cg00983904**	12	6665288	IFFO1;IFFO1	0.0313723	0.504242266712841	0.0865984641183482
**cg25228502**	15	54039894	WDR72;WDR72	-0.023089	-0.0350679214075836	0.0865984641183482
**cg07385501**	16	30913935	CTF1;CTF1	0.0280668	0.522056072249598	0.0865984641183482
**cg16524216**	17	59040381	BCAS3;BCAS3	-0.0366835	-0.0658957994561362	0.0865984641183482
**cg01732449**	2	19563255		0.0204566	0.64928112509497	0.0865984641183482
**cg22704520**	2	200820451	C2orf60;C2orf60;C2orf47;C2orf60	0.056421	1.37797903309821	0.0865984641183482
**cg05225096**	6	34549802		-0.0474488	-0.0864040036220409	0.0865984641183482
**cg10386445**	2	26205648	KIF3C	0.0310945	0.882859397252783	0.0865984641183482
**cg03539382**	10	5884105		-0.0510423	-0.112406046788697	0.0865984641183482
**cg25418176**	14	91613557	C14orf159;C14orf159;C14orf159;C14orf159;C14orf159;C14orf159;C14orf159;C14orf159;C	0.0042571	0.00628387188558973	0.0865984641183482
**cg09999109**	8	54164310	OPRK1	0.0361217	0.703975040477202	0.0865984641183482
**cg21352959**	17	62340143	TEX2	0.0189883	0.807177676143576	0.0865984641183482
**cg26259546**	3	57113458	ARHGEF3	0.0108496	0.507575508046479	0.0865984641183482
**cg15482931**	15	27019448	GABRB3;GABRB3	-0.086952	-0.293742413120997	0.0865984641183482
**cg03448964**	2	32440900	SLC30A6;SLC30A6;SLC30A6;SLC30A6	-0.0635459	-0.112648540872099	0.0865984641183482
**cg07906495**	14	31028426	G2E3;G2E3	0.010425	0.363665460835674	0.0865984641183482
**cg15278043**	7	130353539	TSGA13;COPG2;COPG2	0.0216788	0.59239309815557	0.0865984641183482
**cg06821992**	5	56790874		-0.0537928	-0.266396790286552	0.0865984641183482
**cg25517151**	20	30467953	TTLL9	0.0252	0.618648837865885	0.0865984641183482
**cg21595549**	2	217045817	XRCC5	-0.0580761	-0.0942736682130481	0.0865984641183482
**cg15616888**	7	140397014	LOC100134713;NDUFB2	0.0139026	0.43311485744562	0.0865984641183482
**cg17509893**	11	14335070	RRAS2;RRAS2	-0.0204875	-0.0324564795219129	0.0865984641183482
**cg20942933**	12	29227192		-0.0446155	-0.0712160673313828	0.0865984641183482
**cg04796498**	8	145509700	1,00 BOP	0.00938403	0.014190276576452	0.0865984641183482
**cg08035378**	2	165951781	SCN3A;SCN3A;SCN3A	-0.0274519	-0.0421427739581216	0.0865984641183482
**cg27074174**	6	101847318	GRIK2;GRIK2;GRIK2	0.031598	0.338186465028195	0.0865984641183482
**cg12898144**	20	19861411		-0.0222982	-0.0345977904840878	0.0865984641183482
**cg11894282**	3	33773483		-0.0537341	-0.101371564096693	0.0865984641183482
**cg23925619**	9	140473536	DPH7	0.049972	0.95914802304593	0.0865984641183482
**cg09788111**	10	124768255	IKZF5;IKZF5;ACADSB	0.0178148	0.459252994720715	0.0865984641183482
**cg18985987**	12	70637240	CNOT2;CNOT2;CNOT2;LINC01481;LINC01481;LINC01481;CNOT2;CNOT2;CNOT2	0.111411	1.18482716145018	0.0865995146329599
**cg18423154**	13	100032253	UBAC2;UBAC2;UBAC2	0.00444753	0.0065852950510858	0.0865995146329599
**cg05645634**	10	118562797		-0.0118973	-0.0177823169944541	0.0865995146329599
**cg15796682**	11	506809	RNH1;RNH1;RNH1;RNH1;RNH1;RNH1;RNH1;RNH1;RNH1;RNH1;RNH1;RNH1	0.0174194	0.676552951168673	0.0865995146329599
**cg22375623**	15	78444981	IDH3A	-0.0399109	-0.0651557529396185	0.0865995146329599
**cg19189726**	19	34288308	KCTD15;KCTD15;KCTD15	0.0178189	0.432956475075424	0.0865995146329599
**cg24756756**	7	16793323	TSPAN13	0.0177544	0.664146749713995	0.0865995146329599
**cg22637872**	16	67875820	THAP11;CENPT	0.0151874	0.648461191294293	0.0865995146329599
**cg14183907**	9	136114344		0.0464954	0.628922816519897	0.0865995146329599
**cg18779601**	5	175792176	10,00 ARL	0.00949731	0.394287230782297	0.0865995146329599
**cg09556412**	4	1271909		-0.0511227	-0.0818951270058746	0.0865995146329599
**cg15900544**	1	153606818	C1orf77;S100A13;C1orf77	0.00719353	0.383753881573319	0.0865995146329599
**cg07131201**	11	61091489	DDB1	0.00486374	0.00715806553230527	0.0865995146329599
**cg12741435**	10	50822369	CHAT;CHAT;CHAT;CHAT;CHAT;CHAT;CHAT;CHAT;CHAT;CHAT	0.0349528	0.575228587867043	0.0865995146329599
**cg00962672**	18	12493772	SPIRE1;SPIRE1;SPIRE1	-0.0397449	-0.064860274548191	0.0865995146329599
**cg01888767**	5	131832552		0.0113839	0.621463611933485	0.0865995146329599
**cg12556586**	15	59733552	FAM81A	-0.0221726	-0.0345920396648472	0.0865995146329599
**cg00456162**	13	96706773	UGGT2	-0.0405432	-0.0651309126975199	0.0865995146329599
**cg05931684**	6	31865274	EHMT2;EHMT2	0.0573344	0.618820335571025	0.0865995146329599
**cg13481132**	9	138606811	KCNT1	0.0259886	0.815667920424856	0.0865995146329599
**cg05534807**	11	75379613	MAP6;MAP6	0.0124278	0.395181575616371	0.0865995146329599
**cg04308838**	18	21651322	TTC39C;TTC39C	-0.0561893	-0.0987604937485267	0.0866099367482584
**cg16701467**	17	7475727	EIF4A1	0.0106318	0.412630441235052	0.086620059728896
**cg02464768**	9	4490279	SLC1A1	0.010344	0.46371323100588	0.086620059728896
**cg03452047**	1	53067911	GPX7	0.0115978	0.391221052770308	0.086620059728896
**cg02839220**	16	4400790	Magmas	0.0220128	0.728836597526404	0.0867901359322289
**cg22783308**	14	75894315	JDP2;JDP2;JDP2	0.0169708	0.474181051384236	0.0868063804710912
**cg14566061**	12	106583254		-0.042841	-0.071527678192053	0.0868273721916035
**cg25236904**	12	132568643	EP400NL	0.0041435	0.329402899025866	0.0868361517336824
**cg02249648**	12	1703181	FBXL14	0.0201729	1.00751869401682	0.0868920910245392
**cg24725337**	6	89717866		-0.0273414	-0.0438343033639572	0.0868920910245392
**cg05916906**	19	8478510	MARCH2;MARCH2;MARCH2	0.0122015	0.314877227129226	0.0869570860492498
**cg05069934**	17	57287384	SMG8;SMG8	0.0106687	0.635455077761834	0.0869669435746998
**cg18751588**	12	51566859	TFCP2	0.0186263	0.861080130066762	0.0871313720871518
**cg24839693**	17	43212892	ACBD4;ACBD4;ACBD4;ACBD4;ACBD4	0.00818156	0.483194492688204	0.0871313720871518
**cg03088106**	14	21994412	SALL2;SALL2;SALL2;SALL2;SALL2;SALL2	0.041849	0.225782263343658	0.0871313720871518
**cg26616640**	12	58013458	SLC26A10	0.0203664	0.553402321675745	0.0871313720871518
**cg04582164**	3	152881085	RAP2B;RAP2B	0.00372664	0.448676690851428	0.0871313720871518
**cg04371319**	15	75249125	RPP25;RPP25	0.028584	0.614528437165825	0.0871313720871518
**cg18656581**	21	34602557	IFNAR2;IFNAR2;IFNAR2	0.012864	0.436260415383911	0.0871313720871518
**cg14462935**	21	40477951		-0.0875718	-0.174555269390858	0.0871313720871518
**cg18912965**	14	91526996	RPS6KA5;RPS6KA5	0.0581137	0.699020509736065	0.0871313720871518
**cg16575567**	1	201019592	CACNA1S	-0.0555015	-0.123986957547578	0.0871313720871518
**cg12189097**	1	207178154		-0.0170956	-0.0257514197106088	0.0871313720871518
**cg16700296**	6	159125489	SYTL3	0.0176143	0.606104279045694	0.0871313720871518

**cg21042539**	5	176514085	FGFR4;FGFR4	0.0180938	0.605475784910254	0.0871313720871518
**cg12069276**	11	10476242	AMPD3;AMPD3;AMPD3	-0.0202326	-0.0314032486080442	0.0871313720871518
**cg02074591**	16	3220802		0.00554013	0.265553765523099	0.0871313720871518
**cg11314629**	3	48583854	PFKFB4	0.0118163	0.0183121063596729	0.0871313720871518
**cg02150526**	17	38136938	PSMD3	0.00689567	0.408193786451229	0.0871313720871518
**cg23710795**	20	57875655	EDN3;EDN3;EDN3;EDN3;EDN3;EDN3;EDN3;EDN3;EDN3	0.0144888	0.330750157482516	0.0871545927395992
**cg01730032**	4	135248255		-0.0338979	-0.055381418220262	0.0871545927395992
**cg13190924**	15	44084524	SERF2;SERF2	0.0138687	1.17448688683222	0.0871941972236036
**cg21767703**	1	236444768	ERO1LB	0.0222572	0.460346622281909	0.0872145217218695
**cg25542041**	9	124982087	LHX6;LHX6	0.0161711	0.561240447266441	0.0872460946563789
**cg16529563**	19	49560284	CGB7	0.00990721	0.368169961110081	0.0872766515169698
**cg17130815**	7	2558891	LFNG;LFNG;LFNG;LFNG	0.026786	1.14971904742815	0.0873116285634835
**cg06980251**	18	60263900		0.0314279	0.499437827634546	0.0873116285634835
**cg05256313**	3	124926431	SLC12A8;SLC12A8	-0.0127349	-0.0189094438846705	0.0873127323014528
**cg19434470**	17	6917616	RNASEK;RNASEK;RNASEK-C17orf49;C17orf49;C17orf49;C17orf49;RNASEK	0.0254968	0.41757375303449	0.0873127323014528
**cg04680304**	11	4116184	RRM1;RRM1	0.0124246	0.511439066436799	0.0873127323014528
**cg02072992**	16	83960431		0.0314955	0.621345576756324	0.0873410858589919
**cg05885483**	12	49412537	PRKAG1;PRKAG1;PRKAG1;PRKAG1;PRKAG1	0.0208081	0.580239766154345	0.0873707738560687
**cg24126878**	12	7342151	PEX5;PEX5;PEX5;PEX5;PEX5	-0.0127412	-0.852032764041063	0.0873726159235019
**cg12306414**	4	13549355	LOC285548	0.0527431	0.331773642684301	0.0873726159235019
**cg01003803**	8	16859632	FGF20;FGF20	0.0232366	0.457602097105887	0.0873726159235019
**cg15765896**	12	53472985	SPRYD3	0.0161035	0.698552831762123	0.0873726159235019
**cg24814784**	6	31830947	NEU1	0.00912967	0.510805600721979	0.0873726159235019
**cg22376230**	16	67880540	NUTF2;CENPT	0.0126683	0.444358558540035	0.0873726159235019
**cg05144147**	10	6018955	IL15RA;IL15RA	0.0267205	0.994607581793024	0.0873726159235019
**cg02832997**	21	47878903	DIP2A;DIP2A;DIP2A;DIP2A;DIP2A;DIP2A;DIP2A;DIP2A;DIP2A;DIP2A;DIP2A;DIP2A	0.0386174	0.254393984435112	0.0873837676655894
**cg09691861**	4	77870047	SEPT11	0.0354642	1.21147265596216	0.0874130849443185
**cg26681603**	10	75700474		0.0180876	0.534970399481228	0.0874130849443185
**cg12892033**	14	104181379	XRCC3;XRCC3;XRCC3;ZFYVE21;ZFYVE21	0.0409411	0.980641009479062	0.0874876030099216
**cg26622537**	11	101458233		-0.0416305	-0.0696096111480089	0.0874898720472256
**cg08890976**	12	100538115	UHRF1BP1L;UHRF1BP1L	-0.0546722	-0.127954035330262	0.0875262364298879
**cg05402395**	21	47427081		-0.0496481	-0.0813216580169818	0.0875996012402595
**cg07784294**	11	11873490	USP47	-0.0703491	-0.121351262497655	0.0876138973958095
**cg26679668**	3	49218951	C3orf84	-0.0634247	-0.120653222948983	0.0876138973958095
**cg18286862**	4	123300469	ADAD1;ADAD1;ADAD1	0.0121103	0.0187214529864137	0.0876365453329989
**cg06090383**	4	174292579	SAP30	0.032017	0.5211622710008	0.0876365453329989
**cg20204009**	12	94071654	CRADD	0.0294389	0.544662124769178	0.0876365453329989
**cg21076271**	14	32546633	ARHGAP5;ARHGAP5;ARHGAP5;C14orf128;ARHGAP5	0.0232697	0.772981009373977	0.0876365453329989
**cg07274716**	5	134363973	PITX1	0.0282718	0.535026309248913	0.0876365453329989
**cg17089785**	15	99562412		-0.0331015	-0.0512755011086281	0.0876365453329989
**cg04329455**	9	137215364		-0.00811534	-0.0121466149025329	0.0876755105579002
**cg26896160**	1	6454004	ACOT7	0.0216602	0.883308727030421	0.0876911800183251
**cg11762839**	11	17565885	USH1C;USH1C;USH1C;USH1C	0.0130361	0.47904050103536	0.0876911800183251
**cg03568717**	12	109458732	SVOP;SVOP	0.0131988	0.499115577478459	0.0876911800183251
**cg15433056**	20	44993218	SLC35C2;SLC35C2;SLC35C2	0.00827964	0.407281696555966	0.0876911800183251
**cg02875253**	16	3314612		0.0106593	0.423432551981614	0.0876911800183251
**cg02996314**	1	32479425	KHDRBS1	0.0190915	0.704803920588197	0.0876911800183251
**cg13140338**	1	91317255		0.0308751	0.576745392591229	0.0876911800183251
**cg18829560**	17	7298064	PLSCR3	0.00514093	0.254461514959414	0.0876911800183251
**cg17874528**	15	86338361	KLHL25	0.0171094	0.749060316259336	0.0876911800183251
**cg22840361**	15	96887919		0.0219139	0.258591193097248	0.0876911800183251
**cg24168641**	15	74219645	LOXL1	0.0315263	0.429200150408319	0.0876911800183251
**cg11116176**	9	135805713	TSC1;TSC1;TSC1	-0.0911983	-0.163915302549293	0.0876911800183251
**cg26522937**	7	101440743		-0.0339733	-0.0542135813035404	0.0876911800183251
**cg02743222**	5	170736335	TLX3;TLX3	0.0176042	0.455762566241758	0.0876911800183251
**cg03630756**	19	41839506	TGFB1	-0.0401299	-0.0667490396223754	0.0876911800183251
**cg12100721**	17	79316639		0.0292759	0.461557607469209	0.0876911800183251
**cg05827631**	1	121261404	LOC647121	0.00831495	0.358060922308107	0.0876911800183251
**cg10451253**	17	32907705	TMEM132E;C17orf102	0.0101751	0.483137976168779	0.0876911800183251
**cg26442458**	11	65728985	SART1	0.017306	0.984961025033792	0.0876911800183251
**cg17318645**	14	77787612	GSTZ1;POMT2;GSTZ1;GSTZ1	0.0163338	0.632184477345835	0.0876911800183251
**cg23237765**	7	921845	C7orf20	-0.0848101	-0.142426389604335	0.0876968158562571
**cg21604741**	2	27713092	IFT172	-0.0507984	-0.186090143895555	0.0876968158562571
**cg11369958**	8	141451722	TRAPPC9;TRAPPC9	-0.08498	-0.14290505044825	0.0876968158562571
**cg11352339**	6	43482436	YIPF3	-0.0603439	-0.0979958671704588	0.0876968158562571
**cg26928080**	14	95236825	GSC	0.0211178	0.267278072697447	0.0876968158562571
**cg05721281**	3	197477555	FYTTD1;FYTTD1;FYTTD1;KIAA0226	0.0164666	0.519420977300906	0.0876968158562571
**cg15792158**	12	113574084	RASAL1	0.0560287	0.7186664337282	0.0876968158562571
**cg19752627**	7	98467380	TMEM130;TMEM130;TMEM130	0.00992827	0.411417070143745	0.0876968158562571
**cg14959506**	16	1525533	CLCN7;CLCN7	0.0162802	0.478967878467395	0.0876968158562571
**cg07759394**	11	134201954	GLB1L2;GLB1L2	0.00864049	0.490695987343039	0.0876968158562571
**cg02482035**	1	41220017	MIR30E;NFYC;NFYC;NFYC;NFYC;NFYC;NFYC;NFYC	-0.0214787	-0.0343398011146633	0.0876968158562571
**cg18273818**	5	5422589	KIAA0947	0.0103743	0.615173594273549	0.0876968158562571
**cg18410117**	6	90645081	BACH2;BACH2	-0.00848131	-0.0125555957870713	0.0877046793049971
**cg02893015**	8	82755558	SNX16;SNX16;SNX16	-0.0463173	-0.104791694601495	0.0877216144658561
**cg26968714**	15	20152530		-0.0292952	-0.0521602715946582	0.0877294765252078
**cg05013825**	14	76870341	ESRRB	-0.0531688	-0.103811233979351	0.0877960490122498
**cg09012337**	11	73019505	ARHGEF17	0.0181161	0.424879897211853	0.0877960490122498
**cg06405860**	1	60539458	C1orf87	0.031692	0.292097777648803	0.0877960490122498
**cg18956250**	1	26798730	HMGN2	0.0108972	0.641472040933614	0.0877960490122498
**cg10122932**	7	99698990	MCM7;MCM7;MCM7;AP4M1	0.0277232	0.848007131211059	0.0877960490122498
**cg03800721**	12	120525338	CCDC64	0.00793653	0.494132685616719	0.0877960490122498

**cg08999477**	11	43702335	HSD17B12;HSD17B12	0.0081712	0.475401118060871	0.0877960490122498
**cg12796916**	11	67471698		-0.0373546	-0.0581220138881617	0.0877960490122498
**cg00436174**	2	128051630	ERCC3	0.0317912	0.375303596950433	0.0877960490122498
**cg01416388**	22	39784598		0.0375956	0.620268281252915	0.0877960490122498
**cg00721468**	6	8102731	EEF1E1;EEF1E1	0.0161106	0.494322454336839	0.0877960490122498
**cg00084591**	9	102793395	ERP44	-0.111109	-0.253072695372862	0.0877960490122498
**cg21741689**	3	128327189		0.0223622	0.775458686689035	0.0877960490122498
**cg23604697**	11	66046125	CNIH2;CNIH2;CNIH2	0.00514147	0.343174902248033	0.0877960490122498
**cg10192736**	19	1028627	CNN2;CNN2	0.0230318	0.694517048365421	0.0877960490122498
**cg27010645**	5	80597288	RNU5E;ZCCHC9;ZCCHC9;RNU5D;ZCCHC9	0.0100181	0.261879596344587	0.0877960490122498
**cg12578536**	5	43003251		-0.110511	-0.198800168437052	0.0877960490122498
**cg06067493**	8	54755683	ATP6V1H;ATP6V1H;ATP6V1H;ATP6V1H;ATP6V1H	0.00825031	0.315869319625885	0.0877960490122498
**cg19318450**	21	34852305	TMEM50B	0.0360116	0.397047694816603	0.0877960490122498
**cg15949708**	11	91506467		-0.044595	-0.0819124821420106	0.0877960490122498
**cg05925327**	15	68127851		0.0257249	0.582310215438493	0.0877960490122498
**cg21116579**	14	92414253	FBLN5	0.0180492	0.636406553672467	0.0877960490122498
**cg15059511**	15	41099719	ZFYVE19;ZFYVE19;DNAJC17	0.00967921	0.468906642981101	0.0877960490122498
**cg21250048**	10	89667551	PTEN;PTEN;PTEN	-0.0966346	-0.173804081960211	0.0877960490122498
**cg14675606**	1	111583567		-0.0473457	-0.0916233942584799	0.0877960490122498
**cg12745174**	11	11179018		0.0220509	0.0365888664648981	0.0877960490122498
**cg08888610**	14	104181384	XRCC3;XRCC3;XRCC3;ZFYVE21;ZFYVE21	0.0259982	0.465113430767571	0.0877960490122498
**cg22712861**	19	41732544	AXL;AXL;AXL	-0.0793007	-0.746144669444364	0.0877960490122498
**cg25240814**	14	77608147	ZDHHC22	0.0113781	0.416405278962191	0.0877960490122498
**cg17044356**	17	7758228	TMEM88	0.0608774	0.10657997464856	0.0877960490122498
**cg02412628**	20	1473638	SIRPB2;SIRPB2	0.0147827	0.0227169331961186	0.0877960490122498
**cg11817993**	14	92572978	ATXN3;ATXN3;ATXN3;ATXN3;ATXN3;ATXN3;ATXN3;ATXN3;ATXN3;ATXN3;ATX	0.0324429	0.749114890938853	0.0877960490122498
**cg00154455**	8	146023573	ZNF517	-0.0463205	-0.0735885458502588	0.0877960490122498
**cg18330675**	4	180980172		0.0365765	0.821209796245921	0.0877960490122498
**cg08828594**	15	99558786		0.00695075	0.475727467019784	0.0877960490122498
**cg08885800**	1	201084119		0.0245901	0.364303564499573	0.0877960490122498
**cg12503190**	17	74733672	MIR636;SFRS2;MFSD11	0.00824797	0.526880013678761	0.0877960490122498
**cg14660449**	1	233030601		-0.0135845	-0.0206114241916289	0.0877960490122498
**cg16277306**	12	105351751		0.0291864	0.313386542123773	0.0877960490122498
**cg00064218**	9	130922498	C9orf16	0.0333638	0.65616123591686	0.0877960490122498
**cg20019673**	2	21022567	LDAH;LDAH;LDAH;LDAH;LDAH;LDAH;LDAH;LDAH	0.0100934	0.340382867496018	0.0877960490122498
**cg09491787**	5	139781927	ANKHD1;ANKHD1-EIF4EBP3;ANKHD1;ANKHD1	0.00499844	0.397345837505921	0.0877960490122498
**cg11445634**	15	61821004		-0.0471841	-0.239445502095433	0.0877960490122498
**cg05490686**	4	141419553	MGAT4D	0.0154362	0.503499016371889	0.0877960490122498
**cg14861020**	8	1772352	ARHGEF10	0.0340543	0.656400630652199	0.0877960490122498
**cg16788234**	11	108093338	NPAT;ATM;NPAT	0.0134384	0.390731478029172	0.0877960490122498
**cg24087678**	4	68830059	TMPRSS11A;TMPRSS11A	-0.091675	-0.185689430722753	0.0877960490122498
**cg26300500**	22	50885288	SBF1	-0.0254651	-0.0417697157988554	0.0877960490122498
**cg22280792**	17	77070793	ENGASE	0.0261244	0.726783391254262	0.0877960490122498
**cg07613693**	19	59031343	ZBTB45	0.00850971	0.750341976977267	0.0877960490122498
**cg25270632**	7	43751042	C7orf44	-0.094371	-0.178189453101049	0.0877960490122498
**cg24447660**	3	127872092	EEFSEC	0.00588754	0.43290426965663	0.0877960490122498
**cg17336584**	6	12750256	PHACTR1	0.0162832	0.625332619043993	0.0877960490122498
**cg00202496**	16	11037903	CLEC16A	-0.0658109	-0.115396812948946	0.0877960490122498
**cg13577676**	2	85936001		-0.0411147	-0.0714229348112305	0.0877960490122498
**cg18614859**	8	75896557	CRISPLD1	0.0170766	0.443006363109607	0.0877960490122498
**cg05231366**	17	1303810	YWHAE;YWHAE	0.0773521	1.62904973204849	0.0877960490122498
**cg05106984**	2	27435511	SLC5A6;C2orf28;SLC5A6;C2orf28;C2orf28	0.014341	0.671174815760098	0.0877960490122498
**cg03165203**	5	42986238	FLJ32255	-0.0793207	-0.162990739127498	0.0877960490122498
**cg18781187**	9	128510438	PBX3;PBX3;PBX3;PBX3	0.017165	0.63825344054903	0.0877960490122498
**cg12066233**	5	72850994	ANKRA2	-0.00878009	-0.012984959329077	0.0877960490122498
**cg24409253**	21	34914741	GART;GART;GART;GART;SON;SON;SON;SON;SON	0.00939153	0.494144113927248	0.0877960490122498
**cg24370247**	8	21646946	GFRA2;GFRA2;GFRA2	0.0435238	0.608068021559804	0.0878213128902921
**cg04255268**	2	220387374	ACCN4;ACCN4	0.035929	0.438514047860555	0.0878213128902921
**cg12174804**	8	18244861		0.0513436	0.472976214647992	0.0878213128902921
**cg00736523**	9	32783202	TMEM215	0.0233796	0.325318423068373	0.0878213128902921
**cg24569270**	16	88730409	MGC23284;MVD;MGC23284	0.0110026	0.520227212709172	0.0878213128902921
**cg04338001**	8	144363930	LINC01604;LINC01604	0.00853536	0.607732915359658	0.0878452514408801
**cg15541398**	10	112160883		-0.0379383	-0.0647352744706582	0.0878452514408801
**cg03533123**	14	55368924	GCH1;GCH1;GCH1;GCH1	0.0188493	0.652129254881778	0.0878452514408801
**cg22256677**	4	85416951	NKX6-1	0.0107037	0.390066538126136	0.0878452514408801
**cg23807112**	7	99533018		-0.108202	-0.266935595590453	0.0878452514408801
**cg26506795**	20	57467130	GNAS;GNAS;GNAS;GNAS;GNAS;GNAS;GNAS;GNAS;GNAS;GNAS;GNAS;GNAS;GNAS	0.0405428	0.588995470925762	0.0878452514408801
**cg00366814**	2	75061219	HK2;HK2	0.0231729	0.413512441347787	0.0878452514408801
**cg03828939**	16	2525452	TBC1D24	0.013429	0.557227988839997	0.0879201326830979
**cg10455971**	5	101119566		0.0747249	0.177600449052322	0.0881598815926446
**cg24667608**	4	128703125	HSPA4L	0.0593009	1.17711082379	0.0882075247290537
**cg25691044**	8	362718	FBXO25;FBXO25;FBXO25	-0.0521734	-0.0943426854691225	0.088246338020053
**cg13823521**	1	228353165	IBA57-AS1;IBA57;IBA57-AS1	0.00720897	0.568318691921654	0.0882617802994151
**cg09286444**	8	27880317	C8orf80	-0.0155872	-0.0238122959477464	0.0882617802994151
**cg21088485**	2	239335541	ASB1	0.0387701	0.363564756946825	0.0883210808063504
**cg18334142**	5	158636736		0.0121728	0.566324862102642	0.0883210808063504
**cg21435190**	3	128208037	GATA2;GATA2;GATA2	0.0137341	0.726838744985892	0.0883210808063504
**cg06522681**	19	15121596	CCDC105;CCDC105	0.0395815	0.504983139105896	0.0883292915585139
**cg05407743**	17	38375453	WIPF2	0.00632668	0.524597960204198	0.0884228563666723
**cg22091609**	22	20780508	SCARF2;SCARF2	0.0371794	0.140827345184609	0.0884228563666723
**cg15412971**	4	956119	DGKQ	0.00881184	0.0132802172069235	0.0884228563666723
**cg12660062**	10	92230339	LOC101926942	-0.065937	-0.149608709705552	0.0884228563666723

**cg24371217**	3	156402033	TIPARP	0.0245537	0.0380324531602961	0.0884228563666723
**cg18101314**	11	72504857	STARD10	0.0287924	0.69282388364639	0.0884228563666723
**cg15405931**	19	52693188	PPP2R1A	0.00860243	0.58989218624091	0.0884228563666723
**cg19044279**	18	658447	TYMS;C18orf56	0.0147594	0.564168355038449	0.0884228563666723
**cg00987699**	7	5229947	WIPI2;WIPI2;WIPI2;WIPI2;WIPI2;WIPI2;WIPI2	0.0529673	0.6467555501489	0.0884228563666723
**cg01976469**	18	56076257		-0.0503598	-0.092441802851527	0.0884228563666723
**cg26748435**	14	64854866	MTHFD1;MTHFD1;MIR548AZ	0.0220145	0.600261371942938	0.0884228563666723
**cg27475235**	16	58659721	CNOT1;CNOT1	-0.0832578	-0.154306331317932	0.0884228563666723
**cg15752230**	14	35005787	EAPP	-0.071998	-0.136064645301829	0.0884228563666723
**cg13588054**	7	126892578	GRM8	0.0101924	0.290129138254495	0.0884228563666723
**cg23924007**	4	140099401		0.00880979	0.524920607381924	0.0884402167465414
**cg21142038**	1	202183444	LGR6;LGR6;LGR6	0.0175064	0.388035933093821	0.0884631503591757
**cg10576257**	2	75428132	TACR1;TACR1	0.0298301	0.913569703882141	0.0885558629913202
**cg13332525**	6	99949744	USP45	-0.0614611	-0.110478855996015	0.0885881997552262
**cg03323770**	14	74960890	NPC2;ISCA2	0.00810729	0.488838282436922	0.0886268200056694
**cg07663404**	19	3979938	EEF2	0.0088309	0.0131774816106332	0.0886365890794188
**cg08857906**	1	28156999	PPP1R8;PPP1R8;PPP1R8	0.0659985	0.260817647687251	0.0886365890794188
**cg04365101**	17	55703965	MSI2;MSI2	-0.0108413	-0.0167249447474204	0.0886365890794188
**cg20615128**	7	140396907	NDUFB2;NDUFB2-AS1	0.00597842	0.539828959275201	0.0886370937928553
**cg10442913**	1	116915309	ATP1A1;ATP1A1	0.00854496	0.329239413401459	0.0886468227782772
**cg25164886**	16	88700624		0.0433969	0.587062919604477	0.0886468227782772
**cg11728900**	10	13203459	MCM10;MCM10	0.00940993	0.512002770089747	0.0886708643414291
**cg02084259**	15	34178478	AVEN	-0.045107	-0.0762311072363058	0.0887003598920323
**cg25847024**	1	111991376	WDR77;ATP5F1	0.0160966	0.446721827726696	0.0887003598920323
**cg18961370**	9	124262142	GGTA1P;GGTA1P	0.0153513	0.438532313217459	0.0887045024649253
**cg00996367**	12	39301695		-0.0305468	-0.0478977083450052	0.0887045024649253
**cg05840756**	5	114696346		-0.0491011	-0.0805896937447548	0.0887045024649253
**cg18922524**	12	54447136	HOXC4;HOXC4	0.00855981	0.382127900451824	0.0887045024649253
**cg00989217**	21	33651492	C21orf45	0.00953562	0.43157743804003	0.0887045024649253
**cg09957933**	15	57065793		0.00693919	0.0103199920419637	0.0887045024649253
**cg01610561**	19	34895766	PDCD2L	0.00924969	0.338652989969779	0.088773360431765
**cg07743799**	20	2821434	FAM113A;VPS16;VPS16	0.00877241	0.693962708344277	0.088773360431765
**cg19112349**	8	28244113	ZNF395	0.0154051	0.967407024406541	0.088773360431765
**cg14084056**	12	48744539	ZNF641	0.00903712	0.452955832385787	0.088773360431765
**cg21032292**	21	34395093		0.00962698	0.45745734108325	0.088788427805005
**cg24036292**	11	70416269	SHANK2;SHANK2	-0.0109096	-0.016260338551304	0.0888086780552478
**cg07785518**	2	140510062		-0.0648215	-0.112432108380904	0.0888086780552478
**cg22414994**	7	77619544		-0.0326882	-0.0526074825567689	0.0888086780552478
**cg03551378**	6	36309528		-0.0698275	-0.126075626082961	0.0888086780552478
**cg11380323**	1	220534666		-0.015055	-0.0227417292595675	0.0888086780552478
**cg07164875**	16	89788709	C16orf7;ZNF276;ZNF276	0.0258635	0.649086736222904	0.0888086780552478
**cg19800413**	6	108077539	SCML4;SCML4	0.0733687	0.115910620385264	0.0888086780552478
**cg01719220**	16	58035188	C16orf57;ZNF319	0.00958668	0.551270089573762	0.0888254393529388
**cg14667731**	6	28891990	TRIM27	0.00575718	0.36363219967237	0.0888989817382632
**cg21361127**	7	27766351		-0.0419402	-0.0678763854526501	0.0889302453829284
**cg12500976**	2	233498078	EFHD1;EFHD1	0.0234824	0.82236519057905	0.0889406020745895
**cg17669438**	3	133615173	RAB6B	0.0109713	0.387812901715694	0.0889406020745895
**cg26373942**	12	108523463	WSCD2	0.0306075	0.725335539952447	0.0889406020745895
**cg10772244**	12	6643804	GAPDH;GAPDH;GAPDH;GAPDH;GAPDH	0.0251031	0.644938874428515	0.0889406020745895
**cg21439852**	19	10628193	S1PR5;S1PR5	0.0327639	0.388205411575406	0.0889406020745895
**cg12646754**	1	2574751		0.0606272	1.81821963352498	0.0889406020745895
**cg24096934**	9	90589828	CDK20;CDK20;CDK20;CDK20;CDK20	0.0264909	0.531075252448357	0.0889406020745895
**cg27603843**	16	32926385		-0.0291786	-0.0476874318731588	0.0889406020745895
**cg17418440**	6	3069353		0.00956465	0.53196112093224	0.0889406020745895
**cg24480260**	17	80098801	CCDC57	-0.0495537	-0.0840753129259729	0.0889531855601125
**cg00583480**	8	134441362		0.00332889	0.00488741132664654	0.0889572462506623
**cg23995249**	3	132440400	NPHP3-AS1;NPHP3;NPHP3-ACAD11	-0.0500266	-0.137525897319688	0.0890449531799747
**cg01435564**	5	132150904	ANKRD43	0.00486217	0.00734057895712357	0.0890449531799747
**cg21667796**	11	63804119	MACROD1	0.0291891	0.995023463629163	0.0890504145849747
**cg14182682**	14	57857589	NAA30	-0.00863309	-0.525542590414428	0.0890504145849747
**cg18635246**	17	55021287	COIL	-0.0403807	-0.0649148293533908	0.0890580053948821
**cg00980047**	12	121642721		-0.0780793	-0.1970979142537	0.0890580053948821
**cg09252196**	1	113933337	MAGI3;MAGI3	0.0137642	0.607634146361661	0.0890580053948821
**cg18991677**	1	16846966		-0.0401832	-0.0852926395921857	0.0890580053948821
**cg16248933**	3	122003561	CASR	0.00395246	0.00580698206157762	0.0890580053948821
**cg11619775**	17	61920126	SMARCD2;SMARCD2	0.0274426	0.79836492136703	0.0890580053948821
**cg18665594**	5	101119420		0.0902522	0.263795381714835	0.0890580053948821
**cg13501117**	1	3566577	WDR8;WDR8	0.0128703	0.442832727139667	0.0890580053948821
**cg14857596**	12	72233493	TBC1D15;TBC1D15;TBC1D15;TBC1D15;TBC1D15;TBC1D15;TBC1D15	0.0230154	1.00552372321533	0.0890580053948821
**cg05403508**	1	47069957	MKNK1;MKNK1;MKNK1;MKNK1;MKNK1;MKNK1;MKNK1;MKNK1	0.00932662	0.443835922155401	0.0890580053948821
**cg24410546**	8	26371551	PNMA2	0.0045053	0.291342622560949	0.0890580053948821
**cg14036868**	2	38604442	ATL2;ATL2;ATL2	0.0335152	0.60967717154075	0.0890580053948821
**cg24848467**	6	28134517	ZNF389	-0.0722459	-0.157029535260242	0.0890580053948821
**cg13862983**	9	73028130	KLF9	0.0109747	0.464207157547605	0.0890580053948821
**cg07292028**	7	134044231		-0.0113265	-0.016985649382528	0.0890580053948821
**cg02506578**	3	25824554	NGLY1;NGLY1;NGLY1;NGLY1	0.00897952	0.509657461825112	0.0890580053948821
**cg01936481**	22	25882506		-0.0489258	-0.0837667598794427	0.0890580053948821
**cg18493085**	17	73474562	KIAA0195	0.00554829	0.00833775505517753	0.0890580053948821
**cg04963066**	8	146126972	ZNF250;ZNF250	0.0191352	0.793035892565235	0.0890580053948821
**cg00353773**	10	44880708	CXCL12;CXCL12;CXCL12	0.0134585	0.261540560311256	0.0890580053948821
**cg10396491**	8	123808147	ZHX2	-0.0485148	-0.101824723828751	0.0890580053948821
**cg09853387**	7	139877374	LOC100134229;JHDM1D	0.0304341	0.682970593052685	0.0890580053948821

**cg26832142**	1	66258441	PDE4B;PDE4B	0.0113967	0.407001666107987	0.0890580053948821
**cg24568647**	10	43903337	HNRNPF;HNRNPF;HNRNPF;HNRNPF	0.00901386	0.506248708375301	0.0890580053948821
**cg25552017**	1	945416		-0.0703949	-0.170372948377519	0.0890580053948821
**cg17222231**	14	105714673	BTBD6;BRF1;BRF1;BRF1;BRF1;BRF1;BRF1	0.0155752	0.665840362815223	0.0890580053948821
**cg25088355**	8	144691901	PYCRL	0.00372233	0.354437181334157	0.0890580053948821
**cg15391499**	5	112630623	MCC;MCC	0.00696992	0.481254965816211	0.0890580053948821
**cg22775801**	12	123636480		0.0296534	0.0501535206136403	0.0890580053948821
**cg06809691**	17	17380297	MED9	0.0154226	0.760210070440155	0.0890580053948821
**cg18716861**	20	43538761	PABPC1L;PABPC1L	0.0171476	0.76311069502501	0.0890580053948821
**cg24751717**	17	4642944	ZMYND15;CXCL16;CXCL16;CXCL16;CXCL16;ZMYND15	0.0238835	0.751715299608699	0.0890580053948821
**cg03320144**	17	14933536	CDRT7	-0.0285427	-0.0448370269569562	0.0890580053948821
**cg15975102**	19	8008829	TIMM44	0.0176649	0.609555757230171	0.0890580053948821
**cg06674310**	6	27357040	ZNF391	0.010605	0.250867258660337	0.0890580053948821
**cg07132926**	13	21480411		-0.0848535	-0.165495231962734	0.0890580053948821
**cg04490178**	19	3179545	S1PR4	0.0282289	0.768584882078409	0.0890580053948821
**cg05137680**	5	138729068	LOC389333	0.0126872	0.748507664448023	0.0890580053948821
**cg04314308**	19	19336150	NCAN	0.00859169	0.493056442506098	0.0890580053948821
**cg13301155**	15	82338574	MEX3B	0.0185433	0.737814115618871	0.0890580053948821
**cg13357518**	1	22263347	HSPG2	0.032915	0.450296119252778	0.0890580053948821
**cg17161020**	19	19302807	RFXANK;RFXANK;RFXANK;RFXANK;MEF2BNB;MEF2BNB;MEF2BNB-MEF2B;MEF2BN	0.0357417	0.309390984470248	0.0890580053948821
**cg07737482**	16	68631748		-0.0285964	-0.0447483153914435	0.0890580053948821
**cg11595203**	16	2098149	TSC2;TSC2;TSC2;NTHL1	0.0136175	0.714413915690609	0.0890580053948821
**cg19176999**	14	92414221	FBLN5	0.00890498	0.370799861441464	0.0890580053948821
**cg19712603**	13	53422777	PCDH8;PCDH8	0.0206135	0.512575259279322	0.0890580053948821
**cg05097593**	17	34890677	MYO19;MYO19;MYO19;PIGW	0.0141653	0.473303164380058	0.0890580053948821
**cg07590922**	11	76152669		-0.0776867	-0.160174365831787	0.0890580053948821
**cg12668472**	17	63327954		-0.0761632	-0.142126806888266	0.0890580053948821
**cg07195197**	16	1662150	IFT140	0.014144	0.705710585780396	0.0890580053948821
**cg07909497**	12	9600950	DDX12	0.0171893	0.459367563245726	0.0890580053948821
**cg00974149**	17	56296512	MKS1;MKS1	0.0331975	0.725645667544736	0.0890580053948821
**cg13672103**	12	57985290	PIP4K2C;PIP4K2C;PIP4K2C;PIP4K2C	0.0415711	0.424697129664964	0.0890580053948821
**cg09964204**	3	66024343	MAGI1;MAGI1;MAGI1;MAGI1;MAGI1;MAGI1	0.0223501	0.431142704199004	0.0890580053948821
**cg14864994**	12	6496214	LTBR	-0.034919	-0.055076682186541	0.0890580053948821
**cg01603004**	1	236462959		-0.0253645	-0.0396638129214135	0.0890580053948821
**cg05838956**	11	6704805	MRPL17	0.0142646	0.580152775399249	0.0890580053948821
**cg13636983**	9	135838862	GFI1B	-0.0222539	-0.0339747168557058	0.0890580053948821
**cg19853434**	10	14286813	FRMD4A	-0.0315731	-0.0558757169116242	0.0890580053948821
**cg00973823**	1	32226349	BAI2	0.0436339	0.432417633422928	0.0890693142187285
**cg08163767**	2	241500709	DUSP28;ANKMY1	0.00846791	0.690112955348012	0.0890693142187285
**cg21826292**	9	80850930	CEP78;CEP78	0.0123248	0.615814766623267	0.0891450630253458
**cg04509133**	1	167734853	MPZL1;MPZL1;MPZL1	-0.0258336	-0.040253048589882	0.0891450630253458
**cg25142954**	15	41245554	CHAC1;CHAC1	0.0241555	0.855252461820159	0.0891450630253458
**cg14224553**	10	63875107		-0.0607726	-0.126995253770261	0.0891450630253458
**cg04270788**	1	89356964	GTF2B	0.00622963	0.457471148976015	0.0891450630253458
**cg07937578**	4	25487481		-0.0646896	-0.116574695180118	0.0891450630253458
**cg09309286**	17	32960898	TMEM132E	-0.069266	-0.127245925906624	0.0891450630253458
**cg11505936**	16	58005235	CNGB1;CNGB1;CNGB1	0.0120789	0.0186254358468773	0.0891450630253458
**cg15717656**	2	74709666	TTC31;TTC31;CCDC142	0.0192388	1.25692211894078	0.0891450630253458
**cg18073966**	1	178482209	TEX35;TEX35;TEX35;TEX35	0.011501	0.018159337611149	0.0891450630253458
**cg08165971**	2	233792394	NGEF;NGEF	0.022152	0.48656460953514	0.0891450630253458
**cg23131955**	1	65457091	LINC01359	-0.0275666	-0.0460747486764094	0.0891450630253458
**cg01609705**	8	38240669	WHSC1L1;WHSC1L1	0.00553507	0.437297595018231	0.0891450630253458
**cg14340610**	19	37568961	ZNF420	0.00995753	0.485096692216678	0.0891450630253458
**cg22845819**	19	58514331	ZNF606;ZNF606;LOC100128398	0.0425861	1.29250457156914	0.0891450630253458
**cg08740862**	11	62314417	AHNAK;AHNAK	0.0367308	0.74971013776671	0.0891450630253458
**cg07951519**	17	4607306	PELP1	0.0174443	0.347339590393983	0.0891450630253458
**cg24892765**	4	56252140	SRD5A3-AS1	-0.00981506	-0.0145062517785612	0.0891450630253458
**cg10991578**	14	96671020	BDKRB2	0.0134145	0.224925774458096	0.0891450630253458
**cg00288562**	7	23571645	TRA2A;TRA2A	0.00764393	0.568286843263829	0.0891450630253458
**cg06908806**	20	42136129		0.0178472	0.526829314248015	0.0891450630253458
**cg05315621**	14	99375443		-0.0185535	-0.0281230622086155	0.0891450630253458
**cg22802813**	12	95942761	USP44;USP44	0.0314353	0.44826737557051	0.0891450630253458
**cg22126266**	1	180199560	LHX4;LHX4	0.00642825	0.49897798404026	0.0891450630253458
**cg13957451**	1	111991591	ATP5F1;WDR77	0.0104205	0.51967500671238	0.0891450630253458
**cg04508332**	16	89916780	SPIRE2	0.00739312	0.0109045413080596	0.0891450630253458
**cg15749350**	5	147257225	SCGB3A2	-0.020808	-0.0324361853954206	0.0891450630253458
**cg27227597**	2	218661518		-0.0334775	-0.0563838878803841	0.0891450630253458
**cg11857246**	1	11741390	MAD2L2;MAD2L2	0.0363992	1.35341266670335	0.0891450630253458
**cg17587023**	1	206223592	AVPR1B	0.0106078	0.570273594102875	0.0891450630253458
**cg23289197**	7	16685473	BZW2;BZW2;ANKMY2;BZW2	0.0115621	0.5256039909763	0.0891450630253458
**cg10455757**	9	35111424	KIAA1539	0.0195841	0.608365894025089	0.0891450630253458
**cg08209577**	8	145635217	CPSF1	0.0111943	0.39758631936003	0.0891450630253458
**cg09786084**	17	60670351	TLK2;TLK2;TLK2	-0.0639215	-0.121222108586389	0.0891450630253458
**cg01582807**	7	99070515	ZNF789;ZNF789;ZNF789;ZNF789	0.0128593	0.536843172613344	0.0891450630253458
**cg02764239**	2	74615548	LOC100189589	0.00441999	0.00655576493224424	0.0891450630253458
**cg23240961**	12	57634410	NDUFA4L2	-0.00624213	-0.330744435152292	0.0891450630253458
**cg22447380**	5	55451073	ANKRD55	0.0446263	0.540600873196172	0.0891450630253458
**cg13283153**	8	9756828		0.00661476	0.198476422192831	0.0891450630253458
**cg01230508**	14	65569312	MAX;MAX;MAX;MAX;MAX;MAX	0.0072729	0.306164490401547	0.0891450630253458
**cg19281363**	13	42031291	C13orf15	0.0114889	0.564390406409639	0.0891450630253458
**cg10450899**	17	685900	RNMTL1;GLOD4	0.0188629	0.872261106546768	0.0891450630253458
**cg01035689**	10	118608698		0.0238443	0.770680858257093	0.0891450630253458

**cg09055355**	8	130951979	FAM49B;FAM49B	0.0234576	0.717194027577518	0.0891450630253458
**cg01679663**	6	81027072	BCKDHB;BCKDHB	-0.0591739	-0.111443682214744	0.0891450630253458
**cg24312390**	7	149158098	ZNF777	0.00381455	0.369983046271768	0.0891450630253458
**cg12951098**	5	157170574	11,00 LSM	0.00311224	0.357623044879138	0.0891450630253458
**cg18690833**	20	13972022	SEL1L2	-0.0749749	-0.14125784436807	0.0891450630253458
**cg06409997**	2	220110347	GLB1L;GLB1L;STK16;STK16	0.0169139	1.13327448540117	0.0891450630253458
**cg20747266**	6	142409507	NMBR	0.0423351	0.262735331483575	0.0891450630253458
**cg05279076**	8	103666388	KLF10;KLF10;KLF10;KLF10	0.00555489	0.305705893719144	0.0891450630253458
**cg10099795**	21	16681618		-0.038726	-0.133699303285653	0.0891450630253458
**cg01039083**	2	241857215		0.00958469	0.0144054676402346	0.0891450630253458
**cg20275133**	1	1342361	MRPL20	0.0137911	0.624307346557911	0.0891450630253458
**cg01779740**	8	142366708	GPR20	-0.0077299	-0.0115213097564728	0.0891450630253458
**cg04179482**	20	34360400	PHF20	0.0050403	0.41572363706409	0.0891450630253458
**cg07831032**	1	46629507		-0.0649677	-0.155546747282263	0.0891450630253458
**cg07702644**	19	43936935		-0.0508455	-0.0892774006246176	0.0891450630253458
**cg09209803**	6	33588932	ITPR3	0.0152452	0.543450045840447	0.0891450630253458
**cg18696576**	6	34203630	HMGA1;HMGA1;HMGA1;HMGA1;HMGA1	0.0251394	0.307998476214534	0.0891450630253458
**cg04123995**	17	71308339	CDC42EP4	0.0235	0.947052857694835	0.0892354232776816
**cg01540571**	1	15911089	AGMAT	0.0227149	0.805879183222799	0.0892394796681224
**cg02884346**	4	6271501	WFS1;WFS1	0.0315346	0.987549557150615	0.0892881772254871
**cg19039291**	17	7229608	NEURL4;NEURL4	-0.0107414	-0.0158829771309281	0.0893452120997153
**cg25135778**	20	60736870	SS18L1;SS18L1;SS18L1;SS18L1	0.00802356	0.0118744556397144	0.0893836408066762
**cg24061931**	15	75654619	MAN2C1;MAN2C1;MAN2C1;MAN2C1	-0.0235554	-0.0363803361138928	0.0894124719428491
**cg10992925**	3	50192260	SEMA3F	0.024019	0.45032402828694	0.0894332128871207
**cg04524552**	19	4670575	C19orf10	0.0241565	0.612758894518592	0.0894332128871207
**cg08886760**	8	123793830	ZHX2	0.00882801	0.449200257944127	0.0894332128871207
**cg10036402**	14	77787255	POMT2;GSTZ1;GSTZ1;GSTZ1;GSTZ1;GSTZ1	0.0345778	0.265325466534982	0.0894332128871207
**cg07495177**	10	88740955	AGAP11	-0.0584936	-0.166011755842338	0.0894332128871207
**cg16508623**	12	102271114	DRAM1;DRAM1	0.0102659	0.963921617193752	0.0894706937839015
**cg08184047**	17	79849980	ANAPC11;ANAPC11;ANAPC11;ANAPC11;ANAPC11;ANAPC11;ANAPC11;ANAPC11;TH	0.0264166	0.727305381612241	0.0895139620795204
**cg08114293**	3	10028395	TMEM111;TMEM111	0.0103273	0.365282388224807	0.0895139620795204
**cg09510269**	6	30292743	HCG18;HCG18	-0.0434588	-0.0711123862581692	0.0895139620795204
**cg09514401**	8	72988022	TRPA1	0.0133084	0.314737746246823	0.0895139620795204
**cg23749739**	3	183979443	ECE2;CAMK2N2	0.00979796	0.455280706977219	0.0895139620795204
**cg07924079**	2	197504206	CCDC150	0.0132508	0.429347382087542	0.0895159799186584
**cg13416901**	9	136019673	RALGDS	0.0180962	0.853767413144674	0.0895159799186584
**cg18573884**	12	51639577	SMAGP;SMAGP;DAZAP2	0.00571346	0.00840682410507371	0.0895374210148951
**cg23813012**	1	14026482	PRDM2	0.00608188	0.422462301042458	0.0895452503693674
**cg14632335**	2	70882265		-0.0203802	-0.0316908078326582	0.0895452503693674
**cg19071293**	17	7765793		-0.0662549	-0.11610280098628	0.0895452503693674
**cg11996632**	16	70284893	EXOSC6;EXOSC6	0.0125357	0.860253620006769	0.089554978030876
**cg14853922**	1	179051253	TOR3A;TOR3A	0.0234908	0.988130147996323	0.089554978030876
**cg08496100**	14	103655428	LINC00605	0.0223012	0.507761598128574	0.089587485225354
**cg19422250**	15	41086034	DNAJC17	-0.0666096	-0.137938689353172	0.0896210217151902
**cg27629898**	8	142070851		-0.0196572	-0.0312438163555979	0.0896210217151902
**cg21012729**	14	51297401	NIN;NIN;NIN	0.0378978	0.63093522547691	0.0896210217151902
**cg13944275**	9	140024112		0.00558581	0.406131831157119	0.0896210217151902
**cg19831545**	1	198076518		-0.0248574	-0.0381393893943132	0.0896575234009509
**cg14926867**	9	4679457	CDC37L1-AS1;CDC37L1-AS1;CDC37L1	0.00280732	0.343026019310008	0.0896575234009509
**cg03781505**	17	16310359		0.0227022	0.673632877400796	0.0896575234009509
**cg19821527**	1	220375253	RAB3GAP2;SNORA36B	-0.0890064	-0.169627759273318	0.0896575234009509
**cg09214759**	10	5328293		-0.105813	-0.221135004207985	0.089702595356346
**cg00771688**	2	120124593	C2orf76;DBI;DBI;DBI;DBI	0.0117639	0.462552856977606	0.089702595356346
**cg18552663**	20	388903	RBCK1;RBCK1;RBCK1;RBCK1	-0.00331404	-0.337423753836614	0.089702595356346
**cg03477080**	3	46448920	CCRL2;CCRL2;CCRL2	0.0245039	0.60536131642699	0.089702595356346
**cg13189086**	15	23034679	NIPA2;NIPA2;NIPA2;NIPA2	0.015691	0.76488729001534	0.089702595356346
**cg03356490**	19	35605867	FXYD3;FXYD3;FXYD3;FXYD3;FXYD3;FXYD3;FXYD3;FXYD3	0.0464093	0.517418501573625	0.089702595356346
**cg15630908**	7	72041048	TYW1B	-0.0065032	-0.00965042413312846	0.0897141419069085
**cg04876095**	11	62296270	AHNAK;AHNAK	-0.0484057	-0.0783060631192003	0.0897913617174765
**cg01791302**	10	12386716		-0.0273726	-0.0428353380989886	0.0897913617174765
**cg18328612**	19	4676623	DPP9	0.00517628	0.00761640581129525	0.0898550015146941
**cg22351824**	22	24084185	ZNF70	-0.0593128	-0.162000256852345	0.0898550015146941
**cg10258271**	2	31361593	GALNT14	0.0240434	0.50008034899802	0.0898550015146941
**cg06892384**	5	84671738		-0.0351722	-0.277148525091371	0.0898550015146941
**cg00208412**	10	99609481	GOLGA7B	0.00745647	0.836775522597861	0.0898550015146941
**cg22316269**	4	571371		0.00865584	0.429121131278236	0.0898807013231869
**cg11475510**	22	30752584	SF3A1;SF3A1;CCDC157	0.0149317	0.557600644412581	0.0898981227194644
**cg04929759**	12	133016214		0.0443186	0.583351138439602	0.0899110251106411
**cg02811627**	14	103395368	AMN	0.0171068	0.353051097319935	0.0899110251106411
**cg27175128**	11	66824169	RHOD	0.00992061	0.557699839230153	0.0899193943829086
**cg15326320**	2	172967665	DLX2	0.00774018	0.570249253982562	0.0899218011791305
**cg06035839**	1	151119150	SEMA6C	0.00758489	0.554831571396892	0.0899460854241058
**cg11896633**	8	144816335	FAM83H	0.00679912	0.533979048498942	0.0899957490546553
**cg00631837**	22	45404212	PHF21B;PHF21B	0.0269475	0.391109137604856	0.0900323072548394
**cg06323912**	16	57481690	CIAPIN1;COQ9	0.00980501	0.394080990277095	0.0900335594097384
**cg21250436**	12	28570321	CCDC91	-0.0460345	-0.0766218192762251	0.0900335594097384
**cg02964602**	2	203130213	NOP58	0.0752825	0.152876415256774	0.0900645673396424
**cg01301138**	16	82660630	CDH13;CDH13	0.0174443	0.36818003493402	0.0900645673396424
**cg02184643**	11	10879647	ZBED5;ZBED5	0.0242083	0.636736560379956	0.0901762175075386
**cg03285219**	7	1499161	MICALL2	0.0141184	0.490779460139883	0.0901919697431964
**cg10435123**	12	111127010	HVCN1;HVCN1	-0.00370207	-0.287078820934078	0.0901919697431964
**cg05751616**	12	6652601	IFFO1;IFFO1;IFFO1	-0.066178	-0.122194200738404	0.0902472450974356

**cg24756528**	16	4466888	CORO7	0.0401841	1.35431194815326	0.0902609423751755
**cg06435847**	17	3562636	CTNS;CTNS	-0.0130218	-0.0196138950509004	0.0902672345691796
**cg01224647**	16	25115281	LCMT1-AS1	-0.0621152	-0.121414245636576	0.0902672345691796
**cg05550371**	3	49841167	C3orf54	0.0173444	0.697935622639103	0.0902672345691796
**cg08522257**	3	152879782	RAP2B	0.0134015	0.48171656479822	0.0902672345691796
**cg22075671**	5	133747798	CDKN2AIPNL	0.0105847	0.556025178229459	0.0902672345691796
**cg07041883**	5	76476158		0.0125934	0.536133812931479	0.0902672345691796
**cg21401143**	8	19171452	SH2D4A;SH2D4A;SH2D4A	0.0225114	0.577192282494582	0.0902672345691796
**cg09571572**	17	78010330	CCDC40;TBC1D16	0.00692833	0.501410686533137	0.0903046051667116
**cg09818962**	17	39822215		0.0127285	0.500705821789464	0.0903139475354995
**cg17651140**	1	40549220	PPT1;PPT1	-0.0168746	-0.0252953535772326	0.0903139475354995
**cg16970804**	5	43042108	LOC153684	0.00861057	0.581492771307386	0.090386873972164
**cg18963377**	6	167316609	RPS6KA2-AS1	0.0105852	0.0157964099072482	0.0904708299857034
**cg17090968**	12	46663694	SLC38A1;SLC38A1	0.00888481	0.319414301765225	0.0904915605356414
**cg21561038**	10	21462743	NEBL-AS1;NEBL-AS1;NEBL;NEBL	0.0225576	0.972798763316392	0.0904915605356414
**cg14471727**	14	97263643	VRK1	0.0220367	0.471772229095459	0.090548874623423
**cg01538984**	7	140396422	LOC100134713;NDUFB2	0.0113637	0.483280669183773	0.090548874623423
**cg11083503**	10	118508385		-0.054397	-0.107211260511199	0.090548874623423
**cg21737632**	10	65281095	REEP3	-0.0353494	-0.442650488267473	0.090548874623423
**cg14021555**	22	43485536	TTLL1;TTLL1	0.0173863	0.517045574367343	0.0905661510222531
**cg11113103**	19	49122630	RPL18;RPL18;RPL18;RPL18;SPHK2;SPHK2;SPHK2;SPHK2;SPHK2;SPHK2;RPL	0.0288027	0.973889138784344	0.0905763321411287
**cg18209104**	1	14893756		-0.0325058	-0.0523062273549708	0.0905763321411287
**cg27540114**	14	61189963	SIX4	0.00585273	0.326416693037619	0.0906055321290435
**cg25631940**	20	43589250	TOMM34	0.006369	0.375830443582782	0.0906055321290435
**cg03540175**	3	49236946	CCDC36;CCDC36;CCDC36	0.0639354	0.398502278536853	0.090627055133043
**cg13743031**	4	178231244	NEIL3	0.00667902	0.301893496514918	0.090627055133043
**cg18714585**	12	123868665	SETD8	0.0328051	0.65817420479791	0.090627055133043
**cg09463890**	4	37960795	PTTG2;TBC1D1	-0.0532027	-0.10900392899386	0.090627055133043
**cg06630141**	5	133862209	JADE2;JADE2;JADE2;JADE2	0.011873	0.450522274477115	0.090627055133043
**cg01379428**	5	180690432	TRIM52-AS1;TRIM52-AS1;TRIM52-AS1;TRIM52-AS1	-0.0543412	-0.0927053804116839	0.090627055133043
**cg06202686**	2	170336108	BBS5	0.00988799	0.46130756875688	0.090627055133043
**cg16754864**	2	96194596		-0.0179942	-0.0270634005272081	0.090627055133043
**cg18483269**	19	11849691	ZNF823;ZNF823	0.00576264	0.376976251134755	0.090627055133043
**cg03933646**	1	55317098	DHCR24	-0.00663669	-0.00980261003645949	0.090627055133043
**cg15803261**	12	11049876	PRH1-PRR4;PRH1;PRH1	-0.0406528	-0.0685927257379446	0.090627055133043
**cg15677368**	1	245133209	EFCAB2;EFCAB2;EFCAB2;EFCAB2;EFCAB2;EFCAB2	0.0365588	0.360627849583909	0.090627055133043
**cg10209223**	2	3523546	ADI1	0.00750741	0.497055329712522	0.090627055133043
**cg10073303**	12	123718004	C12orf65;C12orf65;C12orf65;MPHOSPH9;MPHOSPH9;C12orf65	0.0193268	0.822434352161164	0.090627055133043
**cg09843573**	11	133797846	IGSF9B	0.026912	0.633021424478584	0.090627055133043
**cg15426734**	16	67695272	PARD6A;ACD;ACD;PARD6A;ACD	0.0217664	0.479958317091918	0.090627055133043
**cg04647948**	2	206950423	INO80D	0.012844	0.444301651235364	0.090627055133043
**cg09172043**	10	101025916		-0.0461407	-0.0841347603698404	0.090627055133043
**cg15735388**	4	89786856	FAM13A	-0.0429771	-0.0705344307343358	0.090627055133043
**cg07326720**	5	35614133		-0.0483159	-0.0957960738890387	0.090627055133043
**cg19357918**	11	63953256	STIP1	0.0170446	0.809340783609111	0.090627055133043
**cg09516144**	16	15982637	C16orf63	0.0104131	0.595289380028636	0.0906625193107016
**cg24701257**	7	73097740	WBSCR22;DNAJC30	0.0135328	0.581450901979867	0.0906625193107016
**cg18293298**	3	10053022	LOC401052	0.012345	0.431199817416152	0.0906625193107016
**cg20994084**	17	79269488	SLC38A10;SLC38A10	0.0137577	1.47901351876957	0.0906625193107016
**cg01772700**	19	56904442	ZNF582	-0.0473011	-0.147846031960213	0.0906625193107016
**cg25098497**	12	13254403	GSG1;GSG1	0.00432158	0.270244784333381	0.0907011818065967
**cg14732699**	8	128750039	MYC	0.0220367	0.594101175618182	0.0907011818065967
**cg07304114**	20	32309023	PXMP4;PXMP4	0.00698195	0.0103979250493127	0.0907651646858184
**cg13956914**	16	75600119	GABARAPL2	0.0323995	1.09535402912993	0.0907651646858184
**cg08721076**	2	172967670	DLX2	0.00347544	0.353647396323012	0.0907651646858184
**cg00823789**	12	124001205	RILPL1	-0.0728208	-0.135268242978773	0.0907904666266143
**cg09932730**	7	135198360		-0.0615297	-0.119469733514989	0.0907970210421057
**cg21463790**	7	14025907	ETV1;ETV1;ETV1;ETV1;ETV1;ETV1;ETV1;ETV1;ETV1	-0.0378508	-0.0645613065241993	0.0907970210421057
**cg20557595**	14	45381239		0.0504605	0.245267700704839	0.0907970210421057
**cg00852573**	6	73331405	KCNQ5;KCNQ5;KCNQ5;KCNQ5;KCNQ5	0.0356088	1.50847752051294	0.0907970210421057
**cg06820859**	13	21564784	LATS2	-0.026086	-0.0409398256156025	0.0907970210421057
**cg01173563**	15	73027500	BBS4;BBS4;BBS4;BBS4	0.013642	0.0211837781945181	0.0907970210421057
**cg22860780**	7	48128351	UPP1;UPP1	0.0209302	0.480281322971092	0.0907970210421057
**cg00350409**	1	38354007	INPP5B;INPP5B;INPP5B;INPP5B	0.0105846	0.0160875338857157	0.0907970210421057
**cg01896926**	17	685509	GLOD4;GLOD4;RNMTL1	0.0295923	1.27690720842614	0.0907970210421057
**cg25984791**	11	108535797	DDX10	0.010009	0.431877211691899	0.0907970210421057
**cg13654902**	19	38853053	CATSPERG	0.0650196	1.27167914817665	0.0907970210421057
**cg10628818**	1	38100255	RSPO1;RSPO1	0.012648	0.285556145723062	0.0907970210421057
**cg21841682**	14	69445844	ACTN1;ACTN1;ACTN1;ACTN1;ACTN1;ACTN1	0.0126227	0.856297785312806	0.0908179133432813
**cg25809066**	19	58962741	ZNF324B	0.0151833	0.551129613497316	0.0908179133432813
**cg15695045**	1	204330069	PLEKHA6	-0.133814	-0.264596282317313	0.0908179133432813
**cg11582100**	2	171673207	GAD1;GAD1;GAD1;GAD1	0.00238725	0.288746415404093	0.0908179133432813
**cg26115218**	3	149756649		-0.0166513	-0.0257113235964344	0.0908179133432813
**cg01181817**	3	49941376	MST1R	0.00665833	0.604988364419163	0.0908179133432813
**cg08554936**	19	46519866	CCDC61	0.0302985	0.76424603553246	0.0908179133432813
**cg11888151**	22	21272339	CRKL	0.00477918	0.506321707231244	0.0908179133432813
**cg23587044**	3	196777478	DLG1;DLG1;DLG1;DLG1;DLG1;DLG1	-0.115322	-0.327651350252905	0.0908675146196271
**cg11726911**	1	6241120	CHD5	0.018656	0.572196649779756	0.0908675146196271
**cg23517677**	11	62528272	POLR2G	-0.0298919	-0.0457578868862335	0.0908675146196271
**cg25009629**	6	41748519	FRS3;PRICKLE4;PRICKLE4	0.0230464	0.838642369727285	0.0909180766285681
**cg08616681**	16	718778	RHOT2	0.0294615	0.429907326735942	0.0909180766285681
**cg23829577**	6	160183769	ACAT2	0.0120205	0.599659922292844	0.0909180766285681

**cg07090470**	3	43437351	ANO10;ANO10;ANO10;ANO10;ANO10	-0.0381264	-0.0627594979164546	0.0909180766285681
**cg04977109**	17	46125622	NFE2L1	0.0255535	0.994010442747188	0.0909180766285681
**cg14266436**	19	34302242	KCTD15;KCTD15;KCTD15	-0.0436332	-0.0720338003432369	0.0909180766285681
**cg11128799**	2	42721159	KCNG3;KCNG3;MTA3;KCNG3;KCNG3	0.0410464	0.142134951470348	0.0909180766285681
**cg24083067**	19	48972665	CYTH2;CYTH2;CYTH2;CYTH2	0.0195399	0.708152850819588	0.0909180766285681
**cg17524151**	16	475494	RAB11FIP3	0.0225701	0.831619308960316	0.0909622749539427
**cg09481234**	17	8013680	ALOXE3;ALOXE3	0.0295715	0.450885535546941	0.0909622749539427
**cg22136703**	16	2255607	MLST8;MLST8;MLST8;MLST8	0.0217289	0.561042207780257	0.0909622749539427
**cg09662051**	2	162164883	PSMD14;PSMD14	0.00473581	0.34871376231168	0.0909622749539427
**cg19287103**	12	7055672	PTPN6	0.0143012	0.428601945313993	0.0909622749539427
**cg21724010**	6	164990879		-0.056111	-0.145089689602394	0.0909622749539427
**cg24072640**	20	48291209	B4GALT5	-0.0946469	-0.181448052457411	0.0909622749539427
**cg02280021**	10	134880594		-0.0114874	-0.0170875588986747	0.0909622749539427
**cg25023275**	18	35146450	BRUNOL4;BRUNOL4;BRUNOL4;BRUNOL4	0.0139402	0.319112821936367	0.0909622749539427
**cg27590781**	2	152266272	RIF1	0.00476435	0.398264321947668	0.0909622749539427
**cg00574251**	2	164965991		-0.0327128	-0.0518636098685364	0.0909622749539427
**cg18635645**	1	85667791	SYDE2	0.00840205	0.32373197045284	0.0909622749539427
**cg03309421**	7	32535387	LSM5;AVL9;LSM5	0.0222	0.667292660228334	0.0910017133229697
**cg17389835**	19	46147025	EML2;EML2	0.0136172	0.661453203578696	0.0910017133229697
**cg05289466**	19	5057455	KDM4B	-0.0520533	-0.0930785053363667	0.0910017133229697
**cg03868679**	2	160595278	MARCH7;MARCH7;MARCH7;MARCH7	-0.0697049	-0.180081163733322	0.0910017133229697
**cg12994587**	22	41777817	TEF;TEF	0.0365332	0.889545876706796	0.091005024436104
**cg06467674**	4	159315123		-0.0380307	-0.0704208894072944	0.091005024436104
**cg23499823**	11	507502	RNH1;RNH1;RNH1;RNH1;RNH1;RNH1;RNH1;RNH1	0.01829	0.701056554128925	0.091005024436104
**cg03521358**	1	9242937		0.0195668	0.448409532283678	0.091005024436104
**cg09238957**	16	46723420	ORC6L;VPS35	0.0303459	1.12074490834003	0.091005024436104
**cg15893127**	1	153756397		0.0139728	0.427767254112897	0.091005024436104
**cg13914708**	2	15804963		-0.0128349	-0.0194393878993528	0.091005024436104
**cg21809257**	1	154955635	FLAD1;FLAD1	0.00361625	0.26393203057552	0.091005024436104
**cg16057435**	5	75912664	F2RL2;IQGAP2	-0.0376022	-0.0612158583995704	0.091005024436104
**cg04106948**	2	104408389		-0.0392635	-0.0751924901014443	0.091005024436104
**cg11992550**	15	89533607		-0.0875043	-0.193949580581377	0.091005024436104
**cg22555392**	3	50649683	CISH;CISH	0.0034021	0.283800629054701	0.091005024436104
**cg06657142**	10	123357993	FGFR2;FGFR2;FGFR2;FGFR2;FGFR2	0.0163639	0.38668401095626	0.091005024436104
**cg07190698**	4	331458	ZNF141	0.0139708	0.64537351703444	0.091005024436104
**cg23167456**	1	27942186	FGR;FGR;FGR	0.00206031	0.00302582984556791	0.0910898520296624
**cg26600181**	4	7105115	FLJ36777	0.0165686	0.623311509406897	0.0911305496711236
**cg07318284**	4	154170590	TRIM2;TRIM2	-0.00442221	-0.272247935073215	0.0911305496711236
**cg09629119**	3	71547974	FOXP1;FOXP1;FOXP1;FOXP1;FOXP1	-0.0333791	-0.0529668284216774	0.0911305496711236
**cg21028463**	17	74733682	MIR636;SFRS2;MFSD11	0.0419539	1.08290613232277	0.0911305496711236
**cg01683883**	16	66613053	CMTM2	0.0176762	0.652362151782283	0.0911335855834963
**cg16344026**	5	149109979	PPARGC1B	0.0327061	0.597119350741966	0.0911335855834963
**cg20366603**	17	7218821	GPS2	0.0159375	0.499430838044475	0.0911335855834963
**cg00391018**	13	49049400	RB1	-0.0341669	-0.052897805555932	0.0911715026045264
**cg27268717**	7	56032047	GBAS	0.0124652	0.516629012306463	0.0911724132407382
**cg21066720**	6	32966587		-0.0159653	-0.0244203454446546	0.0911750795028031
**cg20526663**	16	22385655	CDR2;CDR2	0.0102539	0.397866286995331	0.0912114975110082
**cg09286909**	1	213031599	FLVCR1-AS1;FLVCR1-AS1;FLVCR1;FLVCR1	0.00984548	0.685187971894209	0.09121703006949
**cg07934232**	19	36619082		0.0177086	0.84149487622946	0.0912179761210085
**cg01496602**	7	156263586		-0.0351489	-0.0572389093903584	0.0912305539694855
**cg00290498**	5	1230850	SLC6A18	-0.0322519	-0.0523115662260083	0.0912305539694855
**cg01321158**	16	31128800	MYST1;MYST1	0.0210209	0.936964083093458	0.0912357120677123
**cg17896927**	15	93617030	RGMA;RGMA;RGMA;RGMA;RGMA;RGMA;RGMA	0.0357242	0.489848670908688	0.0912357120677123
**cg20655434**	11	67159118	LOC100130987;RAD9A	-0.00196301	-0.273133077448879	0.0912357120677123
**cg04409495**	17	38083144	ORMDL3	0.014484	0.653615811025692	0.0912357120677123
**cg08112354**	14	105452552	C14orf79	0.0148657	0.756126969899312	0.0912357120677123
**cg21809762**	11	94702500	CWC15	-0.017885	-0.026979992014506	0.0912357120677123
**cg17872999**	7	104653783	MLL5;LOC100216545;MLL5	0.0075214	0.53852431797974	0.0912357120677123
**cg20487890**	1	185402344		-0.0624233	-0.104820846172299	0.0912357120677123
**cg03710353**	5	1491489	LPCAT1	0.0175982	0.0271833305169448	0.0912357120677123
**cg24266670**	11	115630629		0.0330205	0.577637797104943	0.0912357120677123
**cg24632597**	12	121648093	P2RX4	0.00937731	0.617982355521267	0.0912357120677123
**cg26922444**	6	24911204	FAM65B	0.0271162	0.53156073615019	0.0912379497460371
**cg10458392**	19	3600539	TBXA2R;TBXA2R	-0.0382267	-0.0650391571357224	0.091500197675576
**cg11065931**	1	89487064	GBP3	-0.0843303	-0.231955325241039	0.091546023514121
**cg07261517**	22	20105424	RANBP1;TRMT2A;TRMT2A	0.00722251	0.478415826032103	0.091546023514121
**cg13840089**	9	80201784	GNA14	-0.0435847	-0.0732890116915495	0.091546023514121
**cg01769392**	6	169286420		-0.0360021	-0.0651291554626421	0.091546023514121
**cg11755740**	8	9413382	TNKS	0.0113874	0.709383650034987	0.091546023514121
**cg11217574**	12	56522261	ESYT1	0.00875956	0.509459233398196	0.091546023514121
**cg05002732**	11	19262474	E2F8;E2F8	0.0113302	0.388847343868593	0.091546023514121
**cg24450312**	1	206681158	RASSF5;RASSF5	0.0270271	0.587785271475057	0.0915586925899091
**cg10480748**	5	176730599	RAB24;RAB24;RAB24;RAB24;RAB24;RAB24;PRELID1;PRELID1	0.00831445	0.640943503160943	0.0915586925899091
**cg04966789**	14	35591779	PPP2R3C;KIAA0391;KIAA0391	0.0127574	0.557141669541884	0.0916396353278088
**cg02002281**	11	66053209	YIF1A;YIF1A;YIF1A;YIF1A	0.0159951	0.0242269332593648	0.0916560153278113
**cg02620388**	7	100271115	GNB2	0.00698103	0.414424913073269	0.0916560153278113
**cg12542506**	16	58133216		-0.00558149	-0.00820250317866611	0.0917033076388215
**cg03909721**	19	3512684	FZR1	0.012279	0.0192574423896919	0.0917691562909892
**cg17951368**	11	17298273	NUCB2	0.00619459	0.354976615508115	0.0917691562909892
**cg10668512**	15	42565522	GANC;TMEM87A;TMEM87A	0.0218945	0.540799638595639	0.0917691562909892
**cg00504756**	16	74330795	PSMD7;PSMD7	0.0370362	0.96953735566908	0.0917691562909892
**cg15000794**	7	86849844	C7orf23	0.0121189	0.690090082947626	0.0917691562909892

**cg07758936**	3	27525996	SLC4A7	0.00636751	0.401775485304928	0.0917691562909892
**cg00322559**	10	99212766	ZDHHC16;ZDHHC16;ZDHHC16;ZDHHC16;ZDHHC16;ZDHHC16;ZDHHC16;ZDHHC16	-0.0100553	-0.0148376686434661	0.0917691562909892
**cg14321859**	8	13010622	DLC1	-0.0541903	-0.092270244828126	0.0917691562909892
**cg20351608**	2	32581948	BIRC6	0.0310873	0.691429029024928	0.0917691562909892
**cg25703309**	16	8962583	CARHSP1;CARHSP1;CARHSP1;CARHSP1;CARHSP1;CARHSP1	0.0388062	0.758502957889014	0.0917841672903965
**cg02257203**	17	75743704		0.0097029	0.0146031470719134	0.0918608400549888
**cg25467876**	4	6566117	PPP2R2C	0.00590903	0.258651281381932	0.0918608400549888
**cg03012876**	2	179315436	PRKRA;PRKRA;PRKRA;MIR548N;PRKRA;DFNB59	0.0206071	0.605245763129105	0.0918608400549888
**cg09080114**	12	6983111	SPSB2;SPSB2	-0.0375272	-0.373047299396232	0.0918608400549888
**cg21032668**	1	245943224	SMYD3;SMYD3	-0.0312002	-0.0498377255162588	0.0918608400549888
**cg03486485**	11	14927004		0.0612674	1.37184395339927	0.0918608400549888
**cg16064811**	6	74005296	C6orf147	-0.025696	-0.0396212631694459	0.0918608400549888
**cg18386131**	11	67797936	NDUFS8	0.00343984	0.346872364597646	0.0918717256394305
**cg22799212**	1	161102325	DEDD;DEDD;DEDD	0.0190665	0.601808176513581	0.0919051715660417
**cg12439006**	11	119461149		-0.0522978	-0.0941390084946636	0.0919051715660417
**cg20988759**	4	24660033		-0.0510781	-0.117400145766645	0.0919051715660417
**cg19145138**	4	177120416		-0.0469503	-0.0815165852016932	0.0919051715660417
**cg18259930**	12	52215034	FIGNL2	0.0103599	0.344503092285716	0.0919051715660417
**cg02928991**	17	46687865	HOXB7	0.0340671	0.539090930897989	0.0919051715660417
**cg13446772**	12	64784173	C12orf56;C12orf56	0.0127817	0.360525169496888	0.0919051715660417
**cg04856858**	2	236402015	AGAP1;AGAP1	0.0257335	0.519840918174162	0.0919051715660417
**cg20426946**	8	104511776	RIMS2	0.0458305	0.272978967981853	0.0919051715660417
**cg23639922**	10	103892660	PPRC1	0.00664457	0.435357826518875	0.0919051715660417
**cg09736968**	2	233351609	ECEL1	0.0475284	0.642251069188734	0.0919051715660417
**cg27425193**	7	75931932	HSPB1;HSPB1	0.0202375	0.593389541472446	0.0919051715660417
**cg08915922**	1	42127381	HIVEP3;HIVEP3	0.0138547	0.559805744502013	0.0919051715660417
**cg18590738**	15	75230567	COX5A	0.00729771	0.488268853430539	0.0919051715660417
**cg04402633**	11	2907113	CDKN1C;CDKN1C;CDKN1C	0.0602326	0.959938624835844	0.0919051715660417
**cg08524474**	10	49813208	ARHGAP22	0.0568087	0.422764561600732	0.0919804894826352
**cg04976850**	1	86905492	CLCA2	-0.0817477	-0.170794785512028	0.0920122695992419
**cg24213508**	19	11040153	YIPF2;C19orf52	0.0343241	0.778681174773757	0.0920858221615516
**cg01602588**	11	57479678	MED19;TMX2;TMX2;TMX2	0.00545827	0.477753507740163	0.0921349927669038
**cg25074426**	14	89017776	PTPN21	0.0204415	0.346901535629845	0.0921349927669038
**cg05732832**	4	4868723		0.0177367	0.651227958490705	0.0921349927669038
**cg23672395**	20	1099079	PSMF1;PSMF1	0.00276376	0.338252407627953	0.0922343303057888
**cg22918432**	16	31471293	ARMC5;ARMC5	0.00673449	0.405671663813254	0.0922343303057888
**cg00667789**	1	59042065	TACSTD2	0.0379946	0.542652986796104	0.0922393375841617
**cg07449447**	14	55228808	SAMD4A;SAMD4A;SAMD4A	-0.0205985	-0.0315609707588156	0.0922767024226861
**cg05886574**	10	27530091	ACBD5;ACBD5;ACBD5	0.0045778	0.465703519335837	0.0922767024226861
**cg21421408**	1	108743240	SLC25A24	0.00299058	0.259274850743402	0.0923928757377145
**cg03217587**	2	239403003		0.0225093	0.622656344780195	0.0924008491230643
**cg08659621**	20	48552817	RNF114	0.00984279	0.523811265285948	0.0924008491230643
**cg15956369**	10	33946232		-0.0691553	-0.128163041781643	0.0924008491230643
**cg13557301**	12	110838588	ANAPC7;ANAPC7	-0.0315828	-0.049436659852585	0.0924008491230643
**cg12294585**	7	100472739	SRRT;SRRT;SRRT;SRRT;SRRT;SRRT;SRRT;SRRT	0.0670203	1.20807758148244	0.0924008491230643
**cg19340909**	1	1821422	GNB1	0.02553	0.92865497521141	0.0924008491230643
**cg19326876**	15	26107658	ATP10A	0.0136112	0.428974462669196	0.0924008491230643
**cg14236413**	2	71787501	DYSF;DYSF;DYSF;DYSF;DYSF;DYSF;DYSF;DYSF;DYSF;DYSF;DYSF;DYSF;DYSF;DYS	-0.0289038	-0.0456541128827712	0.0924008491230643
**cg08092393**	14	64970675	ZBTB25;ZBTB1;ZBTB1	0.0111466	0.496685926261276	0.0924008491230643
**cg15077994**	1	48462558	TRABD2B;TRABD2B	0.013432	0.462701201546237	0.0924008491230643
**cg25389187**	4	153355136	FBXW7;FBXW7	-0.0657927	-0.123302512816864	0.0924008491230643
**cg17293641**	8	119964144	TNFRSF11B;TNFRSF11B	0.0109305	0.716501476372142	0.0924008491230643
**cg26669159**	1	6967037	CAMTA1	0.0389149	0.166592942702134	0.0924397750045276
**cg01627046**	12	125473657	DHX37;DHX37	0.00362045	0.311463150177672	0.0924802388314265
**cg02055615**	6	7108063	RREB1;RREB1;RREB1;RREB1	0.0201137	0.584583105684427	0.0924802388314265
**cg10066305**	10	99447085	AVPI1	0.00678683	0.531762533901303	0.0924802388314265
**cg12471388**	10	98135209	TLL2	0.0267153	0.462081513549126	0.0924802388314265
**cg18042229**	2	219922729	IHH	0.0192608	0.693365377359856	0.0924802388314265
**cg24773676**	11	69633313	FGF3	0.0133268	0.423600385914557	0.0924802388314265
**cg00549040**	2	218048915		-0.0679558	-0.189039407377571	0.0924802388314265
**cg14799209**	7	106809543	HBP1;HBP1	0.0270053	0.377397017366489	0.0924802388314265
**cg09411922**	10	75121857		-0.10366	-0.212699447180038	0.0924802388314265
**cg00101895**	6	108279135	SEC63	0.0270837	0.891237433107822	0.0924802388314265
**cg23528708**	6	13814945	CCDC90A	0.00723843	0.38620845285461	0.0925631196818223
**cg10137463**	15	23085908	NIPA1;NIPA1	0.0173765	0.607168856013904	0.0925631196818223
**cg19174603**	10	123861564	TACC2;TACC2;TACC2;TACC2	-0.00481095	-0.00706929012913472	0.0925677525402793
**cg24011076**	16	2768597	PRSS27	-0.0127672	-0.0193484876902973	0.092609838735336
**cg23024421**	1	116961061	ATP1A1-AS1;ATP1A1-AS1;ATP1A1-AS1;ATP1A1-AS1;ATP1A1-AS1	0.00495812	0.444819273402438	0.0926340227492942
**cg16634936**	10	35033892	PARD3;PARD3;PARD3;PARD3;PARD3;PARD3;PARD3;PARD3;PARD3;PARD3;PARD3	-0.0650906	-0.113144646188939	0.0926340341278344
**cg06858294**	17	7983203	ALOX12B	0.0301985	0.834011244638958	0.092714281170691
**cg11861935**	14	92916462	SLC24A4;SLC24A4;SLC24A4	0.00768724	0.0115491180660211	0.092714281170691
**cg26674967**	3	88198835	C3orf38	0.00943566	0.338069031103821	0.092714281170691
**cg16747061**	3	38178884	MYD88;MYD88;MYD88;MYD88;MYD88;ACAA1;ACAA1;ACAA1	0.0210418	0.52844311011895	0.092714281170691
**cg07156296**	4	108911347	HADH	0.0140224	0.373002193820578	0.092714281170691
**cg14832223**	15	75490372		-0.0539688	-0.102563850914318	0.092714281170691
**cg19198794**	2	74757221	HTRA2;AUP1;HTRA2	0.009077	0.60598820693728	0.092714281170691
**cg22247327**	19	36546048	THAP8;WDR62;WDR62	0.0154042	0.62057661056518	0.092714281170691
**cg17379749**	12	119416855		-0.0537327	-0.0942204548029227	0.092714281170691
**cg16653538**	12	100967609	GAS2L3	0.0123138	0.540602963584345	0.092714281170691
**cg05021638**	8	21647026	GFRA2;GFRA2;GFRA2	0.0379088	0.977402350348405	0.092714281170691
**cg06118587**	11	10655869	MRVI1;MRVI1;MRVI1;MRVI1;MRVI1;MRVI1	-0.0137261	-0.0204813120092028	0.092714281170691
**cg00470768**	15	41285147	INO80	-0.107444	-0.239691027380074	0.092714281170691

**cg21969990**	20	36852920	KIAA1755	-0.0815806	-0.170322989219672	0.092714281170691
**cg07199894**	12	132379104	ULK1	0.0611781	0.282034947934652	0.092714281170691
**cg14732136**	17	66031828	KPNA2	0.0208485	1.15848928380308	0.092714281170691
**cg23185402**	16	47495113	PHKB;PHKB;ITFG1	0.00639299	0.550825023683847	0.092714281170691
**cg21677767**	7	138227378	TRIM24;TRIM24	-0.0495865	-0.0869567648289732	0.092714281170691
**cg21229103**	9	140738145		-0.0274885	-0.0425576881100855	0.092714281170691
**cg06616408**	5	92926504	NR2F1;MIR548AO	-0.0583091	-0.167597544957636	0.092714281170691
**cg25406779**	19	40732712	CNTD2	0.0191783	0.310527859795983	0.092714281170691
**cg12158389**	1	38230244	EPHA10;EPHA10	0.0251118	0.531770136120337	0.092714281170691
**cg09813341**	12	122665500	LRRC43	-0.109237	-0.221052576309218	0.092714281170691
**cg19749898**	11	1712765	HCCA2	-0.0261742	-0.0411433976926592	0.092714281170691
**cg08782481**	6	27839916	HIST1H3I	0.0672372	0.46765271345902	0.092714281170691
**cg09932345**	1	1710561	NADK	0.0156647	0.920641035327018	0.092714281170691
**cg23473088**	12	54529138		-0.0133363	-0.0199357123476128	0.092714281170691
**cg11877270**	2	65658583	SPRED2	0.0352277	1.19762395540624	0.092714281170691
**cg14561881**	17	62503076	CCDC45;DDX5	0.0125157	0.525847482423603	0.0928474024784932
**cg05744193**	2	201828499	ORC2;ORC2	0.0222355	0.852093842548394	0.0928562390610881
**cg22956310**	2	25142878	ADCY3	0.0109171	0.428381518877687	0.0928595676565089
**cg07320742**	7	27844237	TAX1BP1;TAX1BP1;TAX1BP1;TAX1BP1	-0.0621897	-0.117278634412371	0.0928595676565089
**cg02140403**	20	17711766	BANF2;BANF2;BANF2	-0.0272418	-0.0423037217599701	0.0928595676565089
**cg21267291**	20	17549945	DSTN;DSTN;BFSP1	0.0153579	0.370886281969472	0.0928595676565089
**cg26034219**	15	59887797		-0.0259782	-0.0466184068517873	0.0928595676565089
**cg06948384**	14	102504676	DYNC1H1	-0.0280954	-0.0431952639091703	0.0928595676565089
**cg15793624**	1	201476495	CSRP1;CSRP1;CSRP1	0.0371184	1.42378490240184	0.0928595676565089
**cg24088279**	16	69155473	CHTF8;CHTF8;CHTF8	-0.042511	-0.0709978896267638	0.0928595676565089
**cg03399170**	20	19719632		-0.0356959	-0.0627116430060074	0.0928595676565089
**cg15798823**	7	22866267		-0.0344405	-0.0564363851738888	0.0928595676565089
**cg11342046**	2	242641489	ING5	0.0158946	0.603734784046696	0.092912792216469
**cg10643407**	5	131892523	RAD50;RAD50	0.00968752	0.317755167463574	0.092912792216469
**cg15572423**	6	30307205	TRIM39;TRIM39	-0.0879835	-0.158265640623321	0.092912792216469
**cg05349650**	16	20753199	THUMPD1	0.0169613	0.760684859907776	0.092912792216469
**cg24108888**	6	32849992		-0.0850822	-0.174571496457207	0.092912792216469
**cg13710842**	5	72251735	FCHO2;FCHO2	0.0203479	0.928696584108214	0.092912792216469
**cg02727444**	19	12175631	ZNF844;ZNF844	0.0244625	0.655864133452548	0.092912792216469
**cg05479174**	12	132239000	SFRS8	-0.0149635	-0.0224258488777245	0.092912792216469
**cg11328704**	5	108063900		0.0074796	0.460678561404129	0.092912792216469
**cg13650683**	14	44010797		-0.0719523	-0.13241010388866	0.092912792216469
**cg23154002**	22	31741741	PATZ1;PATZ1;PATZ1;PATZ1;PATZ1;PATZ1;PATZ1;PATZ1	0.00417673	0.474092750681754	0.0929196388773307
**cg02655287**	4	1006115	FGFRL1;FGFRL1;FGFRL1	0.0167927	0.520439172793071	0.0929273445128552
**cg01316323**	22	26946742	TPST2;TPST2	-0.0287168	-0.0461091498589716	0.0929385374382797
**cg18011274**	17	43213270	ACBD4;ACBD4;ACBD4;ACBD4;ACBD4;ACBD4;ACBD4;ACBD4;ACBD4	0.0107513	0.440599589502567	0.0930855909060635
**cg16324018**	2	85580627	ELMOD3;ELMOD3;ELMOD3;RETSAT;ELMOD3	-0.0230978	-0.0357062866685709	0.0930951112393189
**cg18473033**	17	47653564	NXPH3;NXPH3	0.0307732	0.983208595167649	0.0931034652900766
**cg21251646**	6	31772441	2,00 LSM	-0.0511745	-0.119448880487942	0.0932137136222129
**cg11216682**	2	131113867	PTPN18;PTPN18	0.0438463	0.377010810093872	0.0932460828570672
**cg16458021**	21	43430507	ZNF295;ZNF295;ZNF295	0.0144788	0.824416971062606	0.0933407762620055
**cg13991635**	5	131133339	FNIP1;FNIP1	0.00942578	0.394800661286533	0.0933407762620055
**cg03630344**	1	7831156	VAMP3	0.00337721	0.267688188861655	0.0933407762620055
**cg08177439**	3	186284992	TBCCD1;TBCCD1;TBCCD1	0.0265939	1.11028443237073	0.0933407762620055
**cg00145284**	1	58852326		-0.0710099	-0.116976229698956	0.0933407762620055
**cg13406896**	2	10220712	CYS1	0.0301403	0.463221488095486	0.0933407762620055
**cg14229774**	1	179198798	ABL2;ABL2;ABL2;ABL2;ABL2;ABL2;ABL2;ABL2;ABL2;ABL2	0.00912094	0.390466945830393	0.0933407762620055
**cg17613375**	17	55162322	AKAP1	0.0217997	0.722263495203747	0.0933407762620055
**cg17960516**	4	3465004	DOK7;DOK7	0.0149192	0.435320458425441	0.0933407762620055
**cg09771049**	17	66031798	KPNA2	0.0396895	0.972549910105994	0.0933407762620055
**cg06468068**	2	147479064		-0.0227966	-0.0369602844600166	0.0933407762620055
**cg06741536**	12	69005242	RAP1B;RAP1B	0.0102886	0.432154447731189	0.0933407762620055
**cg00459937**	8	144699788	TSTA3	0.00696429	0.401597629444575	0.0933407762620055
**cg08486060**	5	180471692	MIR8089;BTNL9;BTNL9	-0.0249334	-0.0430156492200306	0.0933407762620055
**cg19358590**	6	158654166		0.0071684	0.395390065507757	0.0933407762620055
**cg26425064**	1	185290981		-0.0305835	-0.048924352931069	0.0933407762620055
**cg17237320**	3	10053000	LOC401052	0.0250155	0.434060515374166	0.0933407762620055
**cg26290391**	8	57672069		0.00532672	0.00799606846261881	0.0933407762620055
**cg09870092**	9	80938588	PSAT1;PSAT1	-0.046599	-0.0767168699346119	0.0933495251333379
**cg16240572**	3	134204857	CEP63;CEP63;ANAPC13;CEP63;ANAPC13;CEP63;ANAPC13;CEP63;ANAPC13;CEP63	0.00753514	0.561476500402097	0.0934607790344545
**cg14375282**	17	25981923		0.00887243	0.465012428036427	0.0935000085517567
**cg26963271**	1	66259081	PDE4B;PDE4B	0.00729294	0.449798586278492	0.0935815529244265
**cg18362448**	19	18544419	SSBP4;SSBP4	0.0516041	0.210878722657714	0.0935815529244265
**cg06775669**	7	101582572	CUX1;CUX1;CUX1;CUX1;CUX1;CUX1;CUX1	-0.0512868	-0.0822977586286271	0.0935815529244265
**cg10348193**	7	150020240	LRRC61;ACTR3C;LRRC61	0.0267235	0.40884905254101	0.0935935084180323
**cg16523805**	16	31076912	ZNF668;ZNF668;ZNF668;ZNF668	-0.0451869	-0.0823445685386629	0.0935935084180323
**cg21843902**	17	73847414	WBP2	0.00393767	0.00581366443742289	0.0935935084180323
**cg15975865**	2	127413831	GYPC;GYPC;GYPC;GYPC	0.00332133	0.243668113147245	0.0935935084180323
**cg21705104**	2	75144561	LINC01291	-0.0121308	-0.0182284182553724	0.0935935084180323
**cg01072217**	7	1275483	UNCX	0.0331815	0.949574226388947	0.0935935084180323
**cg02274500**	22	31064092	DUSP18	0.0105036	0.560766296494127	0.0935935084180323
**cg12980000**	3	191035318	UTS2D	-0.0127957	-0.0194254144316437	0.0935935084180323
**cg04147657**	18	47007789	C18orf32;C18orf32;RPL17-C18orf32;RPL17-C18orf32	-0.0332563	-0.0525194694301382	0.0935935084180323
**cg14723664**	6	34164073		0.0161936	0.73987912998244	0.0935935084180323
**cg04811762**	6	150039439	LATS1	0.0127232	0.435168171000494	0.0935935084180323
**cg12331743**	10	104153933	NFKB2;NFKB2;NFKB2	0.0158576	0.432220611494856	0.0936659888660074
**cg01506492**	2	219232590	C2orf62	0.011285	0.382524722291908	0.09368062038421

**cg20290336**	1	246741712	CNST;CNST	-0.0782623	-0.153433848671639	0.0936922022277785
**cg00292540**	3	149251606	WWTR1;WWTR1;WWTR1	-0.0117547	-0.0173547968343873	0.0936922022277785
**cg05329982**	11	62359210	TUT1	0.0165437	0.57681019016434	0.0936922022277785
**cg02144030**	14	102621015	WDR20;WDR20;WDR20;WDR20	-0.035076	-0.056539901700789	0.0936922022277785
**cg01222135**	7	99010594	BUD31	-0.0164425	-0.0252418646483893	0.0936922022277785
**cg24237600**	1	155178849	THBS3;THBS3;THBS3;THBS3;MTX1;MTX1	0.0392864	0.464524995706045	0.0936922022277785
**cg10926507**	1	224407167	LOC101927164	-0.0450468	-0.0794409925701158	0.0936922022277785
**cg16082007**	3	134093380	AMOTL2;AMOTL2	0.0248852	0.787371935644761	0.0936922022277785
**cg01836622**	6	107436914	BEND3	0.0129066	0.475169229537537	0.0936922022277785
**cg18530512**	19	14186152		0.0166834	0.596643155599338	0.0936922022277785
**ch.7.1699328F**	7	77226715	PTPN12;PTPN12;PTPN12	0.016854	0.361822476105755	0.0936922022277785
**cg21812670**	1	76251636	SNORD45C;RABGGTB	0.0810732	0.41732609132788	0.0936922022277785
**cg15154199**	17	1389305	MYO1C;MYO1C;MYO1C	0.00999463	0.502183114326954	0.0937154674571163
**cg22079616**	13	21141624	IFT88;IFT88	0.0116426	0.563732581009753	0.0937154674571163
**cg05470609**	9	33524217	ANKRD18B	0.00621779	0.499521937049244	0.0937154674571163
**cg06091566**	1	860621	SAMD11	0.048446	0.728828706244425	0.0937154674571163
**cg22887498**	19	39401301		-0.0324033	-0.0501880742243129	0.0937154674571163
**cg25324936**	17	45398817		-0.0236015	-0.0368226151431451	0.0937371446599504
**cg10474712**	22	42062693		0.0173419	0.469032929048555	0.093782568506474
**cg10546727**	5	1468074	LPCAT1	-0.00673373	-0.00999318028600355	0.093782568506474
**cg14497940**	17	77815166		0.0180417	0.605064789273188	0.0937854834640701
**cg15709375**	8	101716015	PABPC1	-0.0527378	-0.093715074714518	0.0937931491829104
**cg24095889**	15	91537917	PRC1;PRC1;PRC1	0.00858007	0.355008721444768	0.0938245393154144
**cg26442078**	5	180103241		-0.0568988	-0.118230040743497	0.0938245393154144
**cg06979118**	11	70601971	SHANK2	0.0636801	0.690600270301866	0.0938245393154144
**cg15204419**	9	102861339	INVS;INVS;INVS;ERP44	0.0686493	0.815542168705653	0.0938245393154144
**cg24948990**	8	143820828		0.00615562	0.441269561966243	0.0938245393154144
**cg03311606**	2	173292277	ITGA6;ITGA6	0.00967349	0.509929072324887	0.0938245393154144
**cg17819017**	17	79485996		0.0191082	0.935114016204138	0.0938245393154144
**cg20203136**	8	19674775	INTS10	0.027623	0.917389447210473	0.0938245393154144
**cg00599393**	8	22457479	C8orf58	0.00802717	0.374140558780785	0.0938245393154144
**cg17687970**	8	22462467	KIAA1967;KIAA1967	0.0592288	0.72257832858449	0.0938245393154144
**cg11071926**	1	116380316	NHLH2;NHLH2	0.0124717	0.315882002217289	0.0938245393154144
**cg05024927**	8	131370414	ASAP1	0.00632274	0.381487046076016	0.0938245393154144
**cg23247704**	1	116518985	SLC22A15	0.0146253	0.537060984528791	0.0938245393154144
**cg03392679**	3	71773917	EIF4E3;EIF4E3;EIF4E3;EIF4E3	0.0203745	0.853916391117162	0.0938245393154144
**cg14242042**	12	24715250	SOX5	0.0201447	1.21944804686018	0.0938245393154144
**cg10000977**	11	76690719	ACER3;ACER3;ACER3;ACER3	-0.0109149	-0.0161900282149658	0.0938245393154144
**cg07934449**	14	99946007	SETD3;SETD3	-0.0286417	-0.461505193953397	0.0938245393154144
**cg23297891**	8	103754172		-0.00622309	-0.00911730072423587	0.0938245393154144
**cg12071328**	11	20690930	NELL1;NELL1	0.0400018	0.860576823741696	0.0938245393154144
**cg01235203**	3	187461998	BCL6	0.0191329	0.582674960192126	0.0938245393154144
**cg15094636**	3	125094397	ZNF148	0.00624656	0.386618840360423	0.0938245393154144
**cg20011794**	22	37447915	KCTD17	0.020581	0.499882998538193	0.0938245393154144
**cg09709592**	16	2580833	AMDHD2;CEMP1	-0.0619294	-0.120864478052415	0.0938245393154144
**cg13752043**	9	131644109	LRRC8A;CCBL1;CCBL1;LRRC8A;CCBL1;LRRC8A	0.0151625	0.728854072902014	0.0938245393154144
**cg01373473**	7	105925859	NAMPT	0.00874075	0.503490831413298	0.0938245393154144
**cg15857656**	11	8403076		-0.00861205	-0.0128958476125835	0.0938245393154144
**cg08348100**	12	122261828	SETD1B	0.0128416	0.0199705273270756	0.0938245393154144
**cg08268045**	1	33815838	LOC101929464;PHC2;PHC2	0.00603801	0.357698734790287	0.0938245393154144
**cg11393511**	17	18086267	ALKBH5	0.00550917	0.512395415165334	0.0938245393154144
**cg15771432**	7	157477308	PTPRN2;PTPRN2;PTPRN2	0.0534519	0.871661041934456	0.0938245393154144
**cg21298511**	1	201476053	CSRP1;CSRP1;CSRP1	0.0340293	1.06134426349696	0.0938245393154144
**cg26708940**	2	102003884	CREG2	0.0164979	0.419690726553154	0.0938245393154144
**cg06526921**	16	2034455	GFER	0.0337351	1.10009828744102	0.0938245393154144
**cg05570682**	8	42132866	IKBKB	-0.0446518	-0.0740003343357633	0.0938245393154144
**cg10896623**	1	19923370	C1orf151	0.00629534	0.487996104723262	0.0938245393154144
**cg16724248**	3	55148638		-0.0372073	-0.0620943351943631	0.0938245393154144
**cg24168329**	8	25087909	DOCK5	-0.0117548	-0.0178507873737449	0.0938245393154144
**cg04588436**	2	60698937	BCL11A;BCL11A;BCL11A	-0.0152342	-0.0240741206600147	0.0938245393154144
**cg15423212**	2	71115225		0.017174	0.211430198812953	0.0938245393154144
**cg27306581**	1	161247619	PCP4L1	0.00850822	0.0127525344657126	0.0938245393154144
**cg26160196**	6	18264854	DEK;DEK	0.030703	0.502160286073046	0.0938245393154144
**cg00196414**	2	176947764	EVX2	0.0217293	0.559768911920585	0.0938245393154144
**cg17275207**	10	64895683	NRBF2	-0.0479626	-0.0834397730809916	0.0938245393154144
**cg14789642**	12	99149866	ANKS1B;ANKS1B;ANKS1B;ANKS1B;ANKS1B;ANKS1B;ANKS1B;ANKS1B;ANKS1B;ANKS	-0.0605712	-0.104284814971154	0.0938315214335526
**cg10276302**	7	134896411	WDR91	0.0129248	0.469953850561294	0.0938315214335526
**cg03948207**	1	114302014	PHTF1	0.0172662	0.456880641266184	0.0938315214335526
**cg00008103**	2	202009322	CFLAR;CFLAR;CFLAR;CFLAR;CFLAR;CFLAR;CFLAR;CFLAR;CFLAR-AS1	-0.0420327	-0.0693820179567702	0.0938315214335526
**cg03806646**	11	76176384	C11orf30;C11orf30;C11orf30;C11orf30	-0.06399	-0.11198275217885	0.0938315214335526
**cg03332122**	20	34203707	SPAG4	0.0162654	0.573110994288992	0.0938315214335526
**cg06270249**	3	55809537	ERC2	-0.033978	-0.052979942170411	0.0938315214335526
**cg15958576**	3	169380078	MECOM	0.0153872	0.402694683649508	0.0938602613813336
**cg08865636**	13	41267283		-0.0759575	-0.203399945115821	0.0938732500363726
**cg08941873**	5	118308850	DTWD2	-0.0832565	-0.164194849750304	0.0939091387810824
**cg22812697**	15	40401205	BMF	0.0115188	0.55571728936208	0.0939125435070345
**cg10683565**	3	79378041	ROBO1	-0.0602791	-0.112268898958801	0.0939125435070345
**cg01462545**	4	42401242	SHISA3	0.0338024	0.407433328068257	0.0939477504156055
**cg05782717**	15	40624098	C15orf52	0.0108238	0.016722356378093	0.0940241444353209
**cg27463953**	20	3451306	ATRN;ATRN	0.0327418	1.07828521550403	0.0940373072641609
**cg02674126**	19	40336878	FBL	0.013111	0.513900181129833	0.0940654953724335
**cg14125010**	7	67503827		-0.0176649	-0.0269716499694963	0.0941319734882328

**cg06177407**	11	65382100	MAP3K11	0.00986881	0.596911549565297	0.0941319734882328
**cg14398946**	9	132258775		0.0242788	0.616805589536861	0.0941319734882328
**cg07095995**	4	110223980	COL25A1;COL25A1	0.00642541	0.246813525389689	0.0941319734882328
**cg08153345**	12	2862084		0.0140692	0.676189203591728	0.0941319734882328
**cg06840699**	14	23058112	DAD1;DAD1	-0.00168486	-0.25211547433813	0.0941319734882328
**cg21548772**	3	47422338	PTPN23	0.0103198	0.657361061017372	0.0941319734882328
**cg16218980**	15	93631991	RGMA;RGMA	0.021678	0.882344459667338	0.0941319734882328
**cg23853020**	9	134141052	FAM78A	-0.0740889	-0.146989034541215	0.0941319734882328
**cg03217460**	11	70117060	PPFIA1;PPFIA1	0.0109866	0.371872308848139	0.0941319734882328
**cg06557577**	12	56220139	DNAJC14	-0.0322449	-0.0505966883295371	0.0941319734882328
**cg07061692**	19	59010811	SLC27A5	0.0101816	0.606962955787493	0.0941319734882328
**cg04051690**	2	95825276	ZNF514	0.0126393	0.522706237308925	0.0941319734882328
**cg24275354**	2	240964415	NDUFA10	0.00876001	0.539664635093064	0.0941319734882328
**cg12856612**	22	42228819	SREBF2	0.0105632	0.584337760807839	0.0941319734882328
**cg01606127**	8	61588765		-0.0226692	-0.0349165667073932	0.0941742530850551
**cg11236850**	4	8443022	ACOX3;ACOX3	0.0173265	0.731677274250301	0.0941742530850551
**cg00533749**	1	9992130	LZIC	-0.0634347	-0.121933508905673	0.0941742530850551
**cg19642128**	8	26240703	BNIP3L	0.0310462	1.06773644847556	0.0941742530850551
**cg04023028**	11	60681612	TMEM109;TMEM109	0.0101589	0.700645049679181	0.0941742530850551
**cg11063256**	19	52511428	ZNF615;ZNF615;ZNF615;ZNF615	0.0143605	0.418515800747818	0.0941764782362578
**cg00332680**	10	74079303		0.0138279	0.661390057666328	0.0941764782362578
**cg05258261**	3	140770608	SPSB4	0.0276137	0.701572246027919	0.0941764782362578
**cg17373321**	8	21999250	REEP4;REEP4	0.0156194	0.624189990860204	0.0942029584494113
**cg01808603**	16	66408919	CDH5	-0.0472088	-0.0813903557864951	0.0942029584494113
**cg00503587**	20	18118441	PET117	0.0147785	0.973720535758548	0.0942029584494113
**cg23323137**	6	42532021	UBR2	0.00991221	0.511795829917619	0.0942029584494113
**cg23170588**	7	100161127	AGFG2	0.00697325	0.0103395773931544	0.0942029584494113
**cg11281907**	9	77948925		-0.0355748	-0.060683606416549	0.0942029584494113
**cg09588492**	16	25266553	ZKSCAN2	-0.0153155	-0.0243913558438142	0.0942029584494113
**cg12216772**	10	46164062	ANUBL1;ANUBL1	-0.0927574	-0.167293276308685	0.0942029584494113
**cg09662431**	10	73975914	ASCC1;C10orf104	0.0107943	0.385105024888405	0.0942029584494113
**cg24502682**	10	22634192	SPAG6;SPAG6;SPAG6;SPAG6	0.022571	0.340490258313517	0.0942029584494113
**cg02854490**	7	144108266		-0.0475194	-0.0785462169430961	0.0942029584494113
**cg00281153**	16	75682798	TERF2IP;KARS;KARS	0.0205808	0.250894205731629	0.0942029584494113
**cg02311181**	19	14180847		-0.0219994	-0.034079045933374	0.0942029584494113
**cg07344583**	15	89456631	MFGE8;MFGE8;MFGE8;MFGE8	0.0141302	0.800204815005168	0.0942029584494113
**cg16538178**	16	85647585	KIAA0182;KIAA0182	0.0237435	0.634619327428556	0.0942029584494113
**cg10009297**	17	34890195	MYO19;MYO19;MYO19;PIGW	0.0181645	0.663507843786797	0.0942029584494113
**cg14944395**	17	5389859	MIS12;MIS12;MIS12;MIS12;MIS12;MIS12;MIS12;DERL2;DERL2;DERL2;DERL2;DERL2	0.0366884	1.1668480450521	0.0942029584494113
**cg09950479**	7	80610724		-0.0679747	-0.132318091878035	0.0942029584494113
**cg10055227**	16	58768320	GOT2	0.0257212	0.600533289621663	0.0942029584494113
**cg03771121**	16	8962586	CARHSP1;CARHSP1	0.004641	0.306121829574271	0.0942029584494113
**cg11808885**	21	46438743		0.0130681	0.382354882255387	0.0942029584494113
**cg13722539**	11	64085131	PRDX5;PRDX5;TRMT112;PRDX5	0.00593804	0.40647328715405	0.0942029584494113
**cg21123913**	4	89513664	HERC3;HERC3	0.0280158	0.64374571361161	0.0942029584494113
**cg24689245**	11	75479679	DGAT2	0.00443615	0.334026815861528	0.0942029584494113
**cg03754180**	13	37494042	SMAD9;SMAD9	0.00777242	0.462376695262537	0.0942029584494113
**cg10265095**	19	51699773		-0.0164252	-0.0260921799165877	0.0942029584494113
**cg01879556**	21	43932043	SLC37A1	-0.0288269	-0.0474307687774293	0.0942029584494113
**cg16739583**	2	216476434	LINC00607	-0.0151915	-0.0243291701506648	0.0942029584494113
**cg17018976**	3	239036	CHL1;CHL1;CHL1	0.0554454	0.27734024278149	0.0942029584494113
**cg25991122**	5	179246250	SQSTM1;SQSTM1	0.0115493	0.372253613037197	0.0942029584494113
**cg14170756**	1	16563782	C1orf89	0.00342375	0.408866859540687	0.0942029584494113
**cg04372675**	8	107283146	OXR1	0.0185447	0.510015571407564	0.0942029584494113
**cg07062933**	8	134309859	NDRG1;NDRG1	0.0313029	0.713002855635746	0.0942029584494113
**cg10367730**	10	120938495	PRDX3;PRDX3	-0.0114261	-0.280327474319605	0.0942029584494113
**cg06627580**	7	6630016	C7orf26;C7orf26	0.0191523	0.278120662424037	0.0942029584494113
**cg18675644**	2	61634300	USP34	-0.0538897	-0.0871063811774781	0.0942089396365906
**cg13372487**	8	37685049	GPR124	0.00642548	0.00972792281931042	0.0942089396365906
**cg03871887**	1	182295580		-0.0152577	-0.023758936414277	0.0942512511864926
**cg07800627**	5	155916319	SGCD;SGCD;SGCD	-0.0835908	-0.17057147875694	0.0942512511864926
**cg05187833**	6	17706939	NUP153	0.022192	1.11683100296449	0.0942512511864926
**cg05300996**	3	119814693	GSK3B;GSK3B	0.00846922	0.318333299994434	0.0942512511864926
**cg12591645**	18	54684851	WDR7;WDR7	-0.0222383	-0.749216583581074	0.0942512511864926
**cg15074761**	4	80219922	LINC01088	-0.0190829	-0.0290722801205355	0.0942838722725252
**cg19682595**	22	30819402	SEC14L2;SEC14L2;SEC14L2	0.00806291	0.520163783782118	0.0943198048197702
**cg16617633**	10	95847325	PLCE1;PLCE1-AS2;PLCE1;PLCE1	-0.0340757	-0.0532376873642853	0.0943198048197702
**cg02867162**	16	1020843	LMF1	0.0145839	0.754035402941587	0.0943198048197702
**cg22084165**	12	92294605		-0.042225	-0.0662295780694162	0.0943198048197702
**cg10720192**	3	169492135	MYNN;MYNN;MYNN;MYNN;MYNN	-0.0106331	-0.016594996698521	0.0943198048197702
**cg14994056**	11	107992015	ACAT1	0.0194013	0.497183241224881	0.0943233788945205
**cg03406394**	15	60296138	FOXB1	0.0227473	0.527101254370138	0.0943302710328003
**cg12173535**	19	54694174	MBOAT7;MBOAT7;MBOAT7;MBOAT7;TSEN34;TSEN34;TSEN34	0.0440721	1.58746237183092	0.094381270124384
**cg10071602**	16	2028740	TBL3	-0.0524389	-0.105387007189227	0.0943928772789986
**cg16063617**	5	128430663	ISOC1	0.00690046	0.473897527209262	0.0943928772789986
**cg17360140**	4	128886135	C4orf29;MFSD8	0.0339961	1.24264520881165	0.0943928772789986
**cg27096951**	2	35324412		-0.0612221	-0.116612211949647	0.0943928772789986
**cg00641348**	12	72105415		-0.0672365	-0.114705436291188	0.0944156391226568
**cg08221175**	14	69444840	ACTN1;ACTN1;ACTN1	0.0136981	0.448663834397662	0.094466808484364
**cg03108257**	1	115125395	BCAS2	-0.00802168	-0.0119148247035711	0.094466808484364
**cg06744740**	14	56584485	PELI2	-0.0434321	-0.530433727489889	0.0945361333338419
**cg00204782**	22	19753364	TBX1;TBX1;TBX1	0.012929	0.429964953333622	0.0945456998672238

**cg09230996**	10	102498419		0.0172295	0.35198347587174	0.0945456998672238
**cg14112555**	6	135799233	AHI1;AHI1;AHI1;AHI1	-0.0593355	-0.106320605844585	0.0945470383707454
**cg24337786**	19	39108672	MAP4K1;EIF3K;MAP4K1	0.00848789	0.60389455276105	0.0945470383707454
**cg06109608**	7	148725758	PDIA4;PDIA4	0.0106805	0.604062516440381	0.0945470383707454
**cg16931877**	3	32726605	CNOT10	0.038075	0.288833515265243	0.0945470383707454
**cg24257740**	3	79067775	ROBO1;ROBO1;ROBO1;ROBO1;ROBO1	0.00496913	0.295454030748769	0.0945666176831024
**cg03115547**	19	32836416	ZNF507;ZNF507	0.0134108	0.621847505107191	0.0945666176831024
**cg19557518**	17	60704641	MRC2	0.0223761	0.841470503230181	0.0945666176831024
**cg23599455**	22	46409460		0.0120827	0.644667416053761	0.0945666176831024
**cg12670845**	19	12780457	WDR83OS;WDR83OS;WDR83;WDR83;WDR83	0.00195334	0.252345631413429	0.0945666176831024
**cg09032310**	5	141262801		0.0117416	0.387563594484108	0.0945900058693774
**cg22024692**	17	80978417	B3GNTL1	-0.0203176	-0.0318279722251797	0.0945900058693774
**cg14165528**	1	209539810		-0.0517037	-0.0836689288239381	0.0945900058693774
**cg20798677**	17	39968547	FKBP10;SC65	0.037531	0.291725576009693	0.0947303431458132
**cg00923306**	18	3230737		-0.0340238	-0.0572145265857838	0.0947977641288323
**cg26968809**	17	79895355	PYCR1;PYCR1;PYCR1;PYCR1;PYCR1	0.0251285	0.526733680126426	0.0947977641288323
**cg01335738**	19	14019786	CC2D1A	-0.0460789	-0.0854620132723834	0.0947977641288323
**cg03953500**	11	86383478	ME3;ME3;ME3;ME3	0.00910308	0.498924446875469	0.0947977641288323
**cg13795627**	1	157015873	ARHGEF11;ARHGEF11	0.0402288	0.496531340907556	0.0947977641288323
**cg05918193**	19	1556731	MEX3D	-0.0250896	-0.0388541016754008	0.0948242317977083
**cg25640822**	5	134871645	NEUROG1	0.0209995	0.849963898999481	0.0948242317977083
**cg23599640**	2	127864941	BIN1;BIN1;BIN1;BIN1;BIN1;BIN1;BIN1;BIN1;BIN1;BIN1	0.0155108	0.477034635155183	0.0948242317977083
**cg04451615**	9	100991528	MIR6854;TBC1D2;TBC1D2;TBC1D2	-0.028346	-0.0434167058826763	0.0948242317977083
**cg00566164**	1	228559156	OBSCN	-0.0282145	-0.0454023299433766	0.0948242317977083
**cg26506507**	8	23261738	LOXL2	0.0166457	0.425404859860009	0.0948242317977083
**cg08594547**	4	178426921		-0.0185154	-0.0280228032025033	0.0948242317977083
**cg08525989**	20	32262282	NECAB3;NECAB3	0.0185568	0.459617855348096	0.0948242317977083
**cg26473253**	4	71768226	MOB1B;MOB1B;MOB1B;MOB1B;MOB1B;MOB1B	0.00839449	0.647358362643367	0.0948242317977083
**cg25849265**	3	47205643	SETD2	0.0114366	0.66543479910009	0.0948242317977083
**cg11349556**	18	7229672	LRRC30	-0.0499434	-0.108583529064577	0.0948242317977083
**cg02987163**	17	35849291	DUSP14	0.011069	0.314278741302811	0.0948242317977083
**cg07367563**	1	151032068	CDC42SE1;CDC42SE1;CDC42SE1;MLLT11;CDC42SE1	0.0164377	0.677263688546155	0.0948242317977083
**cg24648337**	7	645588	PRKAR1B;PRKAR1B;PRKAR1B;PRKAR1B;PRKAR1B;PRKAR1B	-0.00820284	-0.0121183969207652	0.0948242317977083
**cg26857141**	3	127005632		-0.0376233	-0.0646500682505825	0.0948242317977083
**cg19436929**	3	149703360		-0.0101487	-0.0151594287367157	0.0948242317977083
**cg27160460**	11	66617512	PC;PC;PC	0.0067943	0.0103301976641584	0.0948242317977083
**cg00959238**	19	40749359	AKT2;AKT2;AKT2	-0.0144182	-0.0216077131829228	0.0948242317977083
**cg08990514**	17	48672483	CACNA1G;CACNA1G;CACNA1G;CACNA1G;CACNA1G;CACNA1G;CACNA1G;CACNA1	-0.0321238	-0.055910397434211	0.0948242317977083
**cg12703194**	16	3086867	CCDC64B	0.0308526	0.410866677982351	0.0948242317977083
**cg11314818**	3	128444915	RAB7A	0.00507422	0.373462888076042	0.0948242317977083
**cg11226148**	2	27485298	SLC30A3	0.0177241	0.390313684239813	0.0948242317977083
**cg25183744**	1	150552404	MCL1;MCL1;MCL1	0.0108785	0.523402534631463	0.0948242317977083
**cg17643699**	12	27091484	FGFR1OP2;C12orf11;FGFR1OP2	0.0258901	0.679822892035785	0.0948242317977083
**cg25872752**	19	920370	KISS1R	0.0189442	0.597048800295871	0.0948242317977083
**cg16986746**	3	48594219	PFKFB4;PFKFB4	0.0144232	0.687744076782965	0.0948242317977083
**cg24218995**	18	67873134	RTTN	0.0275502	0.835520507369783	0.0948242317977083
**cg24950082**	16	30772857	C16orf93;RNF40;RNF40;RNF40;RNF40;C16orf93	0.0187155	0.494988114344808	0.0948272254139414
**cg21202700**	3	9799836	CAMK1;OGG1;OGG1;OGG1;OGG1;OGG1	-0.0734474	-0.173083138031517	0.0948272254139414
**cg24198848**	16	16033611		-0.0458803	-0.0740637283419783	0.0948272254139414
**cg12953445**	16	87056872		-0.043891	-0.0715123986000373	0.0948272254139414
**cg24751928**	20	55964459		0.0341133	0.537706820464356	0.0948272254139414
**cg17266780**	9	793023		0.00610891	0.00905705094216689	0.0948272254139414
**cg08114180**	16	54319624	IRX3	0.0362529	0.502185623322346	0.0948272254139414
**cg13290673**	2	159860200	TANC1;TANC1	-0.0109571	-0.016277026967845	0.0948272254139414
**cg27134659**	14	94640653	PPP4R4;PPP4R4;PPP4R4;PPP4R4	0.0238828	0.578320530648353	0.0948272254139414
**cg24907233**	22	46545351	PPARA;PPARA	0.0119992	0.519163644545767	0.0948272254139414
**cg19172750**	17	66287499	ARSG;SLC16A6	0.0115911	0.335467869303889	0.0948272254139414
**cg19336909**	12	69438873		-0.0334008	-0.0522807063280163	0.0948272254139414
**cg13384409**	7	2414466	EIF3B;EIF3B	0.00823772	0.0125018339295638	0.0948272254139414
**cg09061216**	1	1558936	MIB2;MIB2;MIB2;MIB2;MIB2;MIB2	-0.0149595	-0.0224669166656809	0.0948272254139414
**cg08529882**	10	124902456		0.0176121	0.302652243629615	0.0948272254139414
**cg13046107**	9	6757544	KDM4C;KDM4C;KDM4C;KDM4C;KDM4C;KDM4C;KDM4C	0.0204845	0.740561773777761	0.0948272254139414
**ch.7.1628533R**	7	73938938	GTF2IRD1;GTF2IRD1	0.0259484	0.412992001841926	0.0948272254139414
**cg12186917**	21	35013231	CRYZL1	-0.057182	-0.105144610925613	0.0948272254139414
**cg03305840**	8	143482468	TSNARE1	-0.0258773	-0.0398883689041576	0.0948272254139414
**cg06390411**	17	66031805	KPNA2	0.0166886	0.756332730764225	0.0948272254139414
**cg14557714**	9	90341385	CTSL1;CTSL1	0.00438116	0.330686439081364	0.0948272254139414
**cg16692004**	6	149639107	TAB2;TAB2;TAB2;TAB2	0.0403602	0.323994200651012	0.0948272254139414
**cg22825944**	16	68003289	SLC12A4;SLC12A4;SLC12A4	0.00415349	0.457865722416109	0.0948272254139414
**cg03859893**	10	60145150	TFAM	0.0121183	0.564798516051481	0.0948423492185707
**cg17510320**	11	66360559	CCS;CCDC87	0.0411664	0.280874998706766	0.0948423492185707
**cg16682175**	10	70091200	PBLD;PBLD;HNRNPH3;HNRNPH3	0.00968959	0.479982073840663	0.0948423492185707
**cg02222496**	17	10597147	SCO1	-0.0394633	-0.0627613110579855	0.0948423492185707
**cg04134803**	5	150080579	RBM22;RBM22	0.0113686	0.489746926389863	0.0948423492185707
**cg14534987**	2	27341893	CGREF1;CGREF1;CGREF1;CGREF1;CGREF1	0.0640426	0.582858436453073	0.0948707794030221
**cg24202119**	5	35938692	CAPSL;CAPSL;CAPSL	-0.0309756	-0.0594372526222155	0.0949194343865873
**cg01989866**	20	31017450	ASXL1	0.00396587	0.00592998100496294	0.0949470036671092
**cg04052337**	9	136399924	ADAMTSL2;ADAMTSL2	0.00354746	0.275116839513687	0.0949470036671092
**cg13306061**	14	96555594	C14orf132;C14orf132;C14orf132;C14orf132	0.00609375	0.00910298282046019	0.0949470036671092
**cg25203425**	2	121572108	GLI2	-0.0377175	-0.0671198019517124	0.0949470036671092
**cg05504606**	19	1039871	ABCA7	0.0133814	0.574172983461649	0.0949470036671092
**cg06810179**	16	90038862	CENPBD1;AFG3L1;AFG3L1;CENPBD1;AFG3L1	0.011256	0.466055713573436	0.0949470036671092

**cg22792161**	9	5841794		0.0634121	0.245944220912892	0.0949470036671092
**cg12883204**	12	75723847	CAPS2	0.0103389	0.262285495640009	0.0949470036671092
**cg01464969**	4	1768943		0.0581668	0.87343841870624	0.0949470036671092
**cg24209738**	6	16303169	ATXN1;ATXN1	-0.0129183	-0.0195763040587279	0.0949470036671092
**cg23541365**	11	85974795	EED;EED	-0.0342685	-0.0539023146566355	0.0949470036671092
**cg17717259**	7	29846259		0.0141186	0.510338232231248	0.0949470036671092
**cg04007241**	1	179198680	ABL2;ABL2;ABL2;ABL2;ABL2;ABL2;ABL2;ABL2;ABL2;ABL2	0.0194359	0.628106003804106	0.0949470036671092
**cg17336172**	3	49377548	USP4;USP4	0.00852018	0.555524899769679	0.0949470036671092
**cg24222746**	2	148779348	ORC4L;ORC4L;ORC4L	0.0181852	0.520562624423264	0.0949470036671092
**cg07319961**	6	166401460	LINC00602;LINC00473;LINC00473	0.00614991	0.470783804021804	0.0949642750210622
**cg04281845**	10	10215481		-0.0726683	-0.131007666754283	0.0949642750210622
**cg25338843**	17	42441302	FAM171A2	0.0924663	0.545036046624263	0.0949642750210622
**cg21274724**	17	6386543	PITPNM3;PITPNM3	-0.0785747	-0.167035957826367	0.0949642750210622
**cg23576504**	17	7298067	PLSCR3;PLSCR3;PLSCR3;PLSCR3;TMEM256-PLSCR3	0.00624285	0.3509294271467	0.0949642750210622
**cg24567495**	15	45814535	SLC30A4;HMGN2P46	0.0221754	0.73354706676775	0.0949642750210622
**cg20577878**	16	66914781	PDP2	0.015606	0.730674540061282	0.0949642750210622
**cg16677488**	16	57038246		-0.0417253	-0.0748978693722565	0.0949642750210622
**cg08908586**	14	92414185	FBLN5	0.00764207	0.664469420990118	0.0949642750210622
**cg23471890**	12	111051874	TCTN1;TCTN1;TCTN1	0.00870847	0.365344008057624	0.0949674748246165
**cg13273243**	4	169405039		-0.0443949	-0.0710609636739234	0.0949674748246165
**cg05116896**	16	30366237	CD2BP2;CD2BP2	0.00803049	0.508298262240006	0.0949674748246165
**cg20739360**	7	91833683	KRIT1;KRIT1;KRIT1;KRIT1;KRIT1	-0.0466591	-0.0868749940245667	0.0949674748246165
**cg10573190**	2	85843190	USP39	0.00964721	0.478980402921342	0.0949674748246165
**cg17944110**	1	10856657	CASZ1;CASZ1;CASZ1;CASZ1	0.0120608	0.569650129375637	0.0949674748246165
**cg25119654**	11	67085309	LOC100130987	0.0176685	0.640569269431914	0.0949674748246165
**cg11089232**	9	68998480		-0.0746314	-0.201233507684642	0.094983354778471
**cg02896403**	1	155231989	SCAMP3;SCAMP3;SCAMP3;SCAMP3	0.00761706	0.404510978838306	0.0950002499029479
**cg14587604**	5	158758756	IL12B	0.0140118	0.877758166052167	0.0950002499029479
**cg27498669**	20	32262017	NECAB3;NECAB3	0.0128418	0.372549655648997	0.0950183277978001
**cg13842154**	14	103390247	AMN	0.0140281	0.274379554933786	0.0950183277978001
**cg05222995**	12	115124973		0.0206373	0.441197789913517	0.0950183277978001
**cg19419789**	16	2255584	MLST8;MLST8;MLST8;MLST8	0.0224176	0.605138661041434	0.0950183277978001
**cg24500832**	12	54394212	HOXC9	0.00573856	0.324836758574496	0.0950183277978001
**cg11686792**	13	114876061	RASA3	0.0162138	0.511300095785298	0.0950183277978001
**cg18354686**	16	4897378	UBN1;GLYR1	0.00916053	0.57715763720472	0.0950183277978001
**cg03927520**	2	220110237	GLB1L;GLB1L;STK16;STK16;STK16	0.0540082	0.745502235712453	0.0950183277978001
**cg02820958**	19	7099040		0.0697714	0.459316459515737	0.0950183277978001
**cg05166499**	8	133493008	KCNQ3	0.0128095	0.496927830046857	0.0950183277978001
**cg18789663**	1	242688591	PLD5	0.029233	0.809807533071022	0.0950183277978001
**cg09182900**	6	43909955		0.00727403	0.0109334333622453	0.0950183277978001
**cg17986684**	1	228674167	RNF187	0.0150433	0.290832481298557	0.0950183277978001
**cg09864858**	1	65613311	AK3L1;AK3L1;AK3L1;AK3L1	0.0174386	0.479960030838375	0.0950183277978001
**cg13102235**	1	236203522	NID1	-0.0266619	-0.0417829697916978	0.0950183277978001
**cg18278714**	2	127752644		-0.00660664	-0.00970186618252459	0.0950183277978001
**cg04936382**	20	36662003	RPRD1B;KIAA0406;RPRD1B	0.0249972	0.785771558067763	0.0950183277978001
**cg05020775**	20	1246934	SNPH	0.0407951	0.415214038392403	0.0950183277978001
**cg06655143**	8	144499638	MAFA-AS1	0.00391207	0.00587806564145406	0.0950183277978001
**cg04965365**	9	108487768	TMEM38B	-0.0259682	-0.0411943298570594	0.0950183277978001
**cg19100138**	3	183993995	ECE2;ECE2;ECE2;ECE2	0.0244181	0.565017722600119	0.0950183277978001
**cg06089712**	14	35343862	BAZ1A;BAZ1A	0.00722129	0.535603631946742	0.0950183277978001
**cg25523538**	10	28031957	MKX	0.0135634	0.583674321364433	0.0950183277978001
**cg16450103**	17	41174539	VAT1	0.00724396	0.509182948065978	0.0950183277978001
**cg23288521**	20	37075451	SNORA39;SNHG11	0.0120747	0.473618319610704	0.0950183277978001
**cg08888178**	19	344981	MIER2	0.025388	0.500033963484174	0.0950183277978001
**cg23084309**	19	6280164	MLLT1	0.0437262	0.230172647588392	0.0950183277978001
**cg07004372**	3	57386380	DNAH12	-0.073586	-0.144809265875581	0.0950183277978001
**cg21803245**	14	105331124	KIAA0284;KIAA0284	0.0429869	0.584711597462825	0.0950183277978001
**cg08478189**	2	208030666	KLF7	0.00750164	0.258726884242325	0.0950183277978001
**cg24448859**	4	174255105	HMGB2;HMGB2;HMGB2	0.0270651	0.746810692531146	0.0950183277978001
**cg18249149**	2	128785194	SAP130;SAP130	0.0188855	0.678629488577855	0.0950183277978001
**cg25447359**	22	30790057		-0.0593296	-0.122078590908167	0.0950183277978001
**cg08497901**	1	45472676	HECTD3;HECTD3	0.00971126	0.0146375802386213	0.0950183277978001
**cg17843125**	1	100598159	SASS6;CCDC76	0.0207402	0.300258639827096	0.0950183277978001
**cg12187069**	19	41082589	SHKBP1	0.0121276	0.699173559030092	0.0950183277978001
**cg01686044**	19	45754802	MARK4	0.0165834	0.446544660620868	0.0950183277978001
**cg01803835**	8	55223410		-0.0400185	-0.0663343372284033	0.0950183277978001
**cg19273746**	6	71666063	B3GAT2	0.0540787	0.635764475675962	0.0950183277978001
**cg24577940**	12	106469246	NUAK1	-0.0341586	-0.0550813073449949	0.0950183277978001
**cg23097985**	8	22552961		0.0140304	0.917819978531452	0.0950183277978001
**cg19001554**	5	31527598	DROSHA;DROSHA	-0.0568429	-0.0973390471692885	0.0950183277978001
**cg15739955**	19	14183651	LOC113230	0.00577313	0.46600260879792	0.0950183277978001
**cg15203270**	4	77636913	SHROOM3	-0.0441187	-0.0737089880154956	0.0950183277978001
**cg00099976**	13	26625273	SHISA2	0.0389396	0.309784525365142	0.0950183277978001
**cg00973947**	3	143692255	C3orf58;C3orf58;C3orf58	0.0298823	0.652663719298241	0.0950183277978001
**cg11555640**	21	40754783	WRB	-0.0353531	-0.0569141832607637	0.0950183277978001
**cg06770993**	3	49158462	USP19	0.0267783	0.903084099480991	0.0950183277978001
**cg00492003**	15	78572760	DNAJA4;DNAJA4;DNAJA4	-0.0233572	-0.036440437142432	0.0950183277978001
**cg08089031**	6	139456240	HECA	0.0237958	0.713534819186147	0.0950183277978001
**cg26036478**	4	124319962	SPRY1;SPRY1	0.0104229	0.540126318265438	0.0950183277978001
**cg15100599**	1	223538174	SUSD4;SUSD4	0.0327904	0.745757689870319	0.0950183277978001
**cg26711517**	10	131909267	LINC00959	0.00694865	0.531961115838112	0.0950183277978001
**cg17452291**	7	86781755	DMTF1;DMTF1;DMTF1;DMTF1;DMTF1;DMTF1;DMTF1	0.0207001	0.527666394543655	0.0950183277978001

**cg02126603**	17	79455239		0.0154997	0.764420167931768	0.0950183277978001
**cg25037274**	7	29872894	WIPF3	-0.06794	-0.124335836481196	0.0950183277978001
**cg05237820**	20	39945987		0.00802232	0.294485277225959	0.0950183277978001
**cg25512587**	9	136344026	SLC2A6;SLC2A6	0.0112377	0.323281442868116	0.0950183277978001
**cg13388111**	11	61062665	VWCE;VWCE	0.0184524	0.835958500283704	0.0950183277978001
**cg04099264**	1	44435507	DPH2;DPH2	0.00841446	0.434452002648708	0.0950183277978001
**cg12095968**	1	151739679	OAZ3;OAZ3	-0.0391164	-0.064995320273144	0.0950183277978001
**cg27219662**	6	28891728	TRIM27;TRIM27	0.0318771	0.689797571559281	0.0950183277978001
**cg22818988**	3	3168509	TRNT1	0.00385849	0.404722973152917	0.0950183277978001
**cg16459120**	4	81189164	FGF5;FGF5;FGF5	-0.0338725	-0.277321305236348	0.0950183277978001
**cg03654430**	10	91174411	IFIT5;IFIT5	0.010713	0.524534110157572	0.0950183277978001
**cg01037895**	1	21877781	ALPL;ALPL	-0.0442948	-0.0796879318459848	0.0950183277978001
**cg22377913**	1	26758610	DHDDS;DHDDS	0.00640594	0.376578766004739	0.0950183277978001
**cg10141938**	7	26240087	HNRNPA2B1;HNRNPA2B1;CBX3;CBX3	0.00667488	0.439397503208481	0.0950183277978001
**cg00663077**	4	188916709	ZFP42	0.0500775	0.663035156306368	0.0950183277978001
**cg06688733**	5	42813842		-0.0162398	-0.0246463583096094	0.0950183277978001
**cg10241347**	10	124639260	LOC399815;FAM24B	0.0830722	1.405495849072	0.0950183277978001
**cg17526727**	6	43791674		-0.0605544	-0.121211502253473	0.0950183277978001
**cg16589580**	22	30239152		-0.0153895	-0.0233049648258374	0.0950183277978001
**cg16004435**	8	38749768		-0.0134957	-0.0203274325350824	0.0950183277978001
**cg00902415**	17	41174425	VAT1;VAT1	0.00926275	0.341965644153144	0.0950183277978001
**cg20628663**	10	43360327		0.035463	0.144774719333567	0.0950183277978001
**cg27324859**	19	17398922		-0.00970893	-0.0144041023725973	0.0950183277978001
**cg20567721**	16	81040639	CENPN;CENPN;CENPN;CENPN;CENPN;CENPN;C16orf61	0.0114704	0.710295547241124	0.0950183277978001
**cg03414474**	2	105488932		0.00985504	0.525228188208789	0.0950183277978001
**cg26797585**	19	3585609	GIPC3	0.0226762	0.761096706008042	0.0950183277978001
**cg17144508**	2	128283727	IWS1	0.0269546	0.707846380157785	0.0950183277978001
**cg15082212**	12	124068903	TMED2	0.00585046	0.399717183690086	0.0950183277978001
**cg18273700**	3	47319063	KIF9;KIF9;KIF9	0.0117245	0.018087039440486	0.0950183277978001
**cg18390181**	10	102810139		0.00937131	0.399752570631649	0.0950183277978001
**cg14621103**	7	1178041	C7orf50;C7orf50;C7orf50	0.00948624	0.626506134317444	0.0950183277978001
**cg14279539**	20	49998378		-0.0742186	-0.170337219113569	0.0950183277978001
**cg05014952**	14	35873130	NFKBIA	0.0282713	0.789391039650904	0.0950183277978001
**cg15650240**	1	15736530	EFHD2	0.0366648	0.493830714469832	0.0950183277978001
**cg20936291**	9	130679328	ST6GALNAC4;ST6GALNAC4	0.0297882	0.36292254697159	0.0950183277978001
**cg07682083**	14	73957524	HEATR4;C14orf169	0.0103513	0.544890440515002	0.0950517699625633
**cg11562028**	5	150911153	FAT2	-0.0119373	-0.0188138192174902	0.095053273247236
**cg20947082**	1	233749643	KCNK1	0.0646044	1.06922808378946	0.095053273247236
**cg27607338**	3	119041955	CDGAP	0.0117852	0.407698481613085	0.095053273247236
**cg16232013**	11	63952725	STIP1;STIP1;STIP1	0.0270395	0.733115391410683	0.0950828803888212
**cg21975007**	6	132128965	ENPP1	0.030452	1.00361164875414	0.0951010438574133
**cg25975435**	8	127489179		-0.0249372	-0.0388817328662632	0.0951010438574133
**cg02505827**	5	68711277	MARVELD2;MARVELD2	0.0395177	0.534451884314287	0.0951010438574133
**cg20012885**	18	12377404	AFG3L2	0.0329553	0.298675965432123	0.0951010438574133
**cg10670553**	4	65264573	TECRL	-0.0422602	-0.0662903558030511	0.0951310105464896
**cg05762700**	4	18023779	LCORL;LCORL	0.0499882	1.04421006596846	0.0951310105464896
**cg00372297**	17	27139635	FAM222B;FAM222B;FAM222B;FAM222B;FAM222B;FAM222B;FAM222B;FAM222B;FAM2	0.0134664	0.5751808627967	0.0951310105464896
**cg07694872**	9	132048856		0.00799767	0.0120061079310287	0.0951310105464896
**cg14760438**	6	33409448	SYNGAP1	-0.0289456	-0.0461698096533926	0.0951310105464896
**cg21234853**	2	198318200	COQ10B	0.0103133	0.379953493928629	0.0951330787454335
**cg19700341**	17	72857076	GRIN2C	0.0476605	0.547578233081002	0.0951641667523737
**cg01287079**	6	108888941	FOXO3;FOXO3	-0.0269668	-0.0435773155072615	0.0951778856087981
**cg02878891**	2	102090444	RFX8	0.0123871	0.36245307315968	0.0951778856087981
**cg05100916**	7	112090586	IFRD1;IFRD1;IFRD1	0.0151507	0.466315141575755	0.0951778856087981
**cg08533424**	17	37009973	RPL23;SNORA21;RPL23	0.00614482	0.506850207627069	0.0951778856087981
**cg22868034**	19	49092375	SULT2B1;SULT2B1	0.0438337	0.55804454857265	0.0951778856087981
**cg08227526**	4	155663529		0.0112475	0.336221304261956	0.0951778856087981
**cg14550910**	10	54216510		-0.0625541	-0.11277199281924	0.0952824731679905
**cg25837984**	3	167372481	WDR49	-0.0342569	-0.0601901293358314	0.0953506022636338
**cg22610533**	12	64238379	SRGAP1	0.0242338	0.863346080971979	0.0953506022636338
**cg00590851**	13	27335089	GPR12	0.0232774	0.518686150858743	0.0953506022636338
**cg11896345**	17	1532217	SLC43A2	0.017078	0.778549824415653	0.0953506022636338
**cg23248351**	8	108511700	ANGPT1	-0.017291	-0.026154479950358	0.0953693924654778
**cg18035158**	6	107810971	SOBP	0.026732	0.599918642254283	0.0954048686304252
**cg11838475**	2	241556683	CAPN10	-0.0130246	-0.0197830201492203	0.0954048686304252
**cg14204211**	10	81107088	PPIF	0.0166125	0.597493241395508	0.0954048686304252
**cg00723044**	14	71276570	MAP3K9	0.00796615	0.621867458387948	0.0954048686304252
**cg10037754**	15	45563114	LOC101928414;SLC28A2	-0.0563736	-0.0949506552900264	0.0954048686304252
**cg04540836**	8	123854227	ZHX2	0.00751737	0.0111303332053754	0.0954048686304252
**cg24106894**	15	48623432	DUT;DUT;DUT	0.0143691	0.825248902682071	0.0954048686304252
**cg00362614**	1	28052155	FAM76A;FAM76A;FAM76A;FAM76A;FAM76A	0.0213832	0.983227341273231	0.0954048686304252
**cg11169461**	2	224904347	SERPINE2;SERPINE2;SERPINE2	0.00454145	0.359978878422546	0.0954145134085853
**cg04117643**	17	7476796	EIF4A1;SNORA48	0.0141004	0.309562812632341	0.0954252389144774
**cg05301181**	10	35379533	CUL2	0.00662931	0.328687961149588	0.0954252389144774
**cg03543731**	15	50474621	SLC27A2;SLC27A2;SLC27A2;SLC27A2	0.00689344	0.440913392900428	0.0954252389144774
**cg04944802**	1	228603769	TRIM17;TRIM17;TRIM17	0.0139105	0.462085050300827	0.0954252389144774
**cg03727373**	11	1404502		0.0458955	0.748695450106384	0.0954252389144774
**cg19968692**	13	73301900	C13orf37;C13orf34;C13orf37	0.0145	0.821056527644555	0.0954252389144774
**cg06464369**	5	1005292		0.0332049	0.614673197075693	0.0954252389144774
**cg01497487**	22	38141814	TRIOBP;TRIOBP;TRIOBP	0.025358	0.459930497381207	0.0954252389144774
**cg01023787**	5	1243714	SLC6A18	0.0140453	0.0222452356767433	0.0954252389144774
**cg09460159**	17	30771470	PSMD11	0.0116759	0.463721727981234	0.0954252389144774

**cg14132868**	11	18343680	HPS5;HPS5;HPS5;HPS5;HPS5;HPS5;GTF2H1;GTF2H1	0.0171097	0.740262389213655	0.0954252389144774
**cg16464704**	1	26249489		0.0217192	0.396916017267465	0.0954252389144774
**cg13372927**	11	111896293	DLAT	0.0314986	0.974915761165355	0.0954252389144774
**cg22706629**	15	65823539	PTPLAD1	0.0263998	0.596256320067036	0.0954252389144774
**cg08072480**	11	65292666	SCYL1;SCYL1	0.0141482	1.02176355528883	0.0954252389144774
**cg02414034**	17	5185283	RABEP1;RABEP1	0.0214545	0.759006769665077	0.0954252389144774
**cg00485681**	9	124990761	LHX6;LHX6;LHX6;LHX6	0.0118073	0.261838911599401	0.0954252389144774
**cg06737004**	16	31226760	TRIM72	0.0187964	0.647743846281717	0.0954252389144774
**cg13300098**	9	33036573	DNAJA1;DNAJA1	-0.0659726	-0.106318784645194	0.0954252389144774
**cg09039436**	11	46369515	DGKZ;DGKZ;DGKZ	0.0132786	0.582668572383042	0.0954252389144774
**cg14022434**	10	123687714	ATE1;ATE1	0.00824662	0.383503196550631	0.0954252389144774
**cg20673919**	11	125702313	PATE4	-0.0754426	-0.22755627829196	0.0954252389144774
**cg21869055**	19	51522454	KLK10;KLK10;KLK10	0.0170838	0.331000675978514	0.0954252389144774
**cg26884276**	5	139554806	C5orf32;C5orf32	0.020192	0.97711144159788	0.0954252389144774
**cg09980176**	19	11532175	CCDC151	0.0311419	0.586944867842283	0.0954252389144774
**cg11598954**	11	44086756	ACCS;ACCS	-0.0346361	-0.0550982440922402	0.0954252389144774
**cg00265925**	14	39938424		-0.0489933	-0.0898285931911581	0.0954252389144774
**cg22972965**	17	77772650		0.00817606	0.301950873439633	0.0954252389144774
**cg08388245**	3	50171724		-0.0241465	-0.0370383973144767	0.0954252389144774
**cg09816342**	1	42318574	HIVEP3;HIVEP3;HIVEP3;HIVEP3	-0.00730774	-0.0108871481752756	0.0954252389144774
**cg05040338**	16	89270802		-0.064138	-0.113735553173976	0.0954252389144774
**cg21429500**	4	17579272	LAP3	0.00747944	0.428293351734547	0.0954252389144774
**cg09933638**	3	128444901	RAB7A	0.00539827	0.321847509788029	0.0954257306869682
**cg17369438**	1	36184635	C1orf216;C1orf216	0.0234919	0.898380176175597	0.0954384599300855
**cg25004838**	6	32112189		0.0265924	0.0467893656402074	0.0954835620559887
**cg21324037**	12	132674606		0.00749712	0.640553266858248	0.0954835620559887
**cg08329655**	11	7534677	PPFIBP2	0.016044	0.68208411709638	0.0954835620559887
**cg04726800**	3	128598191	ACAD9	0.0116493	0.5438396597469	0.0954835620559887
**cg14292314**	19	28293235		-0.0266796	-0.0468639938748661	0.0954835620559887
**cg14672709**	17	48133110	ITGA3;ITGA3	0.00360314	0.303021881812395	0.0954864999993396
**cg00125159**	1	228290140	C1orf35	0.045642	1.5401932834872	0.0954864999993396
**cg18485839**	19	58378654		-0.034897	-0.0562771684994835	0.0954864999993396
**cg24592391**	11	102653026	WTAPP1	-0.0396363	-0.0635297680968842	0.0954864999993396
**cg07703976**	7	48494734	ABCA13	0.0449567	0.0689510570681153	0.0954864999993396
**cg19721541**	8	18027367	NAT1	-0.0555049	-0.106332436962695	0.0954864999993396
**cg18519694**	11	118307647	MLL	0.0227965	0.551234342963992	0.0954864999993396
**cg04446983**	6	20530262		-0.0568989	-0.0957968862024246	0.0954864999993396
**cg00680572**	14	37050691	NKX2-8	0.0354899	0.536724074624231	0.0954864999993396
**cg18633432**	3	135915556	MSL2	0.0169675	0.892626495703535	0.0954864999993396
**cg02257300**	19	45873380	ERCC2;ERCC2	0.00517108	0.337621414833329	0.0954864999993396
**cg02703787**	15	93256789		-0.0459311	-0.130760950579643	0.0954864999993396
**cg16666935**	13	31736119	HSPH1	0.0192633	0.415729586371867	0.0954864999993396
**cg00093058**	16	717932	RHOT2	0.0103345	0.572549714725399	0.0954864999993396
**cg10186461**	3	107656904		-0.012596	-0.019139856897852	0.0954864999993396
**cg06211164**	3	50359065	HYAL2;HYAL2	0.0125709	0.425111402801373	0.0954864999993396
**cg10143122**	4	147194482	SLC10A7;SLC10A7	0.0258913	0.0405788475119648	0.0954864999993396
**cg05968010**	1	210000883	C1orf107	0.0634474	0.38042865168091	0.0955110095310495
**cg03022556**	1	162335234	NOS1AP;NOS1AP;NOS1AP	0.00804234	0.0120358599947891	0.0955110095310495
**cg06918551**	6	166356928	LINC00473;LINC00473	-0.0223783	-0.034038259049572	0.0955110095310495
**cg25600562**	9	98271449	PTCH1;PTCH1;PTCH1;PTCH1;PTCH1	0.0131108	0.537395342086927	0.0955110095310495
**cg10008636**	14	103542242		0.0151105	0.518757731076409	0.0955110095310495
**cg08987887**	6	76311629	SENP6;SENP6;SENP6;SENP6;SENP6;SENP6	0.0104595	0.640835915802788	0.0955110095310495
**cg08224208**	19	15218165	SYDE1	0.0305838	0.481844026441054	0.0955110095310495
**cg19510699**	20	1297651	SDCBP2	-0.112209	-0.308657918140101	0.0955268073836805
**cg24141233**	1	212781895	ATF3;ATF3;ATF3	0.0314778	0.883153403814401	0.0955268073836805
**cg20873420**	2	208634917	FZD5	0.0159449	0.52845667862048	0.0955268073836805
**cg13563284**	20	21082335		0.0164185	0.566127619597529	0.0955504542920542
**cg21412928**	6	30640498	DHX16;DHX16	0.0408485	0.894855928357725	0.0955507338740581
**cg27344330**	19	17530716	MVB12A;MVB12A;MVB12A	0.0318293	1.18208391462404	0.0955606383551256
**cg27211204**	15	99791508	LRRC28	0.0176224	0.665147810350936	0.0956094217265247
**cg15355400**	16	24857426	SLC5A11;SLC5A11;SLC5A11;SLC5A11;SLC5A11	0.0147722	0.0233714848172026	0.0956160226674209
**cg03509991**	1	46050221	NASP;NASP;NASP	0.00524055	0.40919473830501	0.0956270677270513
**cg16437766**	4	39529126	UGDH;UGDH;UGDH-AS1;UGDH;UGDH;UGDH;UGDH;MIR1273H	0.00548007	0.395991808330334	0.0956270677270513
**cg26990633**	3	44665441	ZNF197;ZNF197;ZNF197-AS1	-0.0945084	-0.20450901850429	0.0957091619425686
**cg25776635**	12	122231757	RHOF	0.021034	0.487915517106921	0.0957165397259027
**cg09063944**	1	3688921	LOC388588	0.00607019	0.430092595733813	0.0957351107314399
**cg06421707**	20	25228305	PYGB	0.00825043	0.573735388724267	0.095742807938221
**cg10534439**	2	69969029	ANXA4	0.0432194	0.453192720797326	0.095742807938221
**cg03546183**	11	128584167	FLI1;FLI1;FLI1;FLI1	0.0301771	0.0495094084088655	0.095742807938221
**cg15744534**	15	70388889	TLE3;TLE3;TLE3;TLE3;TLE3;TLE3	0.0267982	0.358655794238352	0.0957580638353478
**cg25984249**	6	31509765	BAT1;BAT1;SNORD84;BAT1;BAT1	0.0371877	0.28068091504786	0.0957580638353478
**cg26163463**	5	139926773	EIF4EBP3;ANKHD1-EIF4EBP3	-0.0692191	-0.160486219524752	0.0957580638353478
**cg23724858**	6	32055711	TNXB	0.0137926	0.680263384691115	0.0957580638353478
**cg24603408**	8	22562397		0.0228119	0.267439356916834	0.0957580638353478
**cg20729301**	2	233368115		0.00617141	0.761208701325704	0.0957580638353478
**cg21895364**	7	150767428	SLC4A2;SLC4A2;SLC4A2;SLC4A2;SLC4A2;SLC4A2;SLC4A2;SLC4A2	0.0128756	0.0199000109725248	0.0957580638353478
**cg21710066**	6	474967		0.0234274	0.532497444640357	0.0957617831138547
**cg01356910**	16	4323571	TFAP4	0.0708597	1.10894434193932	0.0957617831138547
**cg26270411**	20	43514327	YWHAB;YWHAB	0.0150756	0.488463134883789	0.0957815322303836
**cg01644723**	8	146228246	ZNF252;C8orf77	0.00685306	0.58495902285718	0.0958062771130654
**cg26200974**	10	124383677	DMBT1;DMBT1;DMBT1	0.0196462	0.0313797983236131	0.0958082651067595
**cg24007917**	12	77272927	CSRP2	0.0117651	0.411667654370373	0.0958082651067595

**cg08254315**	8	25593585		-0.0399467	-0.0662505255741801	0.0958082651067595
**cg08646105**	13	32417270		-0.0704279	-0.129229752717816	0.0958180925850529
**cg02073511**	16	3550924	CLUAP1	0.0477755	0.359490972970146	0.0958180925850529
**cg19937493**	1	27722051	GPR3	0.00518868	0.375491119646639	0.0958180925850529
**cg09999876**	14	24521843	LRRC16B	0.007621	0.462553225252996	0.0958180925850529
**cg05112574**	17	76374542	PGS1	0.00868565	0.450874399323036	0.095825285715459
**cg11666559**	1	221067662		0.0130011	0.288387112892645	0.095825285715459
**cg16301728**	2	187717549		-0.0465092	-0.0958590061323111	0.095825285715459
**cg04328477**	16	68270258	ESRP2	0.0458061	0.758829853837869	0.095825285715459
**cg02688760**	1	182573731	RGS16	0.0165242	0.501892611741915	0.095825285715459
**cg05456948**	10	135171583	C10orf125;C10orf125	0.0334141	0.327428465806945	0.095825285715459
**cg02067722**	6	137144131	PEX7	0.00671954	0.496103372705634	0.095825285715459
**cg16501572**	11	71934660	INPPL1	0.0171507	0.951394214926675	0.095825285715459
**cg18942110**	15	91072797	CRTC3;CRTC3	0.0154534	0.475115773503833	0.095825285715459
**cg06869971**	15	69706519	KIF23;KIF23	0.0166184	0.558829379616014	0.095825285715459
**cg12821161**	11	65101842	DPF2	0.0126237	0.492802702285788	0.095825285715459
**cg17186539**	19	10628196	S1PR5;S1PR5	0.0268277	0.438249547481917	0.095825285715459
**cg22861561**	12	48397730	COL2A1;COL2A1	0.0386446	0.418509365845269	0.0958960330108476
**cg00755496**	3	52257183	TLR9	-0.00710135	-0.0104389179381919	0.0959205150229736
**cg25634545**	10	104154207	NFKB2;NFKB2;NFKB2	0.0073417	0.401946821450228	0.0959205150229736
**cg20048602**	1	1310743	AURKAIP1;AURKAIP1;AURKAIP1;AURKAIP1	0.0376998	0.419453989873664	0.0959205150229736
**cg17101358**	17	42422688	GRN;GRN	0.00507082	0.366501503782565	0.0959205150229736
**cg06404838**	11	117015391	PAFAH1B2	0.0118467	0.540470356235641	0.0959205150229736
**cg22674613**	6	44310331	SPATS1	0.0151697	0.483347792132847	0.0959205150229736
**cg00172020**	3	150264474	SERP1;EIF2A	0.0075823	0.364532259916093	0.0959205150229736
**cg22078572**	18	61033618	KDSR	0.00909237	0.400919724585409	0.0959205150229736
**cg00754426**	20	37100437	RALGAPB	-0.0440968	-0.169143528791704	0.0959461581571344
**cg00515575**	2	47168619	TTC7A;TTC7A;TTC7A;TTC7A;TTC7A;MCFD2;MCFD2;TTC7A;TTC7A	0.0345741	0.636008710522672	0.0959601268193507
**cg13675624**	12	6419136	PLEKHG6;PLEKHG6	-0.0431372	-0.084830603282001	0.0959601268193507
**cg04744597**	5	160976197	GABRB2;GABRB2	0.00395748	0.3527432711488	0.0959700551562448
**cg11594927**	6	29720600		0.0143807	0.510259203654395	0.0959700551562448
**cg03826535**	12	113659136	TPCN1;TPCN1;IQCD	0.0109294	0.671248625581166	0.0960488469819885
**cg27314741**	19	46144881	EML2-AS1;EML2-AS1;EML2;EML2;EML2;EML2	0.0204924	0.999836098512718	0.0960488469819885
**cg21845957**	14	103988428	CKB	0.0964629	0.936365775734163	0.0960488469819885
**cg04497885**	19	42747008	GSK3A	0.0124662	0.774179894235665	0.0960488469819885
**cg13863753**	8	38855115	ADAM9;TM2D2;ADAM9;ADAM9;TM2D2;TM2D2;ADAM9;TM2D2	0.0071638	0.24895360796396	0.0960488469819885
**cg10217975**	3	52091484	DUSP7	0.00676693	0.546804439663229	0.0960488469819885
**cg15017004**	18	2906290	EMILIN2	0.0227834	0.598451873682719	0.0960488469819885
**cg00022308**	2	27851831	CCDC121;CCDC121;GPN1;CCDC121;GPN1;GPN1;GPN1;CCDC121;GPN1	0.0149008	0.554967700173088	0.0960488469819885
**cg27470027**	11	63684601	RCOR2	0.0400417	0.222615652684209	0.0960488469819885
**cg02979923**	20	17207337	PCSK2	0.0276014	0.379725565446538	0.0961692096056076
**cg20801666**	22	20103296	RANBP1;TRMT2A;TRMT2A;TRMT2A;TRMT2A;TRMT2A;TRMT2A;MIR6816	0.0120083	0.0204190263329073	0.0961692096056076
**cg14057961**	14	65007327	HSPA2;HSPA2	0.0158858	0.54297304908356	0.0961692096056076
**cg07240937**	1	153650963	1,00 NPR	0.0122948	0.472899094218107	0.0962258183199315
**cg17416165**	5	99382140		-0.0366482	-0.0716551865621851	0.0962258183199315
**cg16852685**	4	154712145		0.0101339	0.270167756910475	0.0962868138220571
**cg12208612**	3	169490789	MYNN	0.00612676	0.432074329806372	0.0962868138220571
**cg06489364**	1	19578250	EMC1;EMC1;EMC1;EMC1;MRTO4;MRTO4	0.0432157	0.618588482562684	0.0963075897510351
**cg16124571**	1	115212670	DENND2C;DENND2C	0.0150701	0.500746068301	0.0963094788156769
**cg19910128**	1	1496936	SSU72	-0.0260168	-0.040060904498872	0.0963094788156769
**cg18645335**	19	17858607	FCHO1;FCHO1;FCHO1;FCHO1	0.017473	0.656126663422306	0.0963463878159874
**cg06704518**	8	11565530	GATA4	0.0116859	0.310171962311147	0.0963463878159874
**cg07357178**	8	49534582	LOC101929217;LOC101929268	-0.0409751	-0.0736128625775434	0.096379483368019
**cg17988623**	11	117025270	PAFAH1B2	-0.0111737	-0.0165392089209149	0.0963816770539127
**cg02852436**	20	2821472	FAM113A;VPS16;VPS16	0.0371954	0.899174949286332	0.0963857721353605
**cg10221240**	4	1004669	FGFRL1;FGFRL1	0.00704783	0.586297304299412	0.0963857721353605
**cg02957340**	14	100070899	CCDC85C	0.0110734	0.496886132865559	0.0964034430402662
**cg11110643**	11	72929135	P2RY2;P2RY2;P2RY2	0.026147	0.317326255008226	0.0964034430402662
**cg09423703**	2	71115230		0.0122531	0.506612334576577	0.0964034430402662
**cg12956230**	9	88556256	NAA35	0.0175839	1.21666927174733	0.0964034430402662
**cg13332172**	19	37178884	ZNF567	0.0205056	0.631792366364404	0.0964034430402662
**cg17271592**	1	149821902	HIST2H2AA4;HIST2H2AA3	0.0146247	0.565116117213023	0.0964034430402662
**cg26691953**	12	49110790	CCNT1	0.00813395	0.478124014135594	0.0964034430402662
**cg22882523**	8	145107012	OPLAH	0.018497	0.492165092140383	0.0964034430402662
**cg01076051**	5	130329564		-0.0561129	-0.0984665813190005	0.0964034430402662
**cg25181699**	11	300688	IFITM5	-0.0424064	-0.114053668533962	0.0964034430402662
**cg08229018**	4	119274133	PRSS12	0.0499507	0.551667056201455	0.0964034430402662
**cg22965663**	14	77787393	POMT2;GSTZ1;GSTZ1;GSTZ1;GSTZ1;GSTZ1	0.0260457	1.08643925505297	0.0964034430402662
**cg08621286**	7	1200209	LOC101927021;LOC101927021;LOC101927021;LOC101927021;LOC101927021;LOC101	0.00721278	0.426623672629618	0.0964034430402662
**cg09692733**	19	10249298	DNMT1;DNMT1;DNMT1;DNMT1	0.00618028	0.00925597718497841	0.0964034430402662
**cg27425511**	19	11039385	YIPF2;C19orf52	0.0184708	0.657003451796382	0.0964034430402662
**cg21988584**	14	31281516		-0.0567468	-0.0941938772778178	0.0964261777650068
**cg11154382**	17	48227323	PPP1R9B	0.00305286	0.280939598773599	0.0964261777650068
**cg15176005**	1	228122465	WNT9A	-0.0458682	-0.0743527520683661	0.0964398543063559
**cg22045228**	2	85581875	ELMOD3;ELMOD3;ELMOD3;ELMOD3;ELMOD3;ELMOD3;RETSAT;ELMOD3;ELMOD3	0.00578353	0.408850419353185	0.0964830393059253
**cg02693002**	10	126106594	OAT	0.0412379	0.252381523172033	0.0964830393059253
**cg09718640**	2	113522225	CKAP2L;CKAP2L	0.00955557	0.740525068397686	0.0964830393059253
**cg08376191**	3	160283074	KPNA4;KPNA4	0.0191003	0.81214081008018	0.0964830393059253
**cg13525064**	2	73114426	SPR	0.0145868	0.455410036538882	0.0964830393059253
**cg27467005**	19	17622279	PGLS	0.0394466	0.899376208540485	0.0964830393059253
**cg17429065**	1	6453910	ACOT7	0.0396877	0.581248763238148	0.0964830393059253
**cg22545535**	17	17495014	PEMT;PEMT;PEMT;PEMT;PEMT;PEMT	0.0243102	0.940719428201807	0.0964943169183351

**cg26378157**	16	89984203	MC1R	0.00647391	0.374298536557293	0.0965612810475103
**cg19331885**	11	119722305		-0.0370423	-0.0595987442263674	0.0966217334135386
**cg11686528**	17	1083002	ABR;ABR	0.0364663	0.699317794355933	0.0966217334135386
**cg17703880**	8	87110636	ATP6V0D2	-0.0296469	-0.0468666762466742	0.0966263586900543
**cg06430572**	21	46221894	UBE2G2;UBE2G2	0.0146731	0.422489140471635	0.0966263586900543
**cg05225390**	11	113644407	ZW10;ZW10	0.0113213	0.392578236732517	0.0966263586900543
**cg12434394**	18	10663708	LOC101927410	-0.0191919	-0.0297518178937672	0.0966700092915516
**cg14902146**	15	90294607	MESP1	0.011959	0.925899746626606	0.0966896620726204
**cg11718780**	1	200728684	CAMSAP1L1	-0.0673931	-0.130117481639868	0.0966896620726204
**cg14940233**	8	3429428	CSMD1	-0.0296755	-0.0502556625088459	0.0966896620726204
**cg07010075**	2	121199631		0.0187699	0.478928106958077	0.0966896620726204
**cg13699414**	5	178957880		0.0444495	0.872012088187862	0.0966896620726204
**cg10583683**	15	23034700	NIPA2;NIPA2;NIPA2;NIPA2	0.0470995	1.27529626075875	0.0966896620726204
**cg02692785**	11	84148821	DLG2;DLG2	0.0063024	0.312962068973882	0.0966896620726204
**cg01840575**	2	38977957	SFRS7	0.0547502	0.765802025391088	0.0966896620726204
**cg09378707**	11	3836728	PGAP2;PGAP2;PGAP2;PGAP2;PGAP2;PGAP2;PGAP2;PGAP2;PGAP2;PGAP2;PGAP2;P	-0.0183248	-0.0278936182695191	0.0966896620726204
**cg04067803**	2	56462511	CCDC85A	-0.0167454	-0.0256081702554842	0.0967040744029192
**cg16439512**	15	57598924	LOC283663	0.0379036	0.366967302127825	0.0967040744029192
**cg17100705**	21	45148737	PDXK	0.0163307	0.956065454596925	0.096714146717445
**cg08894790**	4	177241590	SPCS3	0.00995849	0.573090518361369	0.096714146717445
**cg20132549**	11	442189	ANO9	0.0164221	0.343467730267822	0.0967438331139809
**cg23637624**	8	124387949	ATAD2	-0.0233193	-0.0363454884403469	0.0967663247664824
**cg23422604**	22	43364847	PACSIN2	-0.0731072	-0.131799412132615	0.0967663247664824
**cg02465374**	6	166401700	LOC441177;C6orf176;C6orf176	0.00554467	0.376963986546219	0.0967663247664824
**cg14889079**	19	54606153	NDUFA3	0.0471317	0.350301353072845	0.0967663247664824
**cg09754991**	13	107220236	ARGLU1	0.00450809	0.330699353167246	0.0967663247664824
**cg26924044**	16	2920939		-0.0876869	-0.194240587063345	0.0967663247664824
**cg10835773**	6	19753491		-0.0619211	-0.129504430305123	0.0967663247664824
**cg26687499**	3	135914775	MSL2;MSL2	0.0326865	1.21691168106168	0.0967663247664824
**cg16131972**	20	45282877	SLC13A3	-0.0175236	-0.0265582447171671	0.0968329730628465
**cg09463047**	17	36104218	HNF1B;HNF1B	0.00445612	0.370583717909647	0.0968329730628465
**cg01477385**	14	103995350	TRMT61A	0.00400341	0.434777934487659	0.0968329730628465
**cg10820715**	1	214470024	SMYD2	-0.019688	-0.0306598899967127	0.0968329730628465
**cg18247177**	17	7745472	KDM6B	0.0176396	0.526744688236024	0.0968329730628465
**cg05846056**	1	59762542	FGGY;FGGY	0.00761501	0.518897776068765	0.0968329730628465
**cg16000824**	11	1143665		-0.0468603	-0.0808758339337879	0.0968329730628465
**cg12187687**	7	27253834		-0.103816	-0.241979346040982	0.0968468936374364
**cg18647237**	5	43017561		0.00663785	0.390238688353276	0.0969013265175139
**cg01679512**	13	114108652	ADPRHL1	0.0080569	0.012112355721076	0.0969013265175139
**cg19620922**	21	46824536	COL18A1	0.0331619	0.358320930544104	0.0969013265175139
**cg07158701**	17	40540601	STAT3;STAT3;STAT3	0.00951655	0.931916156829124	0.0969021271937684
**cg06725204**	7	129027898	AHCYL2;AHCYL2;AHCYL2;AHCYL2	-0.00670513	-0.00988737736095067	0.0969938011718534
**cg03476862**	7	98581334	TRRAP	-0.00604082	-0.00886511561224142	0.0969938011718534
**cg03331924**	1	208038413		-0.0478749	-0.0795610057498883	0.0969938011718534
**cg03462171**	16	1664488	CRAMP1L	0.0502273	0.255481877042465	0.0969938011718534
**cg04839422**	22	44287876	PNPLA5	0.00764434	0.415526506514623	0.0969938011718534
**cg05311119**	4	1805090	FGFR3;FGFR3;FGFR3	-0.0374826	-0.0597922855072129	0.0970113870034738
**cg04950743**	2	39002436		-0.0532376	-0.0903457839545397	0.0970816236032976
**cg20723436**	2	139428554	NXPH2	-0.0246452	-0.0401595241316243	0.0970816236032976
**cg07066932**	1	24285945	PNRC2	0.0123453	0.788042155373288	0.0970816236032976
**cg14355911**	17	59473456		0.03333	0.646397361798611	0.0970816236032976
**cg26454158**	19	12273814	ZNF136	0.00449625	0.304325635844268	0.0970816236032976
**cg22876918**	17	4458867	MYBBP1A;MYBBP1A	0.0151767	0.574025497679461	0.0970992549304238
**cg27638453**	6	157470150	ARID1B;ARID1B;ARID1B	0.00339607	0.0049677148761993	0.0970992549304238
**cg05871607**	1	28241317	RPA2	0.0263445	0.62933443820143	0.0970992549304238
**cg13521940**	21	36262239	RUNX1;RUNX1;RUNX1	0.012438	0.57410407496326	0.0970992549304238
**cg01526748**	3	191930926	FGF12;FGF12	0.028255	0.048688452789902	0.0970992549304238
**cg10300729**	10	65284527	REEP3	-0.0861046	-0.17231895045283	0.0971392044553465
**cg11087358**	12	57940980	DCTN2	0.0141974	0.350531988886453	0.0971392044553465
**cg11992317**	20	44520160	CTSA;CTSA;NEURL2;CTSA	0.00903153	0.601528846241619	0.0971392044553465
**cg17739868**	6	157751337		0.00968613	0.014379847670877	0.0971392044553465
**cg22437161**	7	155605081	SHH	0.0187452	0.546155843703273	0.0971392044553465
**cg11482099**	11	31833477	PAX6;PAX6;PAX6	0.0111266	0.51388708156276	0.0971392044553465
**cg24902504**	1	206858868	MAPKAPK2;MAPKAPK2	0.00833536	0.510739863814459	0.0971392044553465
**cg02126788**	1	116381660	NHLH2;NHLH2	0.0238042	0.599681261604773	0.0971392044553465
**cg00929855**	6	31783364	HSPA1A;HSPA1L;HSPA1A	0.0249482	1.07014863657023	0.0971392044553465
**cg04220579**	7	915756	C7orf20	0.00966809	0.53160622083214	0.0971392044553465
**cg09217023**	3	9394237		-0.0888773	-0.182712903200915	0.0971392044553465
**cg16259886**	2	32265121	DPY30	0.023834	0.730407870103392	0.0971392044553465
**cg18838941**	10	15376939	FAM171A1	-0.00790557	-0.011722759632983	0.0971392044553465
**cg23828635**	10	14642921	FAM107B	-0.0519412	-0.0862946265650665	0.0971392044553465
**cg13499067**	11	10879629	ZBED5;ZBED5	0.0298143	1.10958548156195	0.0971392044553465
**cg04378177**	9	100505109		-0.037812	-0.0614610123339234	0.0971392044553465
**cg10519154**	11	67212322	CORO1B;CORO1B	0.00688178	0.0104154422435993	0.0971392044553465
**cg10780164**	10	135149009	CALY	0.00986699	0.7599326023285	0.0971392044553465
**cg16808455**	18	12309071	TUBB6	0.0335702	0.471202649777177	0.0971392044553465
**cg24445840**	22	34292239	LARGE;LARGE	-0.0441299	-0.0800350956172378	0.0971392044553465
**cg18021265**	2	220462522	STK11IP	0.00371383	0.295003056280063	0.0971392044553465
**cg01600100**	12	96794307	CDK17;CDK17	0.0314165	0.289289314461773	0.0971392044553465
**cg27190946**	15	32639011		0.0451917	0.814387116064525	0.0971392044553465
**cg12380209**	10	120515094	C10orf46	0.00638668	0.647794232742701	0.0971392044553465
**cg07342403**	6	131081554		-0.0316058	-0.0501569644842207	0.0971392044553465

**cg11105718**	8	102586527	GRHL2	-0.0602998	-0.135937569260882	0.0971392044553465
**cg10017641**	1	203606297	ATP2B4;ATP2B4	-0.0621275	-0.105971695479026	0.0971392044553465
**cg02573276**	1	2344586	PEX10;PEX10	0.00551886	0.336077948294346	0.0971392044553465
**cg24145357**	7	92219539	FAM133B;FAM133B;FAM133B;FAM133DP;FAM133B	0.0438736	0.838960302166194	0.0971392044553465
**cg12442015**	1	2323763	MORN1;RER1	0.0123631	0.623748992237704	0.0971392044553465
**cg09602251**	10	25245759		-0.0539925	-0.101970205114379	0.0971392044553465
**cg18008202**	2	131100479	IMP4;CCDC115	0.0267309	0.696781157395487	0.0971392044553465
**cg16734281**	18	43684176	ATP5A1;ATP5A1;HAUS1;HAUS1	0.022626	0.815796722590457	0.0971392044553465
**cg16364833**	2	70121066	SNRNP27	0.0209897	0.617526693706062	0.0971392044553465
**cg26968387**	19	37569268	ZNF420	0.00423363	0.321685735541239	0.0971392044553465
**cg14310703**	3	126006903		0.0252508	0.468776899642106	0.0971392044553465
**cg01988459**	11	68622903		0.0172022	0.4808775670722	0.0971392044553465
**cg13360126**	14	52292059		-0.0404004	-0.0667018903293892	0.0971392044553465
**cg19196684**	1	53608037	SLC1A7	-0.023936	-0.0380090658574126	0.0971509674954701
**cg26263043**	3	39262442		-0.0359014	-0.253071167425549	0.0971509674954701
**cg03649606**	16	89501735	ANKRD11	-0.0519164	-0.102379878474101	0.0971509674954701
**cg22711340**	17	53418967		-0.0224925	-0.0353089265703771	0.0971509674954701
**cg06186663**	14	81687575	GTF2A1;GTF2A1;GTF2A1	0.0103743	0.448480523707902	0.0972403512064214
**cg11573637**	6	41755210	TOMM6;TOMM6	0.0169283	0.770875925398104	0.0972403512064214
**cg15554875**	2	45236686	SIX2	0.0180449	0.452445222854809	0.0972437984886374
**cg04025889**	20	32891089	AHCY;AHCY;AHCY	0.00637501	0.289129819090631	0.0972437984886374
**cg19510792**	5	16935695	MYO10	0.00694837	0.369609584489084	0.097256766073146
**cg00287831**	3	184081167	POLR2H	0.0115159	0.561239927697744	0.0972598314084044
**cg22629515**	1	243712954	AKT3;AKT3	-0.039824	-0.0639994463941562	0.0972896471212305
**cg04613990**	3	49096748	QRICH1;QRICH1	-0.077406	-0.147815344329603	0.097298752294498
**cg25801746**	8	9046480		-0.0399974	-0.0647784621095468	0.097298752294498
**cg16930880**	2	177373410		-0.0606477	-0.118095198652939	0.0972999594285597
**cg01727346**	22	45596797	C22orf9;C22orf9	-0.0814043	-0.16963319115713	0.0972999594285597
**cg26560222**	1	50888826	DMRTA2;DMRTA2	0.017569	0.395404028367604	0.0972999594285597
**cg08588104**	6	15494473	JARID2;JARID2	-0.0343435	-0.0530985739996355	0.0972999594285597
**cg12442376**	1	38869276		-0.0470784	-0.0793600924102757	0.0972999594285597
**cg11826116**	22	39916314	ATF4;ATF4	0.0233832	0.888434102196265	0.0972999594285597
**cg22114242**	12	110376950	GIT2;GIT2;GIT2;GIT2;GIT2;GIT2;GIT2;GIT2;GIT2;GIT2	0.00692602	0.0103354112833521	0.0972999594285597
**cg22183049**	5	139143972		0.0144089	0.488572649898027	0.0972999594285597
**cg09210331**	6	7108204	RREB1;RREB1;RREB1;RREB1;RREB1;RREB1;RREB1	0.0225634	0.756451778648312	0.0972999594285597
**cg10092374**	17	79988647	RAC3;LRRC45	0.00983194	0.614521730566776	0.0972999594285597
**cg07837102**	6	32940879	BRD2;BRD2	0.0133592	0.365080583536294	0.0972999594285597
**cg14576258**	9	88513789		-0.0325975	-0.0573747955321205	0.0972999594285597
**cg13246328**	12	28179213		-0.0510545	-0.104480460173782	0.0974642850658026
**cg21647170**	7	56119546	CCT6A;PSPH;CCT6A	0.0195124	0.380311112285465	0.0974642850658026
**cg23709617**	10	135030554	KNDC1	0.00694653	0.0102687140218129	0.0974642850658026
**cg17967429**	10	90711500	ACTA2;ACTA2	0.0117439	0.0175165635168293	0.0974642850658026
**cg00934997**	14	73957439	C14orf169;HEATR4	0.02606	0.214553349562155	0.0974642850658026
**cg10216074**	2	25467197	DNMT3A;DNMT3A;DNMT3A;DNMT3A;DNMT3A;DNMT3A	0.015398	0.0250343076139353	0.0974642850658026
**cg24608684**	6	130686865		0.0348688	0.829719382602233	0.0974642850658026
**cg10494282**	1	44440945	ATP6V0B;ATP6V0B	0.0388412	0.48558540637926	0.0974687993320465
**cg21813478**	20	2645987	IDH3B;IDH3B;IDH3B	-0.0491717	-0.0805122075556799	0.0974687993320465
**cg13870080**	11	19303281		-0.102048	-0.221473789001999	0.0974687993320465
**cg26902735**	15	64679755	TRIP4;KIAA0101	0.00398919	0.388162660631123	0.0974687993320465
**cg24931017**	20	59600990		0.0059908	0.00880940469373674	0.0974687993320465
**cg12467096**	11	71936303	INPPL1	0.0123696	0.590196784046286	0.0974687993320465
**cg17552958**	2	74734862	PCGF1	0.0101858	0.45435413148551	0.0974687993320465
**cg10822418**	8	61430157	RAB2A	0.0180252	0.478887899384585	0.0974687993320465
**cg14856684**	14	65439245	RAB15	0.00809957	0.410080254561945	0.0974687993320465
**cg00658427**	14	102431823	DYNC1H1	0.0078433	0.285882088475482	0.0974687993320465
**cg24926008**	4	140374789	RAB33B	0.0151742	0.928015763566676	0.0974687993320465
**cg17846194**	12	49485900	DHH	0.0105995	0.313353783583756	0.0974687993320465
**cg00993710**	7	150722703	ATG9B	-0.0529409	-0.0945819554191462	0.0974687993320465
**cg04832767**	7	96632841	DLX6AS	0.00922232	0.370408303178588	0.0974687993320465
**cg23965429**	14	99740998		0.0384759	0.501704637958639	0.0974687993320465
**cg01188574**	19	925914	ARID3A	0.0165574	0.583658325042038	0.097487839112932
**cg03598754**	17	26711803	SARM1	0.0605669	0.660936716020076	0.0974966086427733
**cg26756119**	2	71836673	DYSF;DYSF;DYSF;DYSF;DYSF;DYSF;DYSF;DYSF;DYSF;DYSF;DYSF;DYSF;DYSF;DYS	-0.0513168	-0.0927314570722934	0.0975440257156536
**cg21354621**	12	46384284	SFRS2IP;SFRS2IP	0.00992794	0.417690902355736	0.0975440257156536
**cg27334906**	1	100443213	SLC35A3;SLC35A3;SLC35A3	-0.0365914	-0.0581693125454442	0.0975440257156536
**cg00371926**	18	55289231	NARS	0.0157407	0.32364462994798	0.0975440257156536
**cg03185820**	7	149158252	ZNF777	0.0108509	0.404633088642033	0.0975440257156536
**cg18586919**	19	40005649	SELV	0.00367227	0.282032141642873	0.0975440257156536
**cg01810763**	12	104683571	TXNRD1;TXNRD1;TXNRD1;TXNRD1;TXNRD1	-0.0628681	-0.147850082562503	0.0975440257156536
**cg20018267**	7	138145918	TRIM24;TRIM24	0.00516847	0.486313383749317	0.0975553260361899
**cg17050724**	17	79008885	FLJ90757;BAIAP2;BAIAP2;BAIAP2;BAIAP2	0.026829	0.334412652985919	0.097598251185222
**cg23004862**	5	175669020	C5orf25	-0.0314354	-0.0501708946274056	0.097598251185222
**cg24123355**	14	102904584	TECPR2	0.0276512	0.601400500479099	0.097598251185222
**cg03210277**	1	26233332	STMN1;STMN1;STMN1;STMN1;STMN1	0.0126971	0.37569859933908	0.0976239424882612
**cg07808805**	13	52487022		-0.0307314	-0.0599892429064045	0.0976239424882612
**cg10204807**	1	234350515	SLC35F3	0.00905716	0.443365298760855	0.0976239424882612
**cg16858898**	1	15479872	TMEM51;TMEM51;TMEM51;TMEM51;C1orf126	0.0184512	0.480361694204561	0.0976239424882612
**cg25048357**	11	118955763	HMBS	0.0241236	0.692179451615493	0.0976239424882612
**cg05694921**	1	29587117	PTPRU;PTPRU;PTPRU	0.0366123	0.390887880864671	0.0976239424882612
**cg03115081**	17	74722049	C17orf95;JMJD6;JMJD6	0.00561189	0.450902421261896	0.0976239424882612
**cg00494761**	2	86564659	REEP1;REEP1;REEP1;REEP1;REEP1;REEP1;REEP1	0.0188873	0.50760807493419	0.0976239424882612
**cg13385156**	4	1750374		0.00844176	0.306565516330667	0.0976239424882612

**cg12938425**	14	104605300	KIF26A	0.0143426	0.476803165237093	0.0976441713770261
**cg06388206**	1	85514343	MCOLN3;MCOLN3	0.00879066	0.440562071098612	0.0976441713770261
**cg01399981**	16	87812658		0.0127438	0.478569855373634	0.0976441713770261
**cg14905657**	12	16760048	LMO3;LMO3	-0.0383318	-0.131198565108907	0.0976441713770261
**cg25754449**	8	145670366	TONSL	0.0274462	1.21988740710483	0.0976461934558231
**cg05673534**	2	233750541	NGEF;NGEF	-0.0479674	-0.0796951749374154	0.0976461934558231
**cg25496627**	7	123177030		-0.0597465	-0.109404344639498	0.0976461934558231
**cg03760387**	16	19566823	C16orf62	0.00992996	0.389857986142347	0.0976461934558231
**cg02584459**	10	91295421	SLC16A12	0.0224261	0.405364921899116	0.0976461934558231
**cg10087092**	20	62711744	RGS19;OPRL1;RGS19;OPRL1	0.0116992	0.31441321874429	0.0976461934558231
**cg17564074**	11	66080543		0.010489	0.476231687445197	0.0976461934558231
**cg10583414**	12	48207063	HDAC7;HDAC7	0.00994914	0.581065008167342	0.0976461934558231
**cg16854524**	4	83931902	LIN54;LIN54;LIN54;LIN54;LIN54	0.0221565	0.461918740509557	0.0976461934558231
**cg18934409**	19	35167332	ZNF302;ZNF302	-0.00984019	-0.0148371223954837	0.0976461934558231
**cg24953506**	1	12079889	MIIP	0.0148162	1.18016546348556	0.0976461934558231
**cg11394151**	11	2372295		-0.0577189	-0.0930101844864254	0.0976461934558231
**cg07129595**	6	116883961		-0.0514945	-0.0976630696546289	0.0976461934558231
**cg13405423**	8	42011090	AP3M2;AP3M2	0.00637675	0.438228997498351	0.0977157487500235
**cg15387286**	7	81638429	LOC101927356;LOC101927356;CACNA2D1	-0.0536388	-0.0994674103882199	0.0977157487500235
**cg09881865**	19	46234197	FBXO46	0.00737296	0.440889642758671	0.0977157487500235
**cg19011063**	5	170171465		0.0180182	0.701871275646364	0.0977157487500235
**cg16372275**	3	183856658	EIF2B5	-0.0381253	-0.0620868856214026	0.0977157487500235
**cg19406040**	1	110527169	AHCYL1	0.0315326	0.633643556181958	0.0977684750089273
**cg21235465**	18	48509680	ELAC1	-0.0445405	-0.0732035881537534	0.0977684750089273
**cg11674373**	10	7708997	ITIH5;ITIH5	0.0157828	0.594126183932058	0.0977684750089273
**cg09091206**	11	57508985	C11orf31;C11orf31;TMX2-CTNND1	0.00551939	0.359904170562092	0.0977684750089273
**cg14743055**	21	43430222	ZNF295;ZNF295;ZNF295	0.0123738	0.477671086856557	0.0977684750089273
**cg00991794**	6	125284212	STL	0.0158516	0.695123254600792	0.0977684750089273
**cg16761035**	7	94285397	SGCE;PEG10;SGCE;PEG10;SGCE	0.0580134	0.736944099320171	0.0977684750089273
**cg26407504**	2	96068329	FAHD2A	0.00673283	0.492357633110451	0.0977684750089273
**cg02756152**	11	62559437	TMEM223	0.00683809	0.467030994708532	0.0977684750089273
**cg01900557**	2	56152001	EFEMP1;EFEMP1	-0.0400659	-0.0665184442333771	0.0977838276877468
**cg11732855**	17	4046412	ZZEF1;CYB5D2;CYB5D2;CYB5D2	0.00676693	0.450659717797602	0.0977838276877468
**cg22502962**	2	95652521		-0.0334753	-0.0635722171912413	0.0977838276877468
**cg17713864**	17	48784765	ANKRD40	0.00969248	0.350273870691383	0.0977838276877468
**cg27100316**	1	151735804	OAZ3;MRPL9	0.0060594	0.473503322826994	0.097815661138673
**cg00449899**	8	53626436	RB1CC1;RB1CC1	0.022969	0.788588523600642	0.097815661138673
**cg11691170**	11	130318691	ADAMTS15	0.0360962	0.615855345901301	0.097815661138673
**cg08343754**	5	73980834	HEXB	0.0192762	0.458466082694391	0.097815661138673
**cg16975576**	17	17991081	DRG2	0.0280583	0.780574909758507	0.097815661138673
**cg18493207**	9	107526321	NIPSNAP3B;NIPSNAP3B;NIPSNAP3B;NIPSNAP3B	0.0238563	0.591919935833236	0.097815661138673
**cg25103172**	2	95825271	ZNF514	0.0141509	0.634148577854232	0.097815661138673
**cg10233133**	1	227505598	CDC42BPA;CDC42BPA;CDC42BPA;CDC42BPA	0.00461123	0.341542512925828	0.097815661138673
**cg06364629**	2	27592939	EIF2B4;SNX17;EIF2B4;EIF2B4	0.0178092	0.675261238729145	0.097820822918348
**cg02701615**	3	14755338	C3orf20	-0.0108551	-0.0165263815533295	0.0978872819582686
**cg00190900**	17	71170372		0.00455054	0.00668951244955317	0.0978872819582686
**cg03787210**	8	141521272	CHRAC1;CHRAC1;CHRAC1	0.0174504	0.806404452059039	0.0978872819582686
**cg17400229**	21	44527180	U2AF1;U2AF1;U2AF1	0.00752061	0.519399542002839	0.0978872819582686
**cg20166204**	3	67048633	KBTBD8	0.00773804	0.471595554879592	0.0978872819582686
**cg11777782**	19	18284937	IFI30	0.0484517	0.514582069524087	0.0978872819582686
**cg07636095**	6	27759817		0.0267814	0.748419333041545	0.0978872819582686
**cg13180110**	8	9008352	PPP1R3B;PPP1R3B	0.00626454	0.339830098614728	0.0978872819582686
**cg21897413**	5	176944084	DDX41	0.0131023	0.782273282571863	0.0979230651306459
**cg14432797**	20	44261290	WFDC9	-0.0300489	-0.0511794693938904	0.0980040271242102
**cg01956354**	22	38349940	C22orf23;POLR2F	0.0271135	0.493527413960874	0.0980097805923784
**cg14809705**	17	33814817	SLFN12L	0.00930775	0.297255924317332	0.0980097805923784
**cg04887328**	17	16945603	MPRIP;MPRIP	0.0120798	0.590286579507277	0.0980097805923784
**cg02985139**	13	112620260		-0.0363171	-0.0617626461326118	0.0980097805923784
**cg16751764**	10	82168743	C10orf58	0.0405427	0.629161551269719	0.0980097805923784
**cg07575812**	5	138883510		-0.0211669	-0.0323649737530469	0.0980097805923784
**cg23959009**	4	83352458	ENOPH1;HNRPDL;HNRPDL	0.0125764	0.666716606817786	0.0980588806849051
**cg24705286**	16	27215306	JMJD5;JMJD5;JMJD5	0.0380714	0.978074698512519	0.098078722609974
**cg21108767**	7	99933721	PILRB;PMS2L1;PILRB	0.0273125	1.26015774862003	0.098078722609974
**cg10334739**	11	118376683	KMT2A;KMT2A	0.00669584	0.00994626860398026	0.098078722609974
**cg03877058**	7	129094356	STRIP2;STRIP2;STRIP2;STRIP2	-0.041621	-0.0656795973833229	0.098078722609974
**cg17130567**	12	66218057	HMGA2;RPSAP52;HMGA2	0.0356145	0.679276316219966	0.0980897710698431
**cg23526204**	20	43992176	SYS1;SYS1-DBNDD2	0.0470537	0.616246463468421	0.0980928387071715
**cg14805303**	11	118984815	C2CD2L;C2CD2L	0.00834282	0.012585101976224	0.0980928387071715
**cg08317416**	19	52703857	PPP2R1A;PPP2R1A	-0.070843	-0.129948577379242	0.09810412527671
**cg05474467**	6	38608039	BTBD9;BTBD9	0.00685071	0.318817054935414	0.09810412527671
**cg07818869**	11	6495247	TRIM3;TRIM3	0.0373626	0.333774190594524	0.09810412527671
**cg04801971**	19	46011889	VASP	0.00378615	0.329926442841641	0.09810412527671
**cg16308174**	11	70049109	FADD	0.00921498	0.546933190312059	0.09810412527671
**cg18069309**	15	42867567	STARD9	0.00427771	0.365809771480021	0.0981084190236303
**cg08730330**	17	61904790	PSMC5;FTSJ3;FTSJ3	0.0285435	0.770383447531305	0.0982051247094847
**cg14848284**	2	73143293	EMX1	0.00733189	0.668573966815062	0.0982051247094847
**cg22448601**	2	220253383	DNPEP	0.014608	0.567459447260103	0.0982051247094847
**cg20190456**	17	1897953	RTN4RL1	-0.0460671	-0.0893062703107666	0.0982097293960118
**cg17431657**	7	99528556	GJC3	0.00483417	0.00710707452461653	0.0982097293960118
**cg22373392**	17	45973408	SP2	0.00281329	0.399391846056694	0.0982394369804626
**cg04242499**	5	140749745	PCDHGA4;PCDHGB3;PCDHGA1;PCDHGA5;PCDHGB1;PCDHGA3;PCDHGB3;PCDHGA	-0.0440035	-0.479731470238667	0.0982469738601786
**cg00177632**	9	34380890	C9orf24;C9orf24;C9orf24;C9orf24;C9orf24;C9orf24;C9orf24	0.00480135	0.303926680718537	0.0982469738601786

**cg07058132**	16	1755945	MAPK8IP3;MAPK8IP3	0.0262478	0.650846315499682	0.0982469738601786
**cg17347396**	1	54327155	YIPF1;YIPF1;YIPF1	0.00330708	0.00485904075835801	0.0982469738601786
**cg10337377**	16	55746561		0.0234904	0.542263013665906	0.0982469738601786
**cg26577993**	16	89993032	TUBB3	0.0323439	0.341600547446871	0.0982469738601786
**cg06600200**	19	49455351		-0.0496726	-0.0959773657583543	0.0982469738601786
**cg06890291**	7	1542315	INTS1	-0.0194055	-0.0298323713664546	0.0982528587619489
**cg27159429**	3	160938964	NMD3	0.00585331	0.496066668766122	0.0982543837110801
**cg08869383**	1	11865661	CLCN6;CLCN6;MTHFR;CLCN6;CLCN6	0.0097274	0.46374576588013	0.0982543837110801
**cg13942312**	8	1995593	MYOM2	0.0105906	0.0161866639382423	0.0982743841251369
**cg18539474**	1	113933413	MAGI3;MAGI3	0.00665293	0.474910578183164	0.0982743841251369
**cg23816619**	15	78730509	IREB2	0.018859	1.22269644770086	0.0982743841251369
**cg10437652**	5	180288206	ZFP62	0.0256684	0.360912162548855	0.0982748031925757
**cg09620687**	11	109567254		0.00570735	0.00845693843058228	0.0982797018333889
**cg02604839**	5	52897443	NDUFS4	-0.0819162	-0.159481440524439	0.0982909585878593
**cg04426620**	10	977622		0.00812607	0.50814813198709	0.0982955707809096
**cg01457653**	16	67193218	FBXL8;TRADD	0.00462134	0.42416055544505	0.0982955707809096
**cg04292718**	1	208084456	CD34;CD34;CD34;CD34	0.0154969	0.798062367303037	0.0983079262790802
**cg21712729**	17	41910036	MPP3;MPP3	0.0130015	0.409375741135933	0.0983079262790802
**cg07899077**	22	38083744	NOL12	-0.0396866	-0.0678143172758719	0.0983373346706227
**cg05338317**	19	10679934	CDKN2D;CDKN2D	-0.003594	-0.351711201658899	0.0983373346706227
**cg14258555**	19	50180725	PRMT1;PRMT1;PRMT1	0.0354821	1.46313527488925	0.0983373346706227
**cg04063194**	19	45596745		0.0212776	0.678777386781506	0.0983373346706227
**cg26963797**	16	51189291		0.0221837	0.304680093983757	0.0983373346706227
**cg25951811**	14	96858416	AK7	0.0625724	0.53617130772347	0.0983373346706227
**cg03726000**	16	19134308		-0.0130786	-0.0195880370943582	0.0983373346706227
**cg09281539**	20	20693126	RALGAPA2;RALGAPA2	0.034814	0.729374576690242	0.0983373346706227
**cg21010407**	1	46955679		0.0330698	0.373701110977802	0.0983373346706227
**cg06081517**	21	45553342	C21orf33;C21orf33	0.00482945	0.526443231172569	0.0983373346706227
**cg21679169**	1	150518766		-0.0343308	-0.0531983909001674	0.0983373346706227
**cg01419479**	1	182360822	GLUL;GLUL;GLUL;GLUL;GLUL	0.00560443	0.595856341239986	0.0983373346706227
**cg26497076**	12	122512410		-0.0760371	-0.155679331091439	0.0984214417279196
**cg26483432**	1	149815043	HIST2H2AA3;HIST2H2AA4	0.0174769	0.665461584885931	0.0984214417279196
**cg09053907**	4	75720055	BTC	0.033925	0.48625990959119	0.098480950324491
**cg23994051**	10	112432175	RBM20	0.0389447	0.839766933560305	0.098480950324491
**cg06514344**	19	17905835	B3GNT3	0.0139344	0.461715683829604	0.098480950324491
**cg12164321**	10	125651085	CPXM2	0.0501537	0.522478882494707	0.098480950324491
**cg00115180**	11	61196867	SDHAF2;CPSF7;CPSF7;CPSF7	0.00898039	0.45436995445411	0.098480950324491
**cg19060203**	11	70150660	PPFIA1;PPFIA1	-0.0385348	-0.0618034776069273	0.0984899315378517
**cg03242101**	16	67143792	C16orf70	0.02512	0.621690115467872	0.0984899315378517
**cg20720059**	2	14772731	FAM84A	0.00712103	0.558730923509042	0.0984899315378517
**cg25090569**	5	156974558	ADAM19	-0.0331264	-0.0526559797522243	0.0984899315378517
**cg10326891**	4	88343821	NUDT9;NUDT9;NUDT9;NUDT9	0.049462	0.621280204215685	0.0984899315378517
**cg21158896**	11	17099219	RPS13;RPS13	0.00532262	0.457109889110886	0.0984899315378517
**cg22597783**	1	51984800	EPS15	0.023595	0.51149764354938	0.0984899315378517
**cg09979728**	19	41903455	BCKDHA;EXOSC5;BCKDHA	0.00580079	0.482467153880986	0.0984899315378517
**cg08278741**	19	36736477		0.0367747	0.616365466721377	0.0984899315378517
**cg22279504**	16	54319404	IRX3	0.0069287	0.336818767159038	0.0984899315378517
**cg26599542**	14	31028234	G2E3;G2E3	0.026153	0.577428977529758	0.0984899315378517
**cg06322891**	1	23504425		0.0222138	0.592722093894623	0.0984899315378517
**cg01110356**	6	27357008	ZNF391	0.00563235	0.389198764908254	0.0984899315378517
**cg10194352**	16	55422784		-0.101896	-0.211275659787828	0.0984899315378517
**cg05874561**	4	154709828	SFRP2	0.0127746	0.549731399641274	0.0984899315378517
**cg16579347**	3	155572233	SLC33A1	0.0101085	0.671177399482868	0.0984899315378517
**cg04569888**	16	30538518	ZNF768	0.0158676	0.608996042999733	0.0984899315378517
**cg12221212**	1	156474724		0.010503	0.518059986480234	0.0984899315378517
**cg13788479**	15	76005247	CSPG4	0.00847249	0.323053155522092	0.0984899315378517
**cg02505554**	1	202779959	PCAT6;PCAT6	0.0144835	0.508949477797908	0.0984899315378517
**cg00939627**	16	4994685		-0.0670682	-0.155990983492983	0.0984899315378517
**cg13564289**	20	62680977	SOX18;SOX18	0.00608998	0.293569692123539	0.0984899315378517
**cg07773137**	14	73603071	PSEN1;PSEN1	0.029442	0.875247426581122	0.0984899315378517
**cg14468408**	9	128719060	PBX3;PBX3;PBX3;PBX3	-0.014433	-0.0215903325977921	0.0984899315378517
**cg21862100**	22	40102855		-0.0358871	-0.0638126827346998	0.0984899315378517
**cg25507508**	2	59661318		-0.023918	-0.0368484033321315	0.0984899315378517
**cg18481187**	6	153472898		-0.0230362	-0.037205914327011	0.0984899315378517
**cg05381183**	1	162025719		-0.0505725	-0.106931675710062	0.0984899315378517
**cg20493469**	17	76626198	LOC101928710;LOC101928710	0.00825715	0.0122819945710048	0.0984899315378517
**cg04197632**	2	11566584		-0.00991364	-0.0148058392108325	0.0984899315378517
**cg03095800**	16	67208728	NOL3	0.0436253	0.286441104663336	0.0984899315378517
**cg06409977**	17	61920084	SMARCD2;SMARCD2	0.00849433	0.472060801677237	0.0984899315378517
**cg12198977**	2	38623389		-0.0198279	-0.0320517136929156	0.0984899315378517
**cg22309051**	8	26870611		-0.0614191	-0.1200047918939	0.0984899315378517
**cg21722700**	1	54691740	SSBP3;SSBP3;SSBP3	0.00221818	0.00327208660056189	0.0984899315378517
**cg09989644**	1	156358119		0.0395753	0.514127105225022	0.0984899315378517
**cg24826645**	13	113952209	LAMP1	0.0114251	0.501026740823669	0.0984899315378517
**cg09690659**	20	33680871	TRPC4AP;TRPC4AP	0.0188452	0.79145968230665	0.0984899315378517
**cg10235961**	3	48471218	PLXNB1;PLXNB1	0.0130617	0.431471322167486	0.0984899315378517
**cg16790682**	12	57984821	PIP4K2C;PIP4K2C;PIP4K2C;PIP4K2C	0.0088776	0.526896735027454	0.0984899315378517
**cg14615342**	8	81451707		-0.056525	-0.0964807480654003	0.0984899315378517
**cg23048660**	19	45004795	ZNF180	0.0127622	0.418858062994796	0.0984899315378517
**cg03655683**	2	63281383	OTX1	0.0154418	0.37780563594026	0.0984899315378517
**cg05939335**	9	124132520	STOM;STOM;STOM;STOM	0.0231994	0.574146584490963	0.0984899315378517
**cg25751198**	12	109729573	FOXN4	0.0250768	0.51155921363405	0.0984899315378517

**cg00659495**	12	54520336	LOC400043	0.0151259	0.546195314973128	0.0984899315378517
**cg09090048**	11	134093731	VPS26B;NCAPD3	0.0510428	0.450985233986898	0.0984899315378517
**cg12307014**	12	51403966	SLC11A2;SLC11A2;SLC11A2;SLC11A2;SLC11A2;SLC11A2;SLC11A2;SLC11A2;SLC11A2	-0.0388877	-0.0651218439227533	0.0984899315378517
**cg01671285**	1	17765232	RCC2;RCC2	0.00957776	0.571452680945267	0.0984899315378517
**cg24609530**	11	18610297	UEVLD;UEVLD	0.0410615	0.751922383206929	0.0984899315378517
**cg11973777**	8	8244085		0.0301427	0.478877691480675	0.0984899315378517
**cg21833572**	1	3817804	LOC100133612;C1orf174	0.00682328	0.518899912095351	0.0984899315378517
**cg13460465**	19	12572864	ZNF709;ZNF709	-0.06971	-0.124543660459304	0.0984899315378517
**cg01118321**	1	45666712	ZSWIM5	-0.0667172	-0.111905885361132	0.0984899315378517
**cg02397124**	6	33168279	SLC39A7;SLC39A7;RXRB;RXRB	0.00861449	0.565927885799044	0.0984899315378517
**cg05171921**	7	157917118	PTPRN2;PTPRN2;PTPRN2	-0.0496632	-0.0893271997390624	0.0985345873976943
**cg00469795**	21	38445171	PIGP;TTC3;PIGP;PIGP	0.0122914	0.492936158673367	0.0985345873976943
**cg18951977**	14	54421397	BMP4;BMP4;BMP4	0.013823	0.516556719976504	0.0985345873976943
**cg08980837**	1	91172337		0.00528396	0.372765829826136	0.0985579136413622
**cg14789178**	19	5904119	VMAC;NDUFA11	0.0398405	0.611789564362576	0.0986085145760862
**cg00427086**	15	52861396	ARPP19	0.00963449	0.536935212788883	0.0986116260758758
**cg21157107**	9	110399894		0.0409743	0.254084751954934	0.0987080432217433
**cg05888406**	7	5280109		0.00558277	0.424268555668805	0.0987080432217433
**cg20015921**	15	41099716	ZFYVE19;ZFYVE19;DNAJC17	0.019997	0.66125162821626	0.0987402556106472
**cg14094277**	2	159036756	CCDC148-AS1;CCDC148;CCDC148	-0.0497779	-0.0847653354862372	0.0987402556106472
**cg13519464**	19	9938554	UBL5;UBL5	0.0128058	0.766657636444562	0.0987402556106472
**cg14500801**	4	49204696		-0.0865527	-0.247483467862058	0.0987402556106472
**cg23098139**	2	10260288		0.00867127	0.370189792923093	0.0987702007499148
**cg10865368**	19	36134154	ETV2	0.0142072	0.570958201769915	0.0988011164590027
**cg25671400**	1	100502496	HIAT1	-0.0244914	-0.0374829052036782	0.0988080640647954
**cg09551996**	11	69455530	CCND1	0.0186677	0.359207747710757	0.0988277938268281
**cg18275495**	19	10527334	PDE4A	0.0148141	0.64175737854565	0.0988277938268281
**cg08786822**	4	3109268	HTT	-0.00506059	-0.00743983167498056	0.0988277938268281
**cg21737024**	16	30441289	DCTPP1	-0.00153076	-0.202971996625228	0.0988277938268281
**cg21778810**	5	139493680	PURA	0.0205365	0.464360876927842	0.0988277938268281
**cg01929065**	12	56122634	CD63;CD63	0.00477876	0.47225113817084	0.0989375059087766
**cg06730340**	3	9811714	CAMK1	0.0337656	0.604979120939132	0.0989375059087766
**cg12093371**	10	130339316		0.00835792	0.349717846352686	0.0989375059087766
**cg12641569**	11	70601781	SHANK2	0.0121834	0.740695322396621	0.0989375059087766
**cg05341610**	19	57701430	ZNF264	-0.103669	-0.248372867126258	0.098944927788196
**cg11797226**	17	27028982	SUPT6H	-0.0122184	-0.0181266011752784	0.099016272604799
**cg26562592**	1	1590640	CDK11B;CDK11B;CDK11B;CDK11B;CDK11B;CDK11B	0.0119676	0.598257291808503	0.099016272604799
**cg21634331**	19	12985203	MAST1	-0.0211825	-0.0326818945832885	0.099016272604799
**cg03443205**	2	43454133	LOC100129726;ZFP36L2	0.0323977	0.900143148758869	0.0990761038409221
**cg18430990**	1	1475941	TMEM240	0.0288198	0.746508373192493	0.0990766729759953
**cg01116831**	16	2190356		0.00774247	0.0117584715187305	0.0990766729759953
**cg12251760**	9	130548442	CDK9	0.0282342	1.02925953196074	0.0990766729759953
**cg19160520**	1	226927217	ITPKB	-0.0347329	-0.448811447763962	0.0990766729759953
**cg16446310**	19	56805883		-0.0145147	-0.0216292986543507	0.0990766729759953
**cg06807960**	7	26904159	SKAP2;SKAP2	0.0202266	1.0046874200632	0.0990766729759953
**cg00599043**	2	85876697		0.00464391	0.00687839292839026	0.0990766729759953
**cg06517246**	11	5267724	BGLT3	-0.0474465	-0.0956483168038226	0.0990766729759953
**cg05719720**	13	25254963	ATP12A	0.023533	0.6275385737077	0.0990766729759953
**cg26283170**	11	132951950	OPCML	0.0642116	0.184799850575548	0.0990766729759953
**cg11469822**	12	6493196	LTBR;LTBR	0.0218722	0.452578419105098	0.0990766729759953
**cg00898282**	9	111775915	CTNNAL1;CTNNAL1	0.0290221	0.496739702964915	0.0990766729759953
**cg20060451**	5	179159945	MAML1;MAML1	0.00653799	0.529823790404906	0.0990766729759953
**cg20154657**	20	49126819	PTPN1	0.00659011	0.85891233657342	0.0990766729759953
**cg22729730**	5	6687387		0.0282665	0.557823991867032	0.0990766729759953
**cg13747962**	4	75720334	BTC	-0.0128824	-0.0197069396377647	0.0990766729759953
**cg03606214**	15	21135690	NF1P1	-0.101749	-0.208615386626829	0.0990766729759953
**cg00809934**	17	73201268	NUP85;NUP85	0.00812321	0.516502734581856	0.0990766729759953
**cg19465165**	2	131130103	PTPN18;PTPN18	0.0106552	0.343130707319324	0.0990766729759953
**cg09172423**	11	62446602	UBXN1	0.00643752	0.534175056499242	0.0990766729759953
**cg10684815**	11	9336354	TMEM41B;TMEM41B;TMEM41B	0.00751136	0.423748280123725	0.0991048244674048
**cg13884470**	9	86595151	HNRNPK;HNRNPK;HNRNPK;RMI1;HNRNPK	0.0356939	0.249161515299219	0.0991689591638574
**cg26121782**	11	8284312	LMO1	0.0256703	0.88052352148946	0.0991689591638574
**cg19774424**	11	63439334	ATL3;ATL3;ATL3	0.013447	0.699757761860469	0.0991689591638574
**cg24688471**	1	2542815	MMEL1	0.0353626	0.205121262029394	0.0991689591638574
**cg27359308**	2	122043034	TFCP2L1	0.0315067	0.495476125500347	0.0991689591638574
**cg23680535**	6	36900905		-0.0156079	-0.0234652478407269	0.0991689591638574
**cg10243684**	6	142393383		-0.035275	-0.0745932415358243	0.0991689591638574
**cg12100751**	1	109203672	C1orf59;C1orf59;C1orf59	0.0129724	0.533284770918152	0.0991689591638574
**cg12585516**	4	83123547		-0.0285275	-0.0450898488557602	0.0991689591638574
**cg11150308**	17	74068434	SRP68	0.0205361	0.534794055850181	0.0991689591638574
**cg08739576**	7	44144360	AEBP1	0.0223549	0.586203372565199	0.0991689591638574
**cg11175310**	10	16859107	RSU1;RSU1	0.00889485	0.678698005365849	0.0991689591638574
**cg12266953**	2	20866261	GDF7	0.00639644	0.408538947145683	0.0991689591638574
**cg22344578**	8	95908002	CCNE2	0.0107145	0.419141092024226	0.0991689591638574
**cg22674717**	16	51185394	SALL1;SALL1	0.00820489	0.364504818422306	0.0991689591638574
**cg23408670**	17	36831499	C17orf96	0.00857806	0.574657493655681	0.0991689591638574
**cg04942472**	16	58497239	NDRG4;NDRG4	0.0159541	0.425156607160078	0.0991689591638574
**cg08747717**	16	50186775	PAPD5;PAPD5	0.0177539	0.503206645170769	0.0991689591638574
**cg06641959**	16	27325254	IL4R;IL4R;IL4R;IL4R	0.00599691	0.373176340563501	0.0991689591638574
**cg25722431**	7	98630446	LOC101927550;SMURF1;SMURF1;SMURF1	-0.0490116	-0.0795628058422907	0.0991689591638574
**cg11691298**	2	26396787	FAM59B	0.0346792	0.422705371905314	0.0991689591638574
**cg08154405**	1	156164745	SLC25A44;SLC25A44;SLC25A44;SLC25A44;SLC25A44	-0.0252631	-0.0397233402141699	0.0991689591638574

**cg22112712**	4	113557475	ZGRF1;LARP7;LARP7;LARP7;LARP7	-0.0470619	-0.445882390134789	0.0991689591638574
**cg00697301**	12	19936477		0.0530278	0.0876931967080422	0.0992094950588176
**cg01545055**	16	67285131	SLC9A5	-0.00928939	-0.0137893819058941	0.0992094950588176
**cg19129369**	4	155665249	LRAT;LRAT	0.0139727	0.373410614793817	0.0992343573792058
**cg02207312**	11	60674164	PRPF19	-0.00188888	-0.286067679677004	0.0992435935047242
**cg11677933**	5	96564720		-0.0511845	-0.0892527538861672	0.0992435935047242
**cg20196129**	10	31609347	ZEB1;ZEB1;LOC220930;ZEB1;ZEB1;ZEB1	0.00994259	0.455167387767737	0.0992597764935839
**cg20688967**	10	70587120	STOX1;STOX1;STOX1;STOX1;STOX1	0.0118336	0.635754461413949	0.0992597764935839
**cg22762076**	19	7069572	ZNF557;ZNF557;ZNF557;ZNF557;ZNF557;ZNF557	0.019116	0.741784124980551	0.0992597764935839
**cg25773620**	3	38071309	PLCD1	0.0161618	0.44595035371771	0.0992597764935839
**cg15980735**	7	44223845	GCK	-0.0379078	-0.0653541094412472	0.0992626452446087
**cg11793978**	5	135188477	LOC153328	-0.020547	-0.0319941756107149	0.0992995643413182
**cg21715802**	8	126424176		-0.0213671	-0.0327458876346788	0.0993063564372709
**cg09424415**	7	23530240	RPS2P32	0.036931	0.179187567108445	0.0993898711611646
**cg24828989**	17	9579648	USP43;USP43	-0.0442427	-0.0769415166661339	0.0994155319490146
**cg22353818**	14	104095074	KLC1;KLC1;KLC1	0.00815675	0.5217800145265	0.0994756168685916
**cg16838574**	3	17784164		0.0324812	0.26125324718752	0.0994756168685916
**cg26199906**	8	145106246	OPLAH	0.027582	0.61689634992405	0.0994756168685916
**cg08042975**	2	213402433	ERBB4;ERBB4	0.0302957	0.852380871850502	0.0994756168685916
**cg12494528**	4	871567	GAK	-0.00532441	-0.00783990231049153	0.0994756168685916
**cg13597632**	5	43313634	HMGCS1;HMGCS1	0.00852558	0.452109270089775	0.0994756168685916
**cg20023231**	16	22825282	HS3ST2	0.019751	0.477840980419842	0.0994756168685916
**cg27192138**	6	11160380		-0.0143822	-0.0217863179532896	0.0994756168685916
**cg14010619**	19	39612741		0.0367624	0.296486282993766	0.0994756168685916
**cg05267934**	3	142654877	LOC100507389	-0.055045	-0.108016292937743	0.0994756168685916
**cg22659936**	3	129930455	COL6A4P2	-0.0278088	-0.0429569962196857	0.0994756168685916
**cg15209489**	10	76819644	DUPD1	-0.0116261	-0.0172185616905448	0.0994756168685916
**cg23719091**	5	135468815	SMAD5-AS1;SMAD5;SMAD5;SMAD5	0.0119008	0.543786082625207	0.0994756168685916
**cg04423696**	17	45249385	CDC27;CDC27;CDC27;CDC27	-0.0814126	-0.165531665111146	0.0994756168685916
**cg02796905**	15	59063699	FAM63B;FAM63B	0.00761433	0.52339292330213	0.0994756168685916
**cg15772797**	12	123868662	SETD8	0.0256197	1.27331957151659	0.0994756168685916
**cg10437425**	11	6474577	TRIM3;TRIM3;TRIM3;TRIM3	0.0123227	0.019621979493223	0.0994756168685916
**cg14527148**	15	80216341	C15orf37;ST20	0.0320312	0.599312265175125	0.0994756168685916
**cg20082830**	2	232395355	NMUR1	0.019487	0.550927360876442	0.0994756168685916
**cg10885414**	5	138609328	MATR3;MATR3;MATR3;SNHG4;SNHG4	0.00410977	0.384514972904554	0.0994756168685916
**cg11979294**	10	103880575	LDB1	0.0335068	0.409439831366956	0.0994756168685916
**cg25340121**	19	23653200		-0.0540843	-0.0997368899406063	0.0994756168685916
**cg00345047**	6	71942797		-0.0410971	-0.0777298829826096	0.0994853966326336
**cg00010266**	8	145734519	MFSD3	0.0217618	0.917246642687987	0.0995236504861325
**cg23061046**	19	52206224	NCRNA00085	0.0443829	0.332051577885484	0.0995773411534889
**cg12981595**	17	39254427	KRTAP4-8	-0.107655	-0.190947781598371	0.0995773411534889
**cg26208419**	5	473447	SLC9A3;LOC25845	0.0154594	0.327630026168242	0.0995773411534889
**cg23378706**	5	81047486	SSBP2	0.00605003	0.336894751570249	0.0995773411534889
**cg06701285**	6	30733368		-0.0467928	-0.0918055790285471	0.0996172398450439
**cg17606115**	3	50266316	GNAI2;GNAI2	0.0107157	0.0163027787251601	0.0996172398450439
**cg12614029**	11	2160564	INS-IGF2;IGF2AS;IGF2;IGF2;IGF2AS;IGF2	0.0111487	0.377025390595103	0.0996172398450439
**cg06388809**	9	130616976	ENG;ENG;ENG;ENG	0.00800378	0.292220575714524	0.099619755791151
**cg01053943**	2	120239310	SCTR	-0.0109688	-0.0164696595146784	0.099619755791151
**cg18425254**	13	113549044		0.0241834	0.611456229749515	0.099619755791151
**cg16510127**	1	156047359	MEX3A	-0.0151837	-0.022844452336848	0.099619755791151
**cg19115393**	20	59829060	CDH4	0.0109172	0.455361685895092	0.0996227463198512
**cg03727342**	9	6758683	KDM4C;KDM4C;KDM4C;KDM4C	0.0195819	0.765052096943774	0.0996227463198512
**cg19065831**	4	48485289	SLC10A4	0.0183542	0.524609131134053	0.0996227463198512
**cg16128308**	3	49132079	QRICH1;QRICH1	0.00253456	0.294406255054472	0.0996227463198512
**cg06927064**	15	90776918	CIB1;CIB1;CIB1;GDPGP1;CIB1;CIB1;CIB1;CIB1;CIB1;CIB1;CIB1;CIB1	0.0114768	0.523515014294325	0.0996227463198512
**cg17064564**	22	29710817	RASL10A;RASL10A	0.0317613	0.599789099479894	0.0996227463198512
**cg26859862**	16	30389551	SEPT1	0.024383	0.704427672218004	0.0996227463198512
**cg14058383**	6	28603099		0.00813371	0.309854707216421	0.0996227463198512
**cg26938153**	7	137686926	CREB3L2	0.00921233	0.538779143915583	0.0996227463198512
**cg12587618**	11	1979879		-0.0307427	-0.0470587417974371	0.0996227463198512
**cg10195567**	7	72987827	TBL2	0.00299981	0.00440066328757395	0.0996227463198512
**cg01786715**	14	65007437	HSPA2;HSPA2	0.00896538	0.582575635400002	0.0996401468984323
**cg27070729**	16	28964954	NFATC2IP	-0.0127547	-0.0190508706926272	0.0996401468984323
**cg00383384**	10	57391271		0.0144551	0.532044431005638	0.0996401468984323
**cg20192527**	10	126077794		0.00845285	0.509087092261193	0.0996401468984323
**cg23758016**	17	73521635	LLGL2;LLGL2;LLGL2	0.0302499	0.630470651617981	0.0996718613274861
**cg15144793**	2	235861303	SH3BP4	0.00609464	0.317226003030101	0.0996840623901426
**cg08224569**	8	142369705	GPR20	0.0600684	0.243675829507528	0.0996840623901426
**cg08630962**	1	179262864	SOAT1	0.0103243	0.592678881776952	0.0997075159070195
**cg05370569**	16	23464705	COG7	0.0112257	0.442838186949058	0.0997075159070195
**cg13971571**	8	6693247	XKR5	0.0115334	0.384116203251004	0.0997075159070195
**cg13473179**	9	15422943	SNAPC3	0.00967267	0.597330966526156	0.0998304212779876
**cg27029077**	4	137753156		-0.0817893	-0.178104149802947	0.099931808017003
**cg03295933**	19	1479358	C19orf25	0.0270405	0.718952412802738	0.099931808017003
**cg19095784**	6	37070475		0.0144308	0.371494114327654	0.099931808017003
**cg05879919**	14	102976503	ANKRD9	0.00713512	0.648196802025723	0.099931808017003
**cg22043745**	8	11565527	GATA4;GATA4;GATA4;GATA4	0.0153138	0.373728961772489	0.0999334499174577
**cg10984017**	2	121010993	RALB	0.026858	0.798946121034588	0.0999552063464506
**cg13777717**	5	76250527	CRHBP	0.0510826	0.764089948043319	0.099970941021729
**cg11149849**	17	1064017	ABR;ABR	-0.0414173	-0.0799107351570193	0.0999948976521798
**cg11239016**	16	90085722	DBNDD1	0.00428672	0.451883392487494	0.0999948976521798
**cg00116628**	5	111755514	FLJ11235;EPB41L4A	0.0274019	0.829348661482635	0.0999948976521798

**cg20342853**	6	15245700	JARID2	0.00293526	0.289141556615811	0.0999948976521798
**cg16068620**	6	88032455	C6orf162;C6orf162;GJB7	0.015089	0.477666731731662	0.0999948976521798
**cg16001793**	1	161369711		0.0165686	0.678324330800394	0.0999948976521798
**cg00109643**	11	85780154	PICALM;PICALM;PICALM;PICALM	0.0157605	0.675879454672502	0.0999948976521798
