# Supplementary material for: Redox Imbalance and Methylation Disturbances in Early Childhood Obesity
Source: Oxid Med Cell Longev. 2021 Aug 17;2021:2207125. doi: 10.1155/2021/2207125 (PMC8387800; doi:10.1155/2021/2207125)
Supplement: Supplementary 3 — Supplementary Table 3: GO term enrichment results for biological processes. [file 2207125.f3.pdf]

GO term	Description	Observed Gene Count	Background G	Strength	FDR
GO:0006979	response to oxidative stress	10	373	1.32	2.51e-08
GO:0055114	oxidation-reduction process	13	932	1.04	2.51e-08
GO:0000096	sulfur amino acid metabolic process	5	39	2.0	7.65e-07
GO:0001666	response to hypoxia	8	288	1.34	7.65e-07
GO:0009628	response to abiotic stimulus	12	1052	0.95	7.65e-07
GO:0009636	response to toxic substance	9	468	1.18	7.68e-07
GO:0046500	S-adenosylmethionine metabolic process	4	13	2.38	7.68e-07
GO:0097237	cellular response to toxic substance	7	195	1.45	7.68e-07
GO:1901700	response to oxygen-containing compound	13	1427	0.85	7.68e-07
GO:0006790	sulfur compound metabolic process	8	343	1.26	9.32e-07
GO:0034599	cellular response to oxidative stress	7	222	1.39	1.16e-06
GO:0006555	methionine metabolic process	4	20	2.19	2.12e-06
GO:0090116	C-5 methylation of cytosine	3	3	2.89	3.36e-06
GO:0032259	methylation	7	288	1.28	5.36e-06
GO:0051186	cofactor metabolic process	8	467	1.13	6.48e-06
GO:0098869	cellular oxidant detoxification	5	86	1.66	6.92e-06
GO:0000302	response to reactive oxygen species	6	189	1.4	8.16e-06
GO:0006575	cellular modified amino acid metabolic process	6	185	1.4	8.16e-06
GO:0033554	cellular response to stress	12	1553	0.78	8.16e-06
GO:0072593	reactive oxygen species metabolic process	5	103	1.58	1.24e-05
GO:0006306	DNA methylation	4	46	1.83	2.42e-05
GO:0043066	negative regulation of apoptotic process	9	859	0.91	3.34e-05
GO:0017144	drug metabolic process	8	622	1.0	3.46e-05
GO:1901701	cellular response to oxygen-containing compound	9	896	0.9	4.14e-05
GO:0042493	response to drug	9	900	0.89	4.17e-05
GO:0050667	homocysteine metabolic process	3	13	2.26	4.17e-05
GO:0031667	response to nutrient levels	7	455	1.08	5.07e-05
GO:0006732	coenzyme metabolic process	6	297	1.2	6.52e-05
GO:0010941	regulation of cell death	11	1638	0.72	7.01e-05
GO:0051881	regulation of mitochondrial membrane potential	4	68	1.66	7.01e-05
GO:0071453	cellular response to oxygen levels	5	164	1.38	7.01e-05
GO:0006520	cellular amino acid metabolic process	6	308	1.18	7.08e-05
GO:0046655	folic acid metabolic process	3	18	2.12	7.52e-05
GO:0000303	response to superoxide	3	19	2.09	8.51e-05
GO:1905710	positive regulation of membrane permeability	3	23	2.01	0.00013
GO:0006730	one-carbon metabolic process	3	24	1.99	0.00014
GO:0019725	cellular homeostasis	8	806	0.89	0.00014
GO:0031323	regulation of cellular metabolic process	19	6082	0.39	0.00014
GO:0033273	response to vitamin	4	87	1.56	0.00014
GO:0051246	regulation of protein metabolic process	13	2668	0.58	0.00015
GO:0007584	response to nutrient	5	208	1.27	0.00016
GO:1901605	alpha-amino acid metabolic process	5	209	1.27	0.00017
GO:0019752	carboxylic acid metabolic process	8	854	0.87	0.00018
GO:0042592	homeostatic process	10	1491	0.72	0.00018
GO:0042981	regulation of apoptotic process	10	1501	0.72	0.00019
GO:0006950	response to stress	14	3267	0.53	0.00021
GO:0043200	response to amino acid	4	103	1.48	0.00022
GO:0043414	macromolecule methylation	5	229	1.23	0.00022
GO:0042743	hydrogen peroxide metabolic process	3	31	1.88	0.00023
GO:0032268	regulation of cellular protein metabolic process	12	2486	0.58	0.00037
GO:0034614	cellular response to reactive oxygen species	4	124	1.4	0.00039
GO:1901698	response to nitrogen compound	8	988	0.8	0.00042
GO:0043603	cellular amide metabolic process	7	732	0.87	0.00054
GO:0009605	response to external stimulus	11	2152	0.6	0.00055
GO:0042221	response to chemical	15	4153	0.45	0.00055
GO:0010035	response to inorganic substance	6	491	0.98	0.00056
GO:0071456	cellular response to hypoxia	4	139	1.35	0.00056
GO:0035694	mitochondrial protein catabolic process	2	5	2.5	0.00058
GO:0044281	small molecule metabolic process	10	1779	0.64	0.00063
GO:0046677	response to antibiotic	5	305	1.11	0.00068
GO:0070887	cellular response to chemical stimulus	12	2672	0.55	0.00068
GO:0035690	cellular response to drug	5	310	1.1	0.00071
GO:0046498	S-adenosylhomocysteine metabolic process	2	6	2.42	0.00072
GO:0001101	response to acid chemical	5	323	1.08	0.00083
GO:0008152	metabolic process	22	9569	0.26	0.00083
GO:0065007	biological regulation	24	11740	0.2	0.00083
GO:0010940	positive regulation of necrotic cell death	2	7	2.35	0.00089
GO:0009893	positive regulation of metabolic process	13	3280	0.49	0.00091
GO:0031060	regulation of histone methylation	3	58	1.61	0.00097
GO:0010917	negative regulation of mitochondrial membrane potential	2	8	2.29	0.0011

**GO:0043045**	DNA methylation involved in embryo development	2	8	2.29	0.0011
**GO:0010243**	response to organonitrogen compound	7	876	0.8	0.0013
**GO:0035999**	tetrahydrofolate interconversion	2	9	2.24	0.0013
**GO:1903201**	regulation of oxidative stress-induced cell death	3	65	1.56	0.0013
**GO:0045454**	cell redox homeostasis	3	68	1.54	0.0014
**GO:0051247**	positive regulation of protein metabolic process	9	1587	0.65	0.0014
**GO:0051573**	negative regulation of histone H3-K9 methylation	2	10	2.19	0.0014
**GO:0043523**	regulation of neuron apoptotic process	4	195	1.21	0.0015
**GO:0006915**	apoptotic process	7	915	0.78	0.0016
**GO:1901564**	organonitrogen compound metabolic process	16	5281	0.37	0.0016
**GO:0080090**	regulation of primary metabolic process	17	5982	0.35	0.0017
**GO:0009086**	methionine biosynthetic process	2	12	2.12	0.0018
**GO:0010942**	positive regulation of cell death	6	663	0.85	0.0020
**GO:0031325**	positive regulation of cellular metabolic process	12	3060	0.49	0.0020
**GO:0019430**	removal of superoxide radicals	2	13	2.08	0.0021
**GO:0048518**	positive regulation of biological process	16	5459	0.36	0.0022
**GO:0000098**	sulfur amino acid catabolic process	2	14	2.05	0.0023
**GO:0006534**	cysteine metabolic process	2	14	2.05	0.0023
**GO:0019372**	lipoxygenase pathway	2	14	2.05	0.0023
**GO:0048522**	positive regulation of cellular process	15	4898	0.38	0.0025
**GO:0097345**	mitochondrial outer membrane permeabilization	2	15	2.02	0.0025
**GO:0043154**	negative regulation of cysteine-type endopeptidase activity involved in apoptotic process	3	91	1.41	0.0026
**GO:0051341**	regulation of oxidoreductase activity	3	93	1.4	0.0027
**GO:0051571**	positive regulation of histone H3-K4 methylation	2	16	1.99	0.0027
**GO:0070131**	positive regulation of mitochondrial translation	2	17	1.96	0.0029
**GO:0040029**	regulation of gene expression, epigenetic	4	251	1.1	0.0031
**GO:0050794**	regulation of cellular process	22	10484	0.22	0.0031
**GO:0033189**	response to vitamin A	2	19	1.92	0.0033
**GO:0009410**	response to xenobiotic stimulus	4	262	1.08	0.0034
**GO:0048523**	negative regulation of cellular process	14	4454	0.39	0.0034
**GO:0033194**	response to hydroperoxide	2	20	1.89	0.0036
**GO:0042744**	hydrogen peroxide catabolic process	2	20	1.89	0.0036
**GO:0032270**	positive regulation of cellular protein metabolic process	8	1496	0.62	0.0039
**GO:0042542**	response to hydrogen peroxide	3	112	1.32	0.0039
**GO:0055093**	response to hyperoxia	2	21	1.87	0.0039
**GO:0051171**	regulation of nitrogen compound metabolic process	16	5827	0.33	0.0040
**GO:0048871**	multicellular organismal homeostasis	4	283	1.04	0.0042
**GO:0045814**	negative regulation of gene expression, epigenetic	3	117	1.3	0.0043
**GO:0016999**	antibiotic metabolic process	3	124	1.28	0.0049
**GO:0071496**	cellular response to external stimulus	4	305	1.01	0.0052
**GO:0051354**	negative regulation of oxidoreductase activity	2	27	1.76	0.0054
**GO:0065008**	regulation of biological quality	12	3559	0.42	0.0056
**GO:0045471**	response to ethanol	3	134	1.24	0.0059
**GO:0060255**	regulation of macromolecule metabolic process	16	6072	0.31	0.0059
**GO:1901699**	cellular response to nitrogen compound	5	568	0.84	0.0059
**GO:0006807**	nitrogen compound metabolic process	19	8349	0.25	0.0063
**GO:0043524**	negative regulation of neuron apoptotic process	3	138	1.23	0.0063
**GO:1902175**	regulation of oxidative stress-induced intrinsic apoptotic signaling pathway	2	30	1.72	0.0063
**GO:0042311**	vasodilation	2	31	1.7	0.0066
**GO:0071276**	cellular response to cadmium ion	2	31	1.7	0.0066
**GO:0010639**	negative regulation of organelle organization	4	333	0.97	0.0067
**GO:0010038**	response to metal ion	4	339	0.97	0.0071
**GO:0010821**	regulation of mitochondrion organization	3	148	1.2	0.0073
**GO:0010212**	response to ionizing radiation	3	149	1.2	0.0074
**GO:0010001**	glial cell differentiation	3	154	1.18	0.0080
**GO:1903146**	regulation of autophagy of mitochondrion	2	35	1.65	0.0080
**GO:0043412**	macromolecule modification	11	3197	0.43	0.0082
**GO:0031399**	regulation of protein modification process	8	1747	0.55	0.0084
**GO:0051129**	negative regulation of cellular component organization	5	632	0.79	0.0084
**GO:0071248**	cellular response to metal ion	3	162	1.16	0.0089
**GO:0009719**	response to endogenous stimulus	7	1353	0.61	0.0091
**GO:0051402**	neuron apoptotic process	2	40	1.59	0.0097
**GO:2000377**	regulation of reactive oxygen species metabolic process	3	169	1.14	0.0097
**GO:0009892**	negative regulation of metabolic process	10	2762	0.45	0.0098
**GO:0055086**	nucleobase-containing small molecule metabolic process	5	662	0.77	0.0098
**GO:0017001**	antibiotic catabolic process	2	42	1.57	0.0103
**GO:0032269**	negative regulation of cellular protein metabolic process	6	1014	0.67	0.0106
**GO:0048678**	response to axon injury	2	43	1.56	0.0107
**GO:1903202**	negative regulation of oxidative stress-induced cell death	2	44	1.55	0.0111
**GO:0044237**	cellular metabolic process	19	8797	0.23	0.0115
**GO:0090199**	regulation of release of cytochrome c from mitochondria	2	45	1.54	0.0115
**GO:0048872**	homeostasis of number of cells	3	193	1.09	0.0131

**GO:0010823**	negative regulation of mitochondrion organization	2	50	1.5	0.0134
**GO:0097366**	response to bronchodilator	2	50	1.5	0.0134
**GO:2000378**	negative regulation of reactive oxygen species metabolic process	2	50	1.5	0.0134
**GO:0042220**	response to cocaine	2	51	1.49	0.0136
**GO:0006749**	glutathione metabolic process	2	52	1.48	0.0141
**GO:0051187**	cofactor catabolic process	2	52	1.48	0.0141
**GO:0009890**	negative regulation of biosynthetic process	7	1501	0.56	0.0143
**GO:0031329**	regulation of cellular catabolic process	5	743	0.72	0.0143
**GO:0051173**	positive regulation of nitrogen compound metabolic process	10	2946	0.42	0.0143
**GO:0031669**	cellular response to nutrient levels	3	206	1.06	0.0146
**GO:0071495**	cellular response to endogenous stimulus	6	1106	0.63	0.0146
**GO:0031324**	negative regulation of cellular metabolic process	9	2463	0.46	0.0153
**GO:0015980**	energy derivation by oxidation of organic compounds	3	217	1.03	0.0165
**GO:0016239**	positive regulation of macroautophagy	2	59	1.42	0.0168
**GO:0090066**	regulation of anatomical structure size	4	464	0.83	0.0168
**GO:0031401**	positive regulation of protein modification process	6	1149	0.61	0.0170
**GO:0006839**	mitochondrial transport	3	223	1.02	0.0171
**GO:0010660**	regulation of muscle cell apoptotic process	2	60	1.42	0.0171
**GO:0033043**	regulation of organelle organization	6	1155	0.61	0.0171
**GO:0071230**	cellular response to amino acid stimulus	2	60	1.42	0.0171
**GO:0071704**	organic substance metabolic process	19	9135	0.21	0.0171
**GO:0006112**	energy reserve metabolic process	2	62	1.4	0.0177
**GO:0045892**	negative regulation of transcription, DNA-templated	6	1169	0.6	0.0178
**GO:0072521**	purine-containing compound metabolic process	4	478	0.82	0.0178
**GO:0010604**	positive regulation of macromolecule metabolic process	10	3081	0.4	0.0182
**GO:0071417**	cellular response to organonitrogen compound	4	485	0.81	0.0185
**GO:0051716**	cellular response to stimulus	15	6212	0.28	0.0192
**GO:0044248**	cellular catabolic process	7	1646	0.52	0.0210
**GO:0009725**	response to hormone	5	854	0.66	0.0224
**GO:0072347**	response to anesthetic	2	73	1.33	0.0224
**GO:0007568**	aging	3	255	0.96	0.0227
**GO:0050896**	response to stimulus	17	7824	0.23	0.0234
**GO:0014070**	response to organic cyclic compound	5	873	0.65	0.0242
**GO:0060047**	heart contraction	2	81	1.29	0.0266
**GO:0034641**	cellular nitrogen compound metabolic process	13	5126	0.3	0.0278
**GO:0030148**	sphingolipid biosynthetic process	2	86	1.26	0.0295
**GO:0044283**	small molecule biosynthetic process	4	569	0.74	0.0295
**GO:0010506**	regulation of autophagy	3	286	0.91	0.0299
**GO:0044238**	primary metabolic process	18	8808	0.2	0.0306
**GO:0010033**	response to organic substance	9	2815	0.4	0.0315
**GO:0021782**	glial cell development	2	91	1.24	0.0320
**GO:0097190**	apoptotic signaling pathway	3	295	0.9	0.0320
**GO:0051172**	negative regulation of nitrogen compound metabolic process	8	2307	0.43	0.0324
**GO:0010628**	positive regulation of gene expression	7	1826	0.48	0.0332
**GO:1901565**	organonitrogen compound catabolic process	5	958	0.61	0.0333
**GO:0009987**	cellular process	24	14652	0.11	0.0341
**GO:0046394**	carboxylic acid biosynthetic process	3	311	0.88	0.0359
**GO:0046916**	cellular transition metal ion homeostasis	2	99	1.2	0.0359
**GO:0045444**	fat cell differentiation	2	100	1.19	0.0362
**GO:0002262**	myeloid cell homeostasis	2	102	1.19	0.0375
**GO:0048878**	chemical homeostasis	5	995	0.59	0.0375
**GO:0071236**	cellular response to antibiotic	2	112	1.15	0.0436
**GO:0042737**	drug catabolic process	2	113	1.14	0.0442
**GO:0055082**	cellular chemical homeostasis	4	665	0.67	0.0449
**GO:0032147**	activation of protein kinase activity	3	347	0.83	0.0452
**GO:0006325**	chromatin organization	4	683	0.66	0.0484
**GO:0060359**	response to ammonium ion	2	122	1.11	0.0496
